# Development of the High-Affinity Carborane-Based Cannabinoid Receptor Type 2 PET Ligand [^18^F]LUZ5-*d*_8_

**DOI:** 10.1021/acs.jmedchem.3c00195

**Published:** 2023-03-21

**Authors:** Lea Ueberham, Daniel Gündel, Martin Kellert, Winnie Deuther-Conrad, Friedrich-Alexander Ludwig, Peter Lönnecke, Aleksandr Kazimir, Klaus Kopka, Peter Brust, Rareş-Petru Moldovan, Evamarie Hey-Hawkins

**Affiliations:** † Universität Leipzig, Faculty of Chemistry and Mineralogy, Institute of Inorganic Chemistry, Johannisallee 29, 04103 Leipzig, Germany; ‡ Helmholtz-Zentrum Dresden-Rossendorf (HZDR), Institute of Radiopharmaceutical Cancer Research, Department of Neuroradiopharmaceuticals, Research Site Leipzig, 04318 Leipzig, Germany; § Faculty of Chemistry and Food Chemistry, School of Science, TU Dresden, 01069 Dresden, Germany; ∥ The Lübeck Institute of Experimental Dermatology, University Medical Center Schleswig-Holstein, 23562 Lübeck, Germany

## Abstract

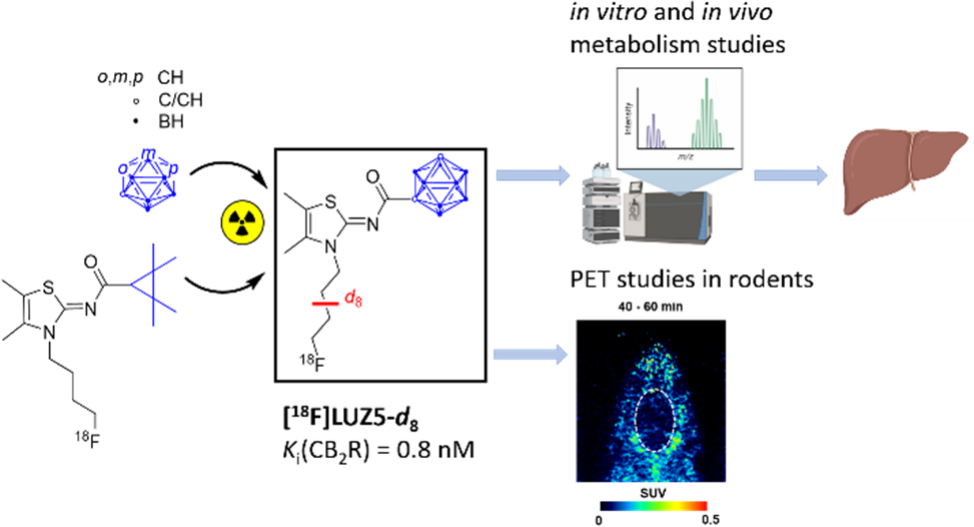

The development of cannabinoid receptor type 2 (CB_2_R) radioligands for positron emission tomography (PET) imaging was intensively explored. To overcome the low metabolic stability and simultaneously increase the binding affinity of known CB_2_R radioligands, a carborane moiety was used as a bioisostere. Here we report the synthesis and characterization of carborane-based 1,8-naphthyridinones and thiazoles as novel CB_2_R ligands. All tested compounds showed low nanomolar CB_2_R affinity, with (*Z*)-*N*-[3-(4-fluorobutyl)-4,5-dimethylthiazole-2(3*H*)-ylidene]-(1,7-dicarba-*closo*-dodecaboranyl)-carboxamide (**LUZ5**) exhibiting the highest affinity (0.8 nM). Compound **[**^**18**^**F]LUZ5-*d***_**8**_ was obtained with an automated radiosynthesizer in high radiochemical yield and purity. *In vivo* evaluation revealed the improved metabolic stability of **[**^**18**^**F]LUZ5-*d***_**8**_ compared to that of **[**^**18**^**F]JHU94620**. PET experiments in rats revealed high uptake in spleen and low uptake in brain. Thus, the introduction of a carborane moiety is an appropriate tool for modifying literature-known CB_2_R ligands and gaining access to a new class of high-affinity CB_2_R ligands, while the *in vivo* pharmacology still needs to be addressed.

## Introduction

The endocannabinoid system (ECS), named after the cannabis plant,^1^ has become the focus of medicinal research, due to its involvement in the modulation of various physiological and pathological processes.^1,2^ With the biological investigation of the main active substance in cannabis, (−)-Δ^9^-*trans*-tetrahydrocannabinol (THC), cannabinoid receptors type 1 (CB_1_R, identified in 1988, cloned in 1990) and 2 (CB_2_R, cloned in 1993) were discovered and studied.^2−7^ The endocannabinoid system is defined as the entirety of the cannabinoid receptors, the endogenous lipids, and the enzymes responsible for the synthesis and degradation thereof^8^ and is still being continuously studied. Other receptors like GPR18 and GPR55 have also been shown to be modulated by cannabinoids, despite sharing a low degree of sequence homology with the CB_1_R and CB_2_R.^9,10^

The CB_1_R and CB_2_R are part of the family of G protein-coupled receptors (GPCRs) and exhibit a degree of similarity of 44% for amino acids across the entire protein and of 68% regarding the transmembrane domains.^4,11^ The binding pockets of the CB_1_R and CB_2_R differ partly from each other.^12^ Recently, the antagonist and agonist bonding of the CB_2_R in the complex with corresponding ligands was revealed through crystal structures.^11,12^ With the knowledge of similarities and differences between the two receptors, especially the constitution and conformation of their binding pockets, the development of selective and high-affinity ligands is being actively pursued.

**Figure 1:**
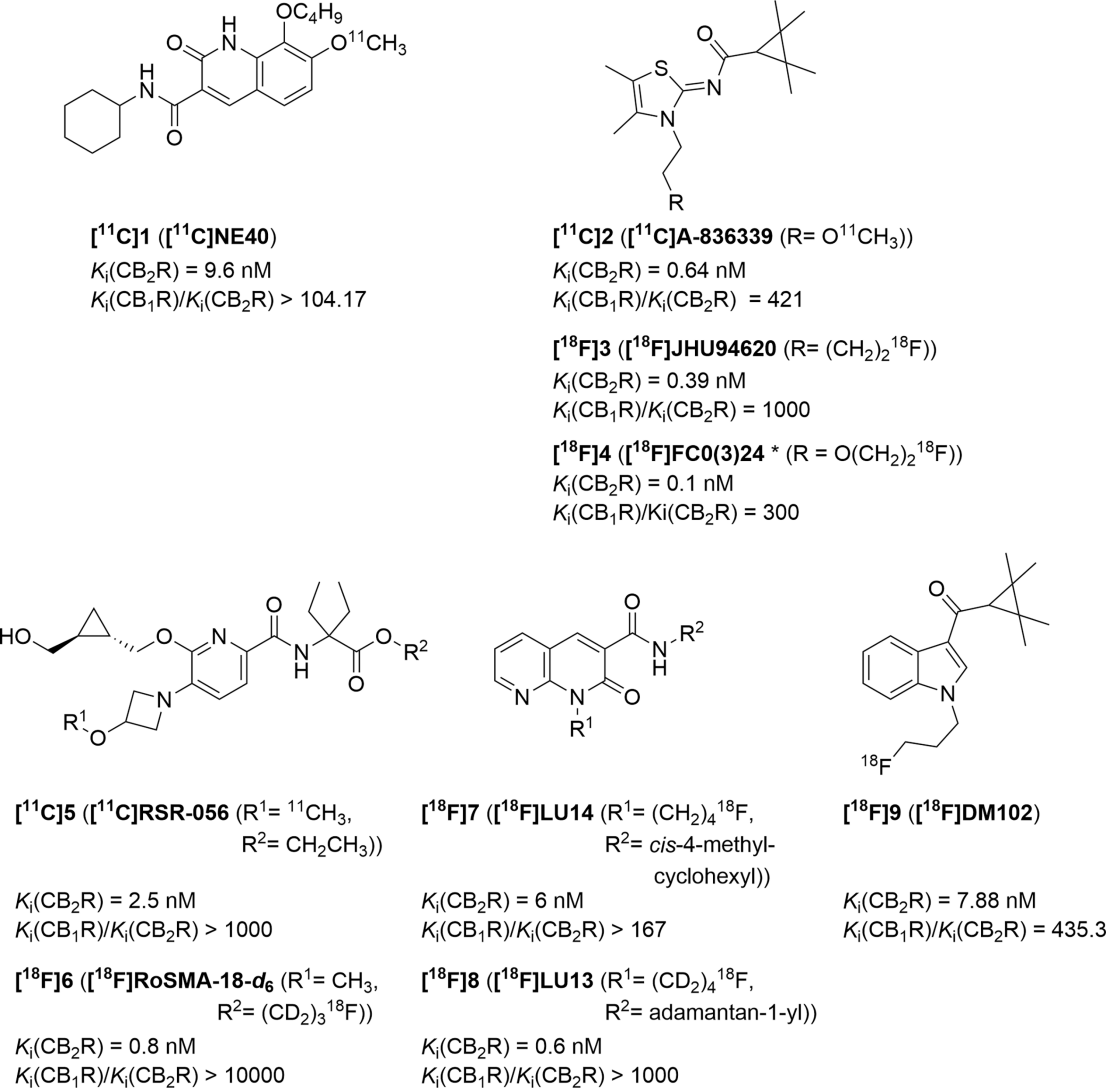
Selected radiolabeled CB_2_R ligands.^24,25,28,30−32,37−41^ *[^**18**^**F]4** was found in the literature as either [^18^F]FC024 or [^18^F]FC0324.^24,34−36^

While the CB_1_R is mainly expressed in the central nervous system (CNS), the level of expression of the CB_2_R in the brain is significantly lower under physiological conditions.^13−17^ The CB_2_R is mainly associated with the immune system and predominantly expressed among others in spleen, tonsils, and thymus.^18,19^ In the brain, the CB_2_R is overexpressed under pathological conditions like inflammation,^20^ neurodegenerative diseases, such as Alzheimer’s, Huntington’s, and Parkinson’s^21^ diseases, or cancer.^20^ The CB_2_R is also connected to rheumatoid arthritis or arteriosclerosis.^19^ The activation of the CB_2_R can lead to beneficial effects, like causing apoptosis of cancer cells.^20^ The diagnosis and treatment of the aforementioned pathological conditions could be achieved by the use of selective CB_2_R ligands. The development of such compounds is ongoing and challenging, because of the strong requirements. The affinity must be high [(sub)-nanomolar range], due to the low expression density of the CB_2_R, and the selectivity must be high (1000-fold higher than for the CB_1_R) to avoid the undesired psychedelic effects of the CB_1_R. Other important characteristics are the metabolic stability, bioavailability, and ability to penetrate membranes or the blood–brain barrier (BBB) (LogP range of 1–3.5).^15,22^ To date, no selective CB_2_R ligand is approved for routine clinical use. To gain more insights into the involvement of the CB_2_R in physiological and pathological processes and to take action for their treatment, the theranostic approach is greatly important. A suitable tool for diagnosis is positron emission tomography (PET), a non-invasive imaging method, with which biochemical features in the body can be monitored.^23^ In addition to the already mentioned requirements for suitable CB_2_R ligands, it is necessary that these compounds can be radiolabeled with a suitable radionuclide for PET. The most frequently used radioisotopes for PET are ^11^C (half-life of 20.4 min) and ^18^F (half-life of 109.8 min).^24^ For the usage of PET tracers, it is important that no radiometabolites are present in the brain and that the level of interaction with plasma proteins is as low as possible.^15^ Evens et al. developed the oxoquinoline-based compound **[**^**11**^**C]NE40** (**[**^**11**^**C]1**)^25^ (Figure 1), a CB_2_R radiotracer tested in patients with Alzheimer’s disease (AD). Whereas promising results have been obtained in healthy humans, the expected better binding of the tracer was not seen in the AD patients, which could possibly be related to the unfavorable biochemical properties of the radioligand.^26^ Other scaffolds of interest are naphthyridinones, indoles, indazoles, pyridines, oxadiazoles, carbazoles, imidazoles, thiophenes, or thiazoles.^15,27−29^ Spinelli et al. published a review in 2017 about structure–affinity and structure–activity relationships of several CB_2_R scaffolds and identified their influence and the importance of different functional groups within the structures on their suitability as diagnostic and therapeutic CB_2_R ligands.^27^ A selection of known radiolabeled CB_2_R ligands is shown in Figure 1. The thiazole-based **[**^**11**^**C]A-836339** (**[**^**11**^**C]2**) is an auspicious lead structure. The non-radiolabeled compound was synthesized by Dart et al.,^30^ further investigated by Yao et al.,^31^ and radiolabeled by Horti et al.^32^ Extensive medicinal chemistry studies performed by us [**[**^**18**^**F]JHU94620** (**[**^**18**^**F]3**)]^28,33^ and Caillé [**[**^**18**^**F]FC0(3)24**, in the literature as either **FC024** or **FC0324** (**[**^**18**^**F]4**)]^24,34−36^ led to the development of ^18^F-labeled analogues of **[**^**11**^**C]2**. While **[**^**18**^**F]3** is a low-nanomolar affinity and selective CB_2_R PET radioligand, the presence of large fractions of radiometabolites in mouse brain limits its application as a PET tracer.^28^ The preclinical evaluation of **[**^**18**^**F]4** in Rhesus monkeys also showed a high *in vivo* metabolism.^35^ The pyridine-based scaffold is also in the focus of research toward the development of a PET radioligand for CB_2_R imaging.^27,37,38^ Slavik et al. developed **[**^**11**^**C]RSR-056** (**[**^**11**^**C]5**)^37^ and Haider et al. prepared and preclinically investigated **[**^**18**^**F]RoSMA-18-*d***_**6**_ (**[**^**18**^**F]6**).^38^ Recently, Modemann et al. reported an indole-based ^18^F-labeled radioligand (**[**^**18**^**F]9**).^39^ The 1,8-naphthyridine-2-one, as another lead structure, was systematically and manifold modified in the past several years.^20,27^ We recently reported **[**^**18**^**F]LU14** (**[**^**18**^**F]7**),^40^ a stereochemically pure 1,8-naphthyridine-2-one radioligand, and **[**^**18**^**F]LU13** (**[**^**18**^**F]8**),^41^ a promising high-affinity radiotracer for targeting CB_2_R overexpression in neurological disease.

**Figure 2:**
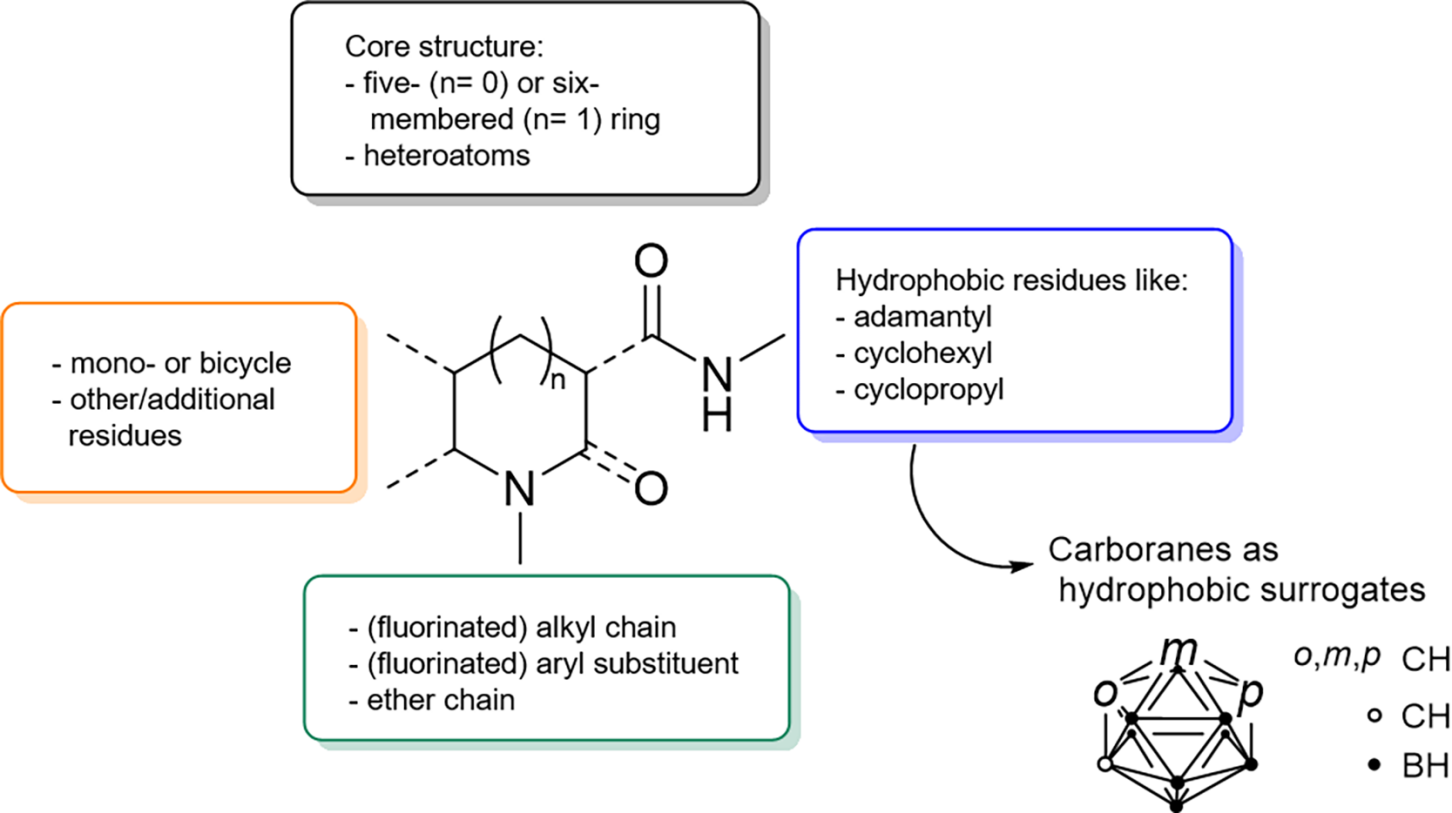
Common structural motifs for CB_2_R ligands, inspired by Spinelli et al.^27^

Common drawbacks of the reported CB_2_R radioligands that hinder the *in vivo* PET imaging of the neuronal CB_2_R are the low brain uptake and the low metabolic stability.^28,29,35,37,42−45^ No CB_2_R PET tracer is currently available for routine clinical usage. The common structure motifs found in most of the published CB_2_R ligands are shown in Figure 2.^27^

**Figure 3:**
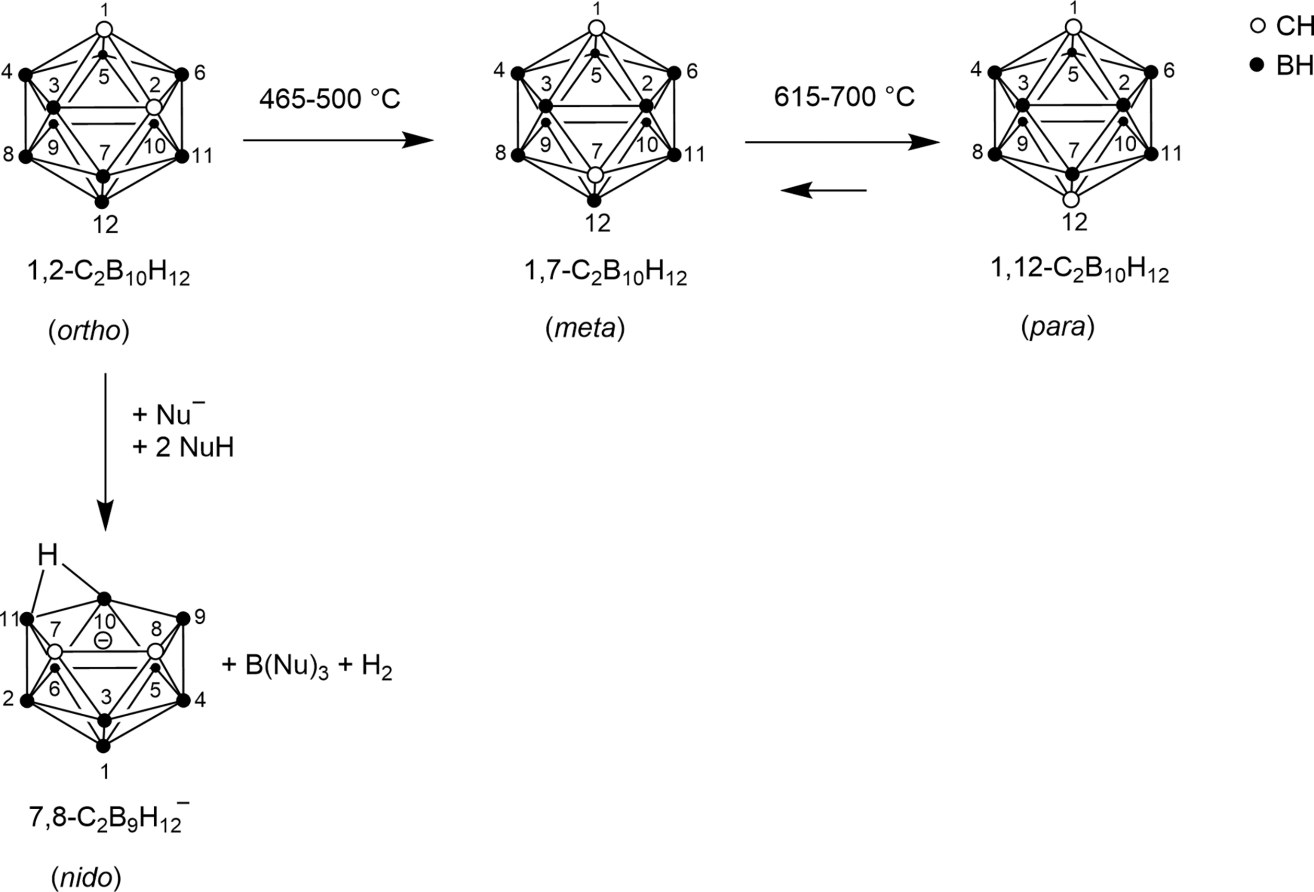
Transformation of the carborane isomers into each other and deboronation of *o*-carborane leading to *nido*-carborate(−1).^51,65,66^

To overcome the fast metabolism and to hone promising candidates, carboranyl moieties can be introduced as substituents to modify organic CB_2_R ligands.^46−50^ The icosahedral dicarba-*closo*-dodecaboranes(12) are cluster compounds consisting of two carbon, 10 boron, and 12 hydrogen atoms and can be divided into *ortho* (*o*)-, *meta* (*m*)-, and *para* (*p*)-carborane (Figure 3).^51^ The three isomers can be transformed into each other (Figure 3). They differ not only in size and volume [core volumes of 11.79 Å^3^ (*ortho*), 11.72 Å^3^ (*meta*), and 11.71 Å^3^ (*para*); van der Waals volumes (*V*_vdW_) based on crystal structures of 148 Å^3^ (*ortho*), 143 Å^3^ (*meta*), and 141 Å^3^ (*para*)]^51^ but also in their reactivity. In particular, the reactivity toward Lewis bases is highest for *o*-carborane and lowest for *p*-carborane. The *o*-carborane is most prone to undergo a deboronation reaction that leads to the *nido* cluster (Figure 3).^51,52^

Due to the delocalization of σ-bonding electrons of the cluster, carboranes are often termed three-dimensional σ-aromatic compounds and compared to benzene (π aromaticity);^52^ however, the average *V*_vdW_ of carboranes (144 Å^3^) is comparable to that of adamantane (136 Å^3^).^51^ Considering the chemical difference between the acidic CH units and the hydridic BH units, orthogonal regioselective substitution reactions at both moieties are possible^51^ and offer a very powerful tool for modifying and adjusting the properties.^46^ Carborane derivatives are applied in catalysis, in polymers, but are also of particular interest as pharmacophores in medicinal chemistry.^46,50−61^ Here, the hydrophobicity can facilitate the passage of cellular membranes and the BBB and is therefore especially important for monitoring effects in the brain.^46^ In addition, due to the inorganic nature of the carborane scaffold, a greater metabolic stability can be expected.^46−50^ Furthermore, the carborane cluster can interact in a noncovalent way with the chosen target, e.g., by forming dihydrogen bonds.^46^

**Figure 4:**
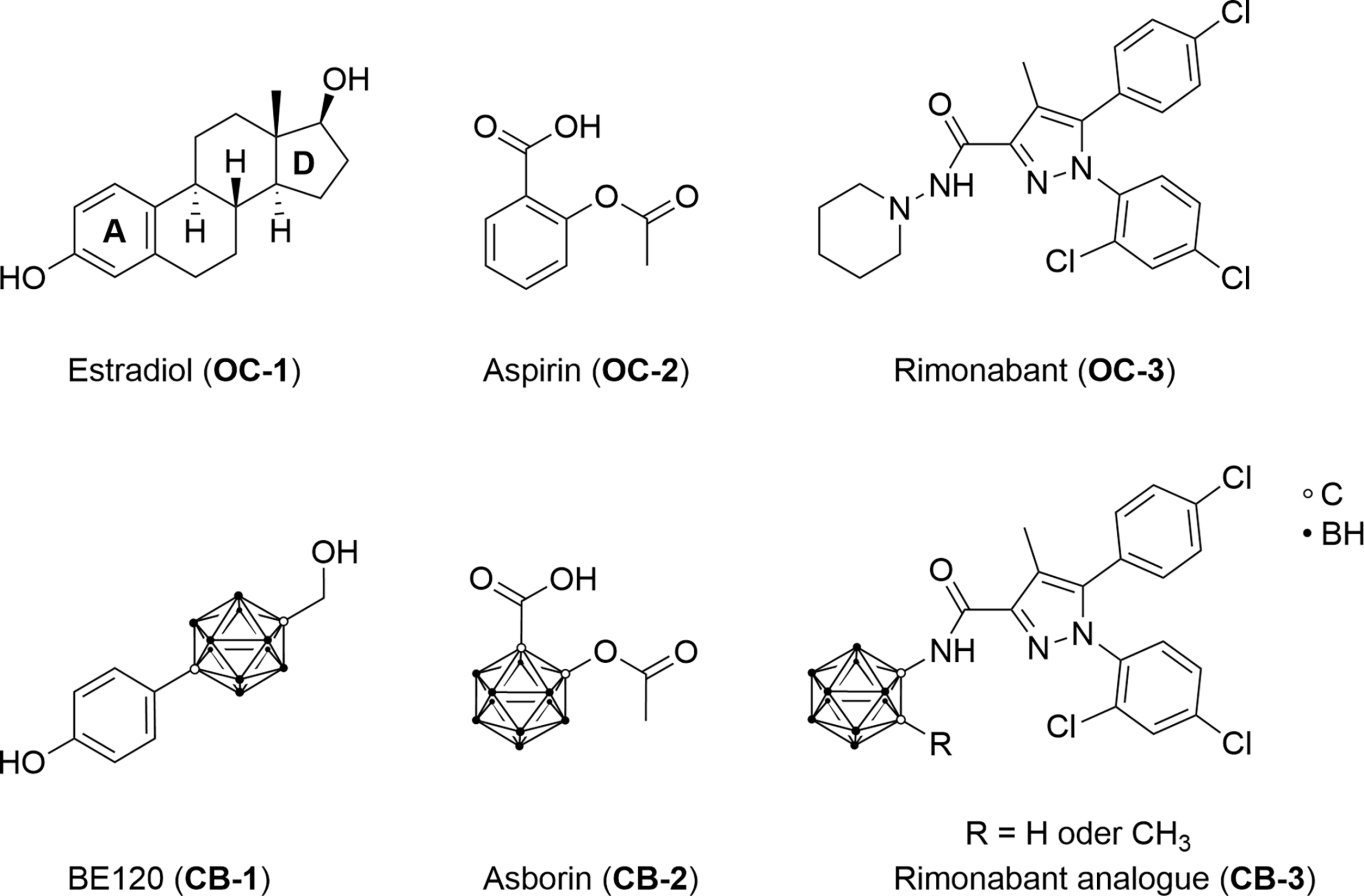
Carborane-based drug mimetics. Organic synthetic drugs **OC-1**–**OC-3** and corresponding carborane analogues **CB-1**–**CB-3**.^62−64^

In the past several decades, carborane-based drug mimetics have attracted much attention.^46^ Endo et al. synthesized a carborane-based estradiol analogue^62^ [BE120 (**CB-1**) (Figure 4)]. Scholz et al. reported asborin^63^ [**CB-2** (Figure 4)], the carborane analogue of aspirin (acetylsalicylic acid). Vázquez et al. published a rimonabant-derived CB_1_R antagonist^64^ [**CB-3** (Figure 4)] bearing a carborane moiety. So far, no CB_2_R ligand bearing a carboranyl moiety has been published, neither as a drug nor as a radiolabeled PET tracer.

**Figure 5:**
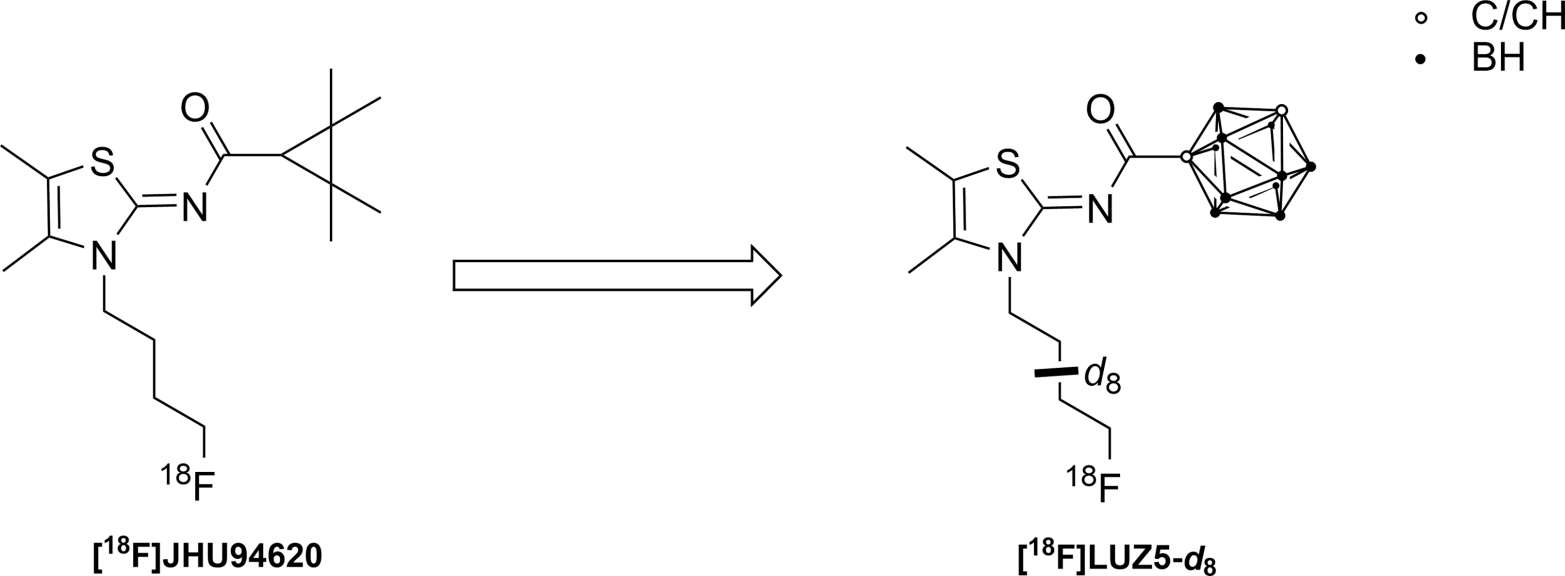
**[**^**18**^**F]JHU94620**(28,33) and its carborane derivative **[**^**18**^**F]LUZ5-*****d***_**8**_.

We here report the synthesis and investigation of the first carborane-based CB_2_R ligands, which were obtained by replacing either the tetramethylcyclopropyl unit in **JHU94620**(28) or the adamantyl residue in **LU13**(41) with *o*-, *m*-, or *p*-carborane. All new *closo*-carborane-containing CB_2_R ligands described herein were evaluated by *in vitro* metabolism studies using liver microsomes. To gain the first insights into the *in vivo* behavior of such carborane compounds, we designed a radiosynthesis for our most CB_2_R potent derivative **LUZ5** [compound **16**]. While a great number of aliphatic radiofluorinated PET candidates suffer from fast *in vivo* metabolism yielding free [^18^F]fluoride,^67,68^ the use of deuterated alkyls was shown to considerably improve the *in vivo* stability.^69^ Thus, a precursor bearing a fully deuterated *N*-butyl chain was synthesized and radiofluorinated in the presence of K[^18^F]F-K_2.2.2_ to give **[**^**18**^**F]LUZ5-*d***_**8**_ (Figure 5). The *in vivo* behavior of **[**^**18**^**F]LUZ5-*d***_**8**_ was evaluated in metabolic and PET studies *in vivo* in healthy female CD-1 mice and healthy male Wistar rats, which are the first preliminary steps in the development of a theranostic agent.

## Results and Discussion

### Synthesis

**Scheme 1:**
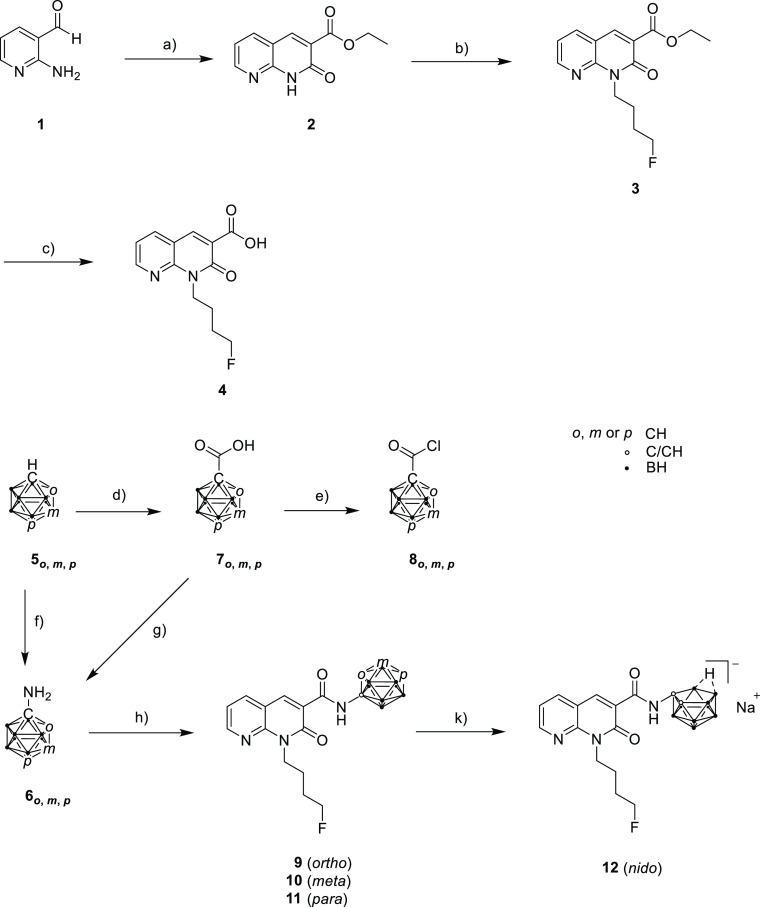
Synthesis of Target Compounds **9–12** Reagents and conditions: (a) diethylmalonate, piperidine, EtOH, reflux, 19 h, 74%; (b) 1-bromo-4-fluorobutane, Na_2_CO_3_, *N*,*N*-dimethylformamide (DMF), 90 °C, 16.5 h, 83%; (c) THF/MeOH/H_2_O [2:1:1 (v/v/v)], LiOH·H_2_O, 60 °C, 2 h, 62%; (d) (i) *n*-buthyllithium (*n*-BuLi), Et_2_O, CO_2_, 16–21 h, rt; (ii) HCl, 97% (**7**_*o*_), 11% (**7**_*m*_), 87% (**7**_*p*_); (e) toluene, PCl_5_, rt, 1 h, 56% (**8**_*o*_), 61% (**8**_*m*_), 66% (**8**_*p*_); (f) for *o*- and *m*-carborane, (i) *n*-BuLi, Et_2_O, benzyl azide, rt, 3 h; (ii) glacial acetic acid, 90 °C, 2 h, 34% (**6**_*o*_), 28% (**6**_*m*_); (g) for *p*-carborane, (iii) SOCl_2_, DMF, reflux, 5 h, (iv) (CH_3_)_3_SiN_3_, toluene, reflux, 1 h, (v) *tert*-BuOH, reflux, 18 h, (vi) TFA, dichloromethane (DCM), rt, 14 h, 8% (**6**_*p*_); (h) (i) **4**, SOCl_2_, 3 h, reflux, (ii) C_2_B_10_H_11_-NH_2_ (**6**_*o***,***m***,***p*_), toluene, compound **9**: 15 h at rt, microwave for 50 min, 150 °C, 48%; compound **10**: microwave for 50 min, 150 °C, 16 h at rt, microwave for 50 min, 150 °C, 42%; compound **11**: microwave for 50 min, 150 °C, 16 h at rt, microwave for 50 min, 150 °C, 50%; (k) for o-carborane: NaF, EtOH/H_2_O [1:1 (v/v)], microwave for 5 min, 150 °C, 79%.

The synthesis of the starting material 4-fluorobutyl-substituted 1,8-naphthyridin-2-one-3-carboxylic acid **4** was performed in three steps starting from 2-aminopyridine-3-carbaldehyde (**1**) as described by Lucchesi et al. and Sircar et al.^20,70^ In brief, aldehyde **1** was reacted in a Knoevenagel condensation with diethylmalonate and piperidine as the base. A nucleophilic substitution at the *N*H group of ester **2** introduced the 4-fluorobutyl chain followed by a basic ester hydrolysis of **3** with LiOH·H_2_O to give **4** (Scheme 1).^71^

For the next step (Scheme 1, h), the corresponding carboranyl amines were required. 1-Amino-1,2-dicarba-*closo*-dodecaborane (**6**_*o*_) and 1-amino-1,7-dicarba-*closo*-dodecaborane (**6**_*m*_) were synthesized as published by Nie et al.^72^ starting from *o*- (**5**_*o*_) or *m*-carborane (**5**_*m*_) (Scheme 1). However, 1-amino-1,12-dicarba-*closo*-dodecaborane (**6**_*p*_) could not be obtained by the same procedure or by varying the reaction time and temperature. Carboranyl amine **6**_*p*_ could finally be obtained by following the synthetic approach of Tsuji et al.^73^ via a multistep synthesis starting from the monosubstituted *p*-carborane-1-carboxylic acid **7**_*p*_, which is first chlorinated *in situ* followed by nucleophilic attack of trimethylsilyl azide at the carboxylic acid chloride and a Curtius rearrangement. The resulting isocyanate is reacted with *tert*-butanol to form the corresponding carbamate. Finally, the *tert*-butyloxycarbonyl (Boc) group was removed with trifluoroacetic acid (TFA) at room temperature (rt) to give compound **6**_*p*_ in 8% overall yield. Compound **6**_*p*_ was used without further purifications for the next synthetic step.

**Figure 6:**
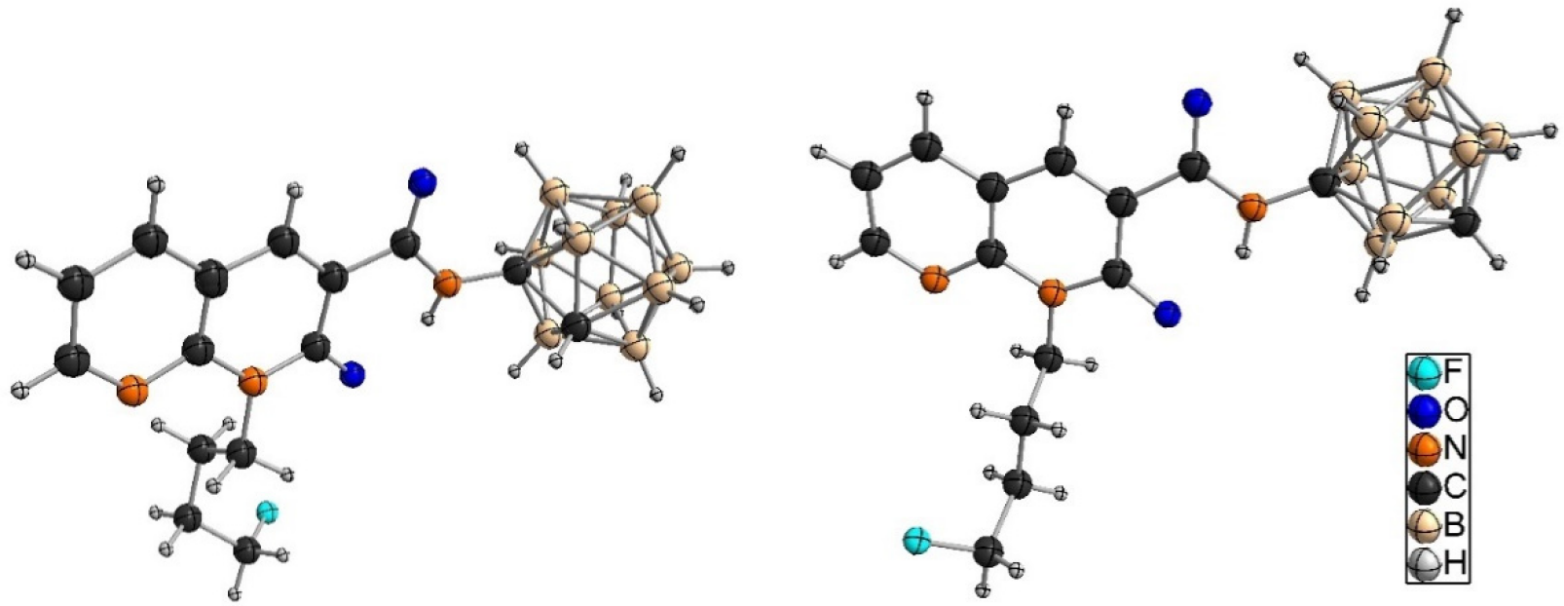
Molecular structures of **9** (left) and **10** (right). For **9**, a disorder of the carborane substituent at two positions (81:19) is observed (not shown).

The synthesis of naphthyridine-based target compounds **9**–**11** (Scheme 1) was adapted from Vázquez et al.^64^ Carboxylic acid **4** was first converted to the corresponding acid chloride that was further reacted with the respective carboranyl amines **6**_*o*_, **6**_*m*_, and **6**_*p*_ in a microwave reaction at 150 °C to give amides **9**–**11**, respectively, which were isolated in 42–50% yield by column chromatography. The three compounds were characterized by one-dimensional (^1^H, ^11^B{^1^H}, and ^13^C{^1^H}) and two-dimensional (COSY, HSQC, and HMBC) NMR spectroscopy and ESI-HRMS. Single crystals of *o*-carborane derivative **9** (Figure 6, left) and *m*-carborane analogue **10** (Figure 6, right) suitable for X-ray structure determination could be obtained from CDCl_3_ (**9**) or *n*-hexane/EtOAc/EtOH (**10** and **11**) (additional information is available in Table S1; the molecular structure of **11** is shown in Figure S82).

**9** was deboronated with NaF in EtOH/H_2_O [1:1 (v/v)] in a microwave reaction,^74,75^ yielding racemic *nido*-carborate(−1) derivative **12** with sodium as the counterion in 79% yield.

**Scheme 2:**
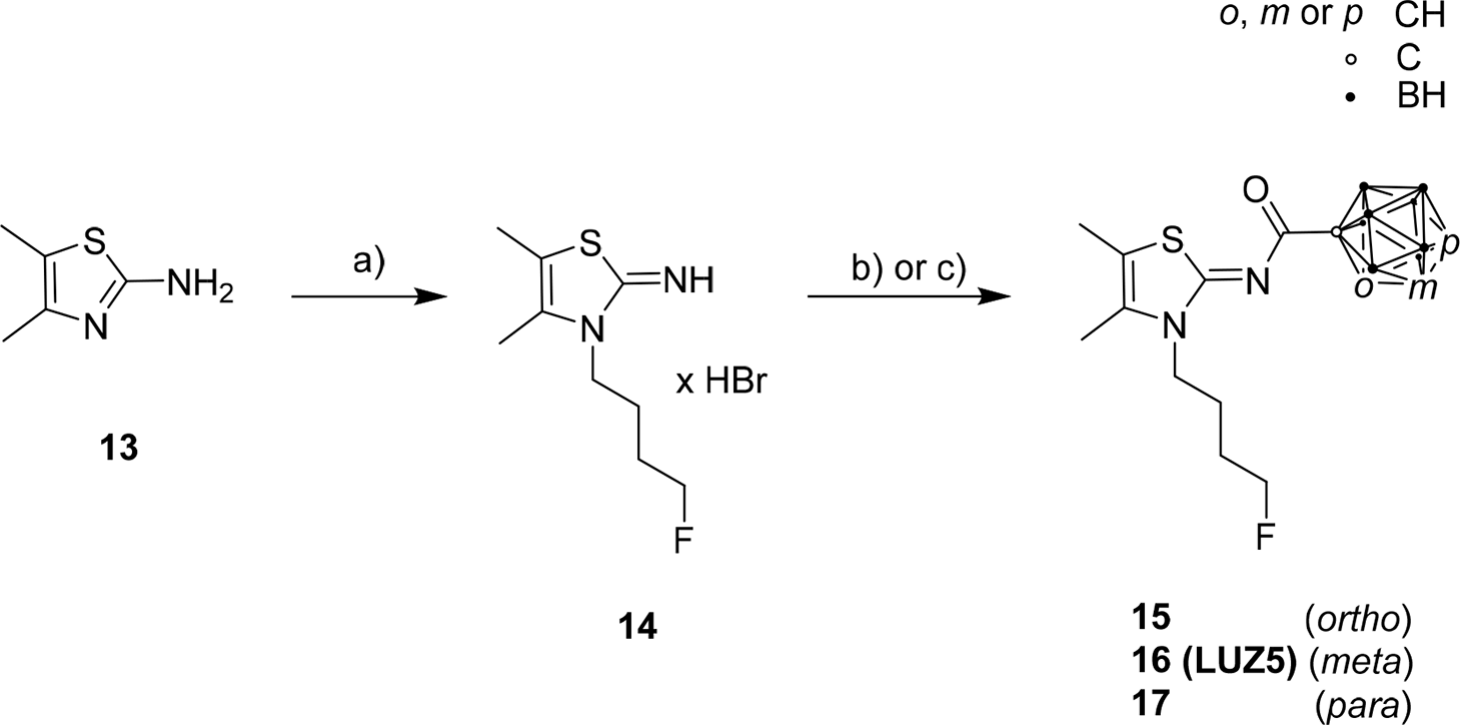
Synthesis of Target Compounds **15**, **16** (**LUZ5**), and **17** Reagents and conditions: (a) 1-bromo-4-fluorobutane, 80–90 °C, 21 h, 25%; (b) for *o*-C_2_B_10_H_11_-COCl (**8**_*o*_), triethylamine (NEt_3_), DCM, 48 h, rt, 24% (compound **15**); (c) for *o*-, *m*-, and *p*-C_2_B_10_H_11_-COCl (**8**_*o***,***m***,***p*_), NEt_3_, CH_3_CN, 20 h, 80 °C, 31% (compound **15**), 45% [compound **16** (**LUZ5**)], 36% (compound **17**).

The synthesis of compound **14** was performed starting from 2-amino-4,5-dimethylthiazole (**13**) and 1-bromo-4-fluorobutane (Scheme 2) as described in the literature.^28^ Target compounds **15**, **16** (**LUZ5**), and **17** (Scheme 2) were obtained by reaction of **14** with carboranyl acid chlorides **8**_*o*_, **8**_*m*_, and **8**_*p*_, respectively, which were obtained following literature procedures (Scheme 1).^76−78^

**Figure 7:**
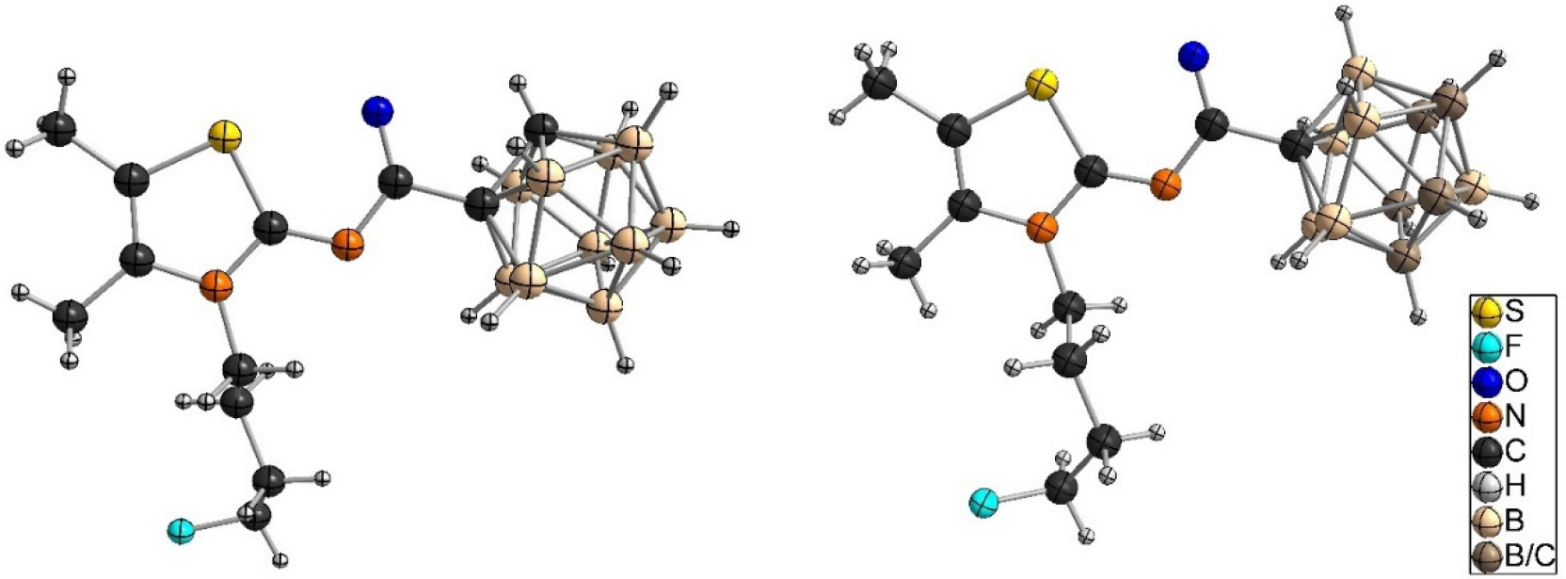
Molecular structures of **15** (left) and **LUZ5** (right). For **LUZ5**, a disorder of the fluorobutyl substituent in two positions is observed (not shown). The carbon atom of the carborane cluster bearing the substituent could be identified, while the second carbon atom of the carborane unit was treated as homogeneously 5-fold disordered over the entire CB_4_ ring.

Compound **15** was synthesized in two ways, either in DCM at rt in 48 h (procedure 1), adapted from Moldovan et al.^28^ (Scheme 2, b), or in CH_3_CN at 80 °C in 20 h (procedure 2), adapted from Richter et al.^79^ (Scheme 2, c). The reaction in acetonitrile at increased temperature was also used for the synthesis of **LUZ5** (45% yield) and **17** (36% yield). The molecular structures of **15** (Figure 7, left, crystallized from CDCl_3_), **LUZ5** (Figure 7, right, crystallized from CDCl_3_), and **17** (Figure S83, crystallized from *n*-hexane/DCM) were obtained by X-ray crystallography.

One of the (major) side products in the synthesis of **LUZ5** could be identified as the *N*3-unsubstitued thiazole-carborane acid amide (**SP1)** (Figures S39, S40, and S58) as shown by ^1^H and ^11^B{^1^H} NMR spectroscopy.

### Stability Tests

A high chemical and metabolic stability of the compounds is required for biological evaluation and application *in vivo*. The method of choice for evaluating the chemical stability was ^1^H and ^11^B{^1^H} NMR spectroscopy, with the latter being particularly suitable for recognizing deboronation. Since the *in vitro* binding assays are performed in aqueous media, the stability tests should be performed under similar conditions. However, due to the high hydrophobicity of the carborane moieties, target compounds **9**–**11** and **15**–**17** were not soluble in aqueous solution without the addition of dimethyl sulfoxide (DMSO). Since a maximum of 1% DMSO can be used in the *in vitro* binding assay, stability measurements were performed in aqueous DMSO-*d*_6_ to see especially if deboronation occurs in the presence of water. Approximately 10 mg of each compound (**9**–**11** and **15**–**17**) was dissolved in the deuterated NMR solvent and monitored via NMR spectroscopy in time intervals of a few minutes (after the addition of the solvent to the respective solid compound) to at least 23 days for the *o*- (**9** and **15**) and *m*-carborane derivatives (**10** and **LUZ5**) and beyond one year for the *p*-carborane analogues (**11** and **17**) (Figures S68–S79).

A general stability trend was observed with *o*-carborane derivatives having the lowest and *p*-carborane derivatives having the highest stability. This observation was in agreement with our expectations, because *o*-carboranes have the highest chemical reactivity of the three isomers and are most prone to undergo deboronation reactions, especially in aqueous solutions or in the presence of strong bases.^51^

Thiazole-based compounds **15** and **LUZ5** proved to be more stable than naphthyridine isomers **9** and **10**, respectively. For compound **9**, additional signals were visible already after 2 h in the ^1^H or ^11^B{^1^H} NMR spectra, indicating that a deboronation to *nido*-cluster derivative **12** had started. Thus, the stability of **9** is too low considering the time necessary to perform the binding affinity assay, which is 90 min. The other target compounds (**10**, **11**, **15**, **LUZ5**, and **17**) showed a stability beyond the binding affinity assay time frame with the following determined stability order: **9** < **15** < **10** < **LUZ5** < **11** = **17**. *p*-Carborane derivatives **11** and **17** were stable for more than one year.

For compound **LUZ5**, additional stability tests were performed with an increased concentration of H_2_O or D_2_O in DMSO-*d*_6_ [1:5 (v/v), in contrast to aqueous DMSO-*d*_6_ for previous measurements]. H_2_O or D_2_O was added to the clear solution, which immediately turned turbid indicating the formation of aggregates.^80^ However, investigation with ^11^B{^1^H} NMR spectroscopy was still possible and indicated that compound **LUZ5** was stable in the solvent mixture with increased amounts of H_2_O for at least 19 days and in the mixture with increased amounts of D_2_O for at least 8 days (Figures S80 and S81).

### *In Vitro* Binding Assay

Target compounds **9**–**11**, **15**, **LUZ5**, and **17** were evaluated in a competitive binding affinity assay using hCB_2_R Chinese hamster ovary (CHO) cells and the agonistic high-affinity CB_2_R radioligand **[**^**3**^**H]WIN55212-2** (Figure S96).

For all target compounds, *K*_i_ values in the nanomolar range could be determined (Table 1 and Figures S84–S87). Naphthyridine-based compounds **9**–**11** showed an increasing affinity of the *o*- to *p*-carborane derivatives toward the CB_2_R. Compound **11** (*K*_i_ = 3.9 nM) showed an affinity (∼10-fold) considerably higher than those of the other two compounds. Despite the low nanomolar range, the affinity of compound **11** is lower than that of reference compound **LU13** (Figure 1). Due to the low stability of **9**, the binding affinity determination might be influenced by the presence of small amounts of the *nido* derivative **12** that is currently under evaluation.

**Table 1:**
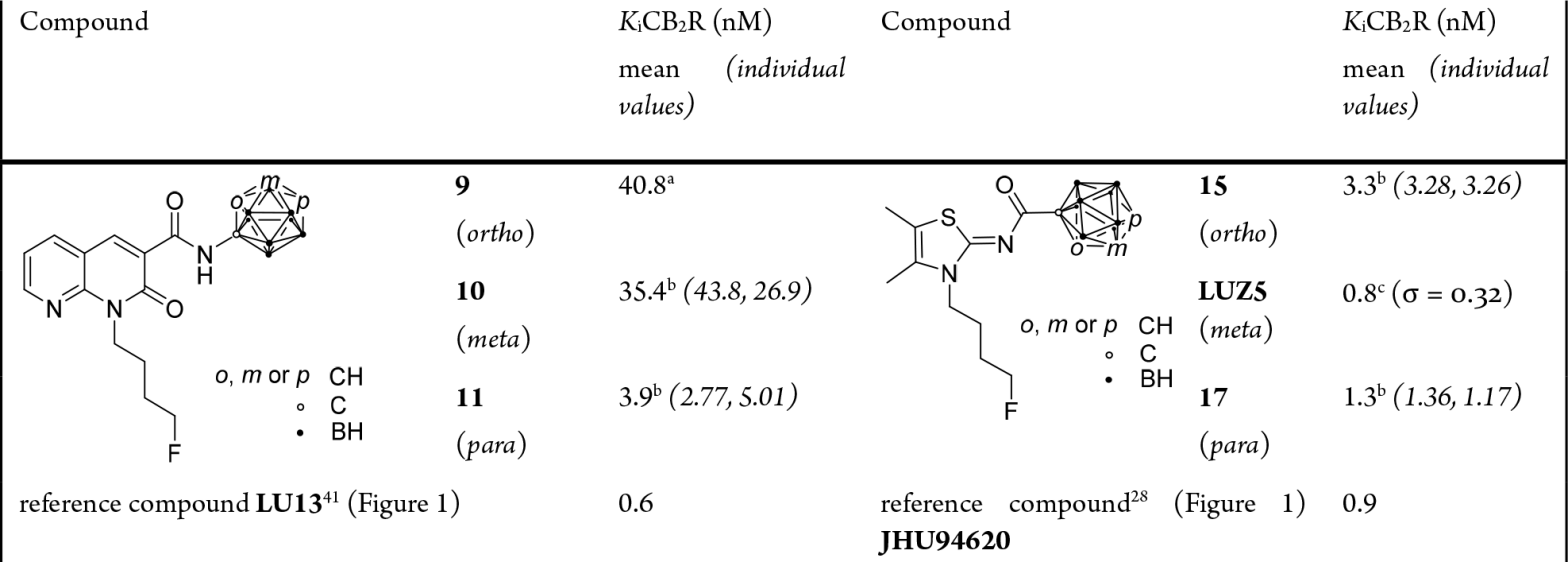
Binding Affinities of Target Compounds **9–11**, **15**, **LUZ5**, **17**, and Reference Compounds to the CB_2_R

a One value.

b Mean of two values.

c Mean of three values; σ is the standard deviation.

From the thiazole-based compounds, **17** and **LUZ5** showed remarkably high affinities of 1.3 and 0.8 nM, respectively, for the CB_2_R, surpassing the affinity of lead compound **JHU94620** (Figure 1). Moreover, compound **LUZ5** proved to be selective for the CB_2_R with a CB_1_R binding affinity of >10 μM (Figure S88). Compounds **9**–**11**, **15**, and **17** at concentrations of up to 1 μM did not displace the CB_1_R-specific radioligand **[^3^H]SR141716A** (Figure S96), and thus, we conclude that these compounds bind with only negligible affinity for the CB_1_R (Figure S89).

The finding that the thiazole-based compounds display a binding affinity higher than those of the respective naphthyridine-based ones is in agreement with the binding energies of the best docked positions (Figure S90). Therefore, for **9** with the lowest binding affinity, the binding energy is the highest (−4.53 kcal/mol) compared to those of **15** (−10.87 kcal/mol) and **LUZ5** (−9.68 kcal/mol). Details about the calculation of binding energies can be found in the Supporting Information [Chapter 7: Docking Data of Compounds **9**, **15** (Procedure 2), **LUZ5**].

### *In Vitro* Metabolism Studies

*In vitro* metabolism studies were performed for target compounds **9**–**11**, **15**, **LUZ5**, and **17**. Each of the compounds was incubated with mouse liver microsomes (MLMs) in phosphate-buffered saline (PBS) in the presence of nicotinamide adenine dinucleotide phosphate (NADPH) for 30 and 60 min at 37 °C, according to a literature protocol.^81,82^ After the addition of cold acetonitrile, the obtained supernatants were analyzed by HPLC-UV-MS (exemplified chromatogram and spectrum in Figures S91 and S92). The correct performance of the experimental setup was confirmed by complete conversion of testosterone under similar conditions. In addition, negative control samples without NADPH or without NADPH and MLM were prepared and analyzed for each target compound.

For each of the compounds investigated, no significant formation of metabolites was detected under the used conditions. This leads to the assumption that cytochrome P450-catalyzed functionalization reactions (phase I), such as oxidation, are not involved in the metabolism of this class of compounds in MLMs. However, the use of human liver microsomes (HLMs) instead of MLMs might provide a different picture, as species differences are described well in the literature^83,84^ and were already reported for CB_2_R ligands by Aly et al.^85^ However, as discussed in the section *In Vivo* Metabolism, radiometabolites were detected in plasma from rodents after administration of **[**^**18**^**F]LUZ5-*d***_**8**_. These findings suggest further *in vitro* investigations with regard to conjugation reactions (phase II), to understand the complexity of the processes taking place *in vivo*.

### Radiochemistry

On the basis of the binding affinity and stability tests, **LUZ5** was selected as the most promising candidate for developing a CB_2_R radioligand for further biological evaluations.

**Scheme 3:**
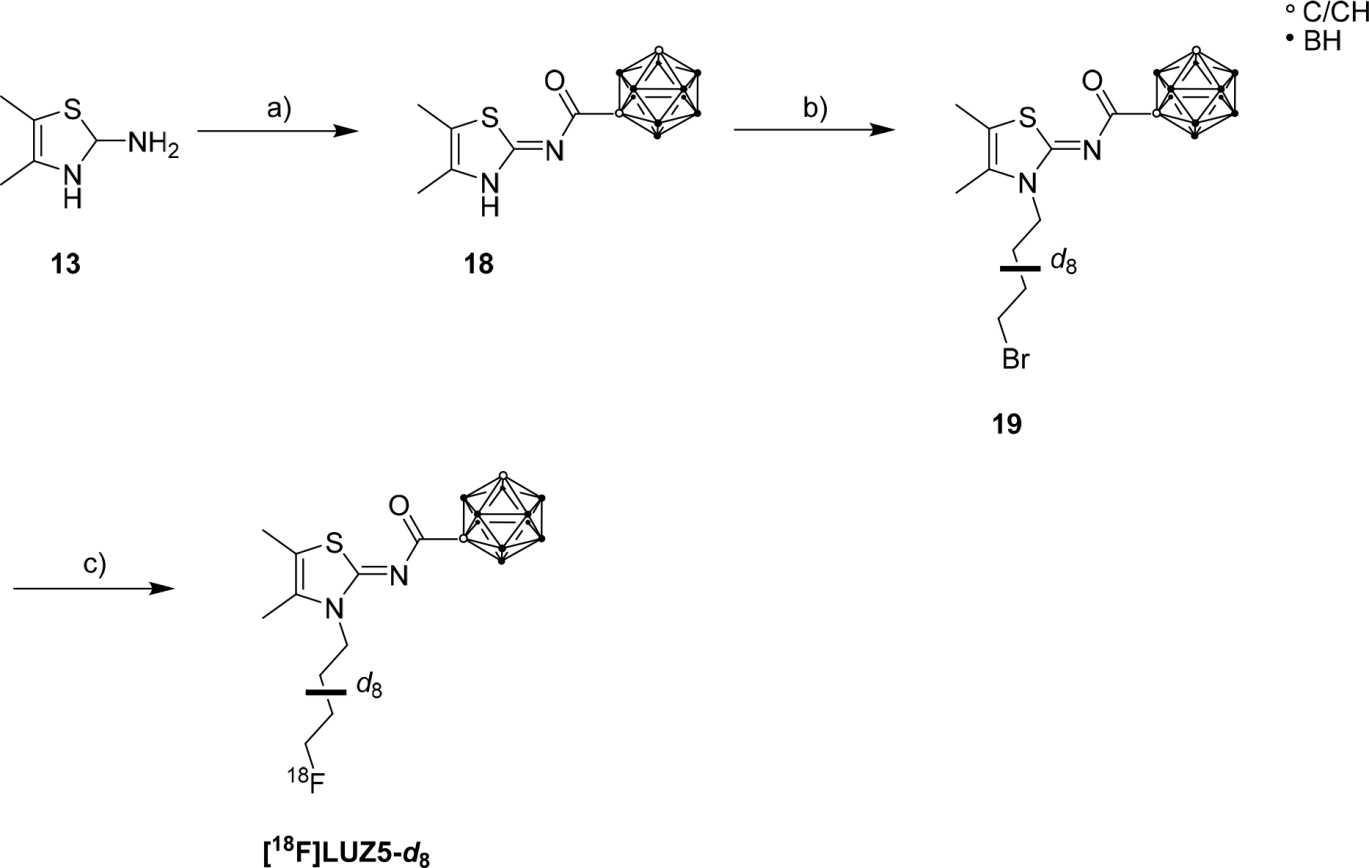
Synthesis of Brominated Precursor **19** and Radiofluorination with K[^**18**^F]F-K_2.2.2._ to Give **[**^**18**^**F]LUZ5-*d*_8_** Reagents and conditions: (a) C_2_B_10_H_11_-COCl (**8**_*m*_), NEt_3_, CH_3_CN, 16 h, 80 °C, 26%; (b) NaH, 1,4-dibromobutane-*d*_8_, DMF, 21 h, rt, 20%; (c) K**[**^18^F]F-K_2.2.2._, K_2_CO_3_, CH_3_CN, 90 °C, 10 min, 5–8% radiochemical yield.

The synthesis of precursor **19** was performed in two steps starting from 2-amino-4,5-dimethylthiazole (**13**) and *m*-carboranyl acid chloride (**8**_*m*_) (Scheme 3). The synthesis of intermediate **18** was analogous to the syntheses of **15**, **LUZ5**, and **17** (Scheme 2).^79^ For alkylation, the *N*3 position of **18** was deprotonated with NaH and reacted with 1,4-dibromobutane-*d*_8_ to give **19** (Scheme 3).^28^ The fully deuterated chain was introduced to confer metabolic stability.^40,41,69^ Brominated precursor **19** was purified by HPLC and then radiofluorinated via nucleophilic substitution under thermal conditions to give **[**^**18**^**F]LUZ5-*d***_**8**_.

**Figure 8:**
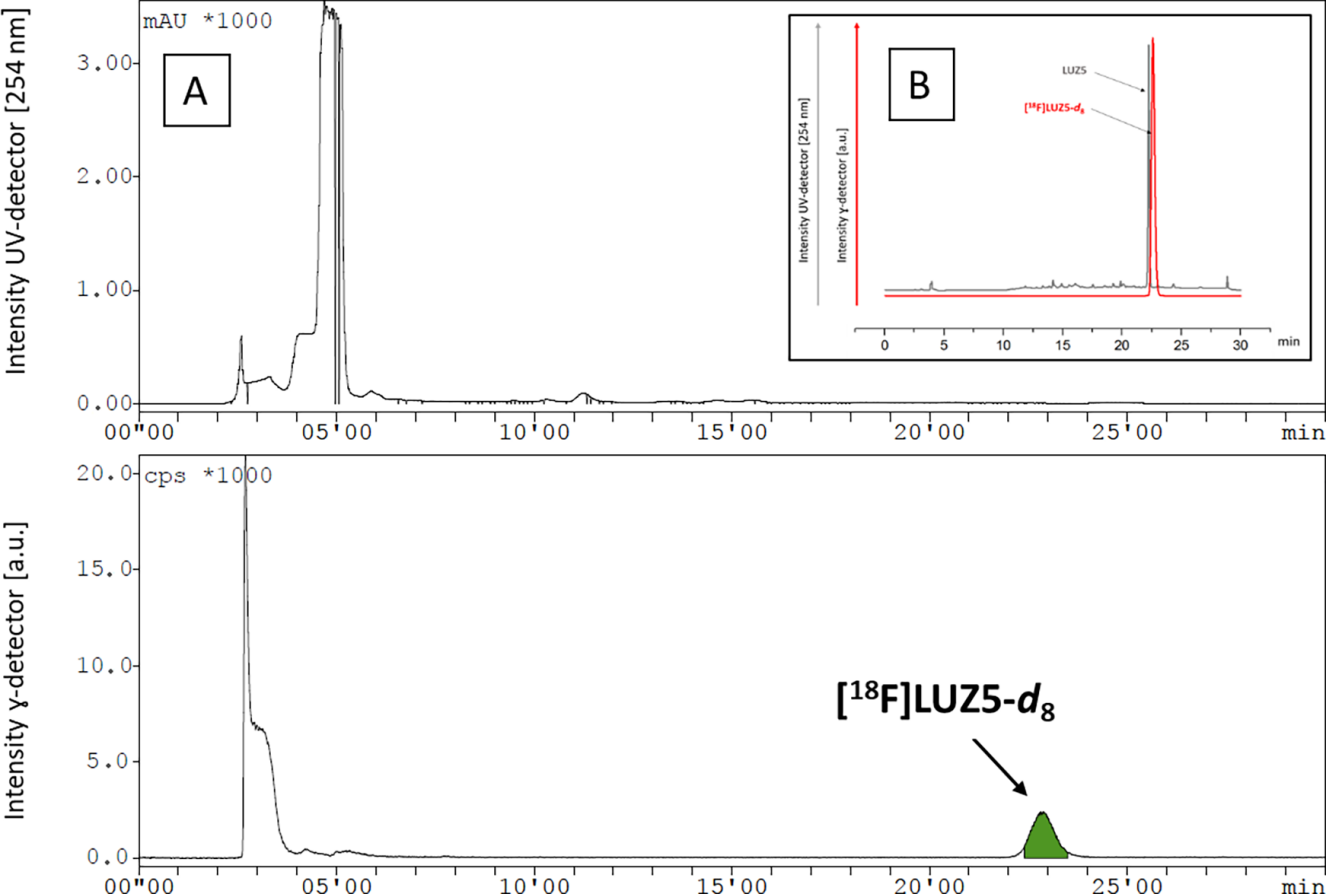
(A) Representative UV- and radio-HPLC chromatograms of **[**^**18**^**F]LUZ5-*d***_**8**_ [conditions, Reprosil-Pur 120 C18-AQ (5 μm, 250 mm × 10 mm), 73% CH_3_CN/20 mM NH_4_OAc_aq_, 4 mL/min] and (B) analytical radio-HPLC chromatogram of formulated **[**^**18**^**F]LUZ5-*d***_**8**_ [conditions: Reprosil-Pur 120 C18-AQ (5 μm, 250 mm × 4.6 mm), 70% CH_3_CN/20 mM NH_4_OAc_aq_, 1 mL/min] and UV-HPLC chromatogram of reference compound **LUZ5**.

The reaction conditions for the radiosynthesis of **[**^**18**^**F]LUZ5-*d***_**8**_ were investigated by using various amounts of 2.2.2-cryptand (K_2.2.2_) (13–40 μmol), K_2_CO_3_ (7.5–15 μmol), and precursor **19** (2.3−4.5 μmol) in CH_3_CN as the solvent under thermal conditions. Under each of the tested reaction conditions, **[**^**18**^**F]LUZ5-*d***_**8**_ was detected in the range between 25% and 50% (radio-HPLC, not isolated). On the basis of these preliminary results, an automated radiosynthesis for **[**^**18**^**F]LUZ5-*d***_**8**_ was developed on an Elysia-Raytest radiosynthesizer. The product was purified by HPLC (Figure 8A) followed by trapping the radiotracer **[**^**18**^**F]LUZ5-*d***_**8**_ on a reverse phase (RP) cartridge and elution with EtOH. For biological experiments, the solvent was evaporated under a stream of nitrogen at 70 °C. The final product was formulated in sterile isotonic saline up to a final concentration of <10% EtOH. The overall synthesis time was ∼85 min. **[**^**18**^**F]LUZ5-*d***_**8**_ was obtained in 5–8% radiochemical yield (decay-corrected from the end of bombardment), high radiochemical purity [>99% (Figure 8B)], and high molar activity in the range of 170–190 GBq/μmol at the end of the synthesis (*n* = 5). The identity of the final product **[**^**18**^**F]LUZ5-*d***_**8**_ was confirmed with analytical HPLC by co-injection with the corresponding reference compound [co-injection (Figure 8B); UV profile of the isolated radiotracer (Figure S93)]. No decomposition of the formulated product was observed within 24 h at room temperature. A logD_7.4_ of 3.0 was experimentally determined for **[**^**18**^**F]LUZ5-*d***_**8**_ by the shake-flask method (*n* = 3), which is well within the range of 1–3.5 recommended for brain-targeting compounds.^22,86^

### *In Vivo* Metabolism

*In vivo* metabolism studies of **[**^**18**^**F]LUZ5-*d***_**8**_ were carried out in healthy female CD-1 mice and healthy male Wistar rats. Samples of blood plasma, brain homogenates, spleen homogenates, and urine were obtained 30 min post-injection (p.i.), and organic solvent denaturation was performed to remove proteins. The extraction was carried out in mice samples by using either MeOH/H_2_O [9:1 (v/v)] or CH_3_CN/H_2_O [9:1 (v/v)], whereby an extraction efficiency of >94% was achieved using the former (*n* = 3). As a result, these conditions were also used to investigate the metabolic stability of **[**^**18**^**F]LUZ5-*d***_**8**_ in rats (*n* = 2).

**Figure 9:**
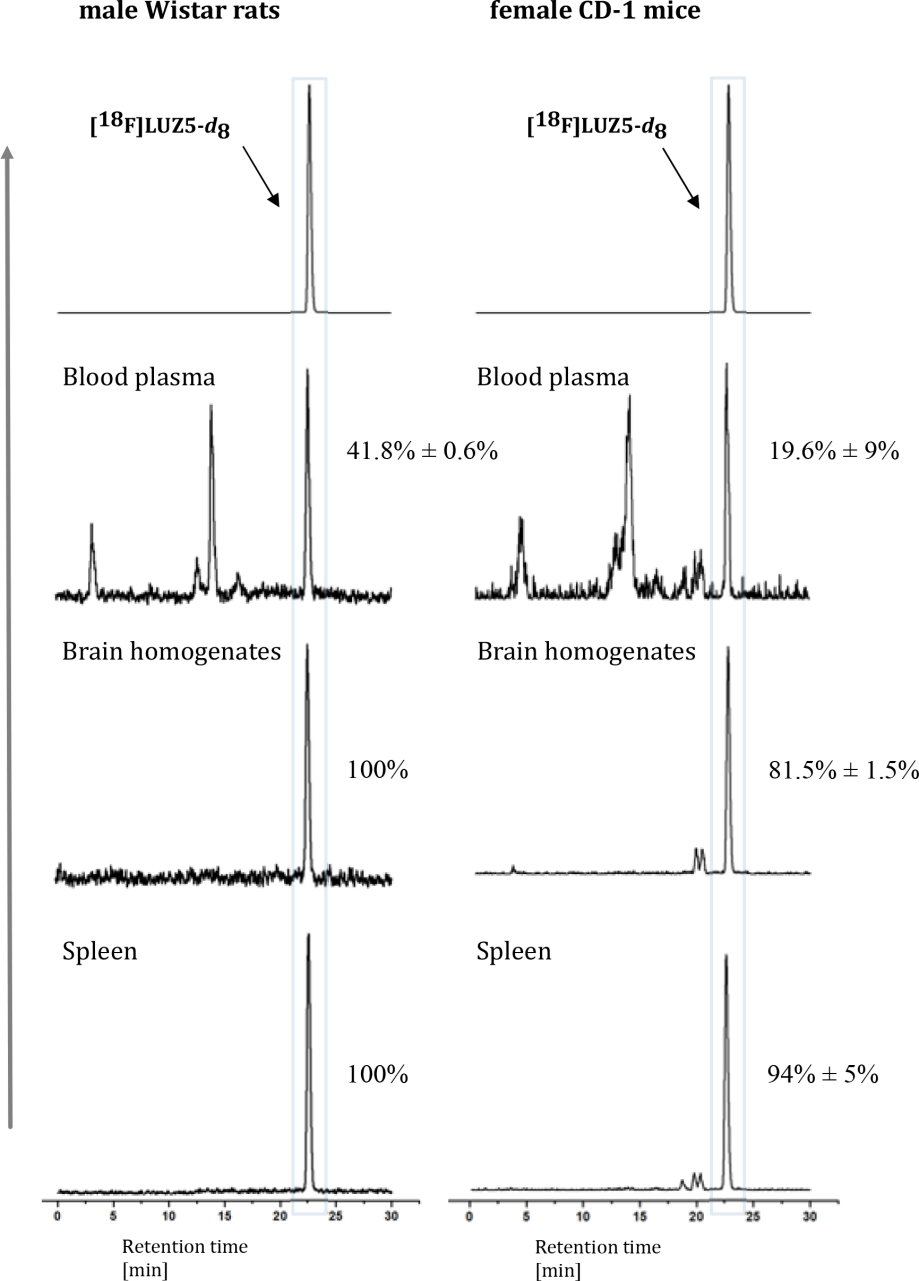
*In vivo* metabolism of **[**^**18**^**F]LUZ5-*****d***_**8**_. Representative radio-HPLC chromatograms of body fluids and organ samples of female CD-1 mice and male Wistar rats obtained at 30 min p.i. HPLC chromatograms of **[**^**18**^**F]LUZ5-*d***_**8**_, followed by samples of blood plasma, brain, and spleen homogenates of rats and mice [extraction with MeOH/H_2_O (9:1), extraction yield of ≥94%]. HPLC conditions: Reprosil-Pur 120 C18-AQ (5 μm, 250 mm × 4.6 mm); gradient mode (10-90-10 CH_3_CN/20 mM NH_4_OAc_aq_, 1 mL/min). Numbers next to the main signal indicate the percentage of the intact tracer.

In all investigated rat samples, larger amounts of an intact radiotracer were found compared to mice (Figure 9). For both mammals, significant amounts of radiometabolites were determined in blood plasma, with mean values of 19.6 ± 9% intact radiotracer in mice and 41.8 ± 0.6% in rats. Compared to the organic analogues **[**^**18**^**F]JHU94620** and **[**^**18**^**F]FC0(3)24** (Figure 1), an average larger amount of intact **[**^**18**^**F]LUZ5-*d***_**8**_ could be found in plasma of mice (19.6 ± 9% compared to 7% for **[**^**18**^**F]JHU94620**)^28^ and rats [41.8 ± 0.6% compared to 25% for **[**^**18**^**F]FC0(3)24**],^24^ respectively. Also in the brain samples, a much larger amount of intact radioligand was found in both mouse (81.5 ± 1.5%) and rat samples (100%) when compared to that of **[**^**18**^**F]JHU94620** [36%, data not reported for **[**^**18**^**F]FC0(3)24**].^24,28^ In the spleen samples, 94 ± 5% of intact tracer for mice and 100% of intact tracer for rats were found (Figure 9). The high metabolic stability can be explained by the presence of the carborane moiety and the implementation of the deuterated substituent at the *N*1 position of the molecule. Both structural changes might be influencing the stability toward enzymes *in vivo* and therefore increase the metabolic stability. In urine, no intact radiotracer could be detected.

### Assessment of the Biodistribution of **[^18^F]LUZ5-*d*_8_** in Rodents

**Figure 10:**
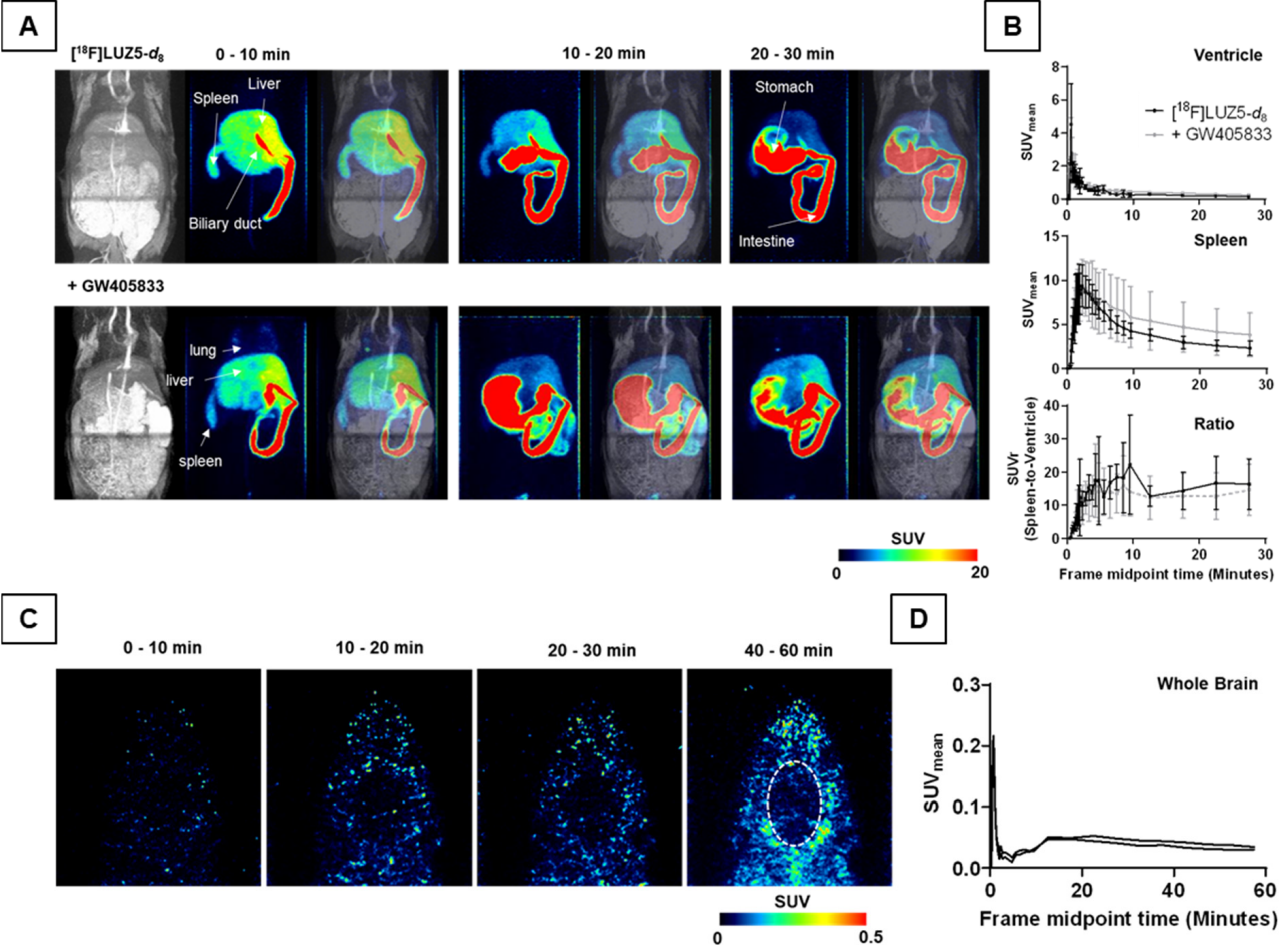
Dynamic PET studies of intravenously (i.v.) injected **[**^**18**^**F]LUZ5-*****d***_**8**_ in male Wistar rats. (A) Exemplary maximal intensity projections (MIPs) of magnetic resonance (MR) (T1-weighted), PET (averaged time frames as indicated), and merging of MR and PET of animals pretreated with or without 1.5 mg of **GW405833**/kg injected 10 min prior to the radiotracer (*n* = 3). (B) Time–activity curves (TACs) of the left ventricle and spleen in mean standardized uptake values (SUV_mean_ ± standard deviation), as well as the spleen uptake normalized to blood [SUVr (spleen-to-ventricle)]. (C) Horizontal sections of an exemplary PET recording of the head, with the dotted ellipse indicating the brain region. (D) Brain TACs of two different animals.

The biodistribution of **[**^**18**^**F]LUZ5-*d***_**8**_ over time was evaluated by dynamic PET imaging under control and blocking conditions using CB_2_R agonist **GW405833** (Figure S96) in healthy male Wistar rats (Figure 10 and Table 2) and in a healthy female CD-1 mouse (Figure S95).

**Table 2:** Noncompartmental Analysis of PET-Derived Rat Tissue TACs after i.v. Injection of **[**^**18**^**F]LUZ5-*d*_8_** with or without Pretreatment with **GW405833** (*n* = 3)

tissue	treatment	time to peak (min)	TAC peak value (SUV)	AUC_0–30 min_ (SUV min)
ventricle	vehicle	0.6 ± 0	4.5 ± 2.4	10 ± 2
	block	0.6 ± 0	3.7 ± 1.3	14 ± 1
	*p* value	1.000	0.6180	0.0516
spleen	vehicle	2.1 ± 0.3	9.5 ± 2.6	112 ± 21
	block	2.4 ± 0.8	9.5 ± 2.9	150 ± 80
	*p* value	0.6164	0.9958	0.4761
liver	vehicle	3.4 ± 0.3	13 ± 0.5	193 ± 16
	block	4.7 ± 0.7	14 ± 0.5	245 ± 46
	*p* value	0.0495	0.0962	0.1369
kidney	vehicle	1.3 ± 0.8	1.4 ± 0.1	18 ± 2
	block	10 ± 15	1.8 ± 0.3	30 ± 8
	*p* value	0.4325	0.1256	0.055
jejunum	vehicle	12 ± 5	42 ± 7	702 ± 82
	block	23 ± 9	15 ± 8	210 ± 128
	*p* value	0.1374	0.0130	0.0050
lung	vehicle	0.7 ± 0.1	10 ± 6	35 ± 29
	block	0.6 ± 0	12 ± 9	39 ± 30
	*p* value	0.1835	0.7244	0.8793
stomach	vehicle	53 ± 9	16 ± 23	94 ± 149
	block	16 ± 13	0.9 ± 0.8	15 ± 14
	*p* value	0.0144	0.3689	0.4552
bone	vehicle	24 ± 25	0.2 ± 0	2.8 ± 1.3
	block	14 ± 13	0.8 ± 0.9	8.7 ± 5.4
	*p* value	0.5494	0.3598	0.1411
muscle	vehicle	13 ± 0	0.2 ± 0	3.9 ± 0.7
	block	16 ± 3	0.2 ± 0	5.1 ± 0.8
	*p* value	0.1835	0.1106	0.1354

In rats, the analysis of the time–activity curves (TACs) of the left ventricle revealed a comparable radioactivity concentration over time in the blood pool AUC_0–30 min_ (area under the curve between 0 and 30 min p.i.) with comparable time-to-peak (TTP) and TAC peak values (TPV) between the control and blocking group. The spleen uptake of **[**^**18**^**F]LUZ5-*d***_**8**_ with a TPV of 9.5 ± 2.6, 2.1 ± 0.3 min post-injection, was high and comparable to the uptake after blocking. This was also reflected by the normalized spleen SUV (standardized uptake value) to the blood pool activity with a mean SUVr (SUV ratio of *x* to reference) between 10 and 22 (control) and 10 and 16 (blocking) from 2 to 30 min p.i. of the radiotracer. Thus, a displaceable uptake of **[**^**18**^**F]LUZ5-*d***_**8**_ into the spleen under these conditions could not be shown *in vivo*. The radiotracer was mainly hepatobiliary excreted as shown by the high AUC_0–30 min_ values of the liver, jejunum, and stomach compared to kidney and the rather low accumulated bladder radioactivity of 0.3 ± 0.1% of the injected activity after 60 min p.i. in the control group (Figure S94). The hepatobiliary excretion was delayed by the blocking compound, which most likely caused the higher renal radioactivity accumulation as shown by the TTP and AUC_0–30 min_ values and the decreased radioactivity concentration in the small intestine. The dynamic PET recordings were limited by the field of view of the device, so altered bladder activity under blocking conditions could unfortunately not be measured, as determination of blood pool activity was prioritized. A negligible radioactivity uptake could be observed in lung, muscle, and bone. Furthermore, the brain uptake of **[**^**18**^**F]LUZ5-*d***_**8**_ in the initial distribution phase was rather low and followed by a fast washout in rats as shown in panels C and D of Figure 10.

Compared to rats, in an exemplary CD-1 mouse (Figure S95) the spleen uptake was lower, with a TPV of 1.2 after 7.5 min p.i. A high hepatobiliary excretion in mouse was found, as well, whereas the activity in bladder after 30 min was 1.9% of the injected radioactivity and therefore 10 times higher than that in rats, which could be in part explained by species-dependent radiometabolite formation. In mouse, the brain uptake as well as uptake in muscle and bone was low and comparable to that in rats.

Taken together, the uptake of **[**^**18**^**F]LUZ5-*****d***_**8**_ in the CB_2_R-rich tissue spleen was higher in rats than in mice but could not be blocked by the CB_2_R agonist **GW405833**. With the exception of the excretion pathway (mainly hepatobiliary), hints of an increased off-target uptake of the radioligand in tissues with a low CB_2_R density could not be found in both species. However, the brain uptake is rather low and a slow defluorination of **[**^**18**^**F]LUZ5-*d***_**8**_ can be assumed due to the low level of accumulation of radioactivity in the bone.

## Conclusions

In this study, a new class consisting of seven carborane-based CB_2_R ligands has been developed and evaluated. The lead structures of target compounds **9**–**11**, **15**, **16** (**LUZ5**), and **17**, based on 1,8-naphthyridinone or thiazole scaffolds, were modified by introducing *o*-, *m*-, and *p*-carborane moieties. The *nido*-compound **12** was generated via deboronation of **9**. Compounds **11**, **15**, and **17** showed good chemical stability in aqueous DMSO-*d*_6_ and low nanomolar affinities (*K*_i_ = 3.3, 3.9, and 1.3 nM, respectively). The binding affinity of the thiazole derivatives surpassed that of the naphthyridine derivatives. In this study, we identified **LUZ5** with subnanomolar affinity exceeding the reference compound **JHU94620**. Encouraged by these results, we developed an automated radiofluorination for **[**^**18**^**F]LUZ5-*d***_**8**_. The *in vivo* metabolic stability of **[**^**18**^**F]LUZ5-*d***_**8**_ bearing a deuterated butyl chain was investigated in rodents. While in mouse plasma only a small fraction of intact tracer was found 30 min p.i., the percentage of intact radioligand in mouse brain and spleen was high (81.5 ± 1.5% and 94 ± 5%, respectively) and superior to that of **[**^**18**^**F]JHU94620**. The metabolic stability of **[**^**18**^**F]LUZ5-*d***_**8**_ in rats exceeded that in mice. Dynamic PET scans in rats with and without blocking agent **GW405833** revealed a fast uptake in spleen and liver, a fast hepatic clearance, and nondisplaceable binding in spleen. The uptake of radiotracer in brain was low. The development of **[**^**18**^**F]LUZ5-*d***_**8**_ gave access to a new class of ligands for CB_2_R bearing a carborane moiety as a bioisostere for an adamantyl or tetramethylcyclopropyl group, which could alter and improve the properties of existing ligands and help to overcome the metabolic stability problems of the known CB_2_R ligands. For the use of such ligands as PET tracers targeting brain, further structural adjustments have to be considered. The foundation for further research toward diagnostic agents for theranostic application has been laid with this preliminary evaluation of an ^18^F-labeled radiotracer.

## Experimental Section

### General Information

All reactions involving carboranes were carried out under a nitrogen atmosphere using the Schlenk technique. Anhydrous DCM, Et_2_O, *n*-hexane, and toluene were dried with the MB SPS-800 solvent purification system (MBRAUN, M.Braun Inertgas-Systeme GmbH, Garching, Germany). Acetonitrile and DMF were dried over calcium hydride and distilled. Dry solvents were stored over molecular sieves (4 or 5 Å). Benzyl azide was prepared according to the literature.^87^ Dicarba-*closo*-dodecaborane-1-carboxylic acids (**7**_*o*_, **7**_*m*_, and **7**_*p*_) can be prepared according to the literature.^76,77^ Dicarba-*closo*-dodecaborane-1-carboxylic acid chlorides (**8**_*o*_, **8**_*m*_, and **8**_*p*_) can be synthesized as described by Kasar et al.^78^ 1,2-and 1,12-Dicarba-*closo*-dodecaborane-1-amine **6**_*o*_ and **6**_*p*_ can be prepared as described by Nie et al.^72^ and Tsuji et al.^73^ The synthesis of 1,7-dicarba-*closo*-dodecaborane-1-amine **6**_*m*_ was adapted from Nie et al.^72^ Compound **4** can be prepared according to a previously published protocol.^20,41,70,71^ Compound **14** can be synthesized as published by Moldovan et al.^28^ All other solvents and chemicals were commercially available and used without further purification. Microwave reactions were performed by using an Initiator+ microwave from Biotage (Uppsala, Sweden). Reaction progress and purification of products were monitored by thin-layer chromatography (TLC) using precoated silica gel 60 F_254_ Alumgram plates (Xtra SIL G) from Macherey-Nagel (Düren, Germany). Parts of TLC plates containing carboranes were stained with a 5–10% PdCl_2_ solution in methanol. Chromatography was performed in air, with silica gel (60 Å, 0.035–0.070 mm particle diameter) or in an automated fashion with an Isolera-4 and ELSD 1080 (Biotage) with commercially available solvents.

NMR spectra were recorded with Avance III HD 400 and Avance DRX 400 spectrometers from Bruker (Billerica, MA). Measurements were performed at 400.13 MHz (^1^H), 128.38 MHz (^11^B), and 100.63 MHz (^13^C). Chemical shifts (δ) are given in parts per million (ppm). ^1^H and ^13^C NMR spectra were referenced to internal deuterated solvent, and ^11^B{^1^H} NMR spectra to the Ξ scale.^88^ Deuterated solvents (CDCl_3_, DMSO-*d*_6_, and acetone-*d*_6_) were purchased from Eurisotop (Saint-Aubin, France) with a deuteration rate of 99.80%. High-resolution mass spectrometry (HRMS) was conducted in positive ion mode with an ESI-TOF microTOF instrument from Bruker Daltonik GmbH (Bremen, Germany). The simulation of mass spectra was carried out with the Web-based MS online tool of Scientific Instrument Services (SISweb, Palmer, MA). The analysis of NMR and MS data was done with MestReNova version 14.1.0.^89^ To determine melting point ranges, the model MPD350.BM2.5 melting point apparatus from Gallenkamp (Cambridge, U.K.) was used. The values are uncorrected. Samples were tested as triplicates, and the mean was calculated. X-ray analysis was performed with single crystals, obtained from CDCl_3_, EtOH/EtOAc/*n*-hexane, or *n*-hexane/DCM at rt by slow evaporation of the solvent or by heating and slowly cooling saturated solutions. The crystals were measured with a Gemini diffractometer (Rigaku Oxford Diffraction) with Mo Kα radiation (λ = 71.073 pm) or Cu-Kα radiation (λ = 154.184 pm) in ω-scan mode. Data reduction was performed with CrysAlis Pro.^90^ Empirical absorption correction was performed with SCALE3 ABSPACK.^91^ Structure solution and anisotropic refinement of all atoms except hydrogen atoms and some disordered parts of molecules were performed with SHELXT^92^ and SHELXL.^93^ The position of the hydrogen atoms was calculated by locating them on difference Fourier maps calculated at the final stage of the structure refinement excluding disordered fragments. For **LUZ5** and for disordered fragments, all hydrogen atoms were calculated on idealized positions. Further details on disordered moieties, fundamental structure parameters and CCDC deposition numbers are given in the Supporting Information (Table S1). The visualizations (figures) were generated with Diamond version 4.^94^ The purity of all of the compounds evaluated in biological tests was ≥95% as determined by HPLC [Jasco, Pfungstadt, Germany, MD-2010Plus, LG-2080-04S, DG-2080-54, AS-2055Plus, LC-NetII/ADC, λ = 280 nm, column ReproSil-Pur C18-AQ (250 mm × 4.6 mm, 5 μm, Dr. Maisch GmbH, Ammerbuch-Entringen, Germany), gradient of CH_3_CN/20 mM ammonium acetate, flow rate of 1 mL/min; method: 10% CH_3_CN/20 mM NH_4_OAc_aq_ from 0 to 5 min, 10% to 90% CH_3_CN/20 mM NH_4_OAc_aq_ from 5 to 20 min, 90% CH_3_CN/20 mM NH_4_OAc_aq_ from 20 to 35 min, 90% to 10% CH_3_CN/20 mM NH_4_OAc_aq_ from 35 to 36 min, and 10% CH_3_CN/20 mM NH_4_OAc_aq_ from 36 to 40 min]. Additionally, for target compound **LUZ5**, an isocratic HPLC method (66% CH_3_CN/20 mM NH_4_OAc_aq_, flow rate of 1 mL/min) was used.

### Chemical Synthesis

*1-(4-Fluorobutyl)-2-oxo-*N*-(dicarba-closo-dodecaboranyl)-1,2-dihydro-1,8-naphthyridine-3-carboxamide* (**9**–**11**).^64^ SOCl_2_ (4.01 equiv) was added to a solution of **4** (0.378 mmol, 1.00 equiv) in dry toluene (10 mL) and stirred for 3 h under reflux. The solvent was removed under reduced pressure after cooling to rt. The acid chloride in dry toluene (1–1.5 mL) was added to a microwave vial of the respective dicarba-*closo*-dodecaborane-1-amine (**6**, 0.377 mmol, 0.996–1.01 equiv), dissolved in dry toluene (1 mL). The reaction mixture was stirred for 15 h at rt and kept for 50 min in the microwave at 150 °C for the *ortho* isomer. For the *meta* and *para* isomers, the microwave reaction was carried out for 50 min at 150 °C, and then the mixture was stirred for 16 h at rt and again microwaved for 50 min at 150 °C. The solvent was removed under reduced pressure, and the crude product was purified by column chromatography. *1-(4-Fluorobutyl)-2-oxo-*N*-(1,2-dicarba-*closo*-dodecaboranyl)-1,2-dihydro-1,8-naphthyridine-3-carboxamide* (**9**) [*n*-hexane/EtOAc, 4:1 (v/v) → 1.5:1 (v/v)] was obtained as a white solid: 0.073 g (0.180 mmol, 48%); ^1^H NMR (400 MHz, CDCl_3_) δ 1.43–3.64 (br, 10H), 1.87 (m, 4H), 4.54 (dt, ^2^*J*_HF_ = 47.5 Hz, ^3^*J* = 5.7 Hz, 2H), 4.64 (t, ^3^*J* = 7.3 Hz, 2H), 5.13 (s, 1H, CH), 7.34 (dd, ^3^*J* = 7.8, 4.6 Hz, 1H), 8.11 (dd, ^3^*J* = 7.8, 1.9 Hz, 1H), 8.78 (dd, ^3^*J* = 4.6, 1.8 Hz, 1H), 8.81 (s, 1H), 11.67 (s, 1H); ^11^B{^1^H} NMR (128 MHz, CDCl_3_) δ −13.9 (s, 3B), −10.9 (s, 5B), −7.1 (s, 1B), −4.0 (s, 1B); ^13^C{^1^H} NMR (101 MHz, CDCl_3_) δ 24.1 (d, ^3^*J*_CF_ = 5.1 Hz), 28.1 (d, ^2^*J*_CF_ = 20.1 Hz), 42.0, 60.6, 78.9, 83.8 (d, ^1^*J*_CF_ = 165.3 Hz), 114.8, 119.9, 121.0, 139.2, 143.7, 150.0, 153.6, 161.9, 162.7; HRMS (ESI+) *m*/*z* for C_15_H_24_B_10_FN_3_NaO_2_ [M + Na]^+^ 428.2764, calcd 428.2754; melting range 177–178 °C.

*1-(4-Fluorobutyl)-2-oxo-*N*-(1,7-dicarba-closo-dodecaboranyl)-1,2-dihydro-1,8-naphthyridine-3-carboxamide* (**10**) [5:1 (v/v) *n*-hexane/EtOAc → 100% EtOAc] was obtained as a white solid: 0.065 g (0.160 mmol, 42%); ^1^H NMR (400 MHz, CDCl_3_) δ 1.40–4.16 (br, 10H), 1.84 (m, 4H), 2.96 (s, 1H), 4.52 (dt, ^2^*J*_HF_ = 47.6 Hz, ^3^*J* = 5.6 Hz, 2H), 4.61 (t, ^3^*J* = 7.2 Hz, 2H), 7.31 (dd, ^3^*J* = 7.8, 4.7 Hz, 1H), 8.07 (dd, ^3^*J* = 8.3, 1.8 Hz, 1H), 8.74 (dd, ^3^*J* = 4.7, 1.7 Hz, 1H), 8.83 (s, 1H), 10.94 (s, 1H); ^11^B{^1^H} NMR (128 MHz, CDCl_3_) δ −15.2 (s, 5B), −12.4 (s, 2B), −10.7 (s, 2B), −3.8 (s, 1B); ^13^C{^1^H} NMR (101 MHz, CDCl_3_) δ 24.1 (d, ^3^*J*_CF_ = 5.2 Hz), 28.1 (d, ^2^*J*_CF_ = 20.0 Hz), 41.8, 53.2, 79.8, 83.9 (d, ^1^*J*_CF_ = 164.9 Hz), 114.9, 119.6, 121.8, 139.0, 143.3, 150.0, 153.1, 161.2, 162.6; HRMS (ESI+) *m*/*z* for C_15_H_25_B_10_FN_3_O_2_ [M + H]^+^ 406.2940, calcd 406.2934; melting range 176–178 °C.

*1-(4-Fluorobutyl)-2-oxo-*N*-(1,7-dicarba-closo-dodecaboranyl)-1,2-dihydro-1,8-naphthyridine-3-carboxamide* (**11**) [9:1 (v/v) *n*-hexane/EtOAc → 100% EtOAc] was obtained as a white solid: 0.077 g (0.190 mmol, 50%); ^1^H NMR (400 MHz, CDCl_3_) δ 1.40–3.58 (br, 10H), 1.83 (m, 4H), 2.71 (s, 1H), 4.49 (dt, ^2^*J*_HF_ = 47.7 Hz, ^3^*J* = 6.0, 5.6 Hz, 2H), 4.59 (t, ^3^*J* = 7.1 Hz, 2H), 7.28 (dd, ^3^*J* = 8.5, 5.3 Hz, 1H), 8.04 (dd, ^3^*J* = 7.9, 1.9 Hz, 1H), 8.72 (dd, ^3^*J* = 4.8, 1.8 Hz, 1H), 8.76 (s, 1H), 10.63 (s, 1H); ^11^B{^1^H} NMR (128 MHz, CDCl_3_) δ −16.5 (s, 5B), −12.5 (s, 5B); ^13^C{^1^H} NMR (101 MHz, CDCl_3_) δ 24.0 (d, ^3^*J*_CF_ = 5.2 Hz), 28.0 (d, ^2^*J*_CF_ = 20.1 Hz), 41.7, 56.3, 83.8 (d, ^1^*J*_CF_ = 165.0 Hz), 88.0, 114.8, 119.5, 122.0, 138.8, 143.1, 149.8, 152.9, 160.3, 162.5; HRMS (ESI+) *m*/*z* for C_15_H_24_B_10_FN_3_NaO_2_ [M + Na]^+^ 428.2755, calcd 428.2754; melting range 209–213 °C.

*Sodium 1-(4-fluorobutyl)-2-oxo-*N*-(7,8-dicarba-nido-dodeca-hydroundecaborate(−1))-1,2-dihydro-1,8-naphthyridine-3-carboxamide* (**12**, racemate).^74,75^ A microwave vial was filled with **9** (65.1 mg, 0.160 mmol, 1.00 equiv), NaF (50.0 mg, 1.19 mmol, 7.43 equiv), and a mixture of degassed EtOH/H_2_O [4.0 mL, 1:1 (v/v)]. The white suspension was stirred for 5 min at 150 °C in the microwave. The solution was decanted, and the solvent was removed under reduced pressure: ^1^H NMR (400 MHz, acetone-*d*_6_) δ −2.03 (d, ^3^*J* = 66.9 Hz, 1H), −0.55–2.96 (br, 9H), 1.77 (m, 4H), 3.26 (s, 1H, CH), 4.42 (t, ^3^*J* = 5.8 Hz, 1H), 4.54 (m, 3H), 7.44 (dd, ^3^*J* = 7.8, 4.6 Hz, 1H), 8.40 (dd, ^3^*J* = 7.8, 1.8 Hz, 1H), 8.74 (dd, ^3^*J* = 4.7, 1.8 Hz, 1H), 8.91 (s, 1H), 10.12 (s, 1H); ^11^B{^1^H} NMR (128 MHz, acetone-*d*_6_) δ −37.9 (s, 1B), −33.5 (s, 2B), −24.2 (s, 1B), −19.6 (s, 1B), −18.1 (s, 1B), −14.3 (s, 1B), −10.5 (s, 2B); ^13^C{^1^H} NMR (101 MHz, acetone-*d*_6_) δ 24.0 (d, ^3^*J*_CF_ = 5.3 Hz), 28.1 (d, ^2^*J*_CF_ = 19.9 Hz), 41.3, 83.8 (d, ^1^*J*_CF_ = 163.2 Hz), 115.2, 119.7, 123.3, 139.4, 142.1, 149.9, 152.5, 162.5, 163.6.

Compound **12** was purified by HPLC using a Reprosil-Pur 120 C18-AQ (250 mm × 20 mm, 10 μm) column with 50% CH_3_CN/20 mM ammonium acetate with a flow rate of 6 mL/min. The retention time was 16 min. **12** was obtained as a yellow solid (53.0 mg, 0.127 mmol, 79% yield).

*(Z)-N-[3-(4-Fluorobutyl)-4,5-dimethylthiazole-2(3H)-ylidene]-(dicarba-closo-dodecaboranyl)-carboxamide* (**15**, **LUZ5**, and **17**). *Procedure 1*.^28^*(Z)-N-[3-(4-Fluorobutyl)-4,5-dimethylthiazole-2(3H)-ylidene]-(1,2-dicarba-*closo*-dodecaboranyl)-carboxamide* (**15**). A solution of **8**_*o*_ (0.348 mmol, 0.987 equiv) in dry DCM (3 mL) was added to a solution of **14** (0.353 mmol, 1.00 equiv) in dry DCM (2 mL). Triethylamine (0.361 mmol, 1.02 equiv) was added, and the mixture was stirred for 48 h at rt. The reaction was quenched by the addition of H_2_O (2 mL), an aqueous saturated NaHCO_3_ solution (10 mL), and EtOAc (15 mL). The phases were separated, and the aqueous layer was washed with EtOAc (2 × 15 mL). The combined organic layers were dried over MgSO_4_ and filtered, and the solvent was removed under reduced pressure. The solid was stored under a nitrogen atmosphere and purified by column chromatography [*n*-hexane/EtOAc, 4:1 (v/v) → 1:1 (v/v)]. Compound **15** was obtained as a white solid (0.032 g, 0.086 mmol, 24% yield): ^1^H NMR (400 MHz, CDCl_3_) δ 1.46–3.25 (br, 10H), 1.82 (m, 4H), 2.24 (m, 6H), 4.18 (m, 2H), 4.31 (s, 1H), 4.51 (dt, ^2^*J*_HF_ = 47.4 Hz, ^3^*J* = 5.6 Hz, 2H); ^11^B{^1^H} NMR (128 MHz, CDCl_3_) δ −14.0 (s, 2B), −11.8 (s, 4B), −9.5 (s, 2B), −3.9 (s, 1B), −3.4 (s, 1B); ^13^C{^1^H} NMR (101 MHz, CDCl_3_) δ 11.2, 11.7, 25.2 (d, ^3^*J*_CF_ = 4.0 Hz), 27.6 (d, ^2^*J*_CF_ = 20.1 Hz), 46.7, 56.7, 75.4, 83.5 (d, ^1^*J*_CF_ = 165.6 Hz), 117.1, 129.4, 166.5, 166.8; HRMS (ESI+) *m*/*z* for C_12_H_26_B_10_FN_2_OS [M + H]^+^ 373.2761, calcd 373.2753.

*Procedure 2*.^79^ The appropriate thiazole was dissolved in dry CH_3_CN and heated to 80 °C. The respective dicarba-*closo*-dodecaborane-1-carboxylic acid chloride (**8**) in dry CH_3_CN was added, and the reaction mixture was stirred for 2 h under reflux. Triethylamine was added at once, and the mixture was stirred for 16–20 h at 80 °C. The reaction flask was placed in an ice bath, and the precipitate formed was isolated by filtration, suspended in CH_3_CN, and filtered. The solvent was removed under reduced pressure. The crude product was stored under nitrogen and purified by column chromatography [*n*-hexane/EtOAc, 4:1 (v/v) → 1:1 (v/v)]. (*Z*)-*N*-[3-(4-Fluorobutyl)-4,5-dimethylthiazole-2(3*H*)-ylidene]-(1,2-dicarba-*closo*-dodecaboranyl)-carboxamide (**15**) was synthesized from **14** (0.353 mmol, 1.00 equiv) in dry CH_3_CN (1.3 mL), **8**_*o*_ (0.348 mmol, 0.987 equiv) in dry CH_3_CN (1.6 mL), triethylamine (0.361 mmol, 1.02 equiv), suspension in CH_3_CN (5 mL), yield of compound **15**: 0.041 g (0.110 mmol, 31%), white solid; ^1^H NMR (400 MHz, CDCl_3_) δ 1.46–3.24 (br, 10H), 1.82 (m, 4H), 2.25 (s, 6H), 4.18 (m, 2H), 4.31 (s, 1H), 4.51 (dt, ^2^*J*_HF_ = 47.5 Hz, ^3^*J* = 5.5 Hz, 2H); ^11^B{^1^H} NMR (128 MHz, CDCl_3_) δ −14.0 (s, 2B), −11.7 (s, 4B), −9.5 (s, 2B), −4.0 (s, 1B), −3.4 (s, 1B); HRMS (ESI+) *m*/*z* for C_12_H_26_B_10_FN_2_OS [M + H]^+^ 373.2753, calcd 373.2753; melting range 184–187 °C.

*(Z)-N-[3-(4-Fluorobutyl)-4,5-dimethylthiazole-2(3H)-ylidene]-(1,7-dicarba-closo-dodecaboranyl)-carboxamide* (**LUZ5**) was synthesized from **14** (0.551 mmol, 1.00 equiv) in dry CH_3_CN (2.0 mL), **8**_*m*_ (0.561 mmol, 1.02 equiv) in dry CH_3_CN (2.6 mL), triethylamine (0.577 mmol, 1.05 equiv) in dry CH_3_CN (1.0 mL), suspension in CH_3_CN (15 mL), yield of compound **LUZ5**: 0.093 g (0.250 mmol, 45%), white solid; ^1^H NMR (400 MHz, CDCl_3_) δ 1.45–3.78 (br, 10H), 1.80 (m, 4H), 2.21 (s, 3H), 2.22 (s, 3H), 2.96 (s, 1H), 4.17 (t, ^3^*J* = 7.2 Hz, 2H), 4.51 (dt, ^2^*J*_HF_ = 47.6 Hz, ^3^*J* = 5.6 Hz, 2H); ^11^B{^1^H} NMR (128 MHz, CDCl_3_) δ −15.6 (s, 2B), −13.8 (s, 2B), −11.1 (s, 4B), −8.2 (s, 1B), −4.8 (s, 1B); ^13^C{^1^H} NMR (101 MHz, CDCl_3_) δ 11.3, 11.9, 25.2 (d, ^3^*J*_CF_ = 4.1 Hz), 27.8 (d, ^2^*J*_CF_ = 20.1 Hz), 46.7, 54.4, 83.6 (d, ^1^*J*_CF_ = 165.4 Hz), 116.5, 129.1, 167.0, 168.2; HRMS (ESI+) *m*/*z* for C_12_H_26_B_10_FN_2_OS [M + H]^+^ 373.2753, calcd 373.2753; melting range 199–202 °C.

*(Z)-N-[3-(4-Fluorobutyl)-4,5-dimethylthiazole-2(3H)-ylidene]-(1,12-dicarba-closo-dodecaboranyl)-carboxamide* (**17**) was synthesized from **14** (0.565 mmol, 1.00 equiv) in dry CH_3_CN (2.0 mL), **8**_*p*_ (0.847 mmol, 1.50 equiv) in dry CH_3_CN (2.8 mL), triethylamine (0.577 mmol, 1.02 equiv) in dry CH_3_CN (1.0 mL), suspension in CH_3_CN (15 mL), yield of compound **17**: 0.075 g (0.201 mmol, 36%), white solid; ^1^H NMR (400 MHz, CDCl_3_) δ 1.00–3.27 (br, 10H), 1.79 (m, 4H), 2.17 (s, 3H), 2.19 (s, 3H), 2.73 (s, 1H), 4.12 (t, ^3^*J* = 7.2 Hz, 2H), 4.51 (dt, ^2^*J*_HF_ = 47.7 Hz, ^3^*J* = 5.5 Hz, 2H); ^11^B{^1^H} NMR (128 MHz, CDCl_3_) δ −15.6 (s, 5B), −13.2 (s, 5B); ^13^C{^1^H} NMR (101 MHz, CDCl_3_) δ 11.2, 11.8, 25.1 (d, ^3^*J*_CF_ = 4.5 Hz), 27.8 (d, ^2^*J*_CF_ = 20.1 Hz), 46.6, 60.7, 83.7 (d, ^1^*J*_CF_ = 165.4 Hz), 116.2, 128.9, 167.0, 168.9; HRMS (ESI+) *m*/*z* for C_12_H_26_B_10_FN_2_OS [M + H]^+^ 373.2755, calcd 373.2753; melting range 205–213 °C.

*(Z)-N-[4,5-Dimethylthiazole-2(3H)-ylidene]-(1,7-dicarba-closo-dodecaboranyl)-carboxamide* (**18**). The preparation of the compound was analogous to procedure 2^79^ given above, synthesized from **13** (1.47 mmol, 1.00 equiv) in dry CH_3_CN (6 mL), **10**_*m*_ (1.50 mmol, 1.02 equiv) in dry CH_3_CN (8 mL), and triethylamine (1.51 mmol, 1.03 equiv) in dry CH_3_CN (4 mL), via column chromatography with 100% *n*-hexane → 4:1 (v/v) *n*-hexane/EtOAc → 1:1 (v/v) *n*-hexane/EtOAc → 100% EtOAc, yielding 0.113 g (0.379 mmol, 26%) of compound **18** as a white solid: ^1^H NMR (400 MHz, CDCl_3_) δ 1.45–3.87 (br, 10H), 2.21 (s, 3H), 2.27 (s, 3H), 3.09 (s, 1H), 8.74 (s, 1H); ^11^B{^1^H} NMR (128 MHz, CDCl_3_) δ −15.6 (s, 2B), −13.0 (s, 2B), −11.5 (s, 1B), −10.6 (s, 2B), −6.6 (s, 2B), −5.8 (s, 1B).

*(Z)-N-[3-(4-Fluorobutyl-d_8_)-4,5-dimethylthiazole-2(3H)-ylidene]-(1,7-dicarba-closo-dodecaboranyl)-carboxamide* (**19**).^28^ NaH (0.027 g, 1.13 mmol, 2.98 equiv) and 1,4-dibromobutane-*d*_8_ (0.5 mL, 0.937 g, 4.18 mmol, 11.0 equiv) were added to a solution of **18** (0.113 g, 0.379 mmol, 1.00 equiv) in dry DMF (0.5 mL), and it was stirred for 21 h at rt. Water (2 mL) was added to stop the reaction. NaHCO_3_ (15 mL) and EtOAc (20 mL) were added, and the phases were separated. After washing with EtOAc (20 mL), the organic phase was dried over MgSO_4_ and filtered, and the solvents were removed under vacuum. The crude product was purified by column chromatography [*n*-hexane/EtOAc, 5:1 (v/v) → 2:1 (v/v), yielding 0.034 g (0.078 mmol, 20%) of compound **19** as a white solid: ^1^H NMR (400 MHz, CDCl_3_) δ 1.00–3.88 (br, 10H), 2.21 (s, 3H), 2.23 (s, 3H), 2.96 (s, 1H); ^11^B{^1^H} NMR (128 MHz, CDCl_3_) δ −15.5 (s, 2B), −13.7 (s, 2B), −11.1 (s, 4B), −8.1 (s, 1B), −4.7 (s, 1B); ^13^C{^1^H} NMR (101 MHz, CDCl_3_) δ 11.3, 11.8, 26.2 (m), 28.6 (m), 32.3 (m), 45.5 (m), 54.4, 116.5, 129.1, 166.9, 168.1; HRMS (ESI+) *m*/*z* for C_12_H_18_D_8_B_10_BrN_2_OS [M + H]^+^ 442.2446, calcd 442.2418.

Compound **19** (10 mg, 0.023 mmol) was purified by HPLC using a Reprosil-Pur 120 C18-AQ (250 mm × 10 mm, 5 μm) column, with 75% aqueous CH_3_CN with a flow rate of 5 mL/min. The retention time was 18 min.

### Stability

Stability tests were performed with ^1^H and ^11^B{^1^H} NMR spectroscopy. Approximately 10 mg of each compound was dissolved in aqueous DMSO-*d*_6_ and measured directly thereafter and at increasing time intervals from a few minutes to a couple of days or beyond a year.

The stability of **LUZ5** was additionally investigated under the conditions described above, but in the presence of larger amounts of H_2_O or D_2_O. For this, **LUZ5** was dissolved in DMSO-*d*_6_ (0.5 mL) and either H_2_O (0.1 mL) or D_2_O (0.1 mL) was added.

### Binding Affinity

The *in vitro* binding affinity assays were performed with membrane homogenates from Chinese hamster ovary (CHO) cells stably transfected with the human CB_2_R according to a previously published protocol.^95^ The screening for CB_1_R binding affinity determination was performed with membrane homogenates from CHO cells stably transfected with the human CB_1_R as described previously.^96^

### *In Vitro* Metabolism Studies

For *in vitro* evaluation of metabolites, the following instruments were used: BioShake iQ (QUANTIFOIL Instruments, Jena, Germany), Centrifuge 5424 (Eppendorf, Hamburg, Germany), UltiMate 3000 UHPLC System (Thermo Scientific, Germering, Germany) including a DAD detector (DAD-3000RS) coupled to an MSQ Plus single quadrupole mass spectrometer (Thermo Scientific, Austin, TX). Compounds **9**–**11**, **15**, **LUZ5**, and **17** were investigated with regard to their metabolism *in vitro* by incubation with mouse liver microsomes (MLMs) and analyses by HPLC-UV-MS according to an already published protocol.^81,82^ In brief, each of the test compounds (dissolved in DMSO, final concentration of 10 μM, final DMSO percentage of 1%) and MLMs (final protein concentration of 1 mg/mL) in PBS (pH 7.4) were preincubated in PBS (pH 7.4) for 5 min at 37 °C. Similarly preincubated NADPH (final concentration of 2 mM) was added to start the incubation. The final volume of each incubation mixture was 250 μL, and samples were prepared in duplicate. After the mixture had been gently shaken for 30 or 60 min at 37 °C, 1 mL of ice-cold CH_3_CN was added. The mixture was shaken vigorously for 30 s, rested on ice for 5 min, shaken for an additional 30 s, and then centrifuged at 14 000 rpm for 10 min. The supernatants were stored at 4 °C until they were measured by HPLC-UV-MS. As a positive control, samples with testosterone (final concentration of 20 μM) as the substrate were prepared like the test compounds, with or without NADPH and incubation for 60 or 90 min. Negative controls without NADPH as well as without both NADPH and MLM were also prepared for each test substance as well as controls without a substrate.

HPLC-UV-MS was performed on a Poroshell 120 EC-C18 column (100 mm × 3 mm, 2.7 μm) (Agilent Technologies, Waldbronn, Germany) at 25 °C (eluent A, H_2_O and 0.1% formic acid; eluent B, CH_3_CN and 0.1% formic acid) at a flow rate of 0.7 mL/min and monitoring a wavelength of 323 nm. The gradient elution method was as follows: 20% B from 0 to 1.5 min, 20% to 100% B from 1.5 to 10 min, 100% B from 10 to 12 min, and 20% B from 12 to 15 min. The MSQ Plus single quadrupole mass spectrometer was operated in positive and negative electrospray ionization mode: probe temperature of 550 °C, needle voltage of 3 V, and cone voltage of 75 V. On the basis of the obtained UV chromatograms, the percentage of unchanged compound was calculated from the peak area divided by the sum of peak areas of all relevant signals.

### Radiochemistry

#### Automated Radiosynthesis of **[^18^F]LUZ5-*d*_8_**

Remote-controlled radiosynthesis was performed using a Synchrom R&D EVO III automated synthesizer (Elysia-Raytest). Briefly, [^18^F]fluoride (5–12 GBq) was trapped on a Waters QMA cartridge, eluted with a H_2_O/CH_3_CN [1 mL, 1:4 (v/v)] solution containing 2.2.2-cryptand (K_2.2.2_) (11 mg) and K_2_CO_3_ (75 μL) into the reaction vessel, and dried via azeotropic distillation. To complete the azeotropic distillation, additional dry CH_3_CN (1.5 mL) was added. After complete dryness, precursor **19** (1 mg, 22.65 μmol) in CH_3_CN (1 mL) was added and the reaction mixture was stirred at 90 °C for 10 min. The reaction mixture was diluted with 4 mL of H_2_O/CH_3_CN (1:1), and the solution was transferred to the semipreparative HPLC instrument. **[**^**18**^**F]LUZ5-*d***_**8**_ was collected via the HPLC collection vial containing 40 mL of H_2_O and trapped in the Sep-Pak C18 light cartridge. The cartridge was washed with 2 mL of H_2_O, and **[**^**18**^**F]LUZ5-*d***_**8**_ was eluted with 1.3 mL of EtOH. This ethanolic solution was transferred outside of the shielded cell; the solvent was evaporated at 70 °C in a gentle stream of nitrogen for 5–10 min, and **[**^**18**^**F]LUZ5-*d***_**8**_ was reconstituted in an isotonic saline solution for further biological characterization. The total synthesis time was ∼85 min.

#### Quality Control

Radio-thin-layer chromatography was performed on Alugram SIL G/UV254 precoated plates (Macherey-Nagel) with PE:EtOAc [1:1 (v/v)]. The plates were exposed to storage phosphor screens (BAS IP MS 2025 E, GE Healthcare Europe GmbH, Freiburg, Germany), and images were recorded using the Amersham Typhoon RGB Biomolecular Imager (GE Healthcare Life Sciences). Images were quantified with ImageQuant version TL8.1 (GE Healthcare Life Sciences).

Analytical chromatographic separations were performed on a JASCO LC-2000 system, incorporating a PU-2080Plus pump, a model AS-2055Plus auto injector (100 μL sample loop), and a model UV-2070Plus detector coupled with a γ-detector (GABI Star; raytest Isotopenmessgeräte GmbH, Straubenhardt, Germany). Data analysis was performed with the Galaxie chromatography software (Agilent Technologies) using the chromatograms obtained at 254 nm.

The radiochemical yield, radiochemical purity, and analyses of plasma and brain samples were assessed via reverse phase HPLC (RP-HPLC) in gradient mode (10% CH_3_CN/20 mM NH_4_OAc_aq_ from 0 to 5 min, 10% → 90% CH_3_CN/20 mM NH_4_OAc_aq_ from 5 to 18 min, 90% CH_3_CN/20 mM NH_4_OAc_aq_ from 18 to 25 min, 90% → 10% CH_3_CN/20 mM NH_4_OAc_aq_ from 25 to 26 min, and 10% CH_3_CN/20 mM NH_4_OAc_aq_ from 26 to 30 min).

The molar activity was determined using analytical radio-HPLC with a Reprosil-Pur C18-AQ column (250 mm × 4.6 mm, 5 μm) and 66% CH_3_CN/20 mM NH_4_OAc_aq_ as the eluent at a flow rate of 1 mL/min and UV detection at 312 nm.

#### Determination of Lipophilicity (logD_7.4_)

The logD_7.4_ of **[**^**18**^**F]LUZ5-*****d***_**8**_ was experimentally determined in *n*-octanol/phosphate-buffered saline (PBS; 0.01 M, pH 7.4) at rt by the shake-flask method. The measurement was performed twice in triplicate.^22^

#### Quantification of Radiometabolites

20 to 30 megabecquerels of **[**^**18**^**F]LUZ5-*****d***_**8**_ dissolved in ∼150 μL of isotonic saline was administered intravenously as a bolus in the tail vein of awake female CD-1 mice weighing ∼33 g (*n* = 3) and male Wistar rats weighing 240 and 380 g (*n* = 2). At 30 min p.i., the animals were anesthetized and blood was withdrawn by retrobulbar bleeding using glass capillaries. Immediately afterward, the animals were euthanized by cervical dislocation, and the released urine was sampled. Blood plasma was obtained from the whole blood sample by centrifugation (2 min, 8000 rpm, room temperature). In addition, the brain and spleen were isolated and homogenized in 1 mL of demineralized water on ice (1000 rpm, 10 strokes; glass vessel, PTFE plunger; Potter S, B. Braun Biotech International, Goettingen, Germany).

The samples were further processed for subsequent radio-chromatographic analyses. Two consecutive extractions were performed as duplicates for plasma and brain determinations. Plasma and brain samples were added to an ice-cold mixture of either MeOH and H_2_O [9:1 (v/v); *n* = 3] or CH_3_CN and H_2_O [9:1 (v/v); *n* = 1] with extraction efficiencies of ≥94% for the former and ≥89% for the latter. The samples were vortexed for 3 min, incubated on ice for 5 min, vortexed for 3 min, and centrifuged at 10 000 rpm for 5 min. Supernatants were collected; the precipitates were redissolved in 100 μL of extraction solvent, and the extraction procedure was repeated. The activities of supernatants and precipitates were measured in a γ-counter (1480 WIZARD, Fa. PerkinElmer), and the extraction efficiencies were calculated as the ratio of radioactivity in the supernatant to the radioactivity in the original sample (supernatant + precipitate). The supernatants from both extractions were combined, concentrated at 70 °C under argon up to a remaining volume of 100 μL, and subsequently analyzed by analytical radio-HPLC with a gradient system (see Quality Control).

### PET Experiments

The *in vivo* biodistribution of **[**^**18**^**F]LUZ5-*d***_**8**_ in male Wistar rats and a female CD-1 mouse was assessed by dynamic small animal PET (Nanoscan, Mediso, Budapest, Hungary), 30 min recordings, followed by T1-weighted (GRE, TR/TE = 15.0/2.4 ms, 252/252, FA = 25°) magnetic resonance (MR) imaging with whole-body coils for anatomical correlation and attenuation correction. Animals were initially anesthetized with 5% isoflurane and placed on a thermostatically heated animal bed where anesthesia was maintained with 2% isoflurane in 60% oxygen/38% room air. The radiotracer was injected into the lateral tail vein (bolus within 5 s) at the start of the PET acquisition.

For the baseline study, 15 ± 3 MBq of the radiotracer was injected into three rats with a body weight of 230 ± 13 g. The blocking studies were conducted by the injection of 1.5 mg of the CB_2_R agonist **GW405833** per kilogram (predissolved in 1:2:7 DMSO/Kolliphor/0.9% NaCl) 10 min prior to the radiotracer (21.6 ± 0.9 MBq) into three rats weighing 228 ± 10 g. Brain uptake studies were performed with two rats (body weights of 213 and 242 g, injected doses of 15.2 and 17.0 MBq, respectively); for the whole body biodistribution study in mouse, one animal was used (body weight of 30.6 g, injected dose of 15.2 MBq).

List-mode PET data were binned as a series of attenuation-corrected sinogram frames and reconstructed by ordered subset expectation maximization (OSEM3D) with four iterations, six subsets, and a voxel size of 0.4 mm^3^ (Nucline version 2.01, Mediso). The analysis of reconstructed data sets was performed with PMOD version 4.103 (PMOD Technologies LLC, Zurich, Switzerland). Non-parametrical analysis of achieved time-activity curves (TACs) was performed with Microsoft Excel to determine the time to peak, the TAC peak value, and the area under the curve (AUC):
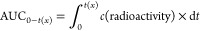
where *c*(radioactivity) is expressed as the standardized uptake value normalized to the body weight in grams (SUV).

Data are shown as means ± the standard deviation. Group differences were tested by a Student’s *t* test, with *p* < 0.05 designated as significant. Graphs were generated with GraphPad Prism version 9.3.1.

## Supporting Information Available

Molecular formula strings of compounds **9**−**12**, **15**, **LUZ5**, **17**, **18**, **19**, **[^18^F]LUZ5** (CSV)
Crystallographic data for compounds **9**−**11**, **15**, **LUZ5**, **17** (CIF)
checkCIF/PLATON report for compounds **9**−**11**, **15**, **LUZ5**, **17** (PDF)
The crystallographic data of CB_2_R containing the synthetic ligand AM2051 (Protein Data Bank, PDB ID: 5ZTY) (PDB)
Docking of compound **9** into the binding pocket of CB_2_R (Protein Data Bank, PDB ID: 5ZTY) (PDB)
Docking of compound **15** into the binding pocket of CB_2_R (Protein Data Bank, PDB ID: 5ZTY) (PDB)
Docking of compound **LUZ5** into the binding pocket of CB_2_R (Protein Data Bank, PDB ID: 5ZTY) (PDB)
NMR spectra of compounds **9**–**12**, **15**, **LUZ5**, **17**–**19**, and **SP1**; HRMS-ESI spectra of compounds **9**–**11**, **15**, **LUZ5**, **17**, **19**, and **SP1**; HPLC purity determination of compounds **9**–**12**, **15**, **LUZ5**, and **17**; stability data of compounds **9**–**11**, **15**, **LUZ5**, and **17**; X-ray data of compounds **9**–**11**, **15**, **LUZ5**, and **17**; *in vitro* binding assay; inhibition curves of **9**–**11**, **15**, **LUZ5**, and **17** with the CB_2_R and CB_1_R; docking data of compounds **9**, **15**, and **LUZ5**; *in vitro* metabolism studies (exemplified spectrum) of **LUZ5**; UV profile of isolated **[**^**18**^**F]LUZ5-*d***_**8**_; additional biodistribution data of **[**^**18**^**F]LUZ5-*d***_**8**_; and chemical structures of CB_2_R agonists and the CB_1_R antagonist/inverse agonist (PDF)

## Supplementary Material

jm3c00195_si_001.csv

jm3c00195_si_002.cif

jm3c00195_si_003.pdf

jm3c00195_si_004.pdb

jm3c00195_si_005.pdb

jm3c00195_si_006.pdb

jm3c00195_si_007.pdb

## Accession Codes

PDB entry 5ZTY for the CB_2_R with synthetic ligand AM2051.

## Abbreviations

AD: Alzheimer’s disease
AUC: area under the curve
BBB: blood–brain barrier
Boc: *tert*-butyloxycarbonyl
br: broad signal
CB_1_R: cannabinoid receptor type 1
CB_2_R: cannabinoid receptor type 2
CHO: Chinese hamster ovary
CNS: central nervous system
COSY: correlated spectroscopy
d: doublet
DCM: dichloromethane
dd: doublet of doublets
DMF: *N*,*N*-dimethylformamide
DMSO: dimethyl sulfoxide
dt: doublet of triplets
ECS: endocannabinoid system
ESI: electrospray ionization
Et_3_N: triethylamine
GPCR or GPR: G protein-coupled receptor
HLMs: human liver microsomes
HMBC: heteronuclear multiple-bond correlation
HPLC: high-performance liquid chromatography
HRMS: high-resolution mass spectrometry
HSQC: heteronuclear single quantum coherence
i.v.: intravenously
K_2.2.2._: 2.2.2-cryptand
*K*_i_: inhibition constant
m: multiplet
MIP: maximal intensity projection
MLMs: mouse liver microsomes
MR: magnetic resonance
NADPH: nicotinamide adenine dinucleotide phosphate
*n*-BuLi: *n*-butyllithium
NMR: nuclear magnetic resonance
p.i.: post-injection
PBS: phosphate-buffered saline
PET: positron emission tomography
RP: reverse phase
rt: room temperature
s: singlet
SUV: standardized uptake value
SUVr: SUV ratio of *x* to reference
t: triplet
TAC: time–activity curve
TFA: trifluoroacetic acid
THC: (−)-Δ^9^-*trans*-tetrahydrocannabinol
TLC: thin-layer chromatography
TPV: TAC peak values
TTP: time to peak
*V*_vdW_: van der Waals volume
