# Supplementary material for: Development of the High-Affinity Carborane-Based Cannabinoid Receptor Type 2 PET Ligand [18F]LUZ5-d8
Source: J Med Chem. 2023 Mar 21;66(7):5242–60. doi: 10.1021/acs.jmedchem.3c00195 (PMC10782483; doi:10.1021/acs.jmedchem.3c00195)

checkCIF/PLATON report

Structure factors have been supplied for datablock(s) comp_luz1_x3301fin, comp_luz2_x3302fin, comp_luz4_x3594fin, comp_luz5_x3531fin, comp_luz6_x3609fin, comp_luz7_x3551fin

THIS REPORT IS FOR GUIDANCE ONLY. IF USED AS PART OF A REVIEW PROCEDURE FOR PUBLICATION, IT SHOULD NOT REPLACE THE EXPERTISE OF AN EXPERIENCED CRYSTALLOGRAPHIC REFEREE.

No syntax errors found.   CIF dictionary   Interpreting this report

Datablock: comp_luz1_x3301fin

Bond precision: C-C = 0.0030 A   Wavelength=0.71073

Cell:	a=15.3336(8)	b=7.3247(3)	c=18.8973(9)
	alpha=90	beta=105.003(5)	gamma=90
Temperature:	130 K		

	Calculated	Reported
Volume	2050.08(17)	2050.08(17)
Space group	P 21/n	P 21/n
Hall group	-P 2yn	-P 2yn
Moiety formula	C15 H24 B10 F N3 O2	C15 H24 B10 F N3 O2
Sum formula	C15 H24 B10 F N3 O2	C15 H24 B10 F N3 O2
Mr	405.47	405.47
Dx,g cm-3	1.314	1.314
Z	4	4
Mu (mm-1)	0.084	0.084
F000	840.0	840.0
F000'	840.30	
h,k,lmax	20,9,25	20,9,24
Nref	5078	4476
Tmin,Tmax	0.997,0.998	0.715,1.000
Tmin'	0.967	

Correction method= # Reported T Limits: Tmin=0.715 Tmax=1.000
AbsCorr = MULTI-SCAN

Data completeness= 0.881   Theta(max)= 28.262

R(reflections)= 0.0565(2702)   wR2(reflections)= 0.1427(4476)

S = 1.011   Npar= 401

The following ALERTS were generated. Each ALERT has the format
test-name_ALERT_alert-type_alert-level.
Click on the hyperlinks for more details of the test.

Alert level C

```
PLAT220_ALERT_2_C NonSolvent   Resd 1  C   Ueq(max)/Ueq(min) Range        3.8 Ratio
PLAT220_ALERT_2_C NonSolvent   Resd 1  O   Ueq(max)/Ueq(min) Range        3.5 Ratio
PLAT222_ALERT_3_C NonSolvent Resd 1  H   Uiso(max)/Uiso(min) Range        7.1 Ratio
PLAT906_ALERT_3_C Large K Value in the Analysis of Variance ......      4.634 Check
```

Alert level G

```
PLAT002_ALERT_2_G Number of Distance or Angle Restraints on AtSite         39 Note
PLAT003_ALERT_2_G Number of Uiso or Uij Restrained non-H Atoms ...         19 Report
PLAT007_ALERT_5_G Number of Unrefined Donor-H Atoms ..............          2 Report
PLAT172_ALERT_4_G The CIF-Embedded .res File Contains DFIX Records          4 Report
PLAT174_ALERT_4_G The CIF-Embedded .res File Contains FLAT Records          1 Report
PLAT175_ALERT_4_G The CIF-Embedded .res File Contains SAME Records          1 Report
PLAT176_ALERT_4_G The CIF-Embedded .res File Contains SADI Records          2 Report
PLAT178_ALERT_4_G The CIF-Embedded .res File Contains SIMU Records          1 Report
PLAT301_ALERT_3_G Main Residue  Disorder ..............(Resd  1  )        61% Note
PLAT343_ALERT_2_G Unusual sp?      Angle Range in Main Residue for         C1 Check
PLAT367_ALERT_2_G Long?  C(sp?)-C(sp?) Bond  C1       - C2       .       1.68 Ang.
PLAT720_ALERT_4_G Number of Unusual/Non-Standard Labels ..........          2 Note
PLAT811_ALERT_5_G No ADDSYM Analysis: Too Many Excluded Atoms ....          ! Info
PLAT860_ALERT_3_G Number of Least-Squares Restraints .............         80 Note
PLAT910_ALERT_3_G Missing # of FCF Reflection(s) Below Theta(Min).          1 Note
PLAT912_ALERT_4_G Missing # of FCF Reflections Above STh/L=  0.600        596 Note
PLAT941_ALERT_3_G Average HKL Measurement Multiplicity ...........        3.8 Low
PLAT978_ALERT_2_G Number C-C Bonds with Positive Residual Density.          4 Info
```

0 **ALERT level A** = Most likely a serious problem - resolve or explain
0 **ALERT level B** = A potentially serious problem, consider carefully
4 **ALERT level C** = Check. Ensure it is not caused by an omission or oversight
18 **ALERT level G** = General information/check it is not something unexpected

0 ALERT type 1 CIF construction/syntax error, inconsistent or missing data
7 ALERT type 2 Indicator that the structure model may be wrong or deficient
6 ALERT type 3 Indicator that the structure quality may be low
7 ALERT type 4 Improvement, methodology, query or suggestion
2 ALERT type 5 Informative message, check

Datablock: comp_luz2_x3302fin

Bond precision:	C-C = 0.0026 A		Wavelength=0.71073
Cell:	a=7.2038(2)	b=24.2149(6)	c=10.9508(3)
	alpha=90	beta=93.956(2)	gamma=90
Temperature:	130 K		

	Calculated	Reported
Volume	1905.70(9)	1905.70(9)
Space group	P 21/n	P 21/n
Hall group	-P 2yn	-P 2yn
Moiety formula	C12 H25 B10 F N2 O S	C12 H25 B10 F N2 O S
Sum formula	C12 H25 B10 F N2 O S	C12 H25 B10 F N2 O S
Mr	372.50	372.50
Dx,g cm-3	1.298	1.298
Z	4	4
Mu (mm-1)	0.184	0.184
F000	776.0	776.0
F000'	776.72	
h,k,lmax	10,34,15	10,34,15
Nref	5814	5284
Tmin,Tmax	0.946,0.991	0.960,1.000
Tmin'	0.946	

Correction method= # Reported T Limits: Tmin=0.960 Tmax=1.000
AbsCorr = MULTI-SCAN

Data completeness= 0.909 Theta(max)= 30.479

R(reflections)= 0.0493(3941) wR2(reflections)= 0.1206(5284)

S = 1.023 Npar= 344

The following ALERTS were generated. Each ALERT has the format
test-name_ALERT_alert-type_alert-level.
Click on the hyperlinks for more details of the test.

Alert level C

```
PLAT222_ALERT_3_C NonSolvent Resd 1   H   Uiso(max)/Uiso(min) Range        4.3 Ratio
PLAT906_ALERT_3_C Large K Value in the Analysis of Variance ......      6.015 Check
```

Alert level G

```
PLAT002_ALERT_2_G Number of Distance or Angle Restraints on AtSite          4 Note
PLAT176_ALERT_4_G The CIF-Embedded .res File Contains SADI Records          2 Report
PLAT343_ALERT_2_G Unusual sp?      Angle Range in Main Residue for         C1 Check
PLAT343_ALERT_2_G Unusual sp?      Angle Range in Main Residue for         C2 Check
PLAT367_ALERT_2_G Long?  C(sp?)-C(sp?) Bond  C1       - C2       .       1.63 Ang.
PLAT367_ALERT_2_G Long?  C(sp?)-C(sp?) Bond  C2       - C3       .       1.52 Ang.
PLAT860_ALERT_3_G Number of Least-Squares Restraints .............          6 Note
PLAT912_ALERT_4_G Missing # of FCF Reflections Above STh/L=  0.600        531 Note
PLAT941_ALERT_3_G Average HKL Measurement Multiplicity ...........        3.4 Low
PLAT978_ALERT_2_G Number C-C Bonds with Positive Residual Density.          2 Info
```

```
   0 ALERT level A = Most likely a serious problem - resolve or explain
   0 ALERT level B = A potentially serious problem, consider carefully
   2 ALERT level C = Check. Ensure it is not caused by an omission or oversight
  10 ALERT level G = General information/check it is not something unexpected

   0 ALERT type 1 CIF construction/syntax error, inconsistent or missing data
   6 ALERT type 2 Indicator that the structure model may be wrong or deficient
   4 ALERT type 3 Indicator that the structure quality may be low
   2 ALERT type 4 Improvement, methodology, query or suggestion
   0 ALERT type 5 Informative message, check
```

Datablock: comp_luz4_x3594fin

Bond precision: C-C = 0.0016 A Wavelength=0.71073

Cell:	a=6.9499(2)	b=12.2343(5)	c=13.0166(5)
	alpha=109.825(4)	beta=94.822(3)	gamma=92.968(3)
Temperature:	130 K		

	Calculated	Reported
Volume	1033.67(7)	1033.67(7)
Space group	P -1	P -1
Hall group	-P 1	-P 1
Moiety formula	C15 H24 B10 F N3 O2	C15 H24 B10 F N3 O2
Sum formula	C15 H24 B10 F N3 O2	C15 H24 B10 F N3 O2
Mr	405.47	405.47
Dx,g cm-3	1.303	1.303
Z	2	2
Mu (mm-1)	0.083	0.083
F000	420.0	420.0
F000'	420.15	
h,k,lmax	10,18,19	10,18,19
Nref	7481	6887
Tmin,Tmax	0.964,0.993	0.993,1.000
Tmin'	0.964	

Correction method= # Reported T Limits: Tmin=0.993 Tmax=1.000
AbsCorr = MULTI-SCAN

Data completeness= 0.921 Theta(max)= 32.473

R(reflections)= 0.0489(5493) wR2(reflections)= 0.1370(6887)

S = 1.024 Npar= 376

The following ALERTS were generated. Each ALERT has the format
test-name_ALERT_alert-type_alert-level.
Click on the hyperlinks for more details of the test.

Alert level C

PLAT906_ALERT_3_C Large K Value in the Analysis of Variance ...... 3.141 Check

Alert level G

PLAT343_ALERT_2_G Unusual sp? Angle Range in Main Residue for C1 Check
PLAT343_ALERT_2_G Unusual sp? Angle Range in Main Residue for C2 Check
PLAT720_ALERT_4_G Number of Unusual/Non-Standard Labels .... 1 Note
PLAT910_ALERT_3_G Missing # of FCF Reflection(s) Below Theta(Min). 3 Note
PLAT912_ALERT_4_G Missing # of FCF Reflections Above STh/L= 0.600 592 Note
PLAT941_ALERT_3_G Average HKL Measurement Multiplicity .... 3.1 Low
PLAT978_ALERT_2_G Number C-C Bonds with Positive Residual Density. 12 Info
PLAT992_ALERT_5_G Repd & Actual _reflns_number_gt Values Differ by 3 Check

0 **ALERT level A** = Most likely a serious problem - resolve or explain
0 **ALERT level B** = A potentially serious problem, consider carefully
1 **ALERT level C** = Check. Ensure it is not caused by an omission or oversight
8 **ALERT level G** = General information/check it is not something unexpected

0 ALERT type 1 CIF construction/syntax error, inconsistent or missing data
3 ALERT type 2 Indicator that the structure model may be wrong or deficient
3 ALERT type 3 Indicator that the structure quality may be low
2 ALERT type 4 Improvement, methodology, query or suggestion
1 ALERT type 5 Informative message, check

Datablock: comp_luz5_x3531fin

Bond precision:	C-C = 0.0030 A		Wavelength=0.71073
Cell:	a=13.2567(4)	b=7.4299(2)	c=20.6105(6)
	alpha=90	beta=104.120(3)	gamma=90
Temperature:	293 K		

	Calculated	Reported
Volume	1968.72(10)	1968.72(10)
Space group	P 21/n	P 21/n
Hall group	-P 2yn	-P 2yn
Moiety formula	C12 H25 B10 F N2 O S	C12 H25 B10 F N2 O S
Sum formula	C12 H25 B10 F N2 O S	C12 H25 B10 F N2 O S
Mr	372.50	372.50
Dx,g cm-3	1.257	1.257
Z	4	4
Mu (mm-1)	0.178	0.178
F000	776.0	776.0
F000'	776.72	
h,k,lmax	17,9,27	17,9,26
Nref	4951	4464
Tmin,Tmax	0.973,0.984	0.996,1.000
Tmin'	0.931	

Correction method= # Reported T Limits: Tmin=0.996 Tmax=1.000
AbsCorr = MULTI-SCAN

Data completeness= 0.902 Theta(max)= 28.424

R(reflections)= 0.0501(2997) wR2(reflections)= 0.1427(4464)

S = 1.021 Npar= 284

The following ALERTS were generated. Each ALERT has the format
test-name_ALERT_alert-type_alert-level.
Click on the hyperlinks for more details of the test.

Alert level C

PLAT906_ALERT_3_C Large K Value in the Analysis of Variance ...... 5.148 Check

---

Alert level G

PLAT002_ALERT_2_G Number of Distance or Angle Restraints on AtSite 11 Note
PLAT003_ALERT_2_G Number of Uiso or Uij Restrained non-H Atoms ... 10 Report
PLAT168_ALERT_4_G The CIF-Embedded .res File Contains EXYZ Records 5 Report
PLAT171_ALERT_4_G The CIF-Embedded .res File Contains EADP Records 6 Report
PLAT176_ALERT_4_G The CIF-Embedded .res File Contains SADI Records 4 Report
PLAT178_ALERT_4_G The CIF-Embedded .res File Contains SIMU Records 2 Report
PLAT188_ALERT_3_G A Non-default SIMU Restraint Value has been used 0.0100 Report
PLAT188_ALERT_3_G A Non-default SIMU Restraint Value has been used 0.0100 Report
PLAT191_ALERT_3_G A Non-default SADI Restraint Value has been used 0.0100 Report
PLAT191_ALERT_3_G A Non-default SADI Restraint Value has been used 0.0100 Report
PLAT199_ALERT_1_G Reported _cell_measurement_temperature ..... (K) 293 Check
PLAT200_ALERT_1_G Reported _diffrn_ambient_temperature ..... (K) 293 Check
PLAT230_ALERT_2_G Hirshfeld Test Diff for B0 --B6 . 5.5 s.u.

```
PLAT300_ALERT_4_G Atom Site Occupancy of B0        Constrained at        0.8 Check
PLAT300_ALERT_4_G Atom Site Occupancy of B3        Constrained at        0.8 Check
PLAT300_ALERT_4_G Atom Site Occupancy of B5        Constrained at        0.8 Check
PLAT300_ALERT_4_G Atom Site Occupancy of B7        Constrained at        0.8 Check
PLAT300_ALERT_4_G Atom Site Occupancy of B10       Constrained at        0.8 Check
PLAT300_ALERT_4_G Atom Site Occupancy of C1        Constrained at        0.2 Check
PLAT300_ALERT_4_G Atom Site Occupancy of C1F       Constrained at        0.2 Check
PLAT300_ALERT_4_G Atom Site Occupancy of C1G       Constrained at        0.2 Check
PLAT300_ALERT_4_G Atom Site Occupancy of C1H       Constrained at        0.2 Check
PLAT300_ALERT_4_G Atom Site Occupancy of C1I       Constrained at        0.2 Check
PLAT300_ALERT_4_G Atom Site Occupancy of H0X       Constrained at        0.8 Check
PLAT300_ALERT_4_G Atom Site Occupancy of H3X       Constrained at        0.8 Check
PLAT300_ALERT_4_G Atom Site Occupancy of H5X       Constrained at        0.8 Check
PLAT300_ALERT_4_G Atom Site Occupancy of H7X       Constrained at        0.8 Check
PLAT300_ALERT_4_G Atom Site Occupancy of H10X      Constrained at        0.8 Check
PLAT300_ALERT_4_G Atom Site Occupancy of H11A      Constrained at        0.5 Check
PLAT300_ALERT_4_G Atom Site Occupancy of H11B      Constrained at        0.5 Check
PLAT300_ALERT_4_G Atom Site Occupancy of H11C      Constrained at        0.5 Check
PLAT300_ALERT_4_G Atom Site Occupancy of H11D      Constrained at        0.5 Check
PLAT300_ALERT_4_G Atom Site Occupancy of H11E      Constrained at        0.5 Check
PLAT300_ALERT_4_G Atom Site Occupancy of H11F      Constrained at        0.5 Check
PLAT300_ALERT_4_G Atom Site Occupancy of H12A      Constrained at        0.5 Check
PLAT300_ALERT_4_G Atom Site Occupancy of H12B      Constrained at        0.5 Check
PLAT300_ALERT_4_G Atom Site Occupancy of H12C      Constrained at        0.5 Check
PLAT300_ALERT_4_G Atom Site Occupancy of H12D      Constrained at        0.5 Check
PLAT300_ALERT_4_G Atom Site Occupancy of H12E      Constrained at        0.5 Check
PLAT300_ALERT_4_G Atom Site Occupancy of H12F      Constrained at        0.5 Check
PLAT300_ALERT_4_G Atom Site Occupancy of H1        Constrained at        0.2 Check
PLAT300_ALERT_4_G Atom Site Occupancy of H1F       Constrained at        0.2 Check
PLAT300_ALERT_4_G Atom Site Occupancy of H1G       Constrained at        0.2 Check
PLAT300_ALERT_4_G Atom Site Occupancy of H1H       Constrained at        0.2 Check
PLAT300_ALERT_4_G Atom Site Occupancy of H1I       Constrained at        0.2 Check
PLAT301_ALERT_3_G Main Residue  Disorder .............(Resd  1  )        37% Note
PLAT343_ALERT_2_G Unusual sp?      Angle Range in Main Residue for        C2 Check
PLAT367_ALERT_2_G Long?  C(sp?)-C(sp?) Bond  C2       - C3       .      1.52 Ang.
PLAT720_ALERT_4_G Number of Unusual/Non-Standard Labels .........          2 Note
PLAT773_ALERT_2_G Check long C-C Bond in CIF: C1F      --C1H            1.75 Ang.
PLAT773_ALERT_2_G Check long C-C Bond in CIF: C1G      --C1I            1.73 Ang.
PLAT773_ALERT_2_G Check long C-C Bond in CIF: C1H      --C1I            1.73 Ang.
PLAT811_ALERT_5_G No ADDSYM Analysis: Too Many Excluded Atoms ....         ! Info
PLAT860_ALERT_3_G Number of Least-Squares Restraints ............         71 Note
PLAT910_ALERT_3_G Missing # of FCF Reflection(s) Below Theta(Min).         3 Note
PLAT912_ALERT_4_G Missing # of FCF Reflections Above STh/L=  0.600       472 Note
PLAT941_ALERT_3_G Average HKL Measurement Multiplicity ..........        4.9 Low
PLAT978_ALERT_2_G Number C-C Bonds with Positive Residual Density.         0 Info
```

---

   0 **ALERT level A** = Most likely a serious problem - resolve or explain
   0 **ALERT level B** = A potentially serious problem, consider carefully
   1 **ALERT level C** = Check. Ensure it is not caused by an omission or oversight
  58 **ALERT level G** = General information/check it is not something unexpected

   2 ALERT type 1 CIF construction/syntax error, inconsistent or missing data
   9 ALERT type 2 Indicator that the structure model may be wrong or deficient
   9 ALERT type 3 Indicator that the structure quality may be low
  38 ALERT type 4 Improvement, methodology, query or suggestion
   1 ALERT type 5 Informative message, check

Datablock: comp_luz6_x3609fin

Bond precision:	C-C = 0.0030 A		Wavelength=0.71073
Cell:	a=22.6667(7)	b=7.4977(2)	c=24.4845(7)
	alpha=90	beta=97.632(3)	gamma=90
Temperature:	130 K		

	Calculated	Reported
Volume	4124.2(2)	4124.2(2)
Space group	I 2/a	I 2/a
Hall group	-I 2ya	-I 2ya
Moiety formula	C15 H24 B10 F N3 O2	C15 H24 B10 F N3 O2
Sum formula	C15 H24 B10 F N3 O2	C15 H24 B10 F N3 O2
Mr	405.47	405.47
Dx,g cm-3	1.306	1.306
Z	8	8
Mu (mm-1)	0.083	0.083
F000	1680.0	1680.0
F000'	1680.59	
h,k,lmax	30,9,32	29,9,31
Nref	5092	4617
Tmin,Tmax	0.985,0.992	0.947,1.000
Tmin'	0.963	

Correction method= # Reported T Limits: Tmin=0.947 Tmax=1.000
AbsCorr = MULTI-SCAN

Data completeness= 0.907 Theta(max)= 28.237

R(reflections)= 0.0557(3112) wR2(reflections)= 0.1453(4617)

S = 1.016 Npar= 372

The following ALERTS were generated. Each ALERT has the format
test-name_ALERT_alert-type_alert-level.
Click on the hyperlinks for more details of the test.

Alert level C

```
PLAT213_ALERT_2_C Atom C12           has ADP max/min Ratio .....        3.8 oblate
PLAT213_ALERT_2_C Atom C12F          has ADP max/min Ratio .....        3.8 oblate
PLAT906_ALERT_3_C Large K Value in the Analysis of Variance ......    2.448 Check
```

Alert level G

```
PLAT002_ALERT_2_G Number of Distance or Angle Restraints on AtSite         11 Note
PLAT171_ALERT_4_G The CIF-Embedded .res File Contains EADP Records          3 Report
PLAT172_ALERT_4_G The CIF-Embedded .res File Contains DFIX Records          1 Report
PLAT176_ALERT_4_G The CIF-Embedded .res File Contains SADI Records          3 Report
PLAT191_ALERT_3_G A Non-default SADI Restraint Value has been used     0.0030 Report
PLAT191_ALERT_3_G A Non-default SADI Restraint Value has been used     0.0030 Report
PLAT191_ALERT_3_G A Non-default SADI Restraint Value has been used     0.0030 Report
PLAT301_ALERT_3_G Main Residue  Disorder .............(Resd  1  )         16% Note
PLAT343_ALERT_2_G Unusual sp?      Angle Range in Main Residue for         C1 Check
PLAT343_ALERT_2_G Unusual sp?      Angle Range in Main Residue for         C2 Check
PLAT720_ALERT_4_G Number of Unusual/Non-Standard Labels .........           1 Note
PLAT860_ALERT_3_G Number of Least-Squares Restraints ............          14 Note
PLAT910_ALERT_3_G Missing # of FCF Reflection(s) Below Theta(Min).          2 Note
PLAT912_ALERT_4_G Missing # of FCF Reflections Above STh/L=  0.600        460 Note
PLAT978_ALERT_2_G Number C-C Bonds with Positive Residual Density.          3 Info
```

---

0 **ALERT level A** = Most likely a serious problem - resolve or explain
0 **ALERT level B** = A potentially serious problem, consider carefully
3 **ALERT level C** = Check. Ensure it is not caused by an omission or oversight
15 **ALERT level G** = General information/check it is not something unexpected

0 ALERT type 1 CIF construction/syntax error, inconsistent or missing data
6 ALERT type 2 Indicator that the structure model may be wrong or deficient
7 ALERT type 3 Indicator that the structure quality may be low
5 ALERT type 4 Improvement, methodology, query or suggestion
0 ALERT type 5 Informative message, check

Datablock: comp_luz7_x3551fin

Bond precision: C-C = 0.0019 A Wavelength=1.54184

Cell:	a=6.8918(2)	b=10.4396(3)	c=14.0592(3)
	alpha=99.793(2)	beta=100.469(2)	gamma=92.309(2)
Temperature:	130 K		

	Calculated	Reported
Volume	977.55(5)	977.54(5)
Space group	P -1	P -1
Hall group	-P 1	-P 1
Moiety formula	C12 H25 B10 F N2 O S	C12 H25 B10 F N2 O S
Sum formula	C12 H25 B10 F N2 O S	C12 H25 B10 F N2 O S
Mr	372.50	372.50
Dx,g cm-3	1.265	1.266
Z	2	2
Mu (mm-1)	1.561	1.561
F000	388.0	388.0
F000'	389.58	
h,k,lmax	8,12,16	8,12,16
Nref	3573	3551
Tmin,Tmax	0.755,0.869	0.835,1.000
Tmin'	0.519	

Correction method= # Reported T Limits: Tmin=0.835 Tmax=1.000
AbsCorr = MULTI-SCAN

Data completeness= 0.994 Theta(max)= 67.974

R(reflections)= 0.0368(3332) wR2(reflections)= 0.1015(3551)

S = 1.047 Npar= 344

The following ALERTS were generated. Each ALERT has the format
test-name_ALERT_alert-type_alert-level.
Click on the hyperlinks for more details of the test.

Alert level C

PLAT911_ALERT_3_C Missing FCF Refl Between Thmin & STh/L= 0.600 8 Report

---

Alert level G

PLAT154_ALERT_1_G The s.u.'s on the Cell Angles are Equal ..(Note) 0.002 Degree
PLAT343_ALERT_2_G Unusual sp? Angle Range in Main Residue for C1 Check
PLAT343_ALERT_2_G Unusual sp? Angle Range in Main Residue for C2 Check
PLAT367_ALERT_2_G Long? C(sp?)-C(sp?) Bond C2 - C3 . 1.53 Ang.
PLAT909_ALERT_3_G Percentage of I>2sig(I) Data at Theta(Max) Still 89% Note
PLAT912_ALERT_4_G Missing # of FCF Reflections Above STh/L= 0.600 14 Note
PLAT941_ALERT_3_G Average HKL Measurement Multiplicity .... 4.5 Low
PLAT978_ALERT_2_G Number C-C Bonds with Positive Residual Density. 4 Info

---

0 **ALERT level A** = Most likely a serious problem - resolve or explain
0 **ALERT level B** = A potentially serious problem, consider carefully
1 **ALERT level C** = Check. Ensure it is not caused by an omission or oversight

```
8 ALERT level G = General information/check it is not something unexpected

1 ALERT type 1 CIF construction/syntax error, inconsistent or missing data
4 ALERT type 2 Indicator that the structure model may be wrong or deficient
3 ALERT type 3 Indicator that the structure quality may be low
1 ALERT type 4 Improvement, methodology, query or suggestion
0 ALERT type 5 Informative message, check
```

It is advisable to attempt to resolve as many as possible of the alerts in all categories. Often the minor alerts point to easily fixed oversights, errors and omissions in your CIF or refinement strategy, so attention to these fine details can be worthwhile. In order to resolve some of the more serious problems it may be necessary to carry out additional measurements or structure refinements. However, the purpose of your study may justify the reported deviations and the more serious of these should normally be commented upon in the discussion or experimental section of a paper or in the "special_details" fields of the CIF. checkCIF was carefully designed to identify outliers and unusual parameters, but every test has its limitations and alerts that are not important in a particular case may appear. Conversely, the absence of alerts does not guarantee there are no aspects of the results needing attention. It is up to the individual to critically assess their own results and, if necessary, seek expert advice.

Publication of your CIF in IUCr journals

A basic structural check has been run on your CIF. These basic checks will be run on all CIFs submitted for publication in IUCr journals (*Acta Crystallographica*, *Journal of Applied Crystallography*, *Journal of Synchrotron Radiation*); however, if you intend to submit to *Acta Crystallographica Section C* or *E* or *IUCrData*, you should make sure that full publication checks are run on the final version of your CIF prior to submission.

Publication of your CIF in other journals

Please refer to the *Notes for Authors* of the relevant journal for any special instructions relating to CIF submission.

PLATON version of 28/11/2022; check.def file version of 28/11/2022

Datablock comp_luz1_x3301fin - ellipsoid plot

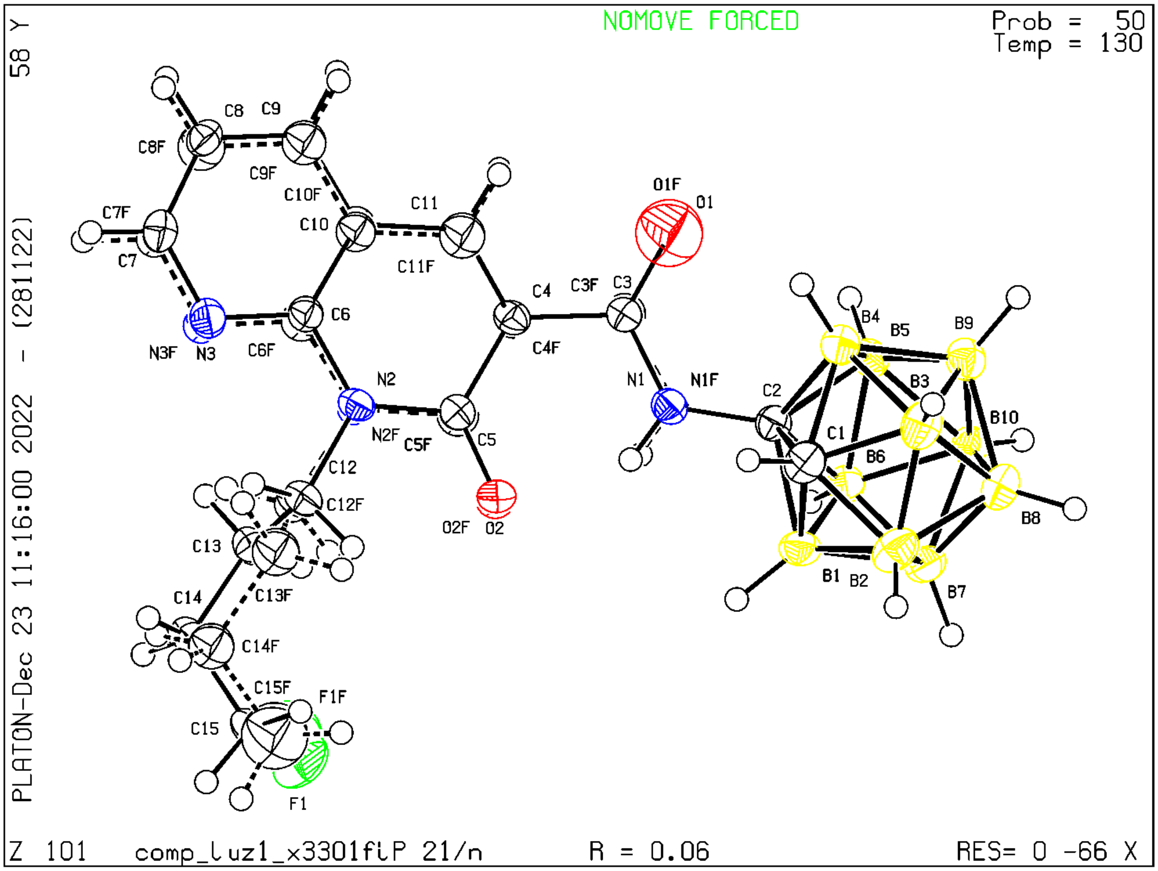

Datablock comp_luz2_x3302fin - ellipsoid plot

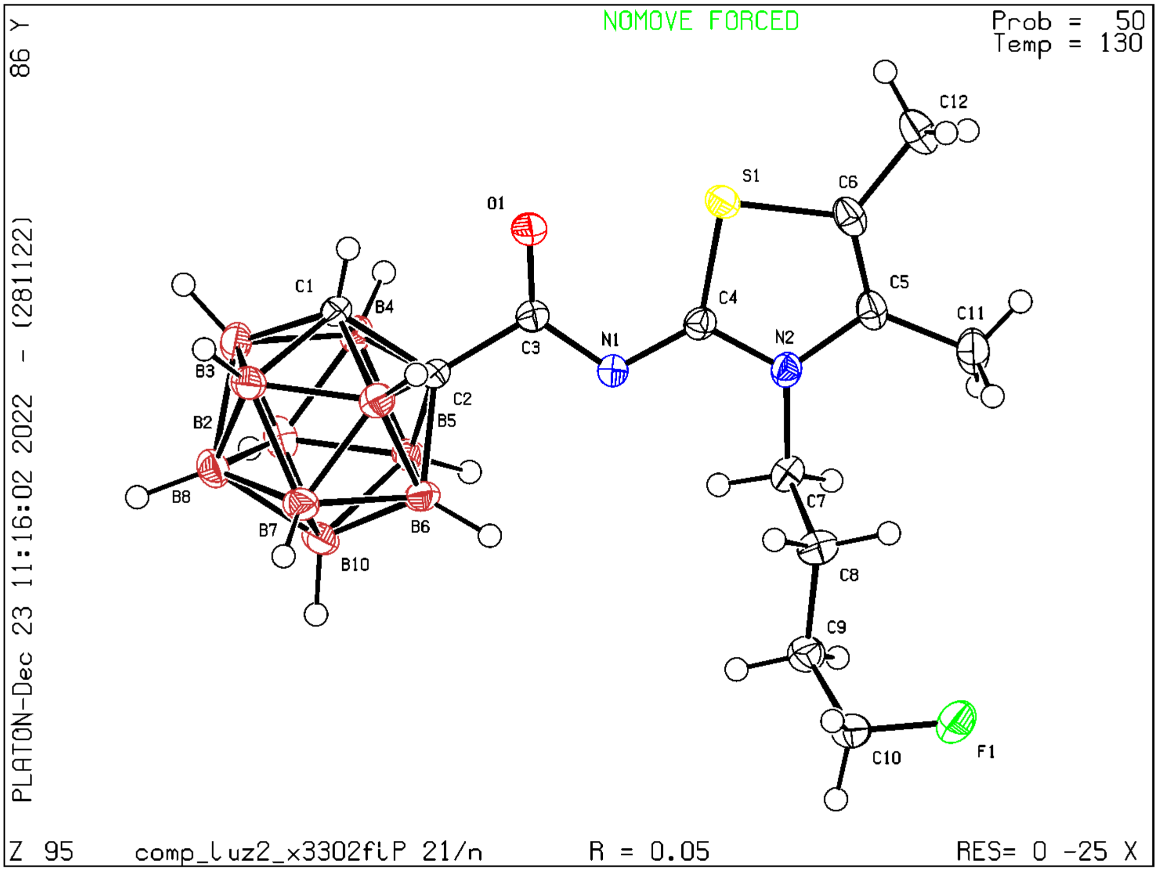

Datablock comp_luz4_x3594fin - ellipsoid plot

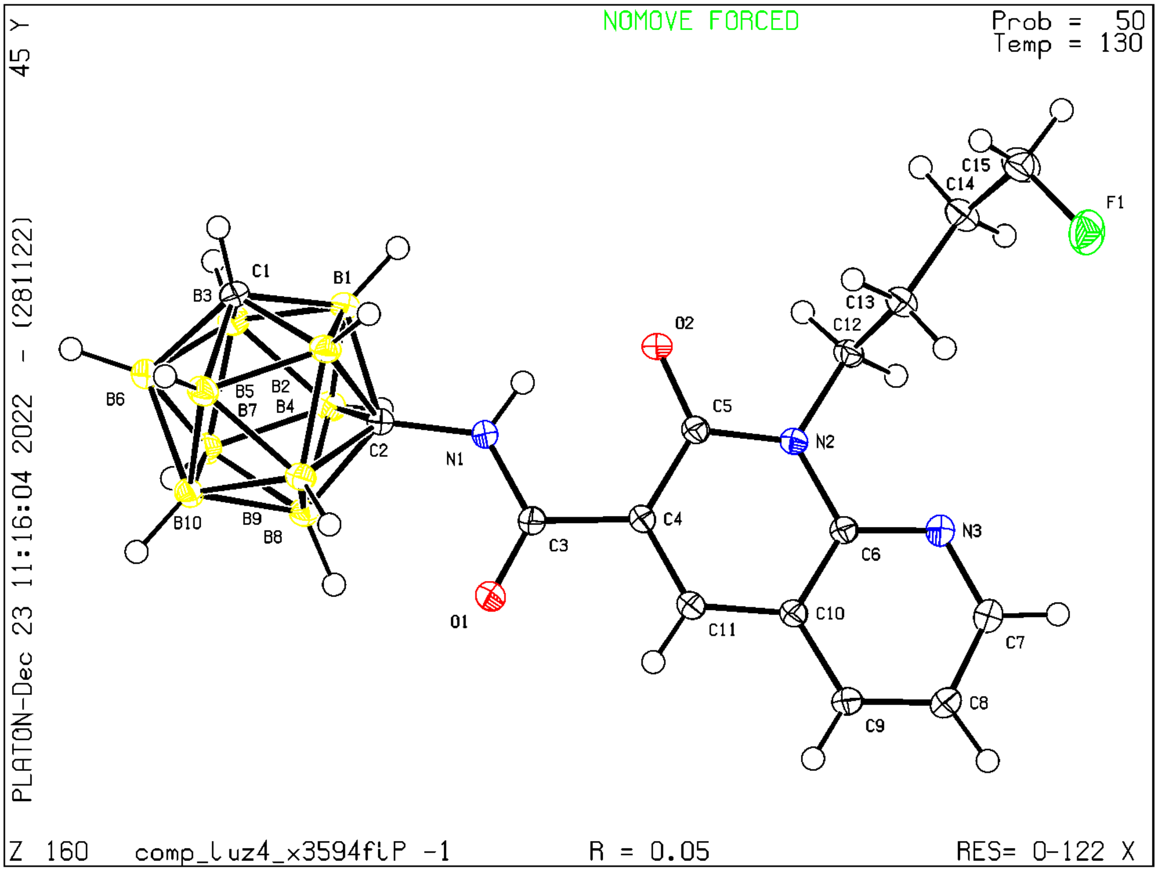

Datablock comp_luz5_x3531fin - ellipsoid plot

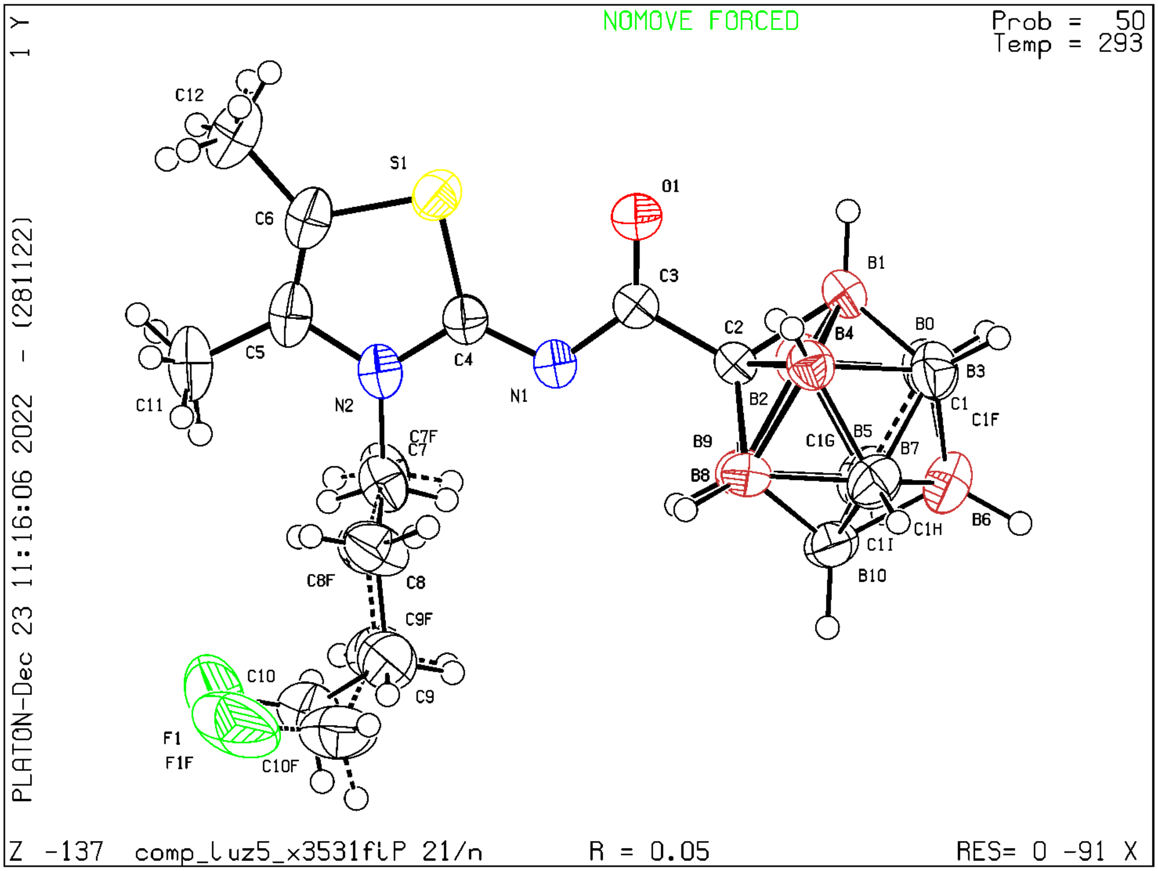

Datablock comp_luz6_x3609fin - ellipsoid plot

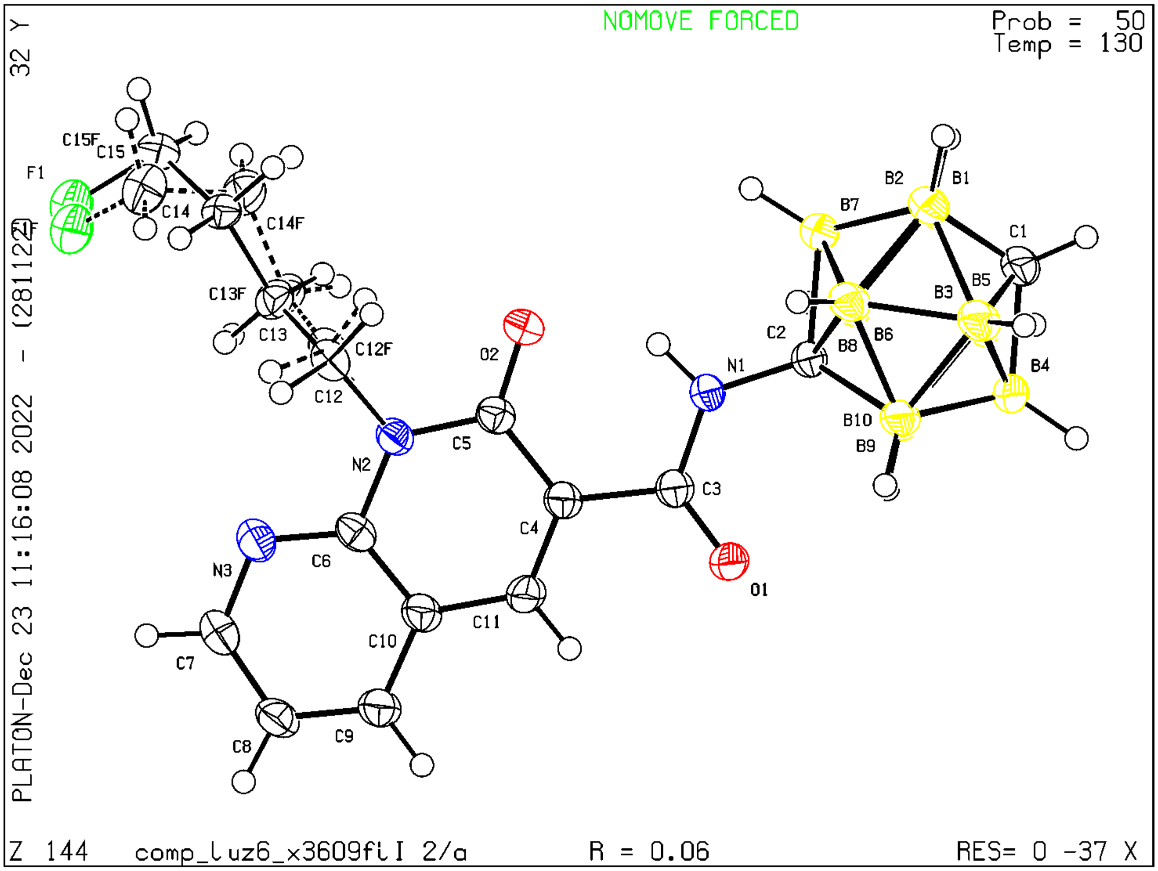

Datablock comp_luz7_x3551fin - ellipsoid plot

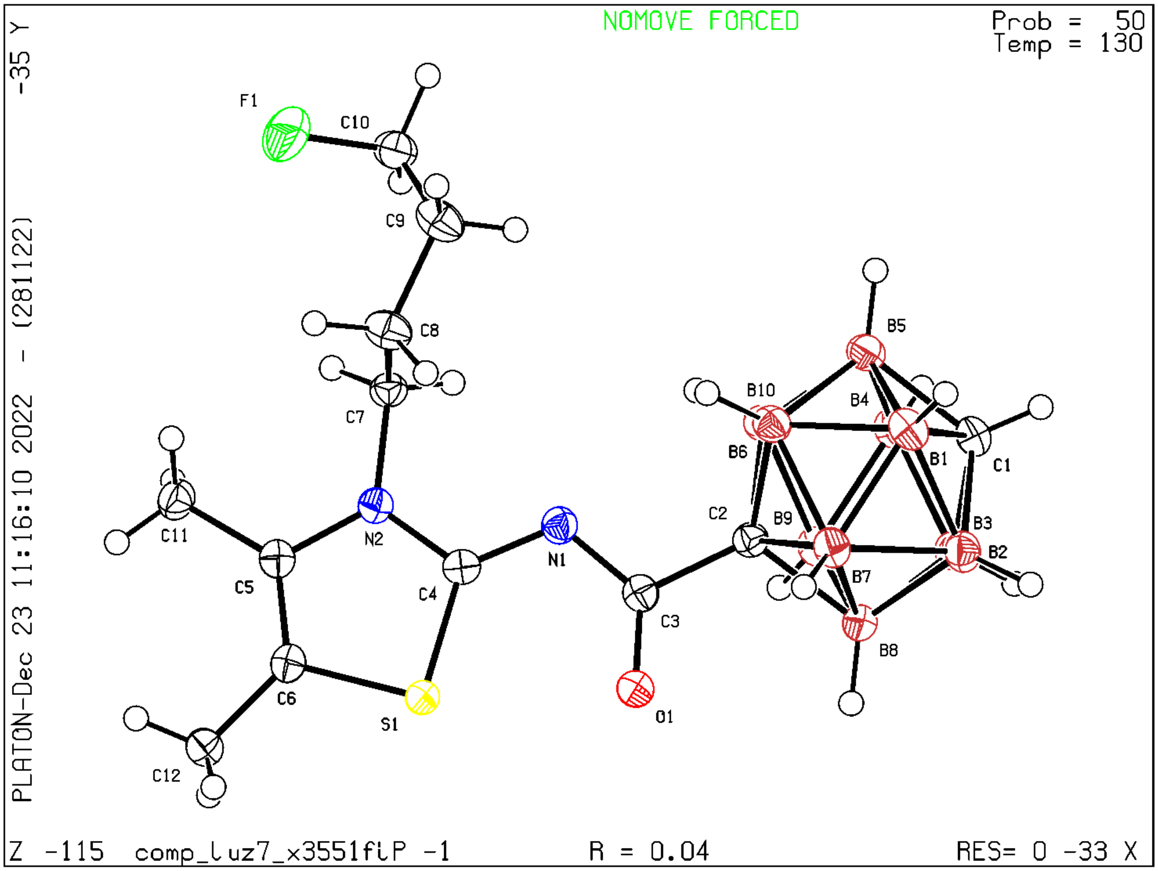

Supplement: Supplementary file 3 — jm3c00195_si_003.pdf [file jm3c00195_si_003.pdf]
