# Supplementary material for: Development of the High-Affinity Carborane-Based Cannabinoid Receptor Type 2 PET Ligand [18F]LUZ5-d8
Source: J Med Chem. 2023 Mar 21;66(7):5242–60. doi: 10.1021/acs.jmedchem.3c00195 (PMC10782483; doi:10.1021/acs.jmedchem.3c00195)
Supplement: Supplementary file 8 — jm3c00195_si_008.pdf [file jm3c00195_si_008.pdf]

Supporting Information

Development of the High-Affinity Carborane-Based Cannabinoid Receptor Type 2 PET Ligand [^{18}F]LUZ5-*d*$_8$

Lea Ueberham[1], Daniel Gündel[2], Martin Kellert[1,‡], Winnie Deuther-Conrad[2], Friedrich-Alexander Ludwig[2], Peter Lönnecke[1], Aleksandr Kazimir[1], Klaus Kopka[2,3], Peter Brust[2,4], Rareş-Petru Moldovan[2,*], Evamarie Hey-Hawkins[1,*]

[1]Universität Leipzig, Faculty of Chemistry and Mineralogy, Institute of Inorganic Chemistry, Johannisallee 29, 04103 Leipzig, Germany

[2]Helmholtz-Zentrum Dresden-Rossendorf (HZDR), Institute of Radiopharmaceutical Cancer Research, Department of Neuroradiopharmaceuticals, Research site Leipzig, 04318 Leipzig, Germany

[3]Faculty of Chemistry and Food Chemistry, School of Science, TU Dresden, 01069 Dresden, Germany

[4]The Lübeck Institute of Experimental Dermatology, University Medical Center Schleswig-Holstein, 23562 Lübeck, Germany

‡Arevipharma GmbH, Meißner Str. 35, 01445 Radebeul, Germany

*Correspondence: Evamarie Hey-Hawkins (hey@uni-leipzig.de, Tel.: +49 341 97 36151)

Rareş-Petru Moldovan (r.moldovan@hzdr.de, Tel.: +49 3412341794634)

Content:

1 NMR SPECTRA OF COMPOUNDS 9, 10, 11, 12, 15 (PROCEDURE 1, 2), 16 (LUZ5), SP1, 17, 18, 19 .... S3

2 HR-ESI MASS SPECTRA OF COMPOUNDS 9, 10, 11, 15 (PROCEDURE 1, 2), LUZ5, SP1, 17, 19 .... S29

3 HPLC PURITY DETERMINATION OF COMPOUNDS 9, 10, 11, 12, 15 (PROCEDURE 2), LUZ5, 17 .... S34

4 STABILITY DATA OF COMPOUNDS 9, 10, 11, 15 (PROCEDURE 2), LUZ5, 17 ... S39

4.1 Measurements in aq. DMSO-*d*$_6$ S39

4.2 Measurements of LUZ5 in aq. DMSO-*d*$_6$, with increased H_2O/ D_2O amount S46

5 X-RAY CRYSTALLOGRAPHY DATA OF COMPOUNDS 9, 10, 11, 15 (PROCEDURE 2), LUZ5, 17 AND MOLECULAR STRUCTURES OF 11, 17 .... S47

6 *IN VITRO* BINDING ASSAY: INHIBITION CURVES OF COMPOUNDS 9, 10, 11, 15 (PROCEDURE 2), LUZ5, 17 .... S51

Supporting Information

6.1 Inhibition curves of binding affinity assays of compounds 9, 10, 11, 15 (Procedure 2), LUZ5, 17 to CB_2R S51

6.2 Inhibition curves of binding affinity assays of compounds 9, 10, 11, 15 (Procedure 2), LUZ5, 17 to CB_1R S53

7 DOCKING DATA OF COMPOUNDS 9, 15 (PROCEDURE 2), LUZ5 .... S54

8 *IN VITRO* METABOLISM STUDIES: LC-MS EXEMPLIFIED SPECTRUM OF LUZ5 .... S56

9 UV PROFILE OF ISOLATED [^{18}F]LUZ5-d_8 .... S56

10 ASSESSMENT OF THE BIODISTRIBUTION OF [^{18}F]LUZ5-d_8 IN RODENT MODELS S57

11 CHEMICAL STRUCTURES OF CB_2R AGONISTS AND CB_1R ANTAGONIST/ INVERSE AGONIST USED IN BIOLOGICAL EXPERIMENTS S58

12 REFERENCES S59

Supporting Information

1 NMR Spectra of Compounds 9, 10, 11, 12, 15 (Procedure 1, 2), 16 (LUZ5), SP1, 17, 18, 19

Figure S1. ^{1}H NMR spectrum of compound **9** in $CDCl_3$.

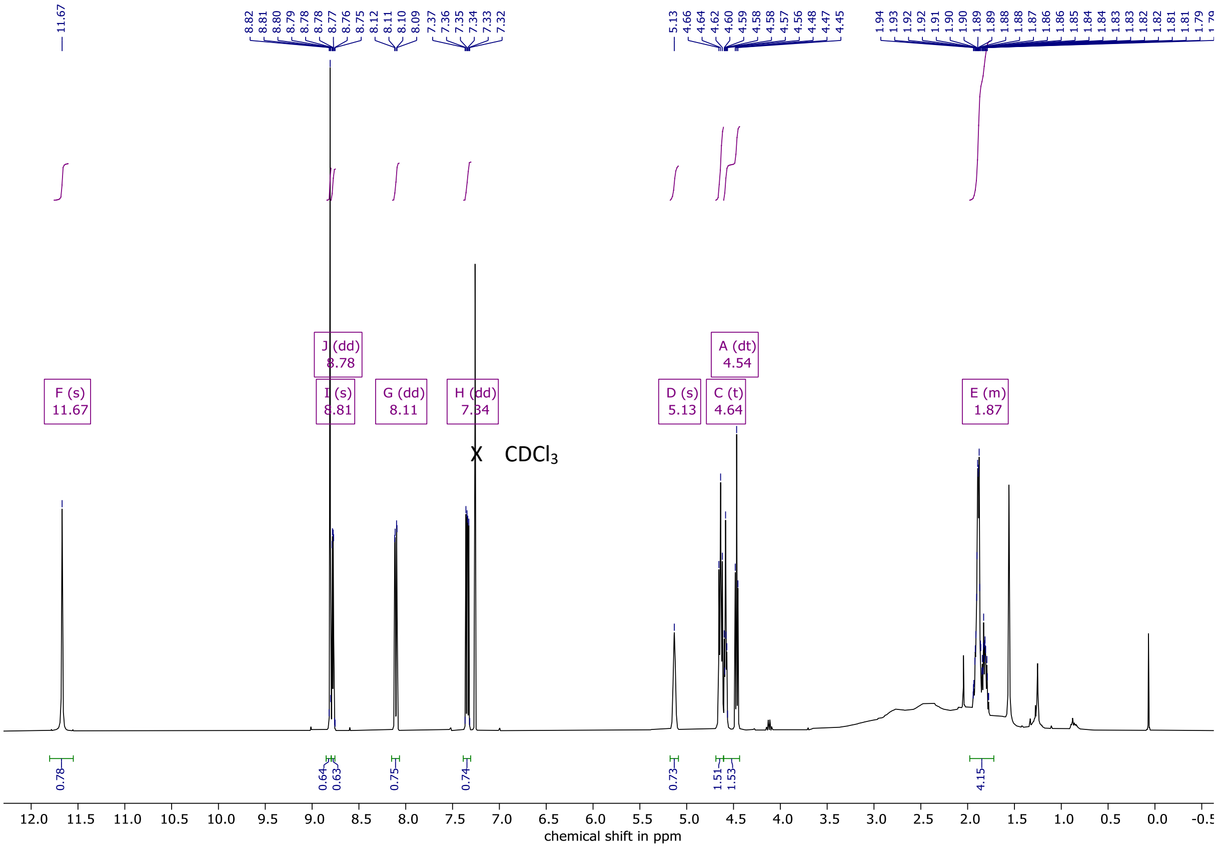

Supporting Information

Figure S2. $^{11}B\{^1H\}$ NMR spectrum of compound **9** in $CDCl_3$.

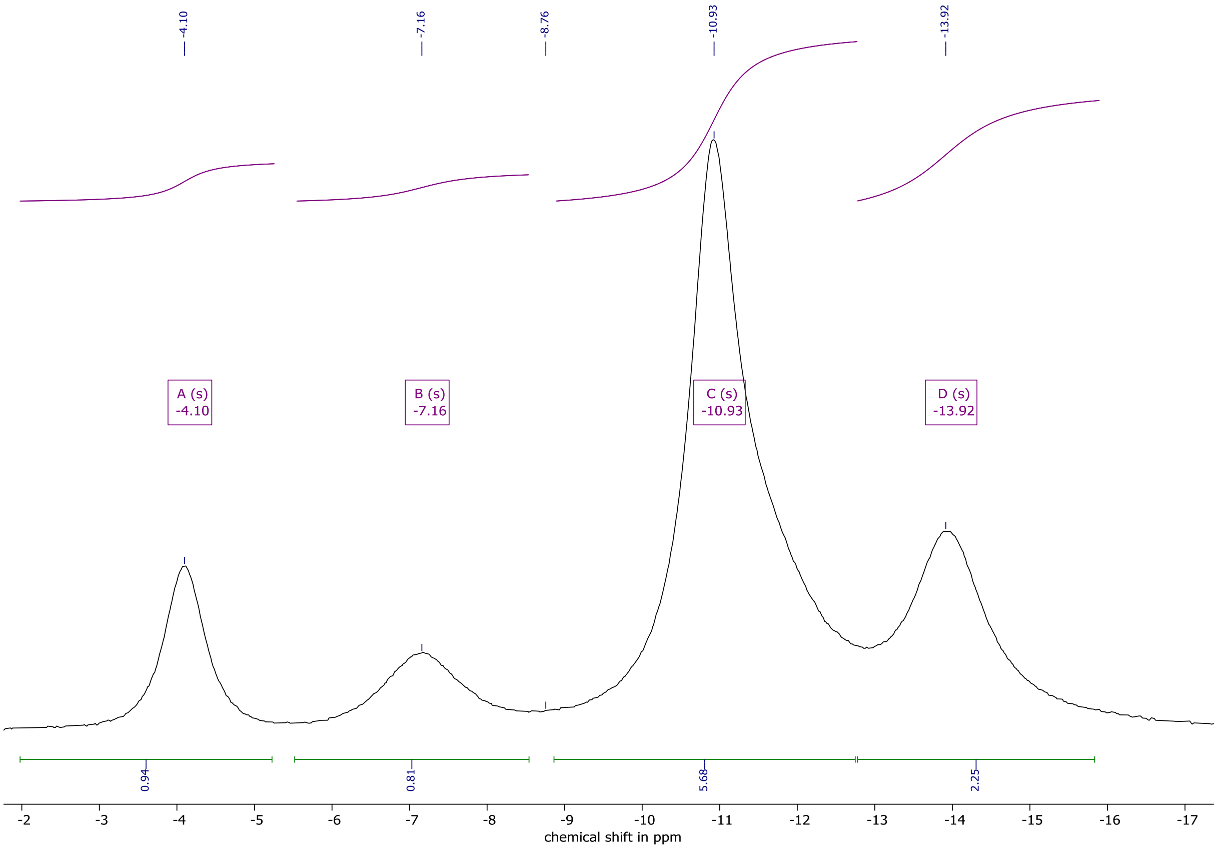

Figure S3. $^{13}C\{^1H\}$ NMR spectrum of compound **9** in $CDCl_3$.

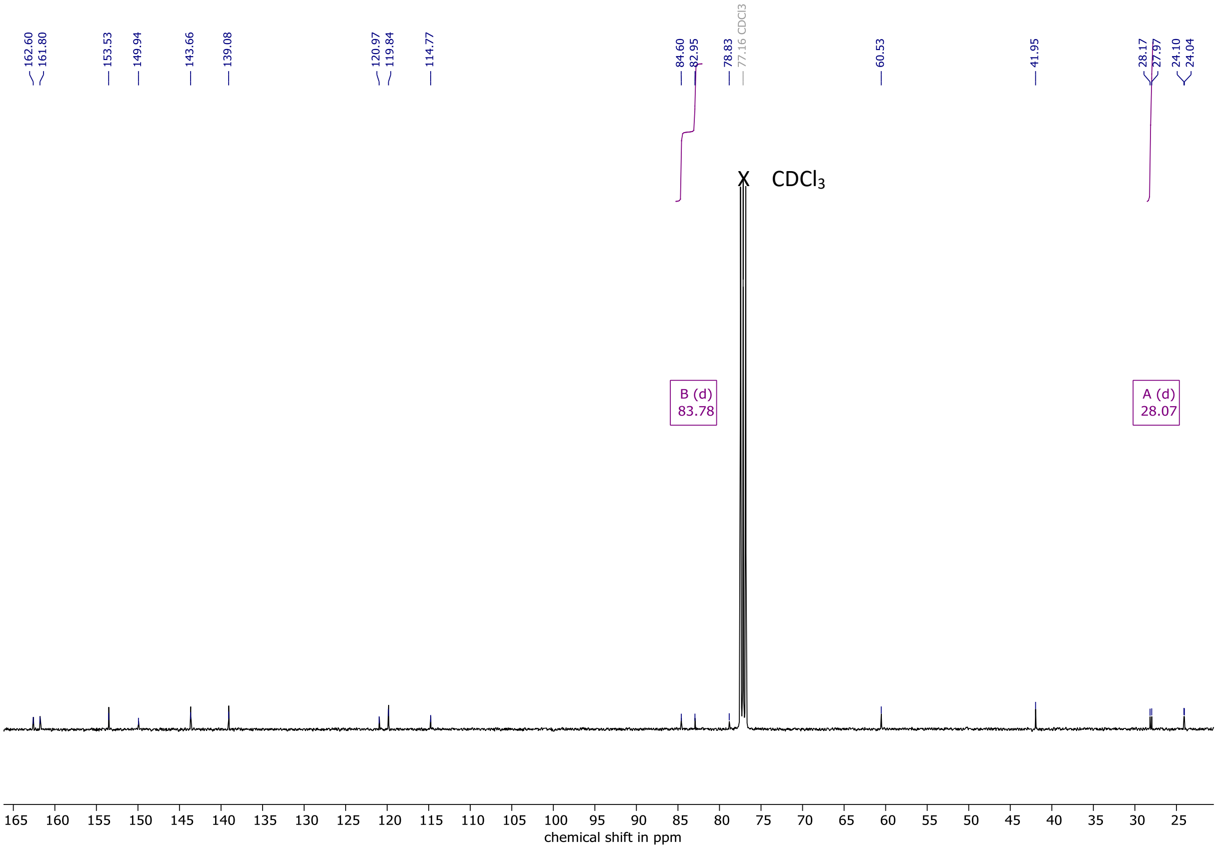

Figure S4. COSY(^1H-^1H) NMR spectrum of compound **9** in $CDCl_3$.

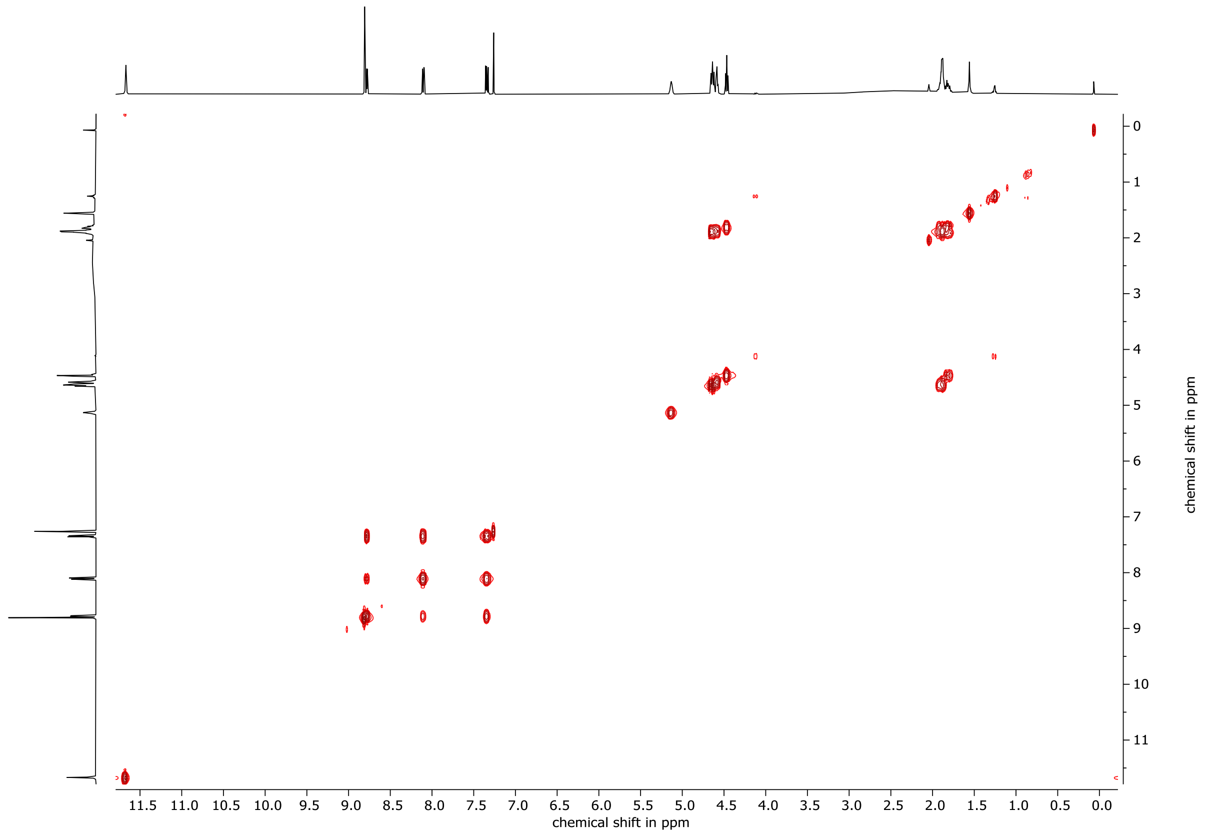

Figure S5. HSQC(^1H-^{13}C} NMR spectrum of compound **9** in $CDCl_3$.

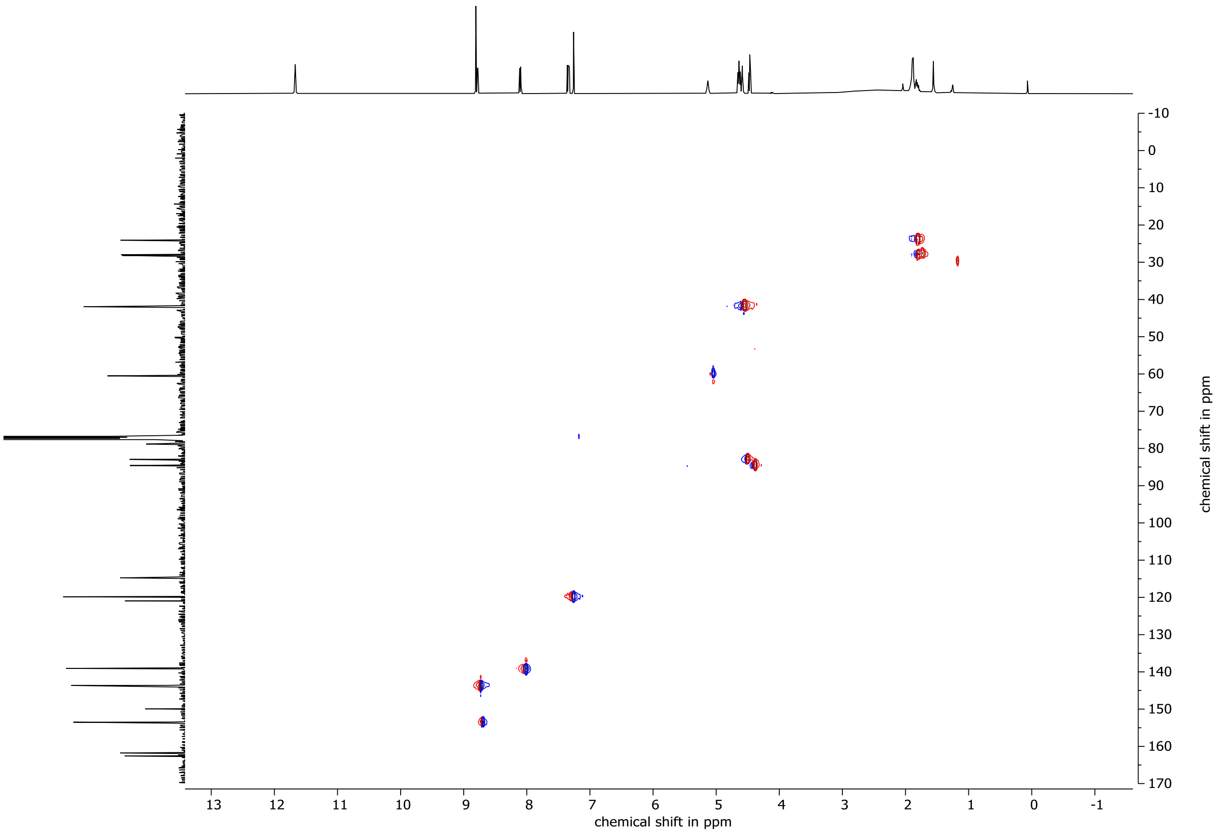

Figure S6. HMBC(^{1}H-^{13}C) NMR spectrum of compound **9** in $CDCl_3$.

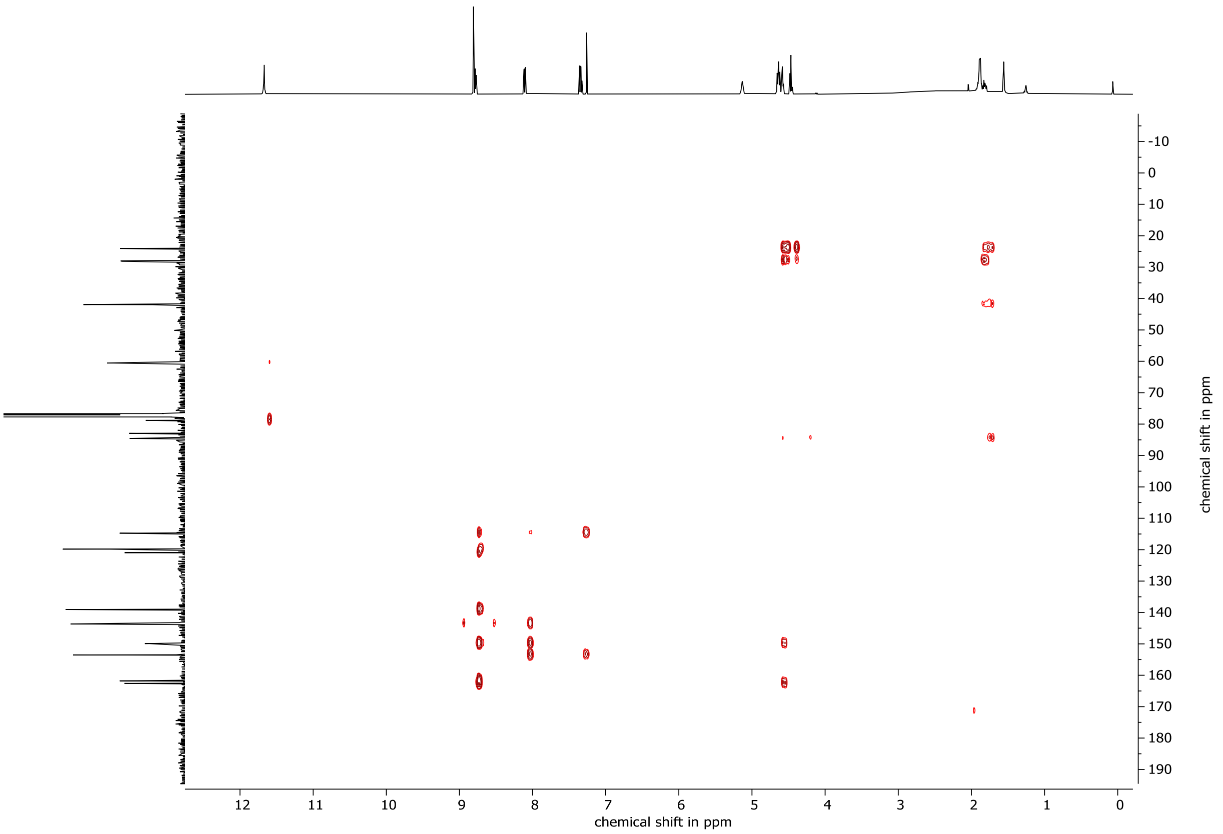

Figure S7. ^{1}H NMR spectrum of compound **10** in $CDCl_3$.

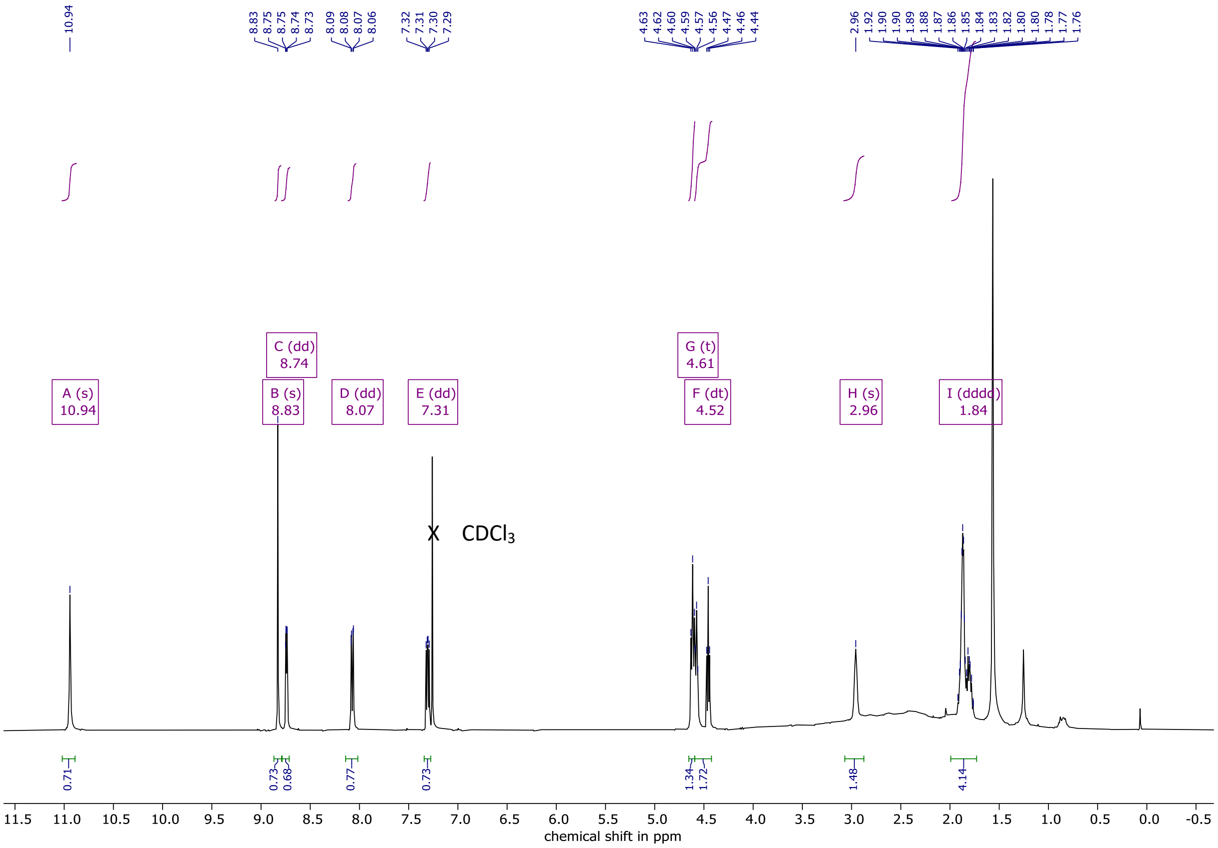

Supporting Information

Figure S8. $^{11}B\{^{1}H\}$ NMR spectrum of compound **10** in $CDCl_3$.

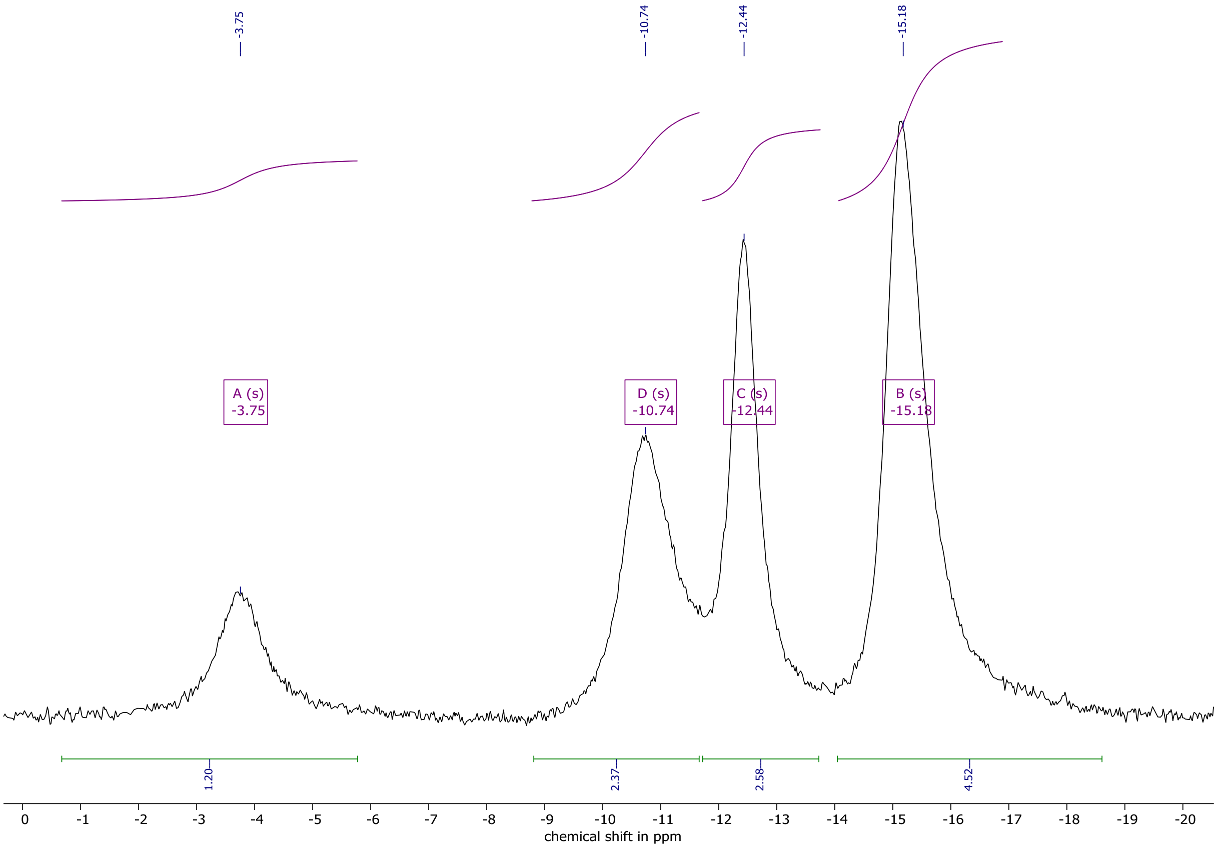

Figure S9. $^{13}C\{^{1}H\}$ NMR spectrum of compound **10** in $CDCl_3$.

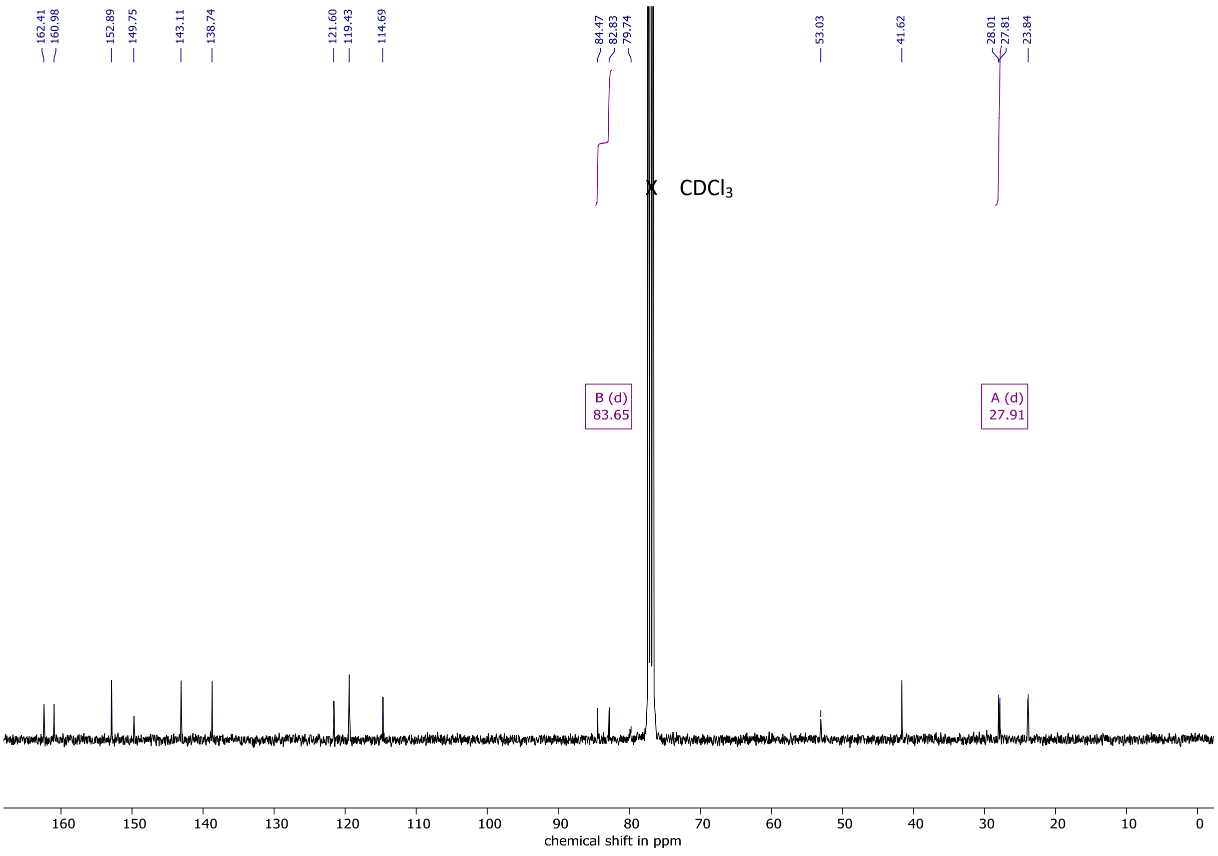

Supporting Information

Figure S10. COSY(^{1}H-^{1}H) NMR spectrum of compound **10** in $CDCl_3$.

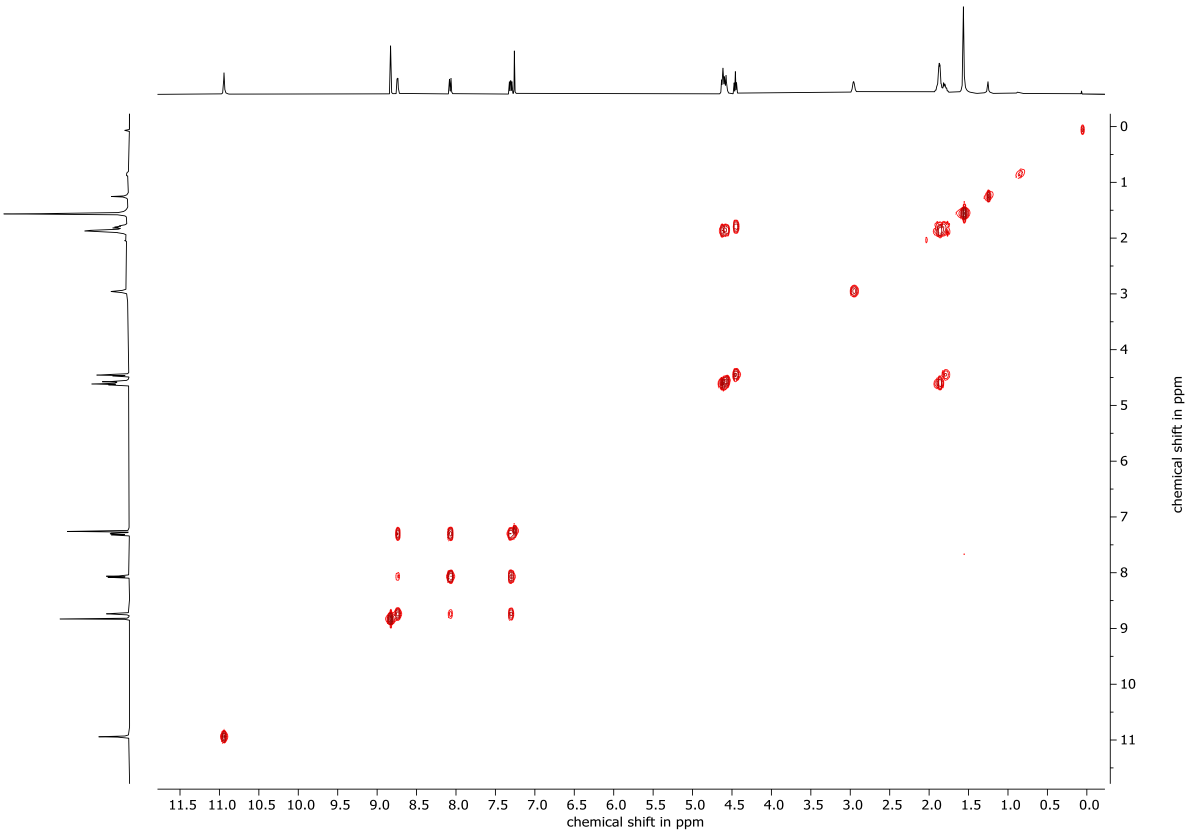

Figure S11. HSQC(^{1}H-^{13}C} NMR spectrum of compound **10** in $CDCl_3$.

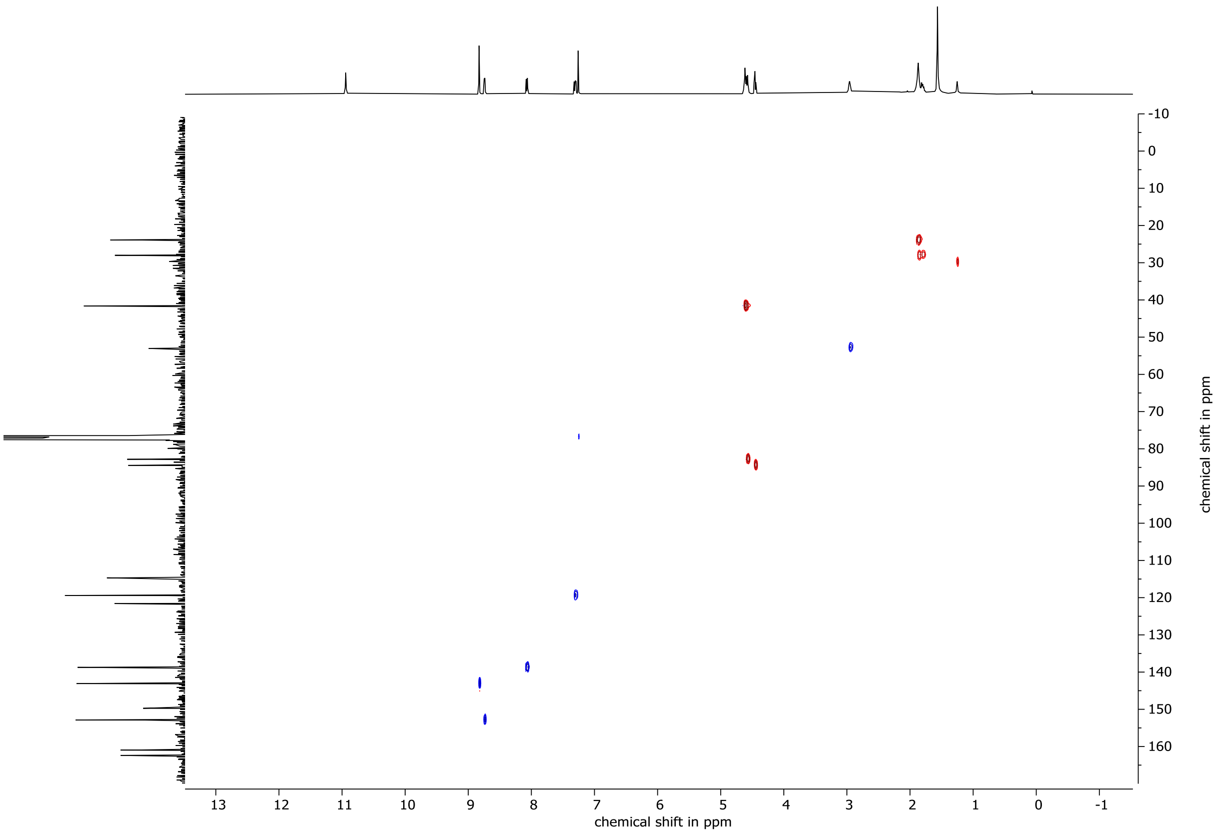

Supporting Information

Figure S12. HMBC(^1H-^{13}C) NMR spectrum of compound **10** in $CDCl_3$.

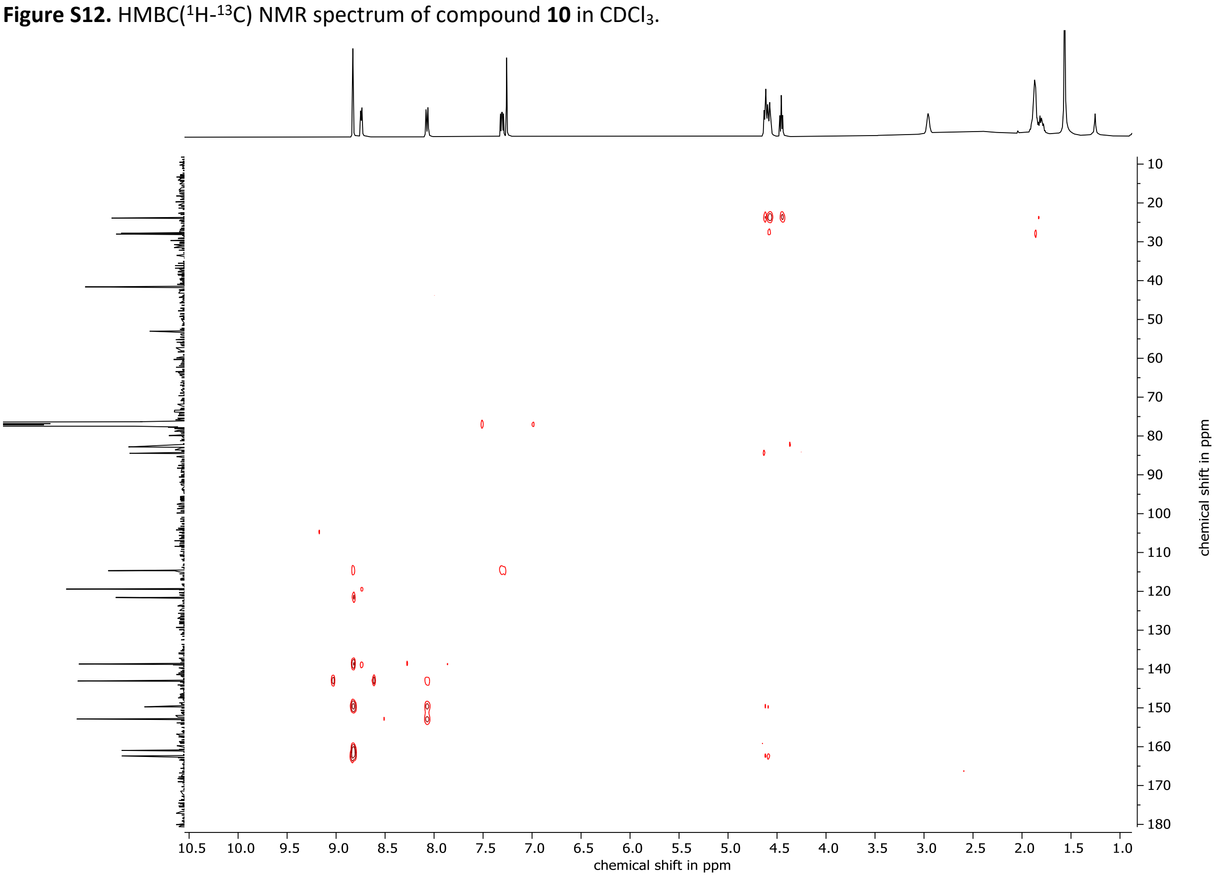

Figure S13. ^1H NMR spectrum of compound **11** in $CDCl_3$.

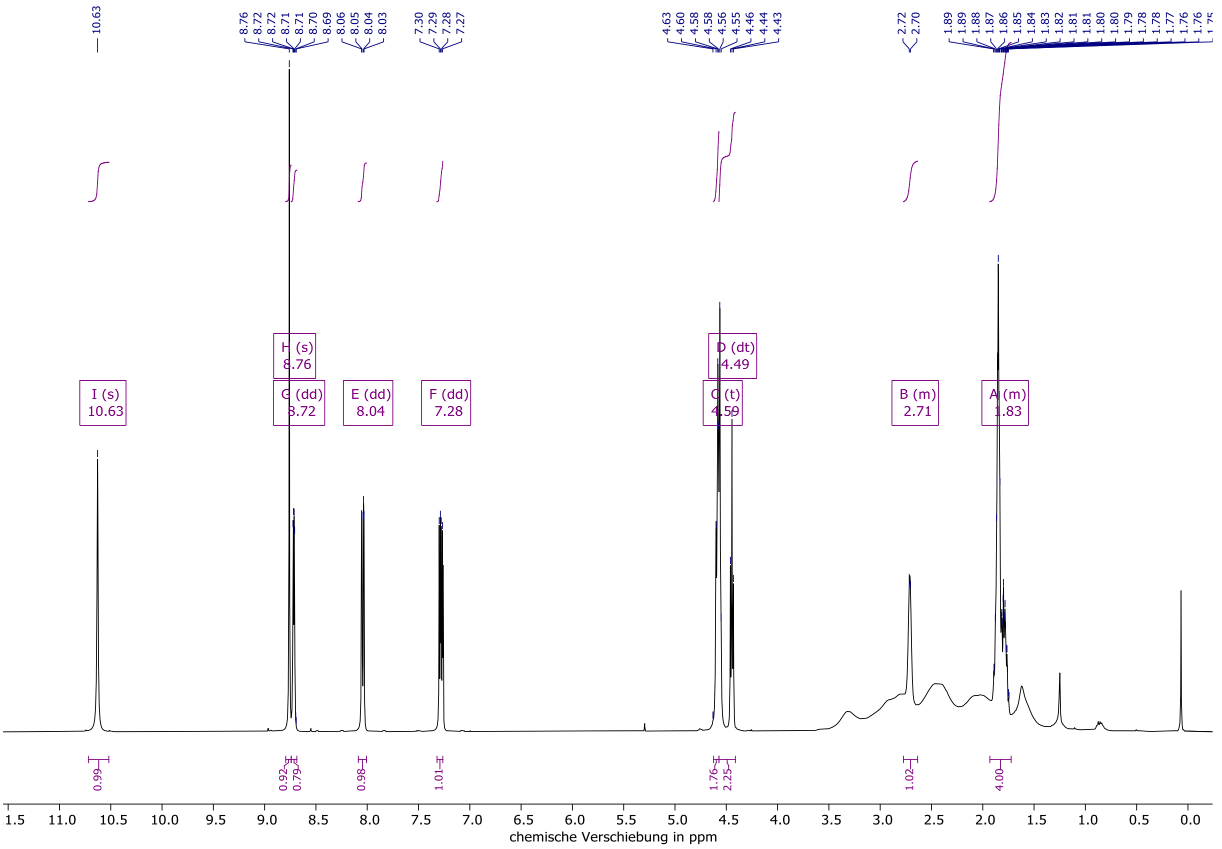

Figure S14. $^{11}B\{^1H\}$ NMR spectrum of compound **11** in $CDCl_3$.

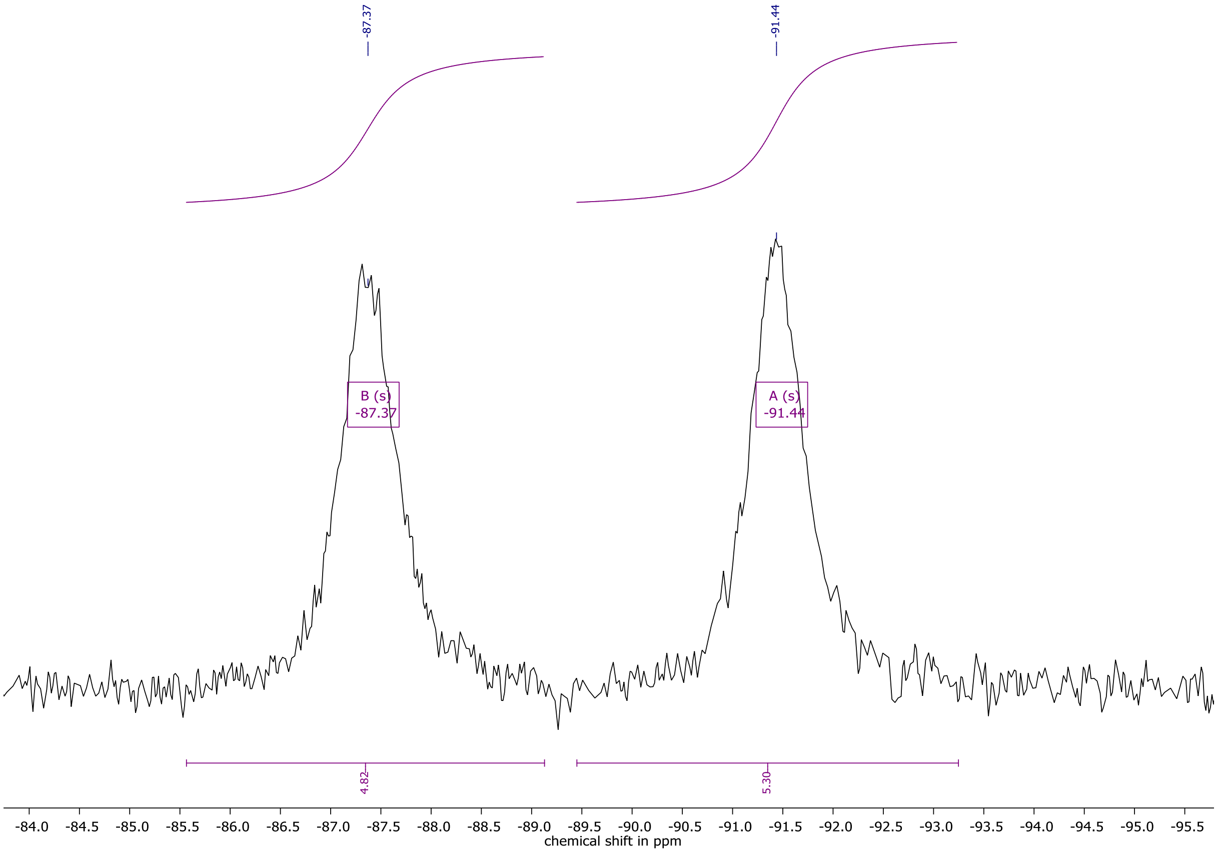

Figure S15. $^{13}C\{^1H\}$ NMR spectrum of compound **11** in $CDCl_3$.

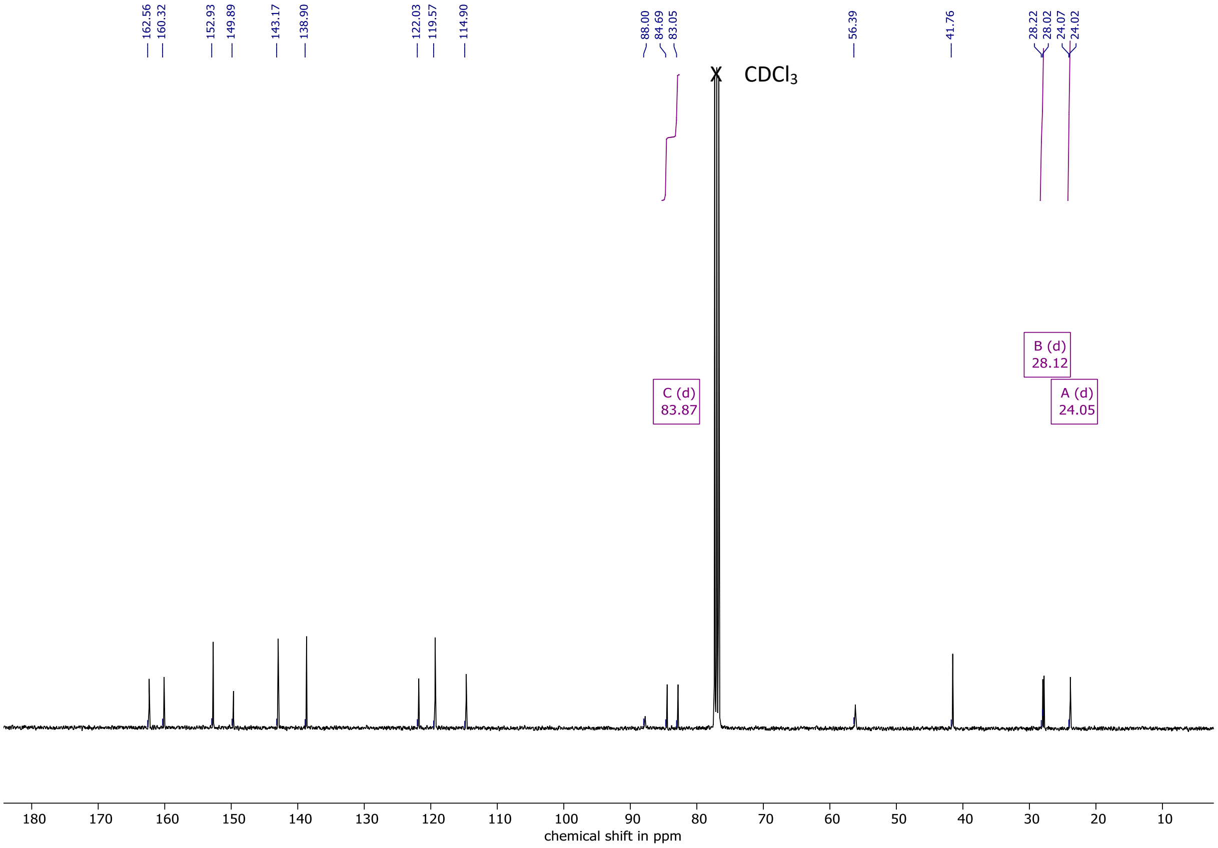

Supporting Information

Figure S16. COSY(^1H-^1H) NMR spectrum of compound **11** in $CDCl_3$.

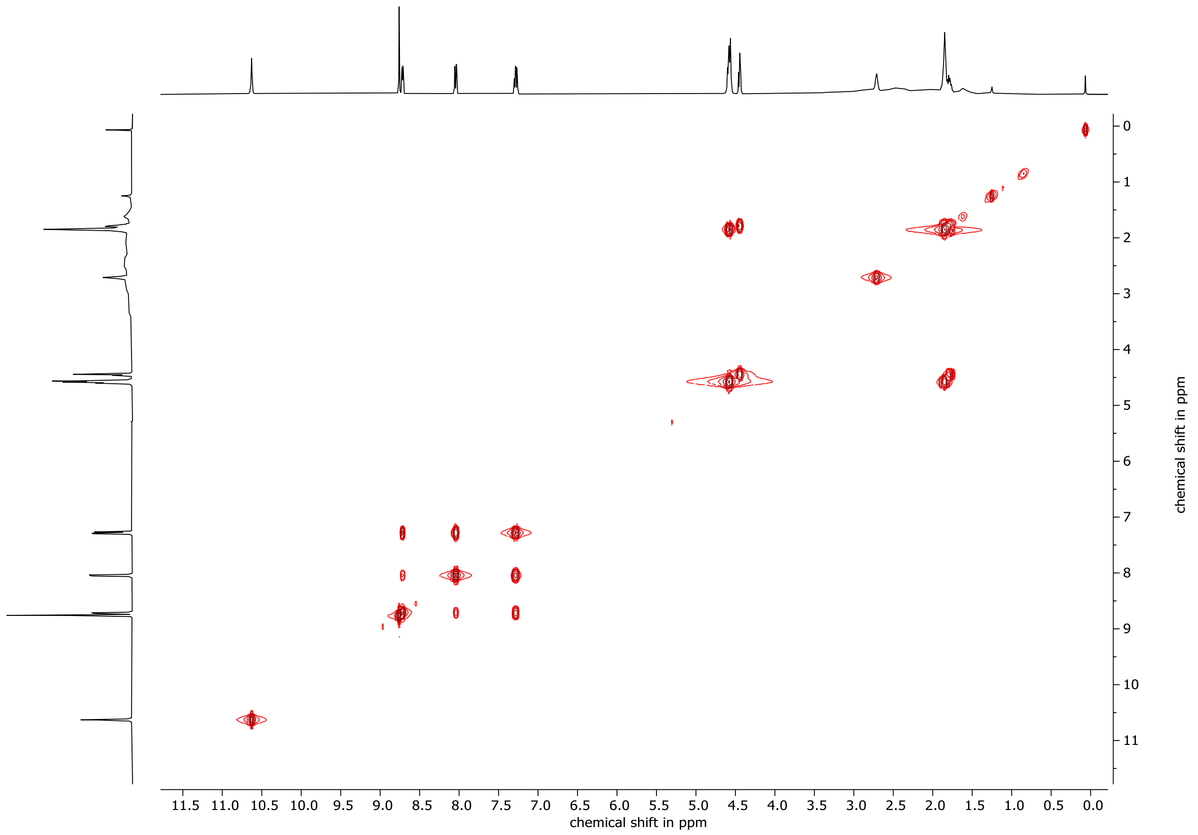

Figure S17. HSQC{^1H-^{13}C} NMR spectrum of compound **11** in $CDCl_3$.

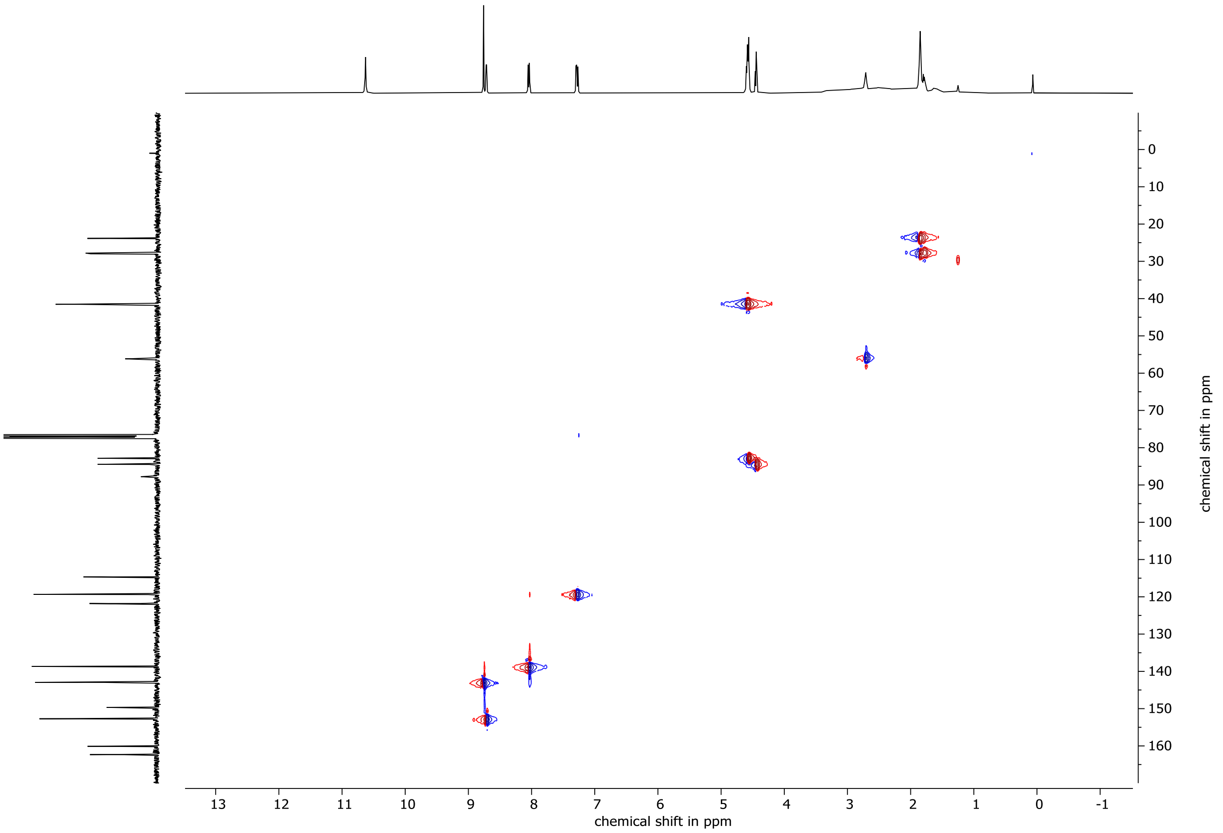

Supporting Information

Figure S18. HMBC(^{1}H-^{13}C) NMR spectrum of compound **11** in $CDCl_3$.

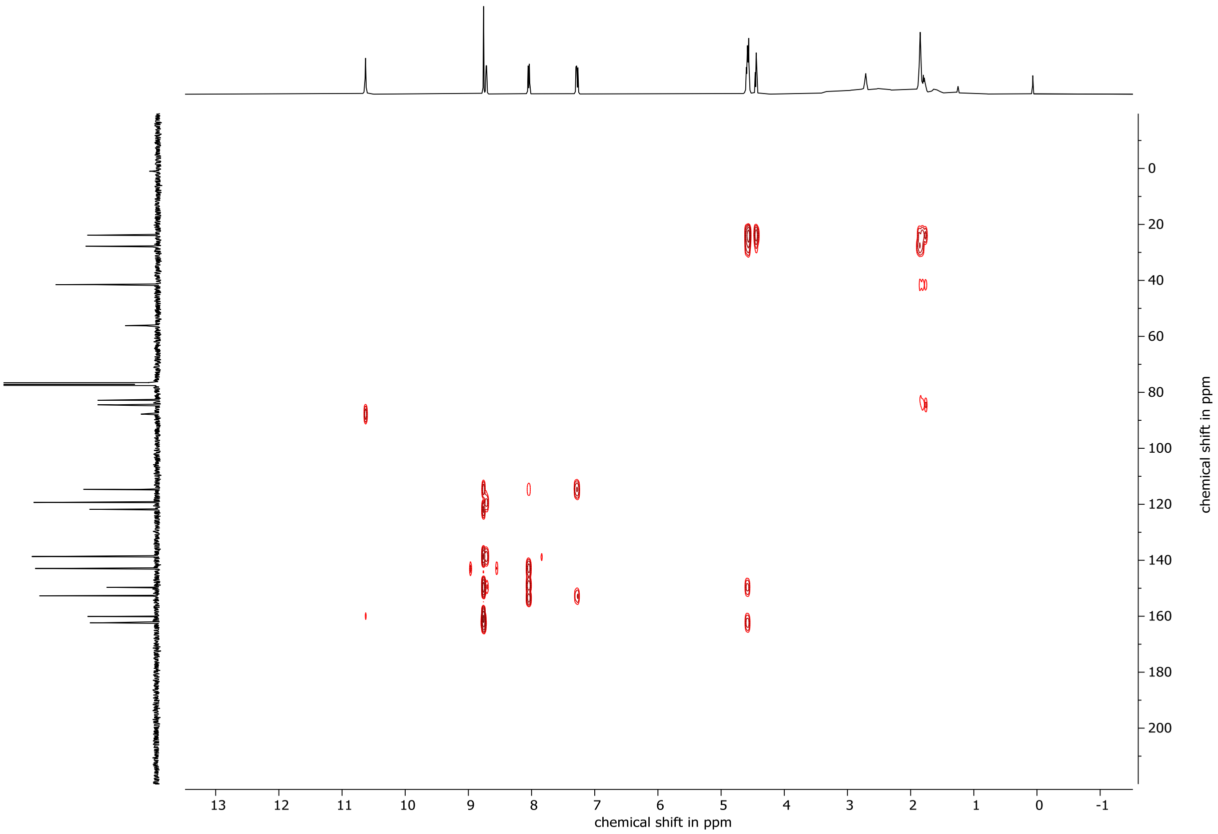

Figure S19. ^{1}H NMR spectrum of compound **12** in acetone-d_6 before HPLC-purification.

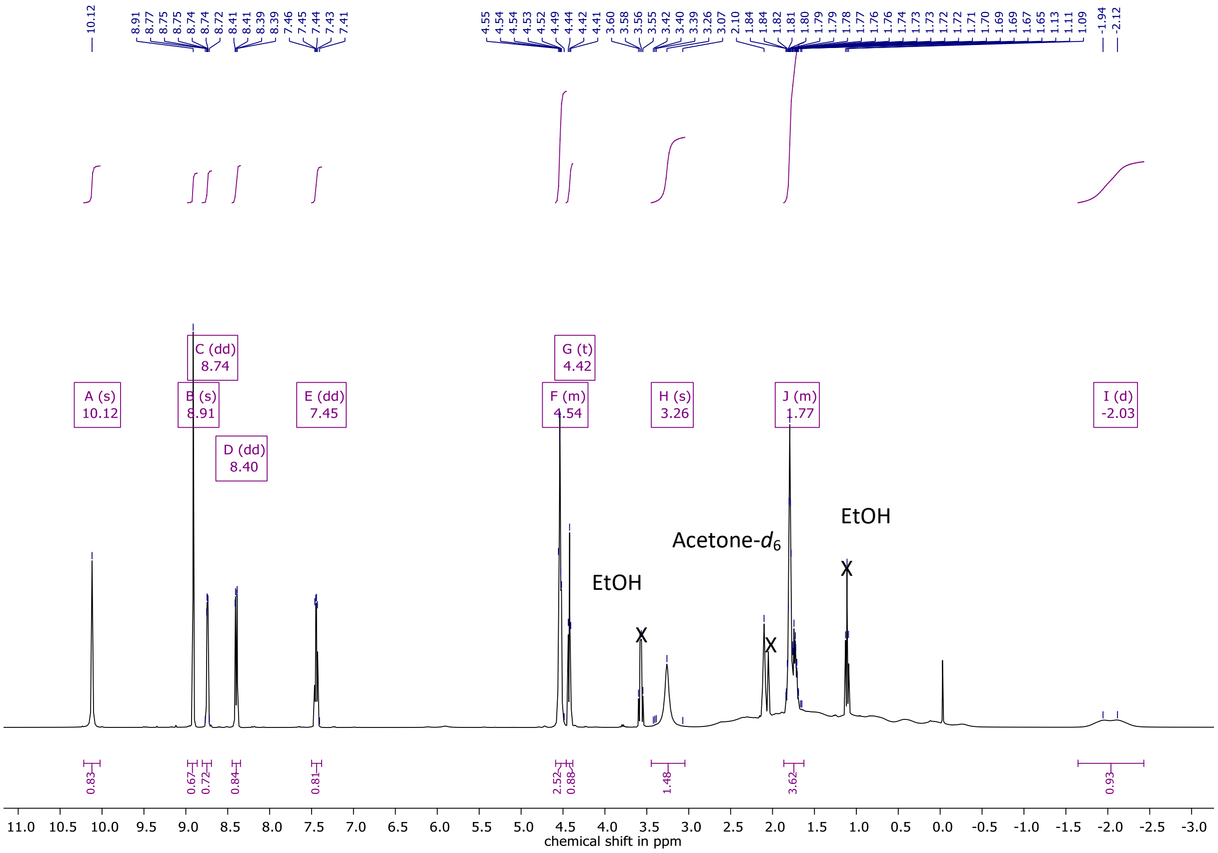

Figure S20. $^{11}B\{^{1}H\}$ NMR spectrum of compound **12** in acetone-d_6 before HPLC-purification.

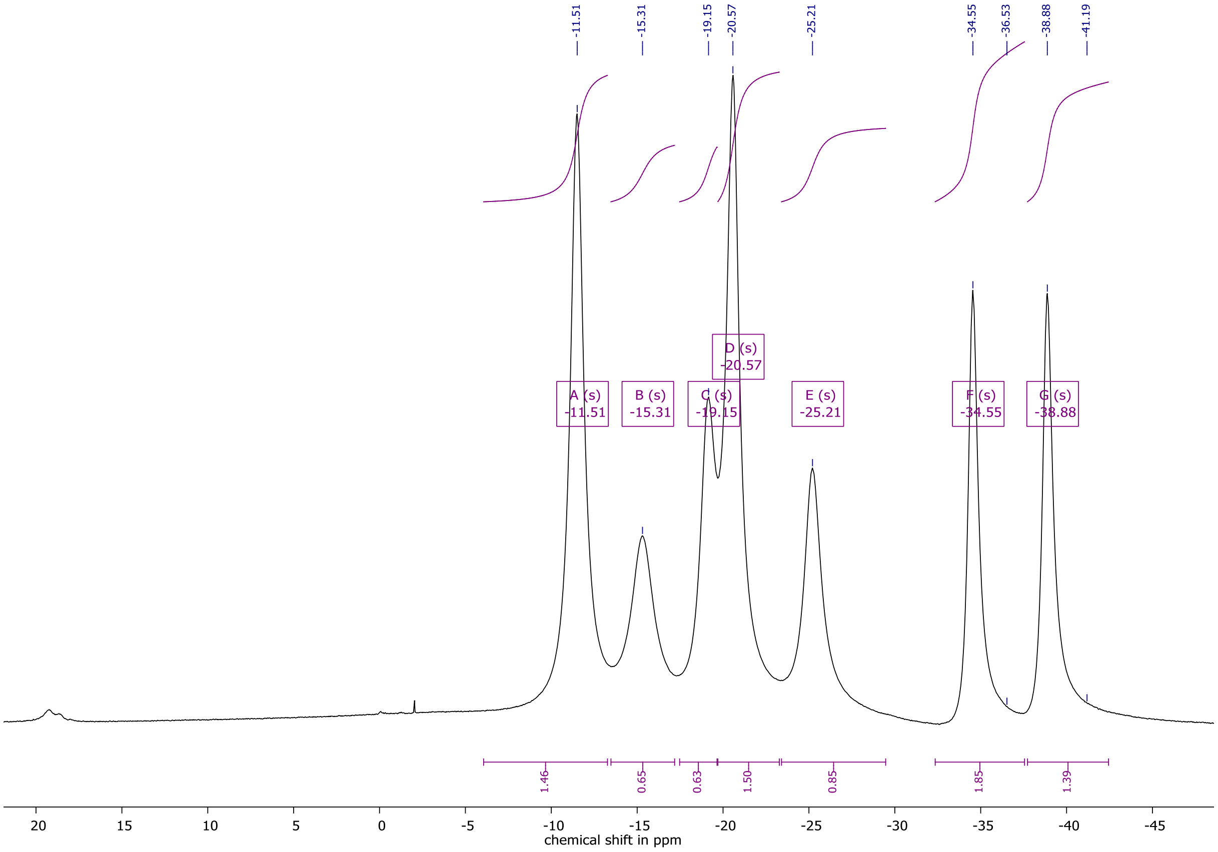

Figure S21. $^{13}C\{^{1}H\}$ NMR spectrum of compound **12** in acetone-d_6 before HPLC-purification.

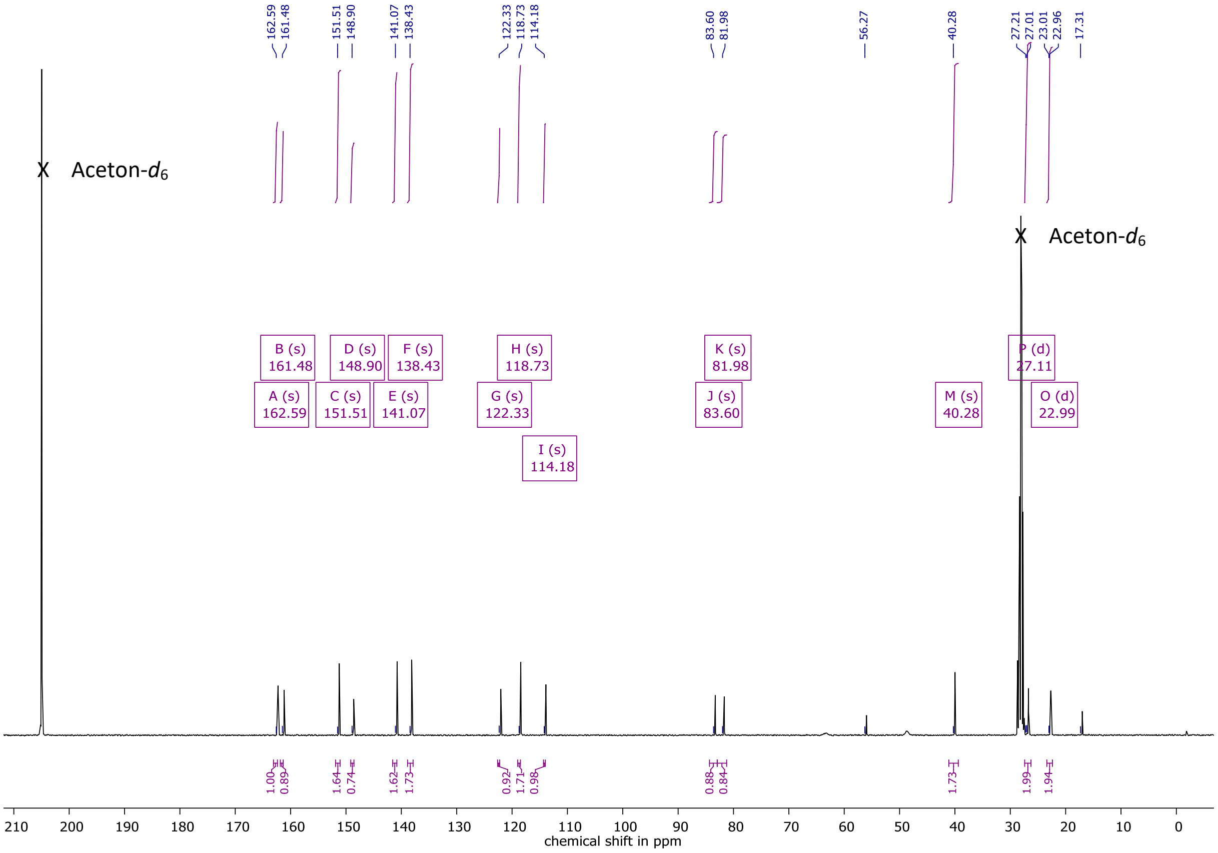

Figure S22. COSY(^{1}H-^{1}H) NMR spectrum of compound **12** in acetone-d_6 before HPLC-purification.

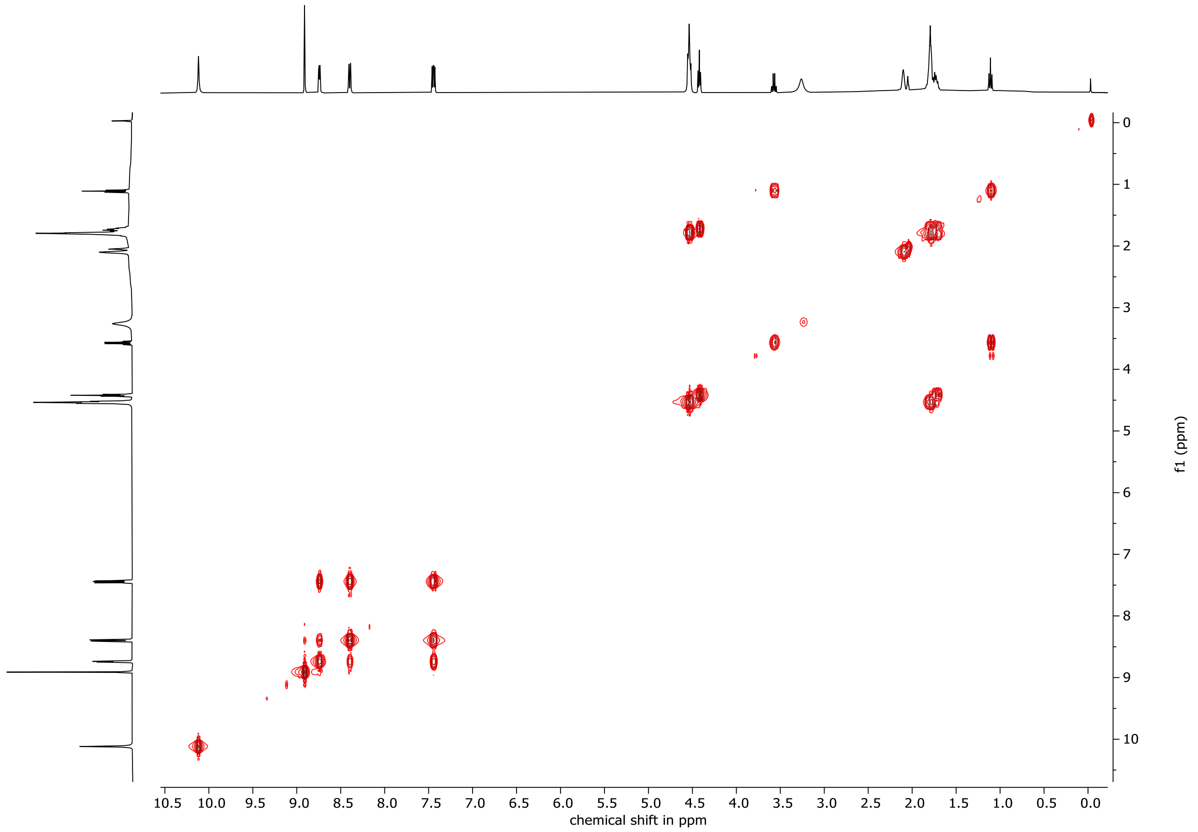

Figure S23. HSQC(^{1}H-^{13}C} NMR spectrum of compound **12** in acetone-d_6 before HPLC-purification.

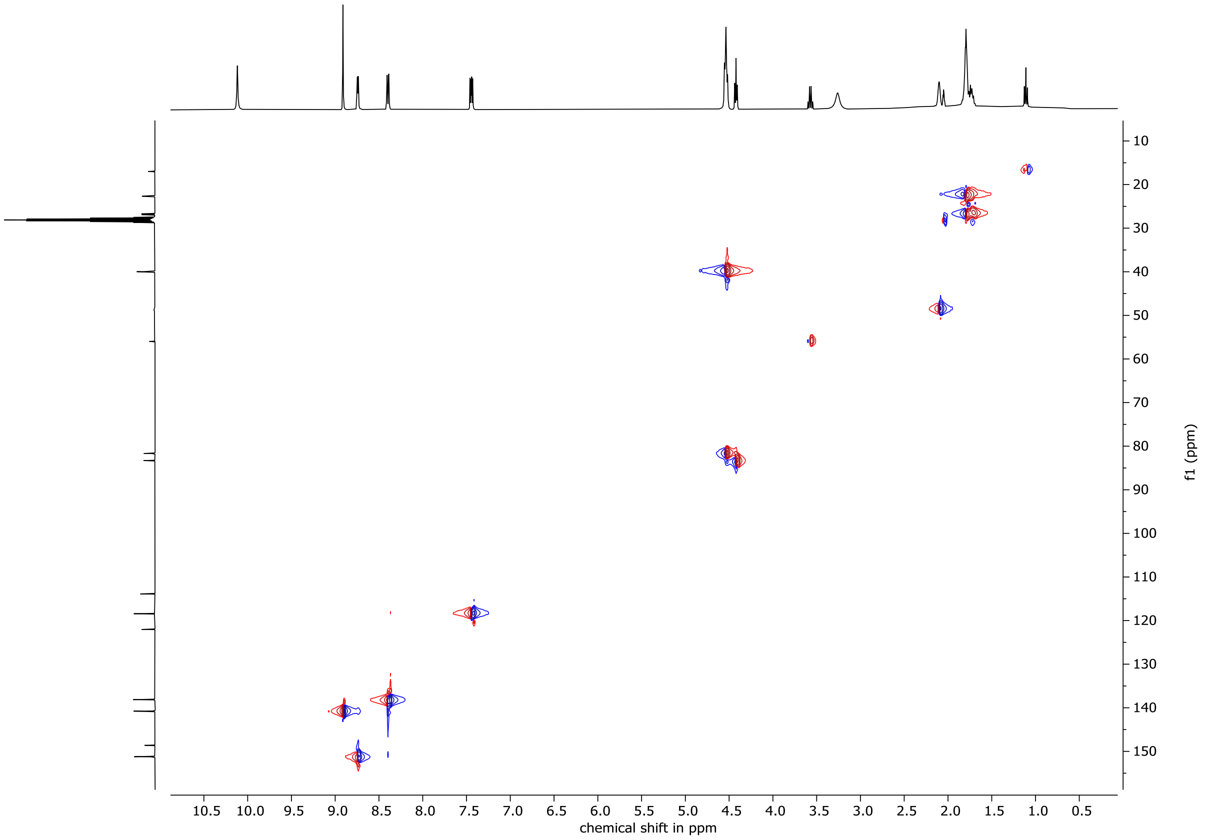

Figure S24. ^{1}H NMR spectrum of compound **15 (Procedure 1)** in $CDCl_3$.

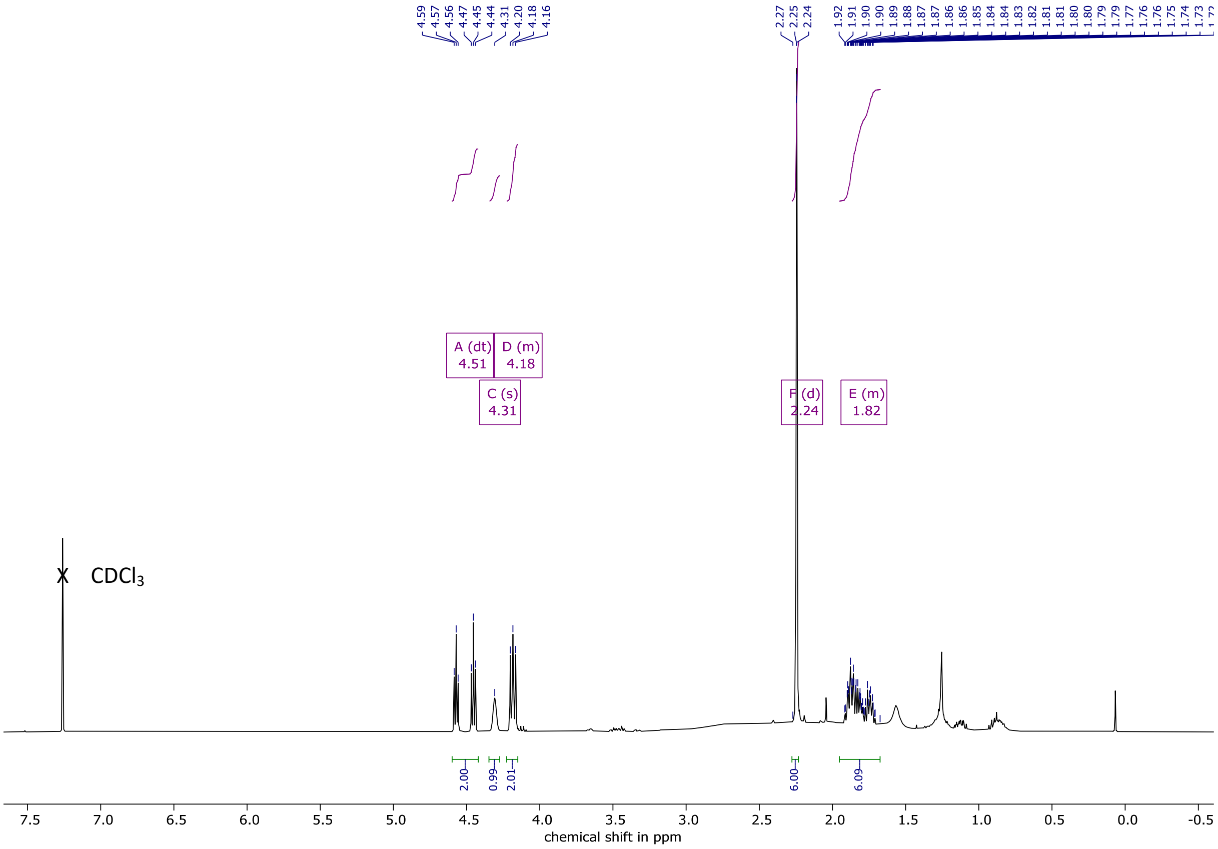

Figure S25. $^{11}B\{^{1}H\}$ NMR spectrum of compound **15 (Procedure 1)** in $CDCl_3$.

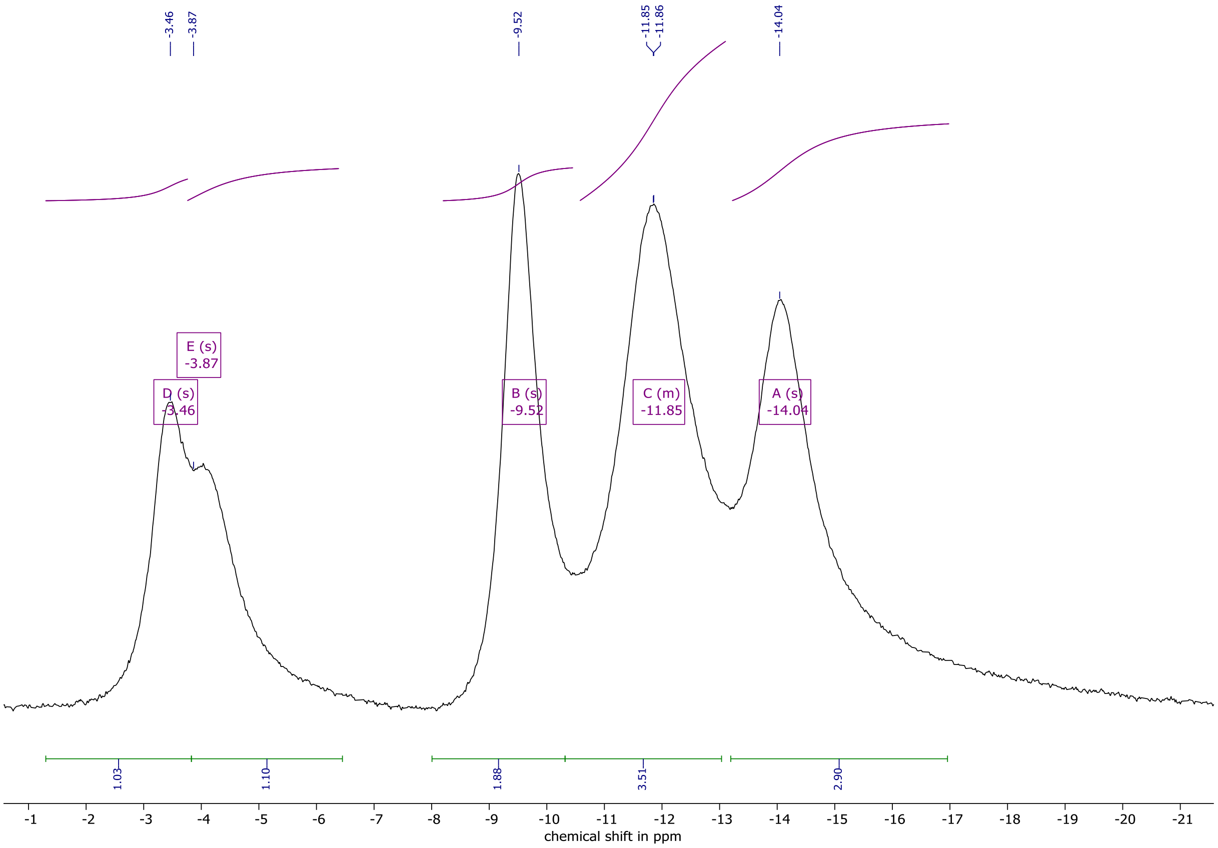

Figure S26. $^{13}C\{^1H\}$ NMR spectrum of compound **15 (Procedure 1)** in $CDCl_3$.

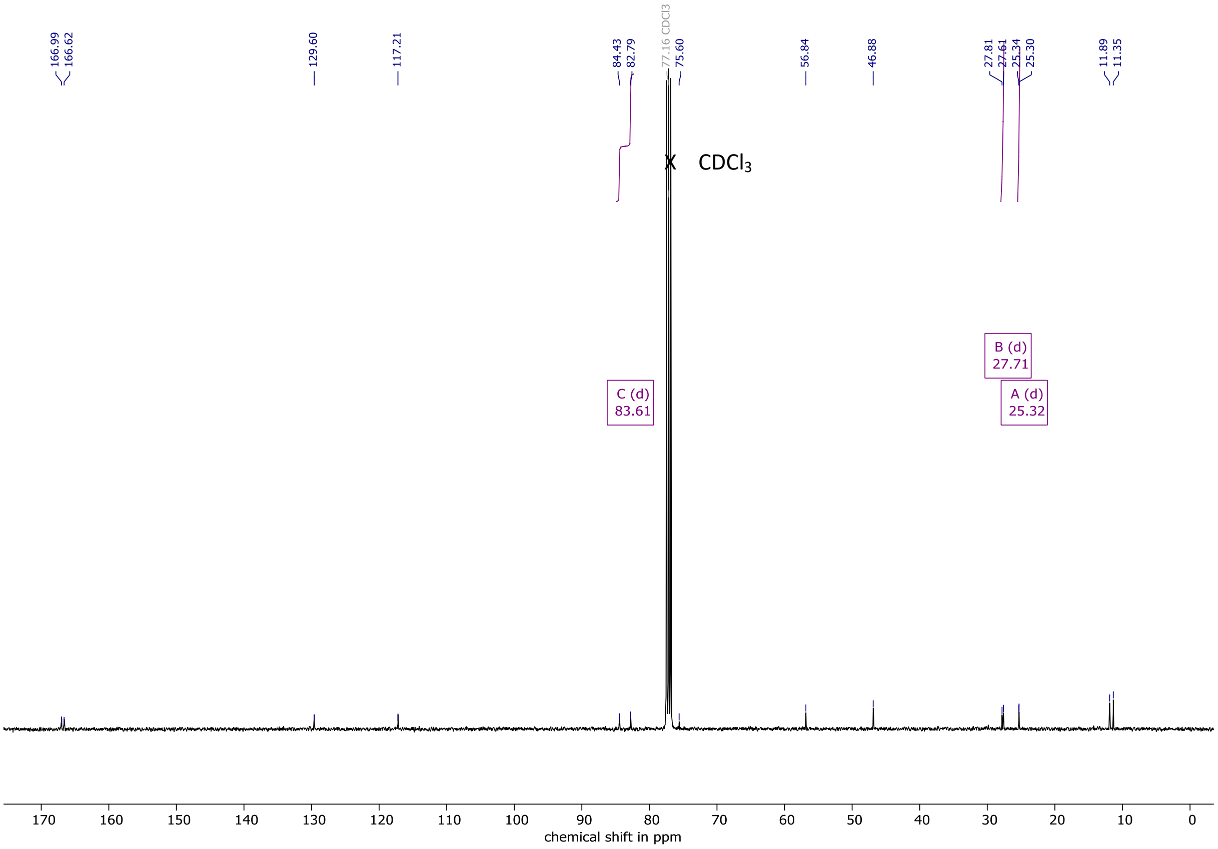

Figure S27. COSY(^1H-^1H) NMR spectrum of compound **15 (Procedure 1)** in $CDCl_3$.

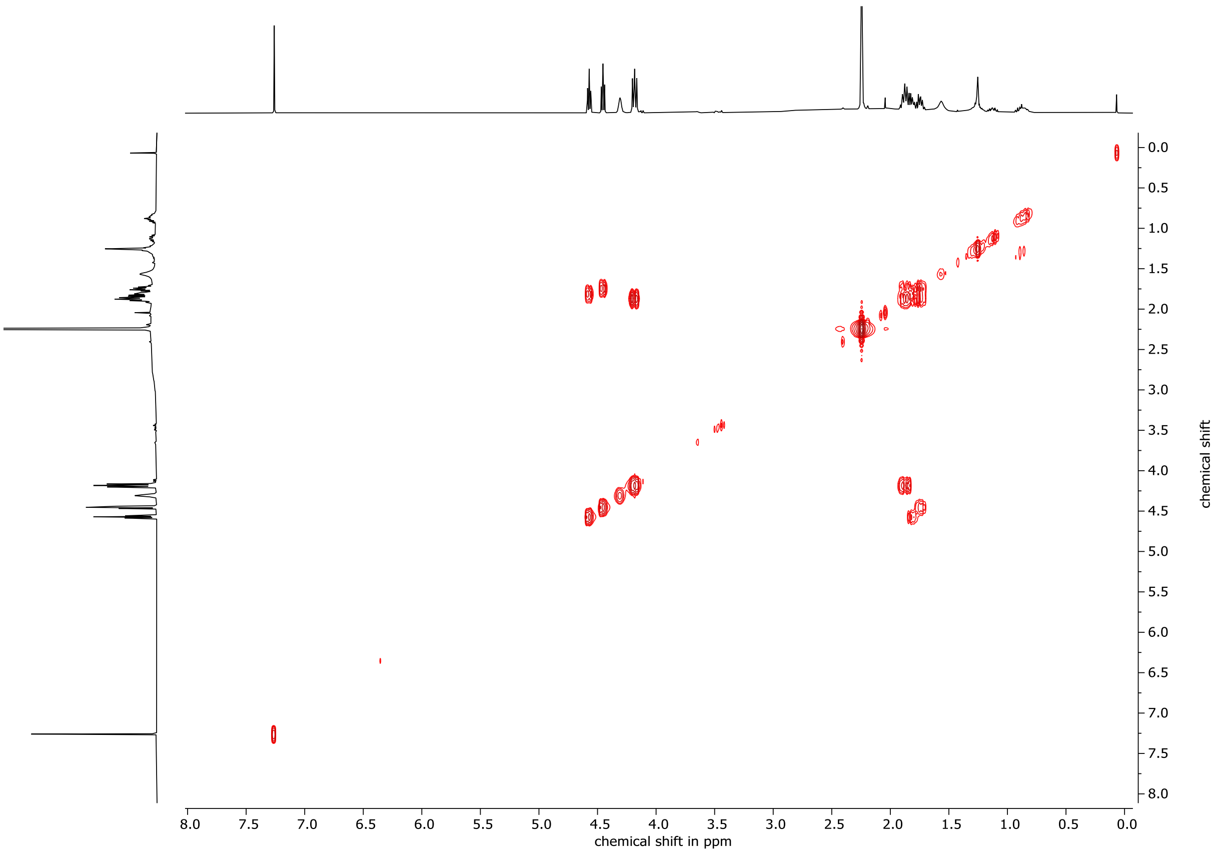

Figure S28. HSQC(^{1}H-^{13}C} NMR spectrum of compound **15 (Procedure 1)** in $CDCl_3$.

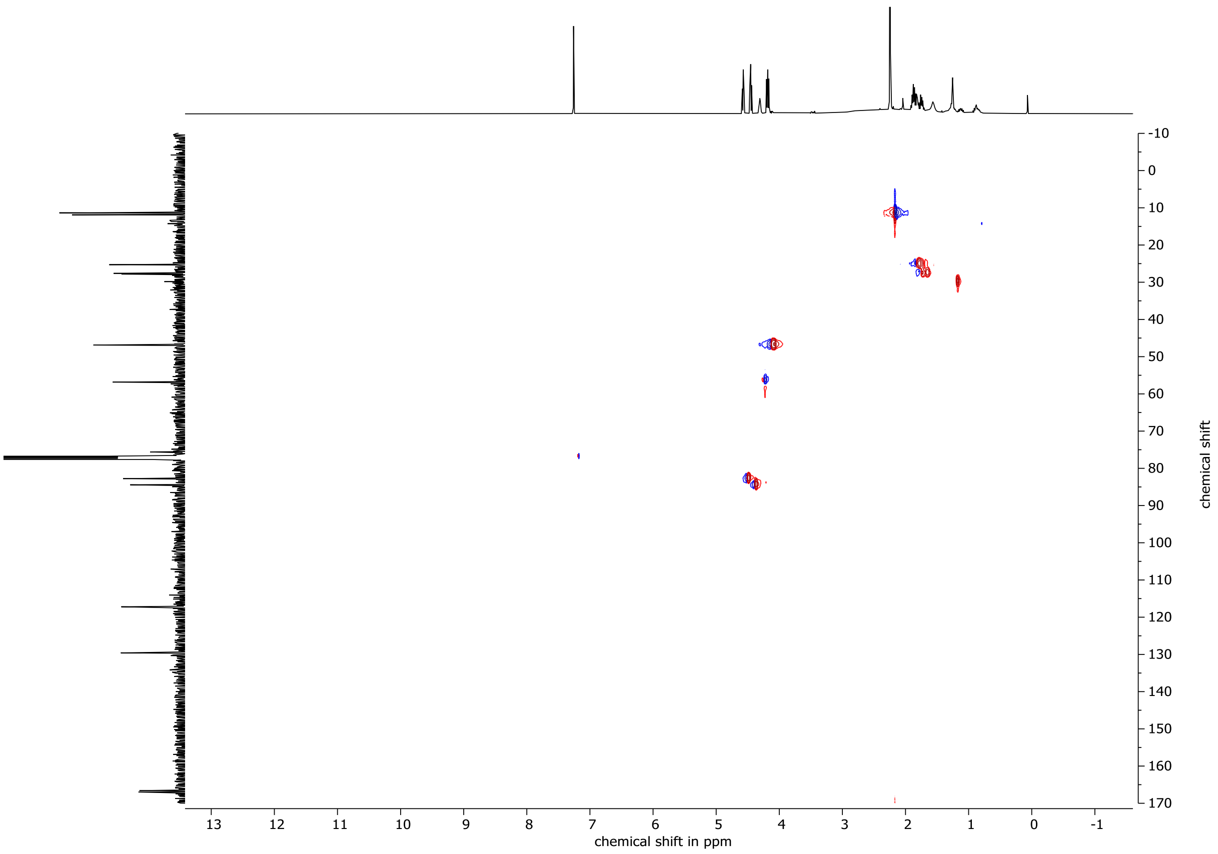

Figure S29. HMBC(^{1}H-^{13}C) NMR spectrum of compound **15 (Procedure 1)** in $CDCl_3$.

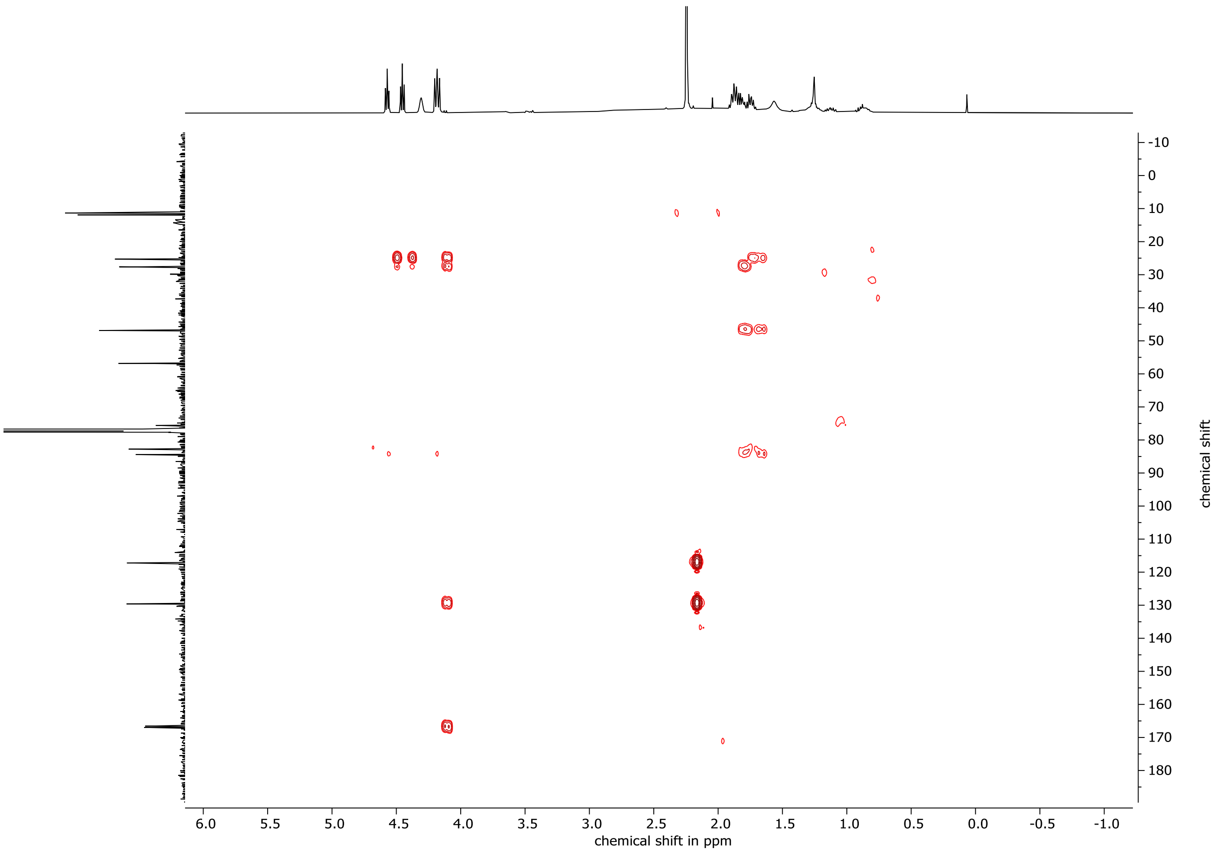

Figure S30. ^{1}H NMR spectrum of compound **15 (Procedure 2)** in $CDCl_3$.

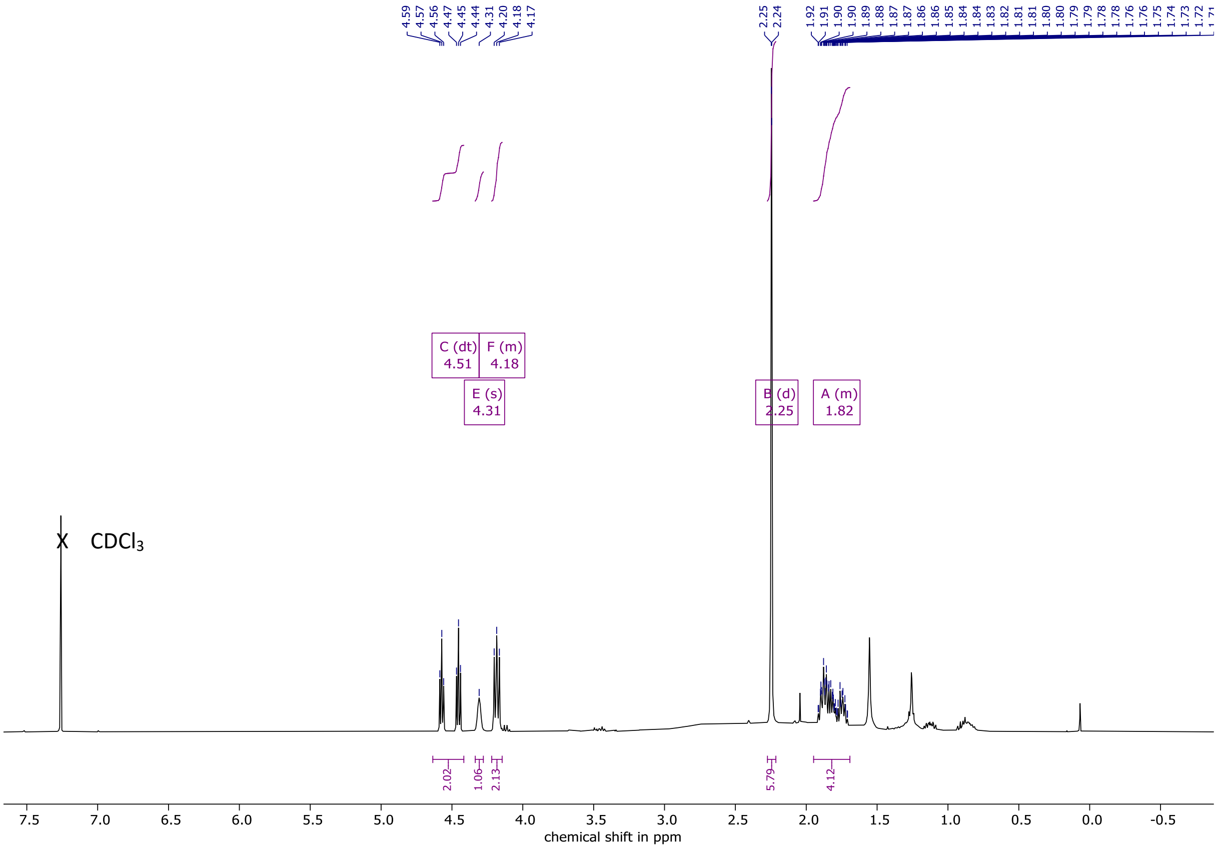

Figure S31. $^{11}B\{^{1}H\}$ NMR spectrum of compound **15 (Procedure 2)** in $CDCl_3$.

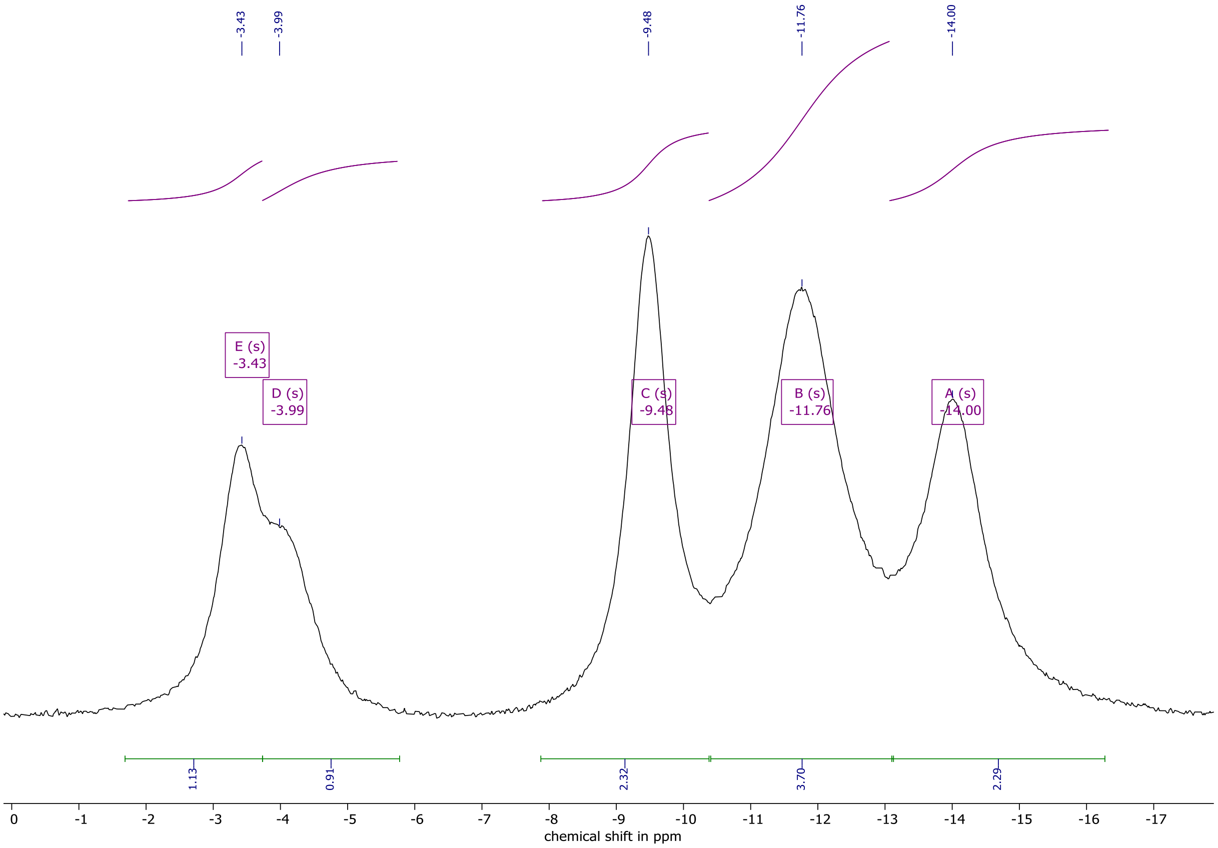

Supporting Information

Figure S32. COSY(^{1}H-^{1}H) NMR spectrum of compound **15 (Procedure 2)** in $CDCl_3$.

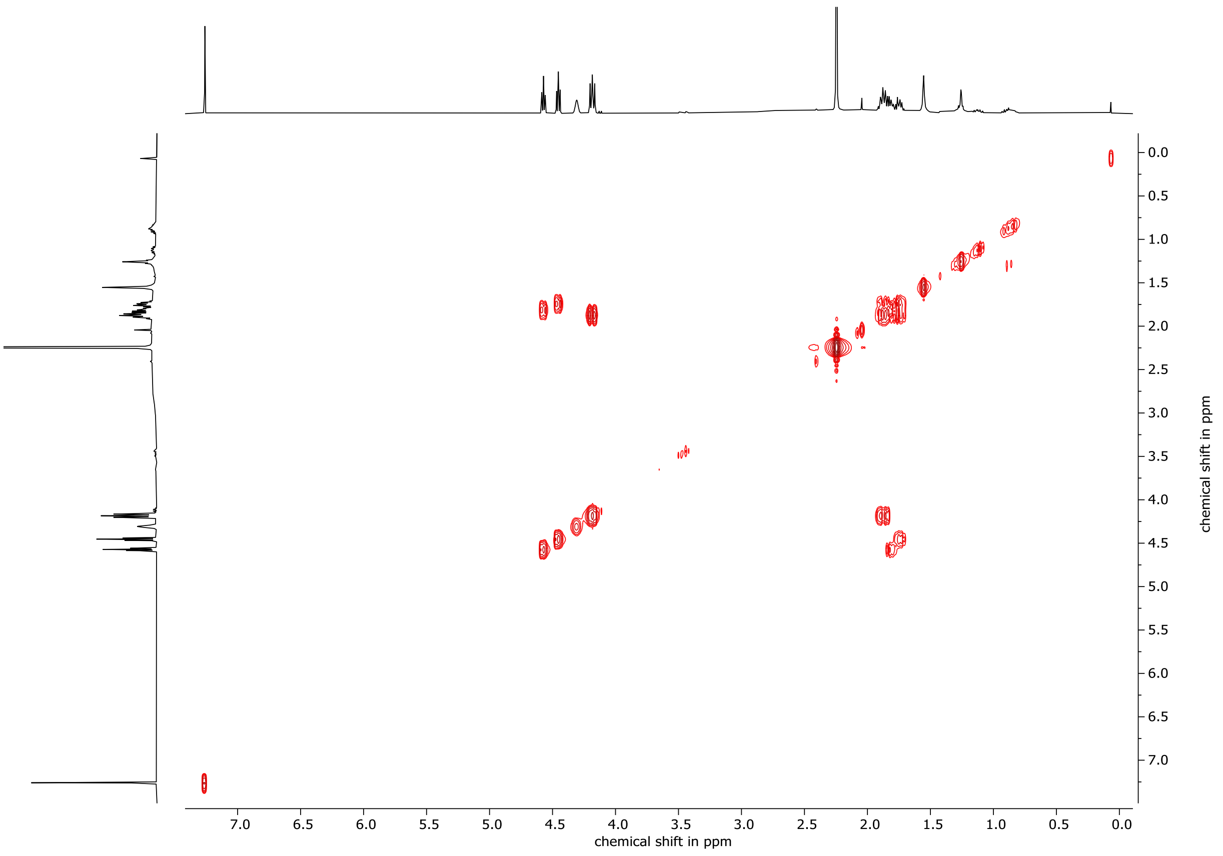

Figure S33. ^{1}H NMR spectrum of compound **LUZ5** in $CDCl_3$.

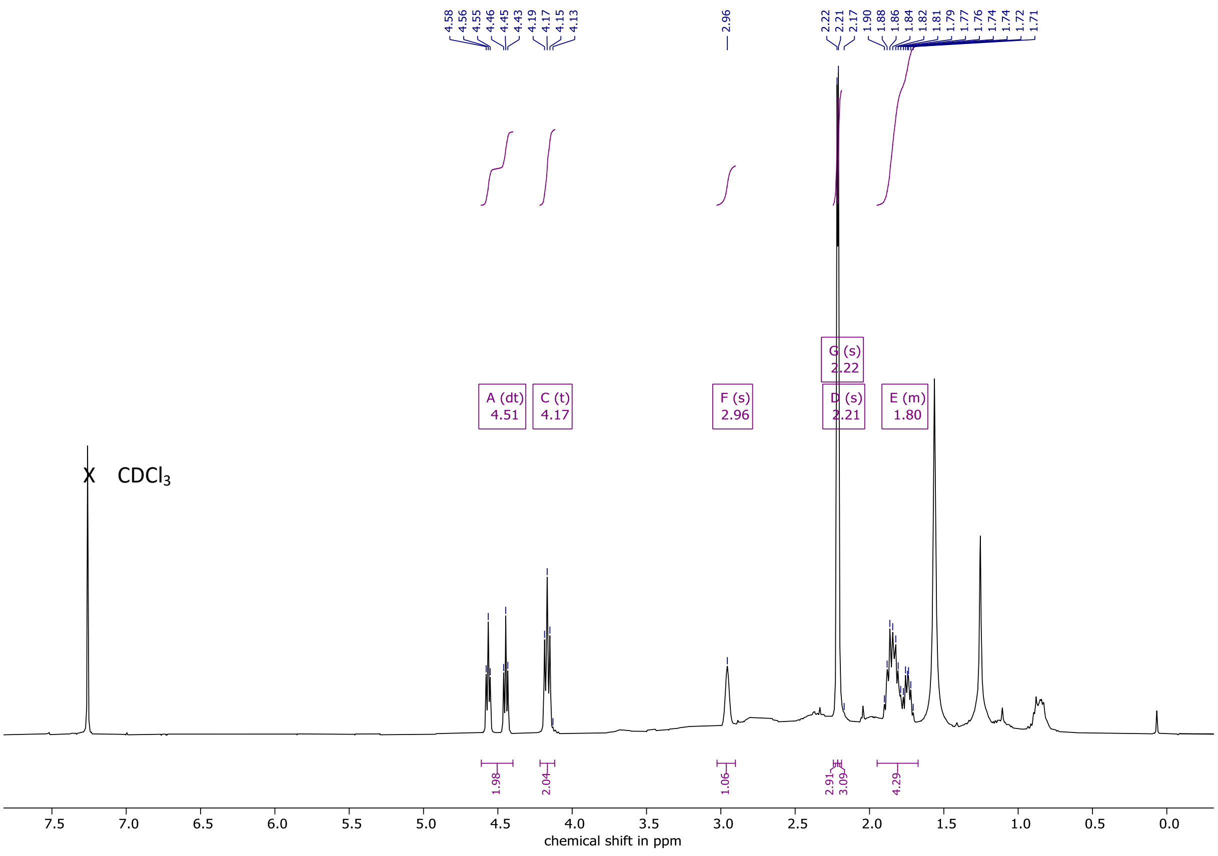

Figure S34. $^{11}B\{^{1}H\}$ NMR spectrum of compound **LUZ5** in $CDCl_3$.

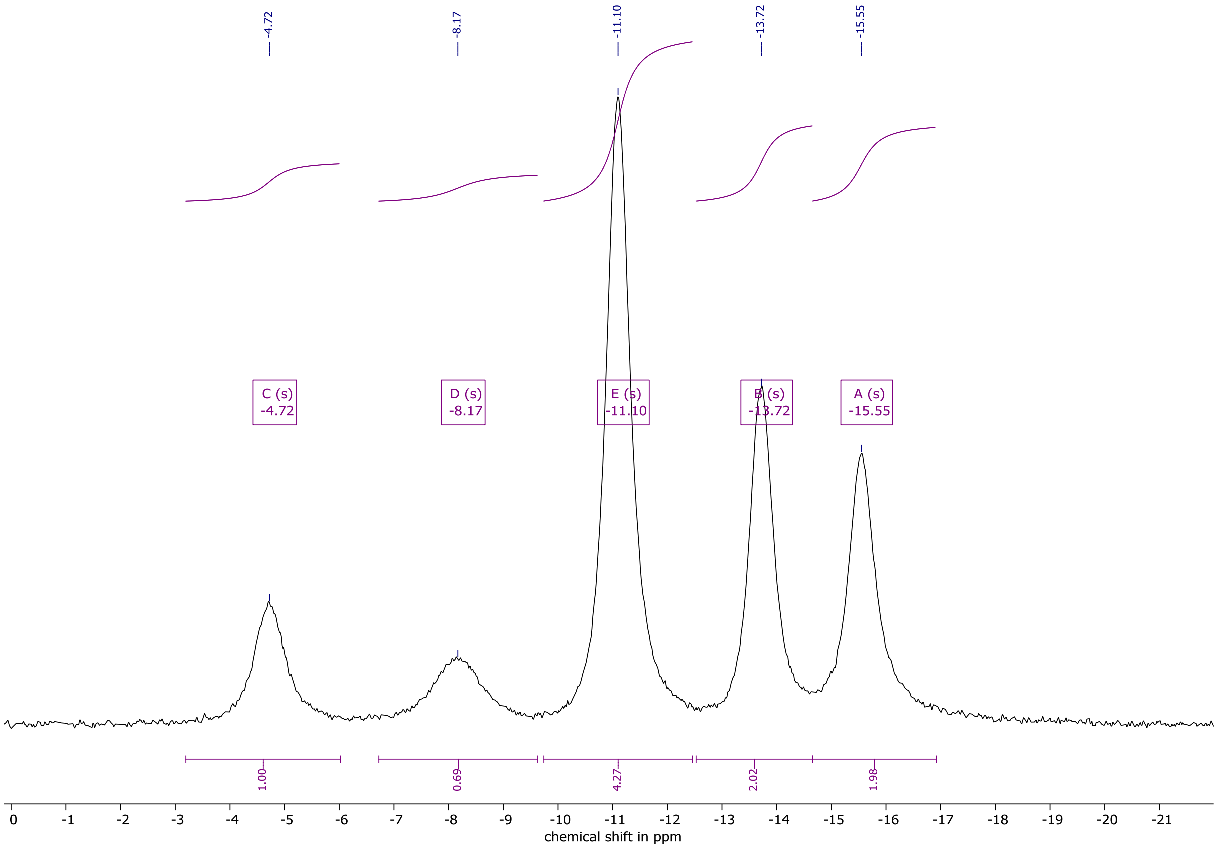

Figure S35. $^{13}C\{^{1}H\}$ NMR spectrum of compound **LUZ5** in $CDCl_3$.

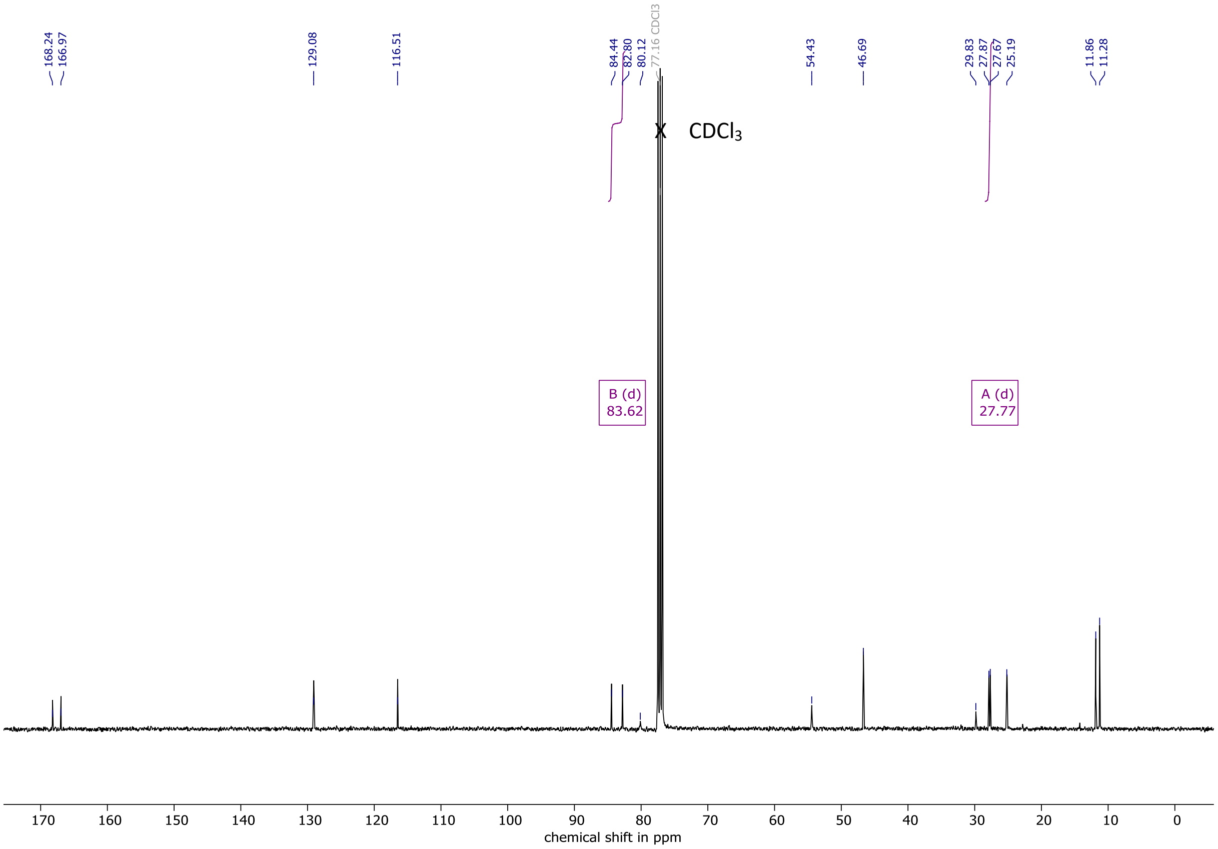

Figure S36. COSY(^1H-^1H) NMR spectrum of compound **LUZ5**.

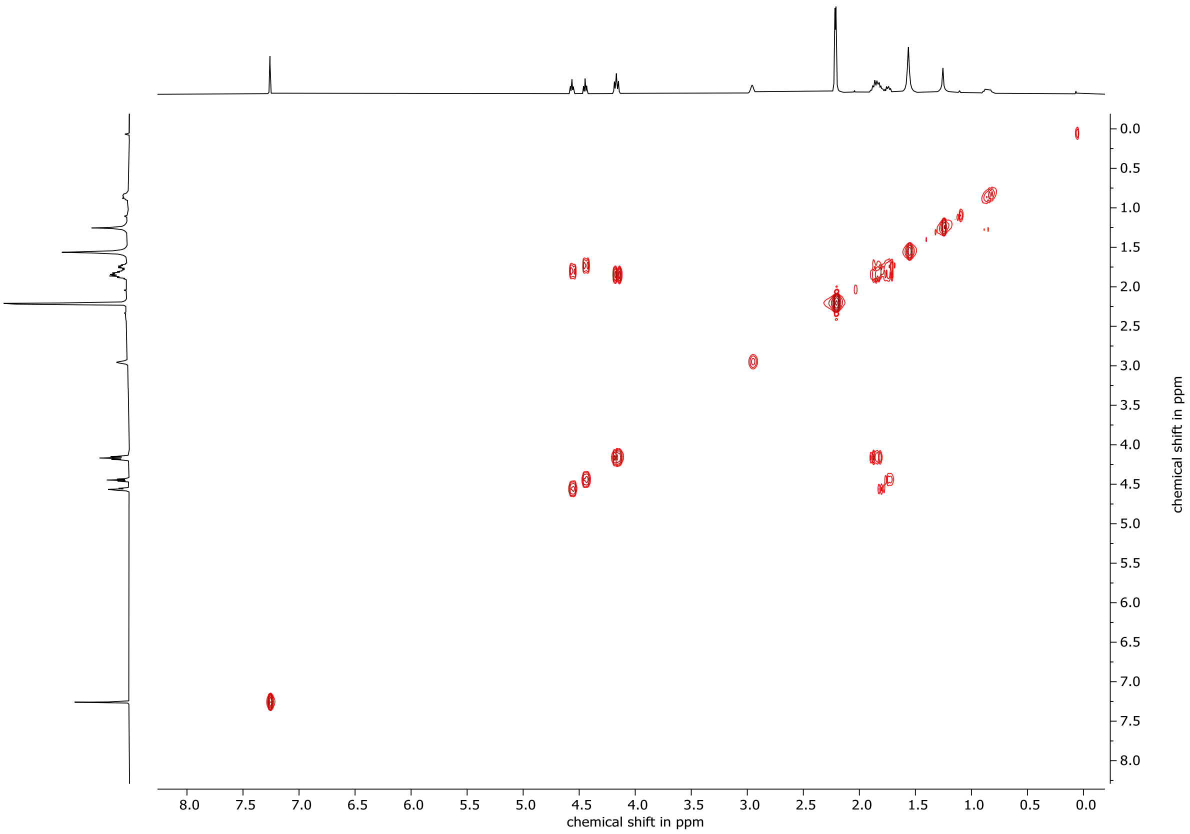

Figure S37. HSQC(^1H-^{13}C} NMR spectrum of compound **LUZ5** in $CDCl_3$.

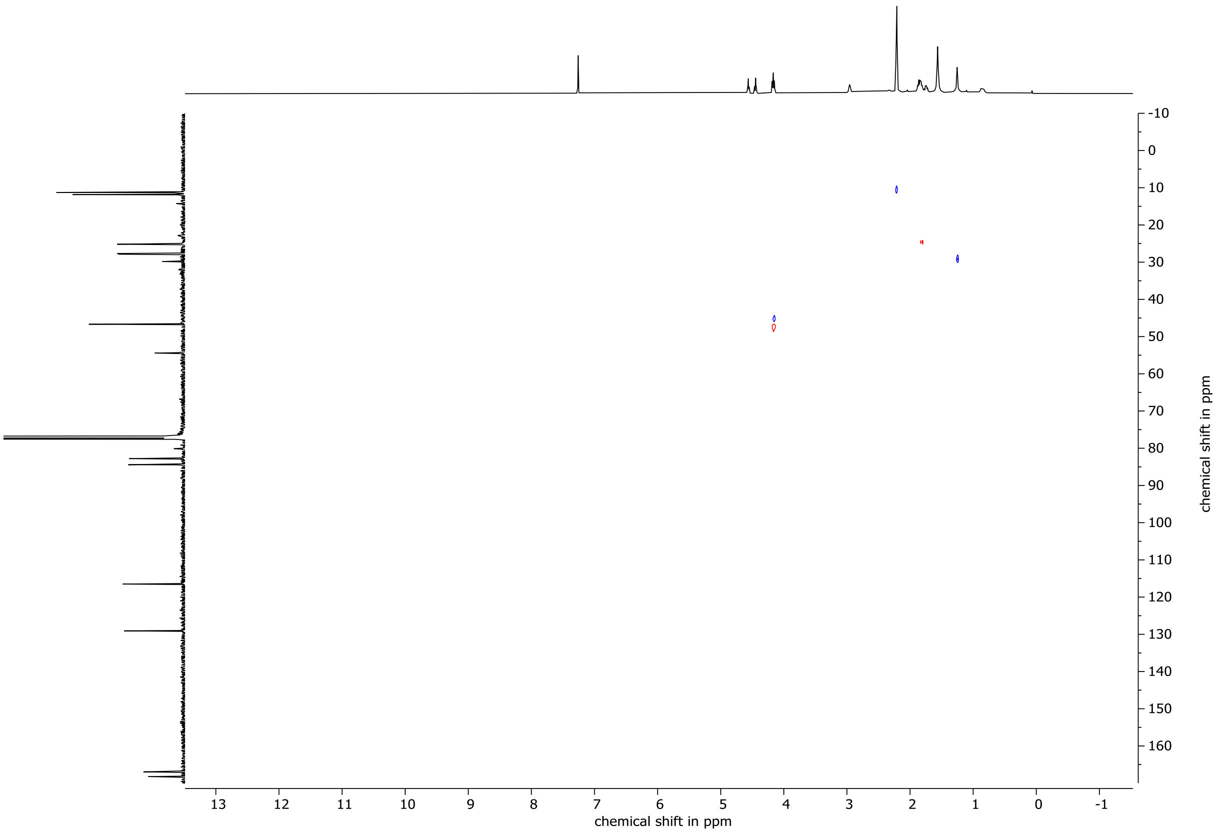

Figure S38. HMBC(^{1}H-^{13}C) NMR spectrum of compound **LUZ5** in $CDCl_3$.

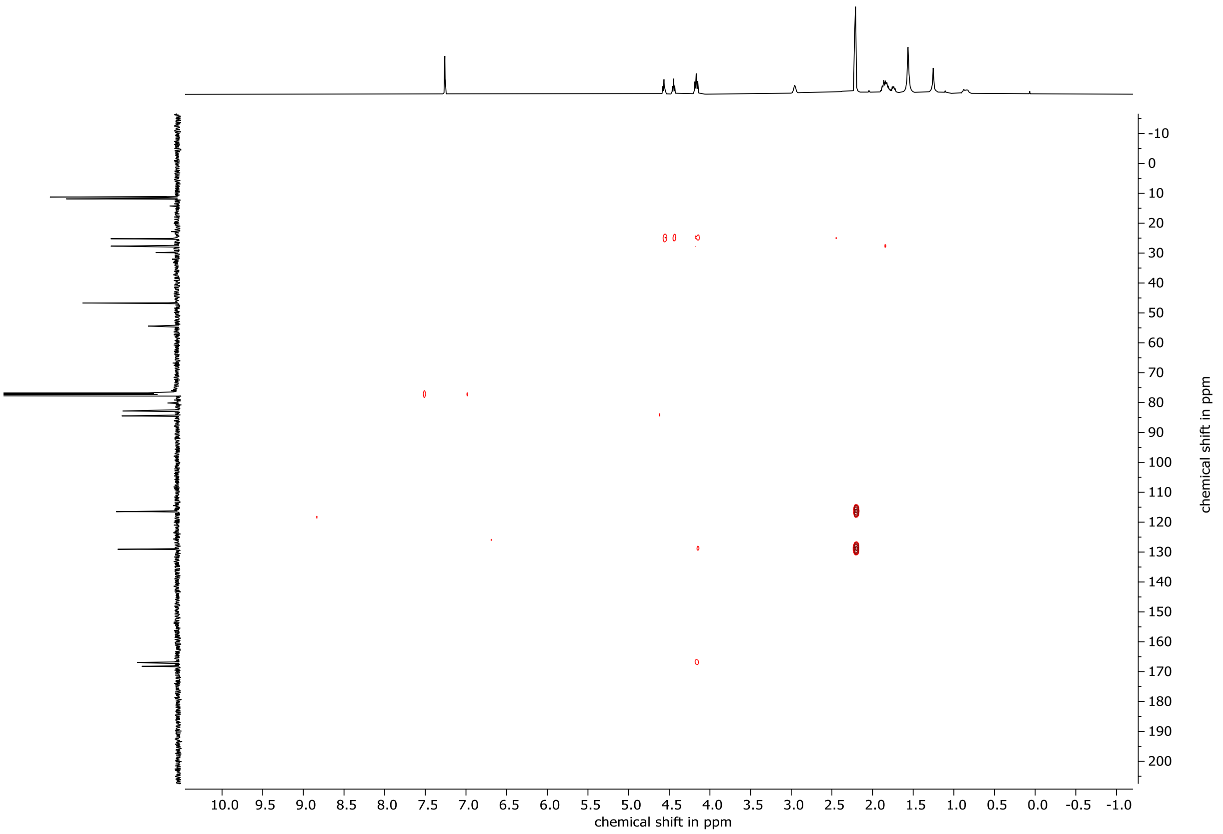

Figure S39. ^{1}H NMR spectrum of side product **SP1** of **LUZ5** in $CDCl_3$.

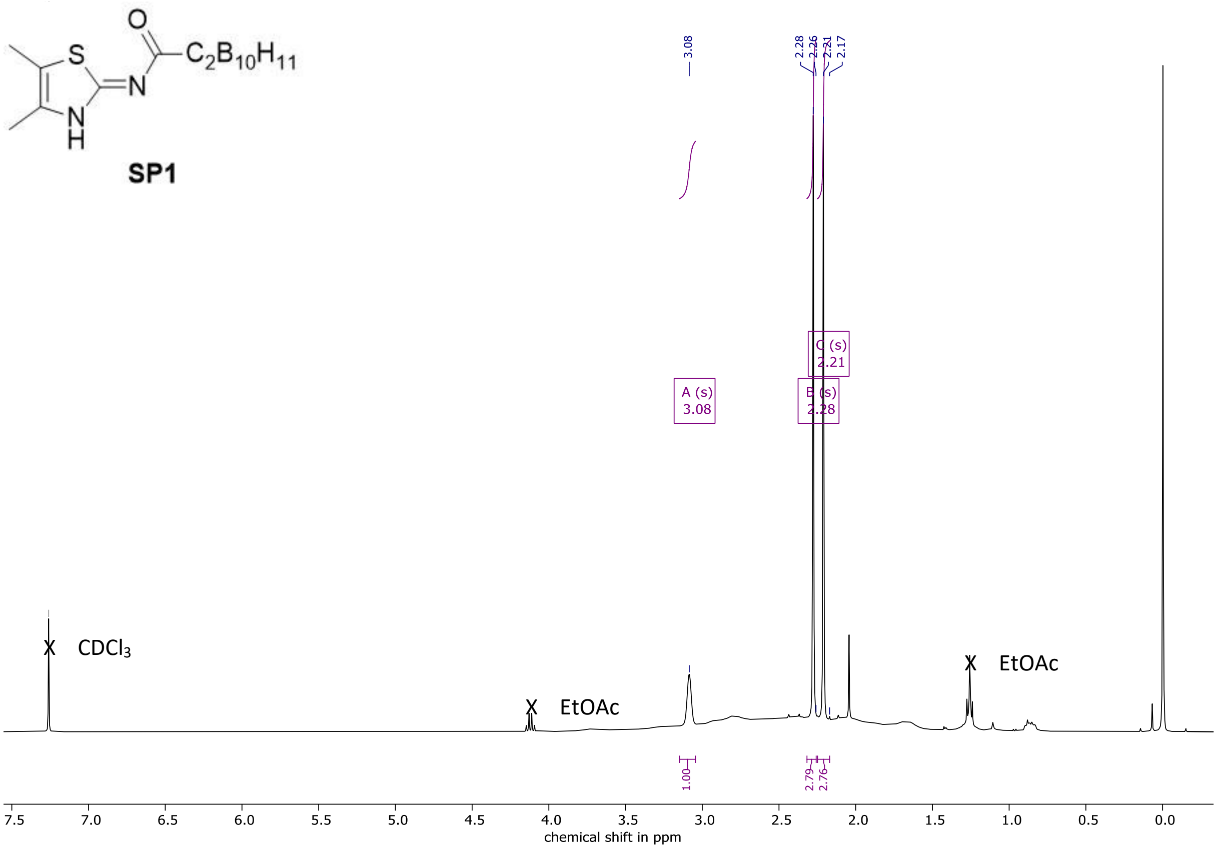

Figure S40. $^{11}B\{^{1}H\}$ NMR spectrum of side product **SP1** of **LUZ5** in $CDCl_3$.

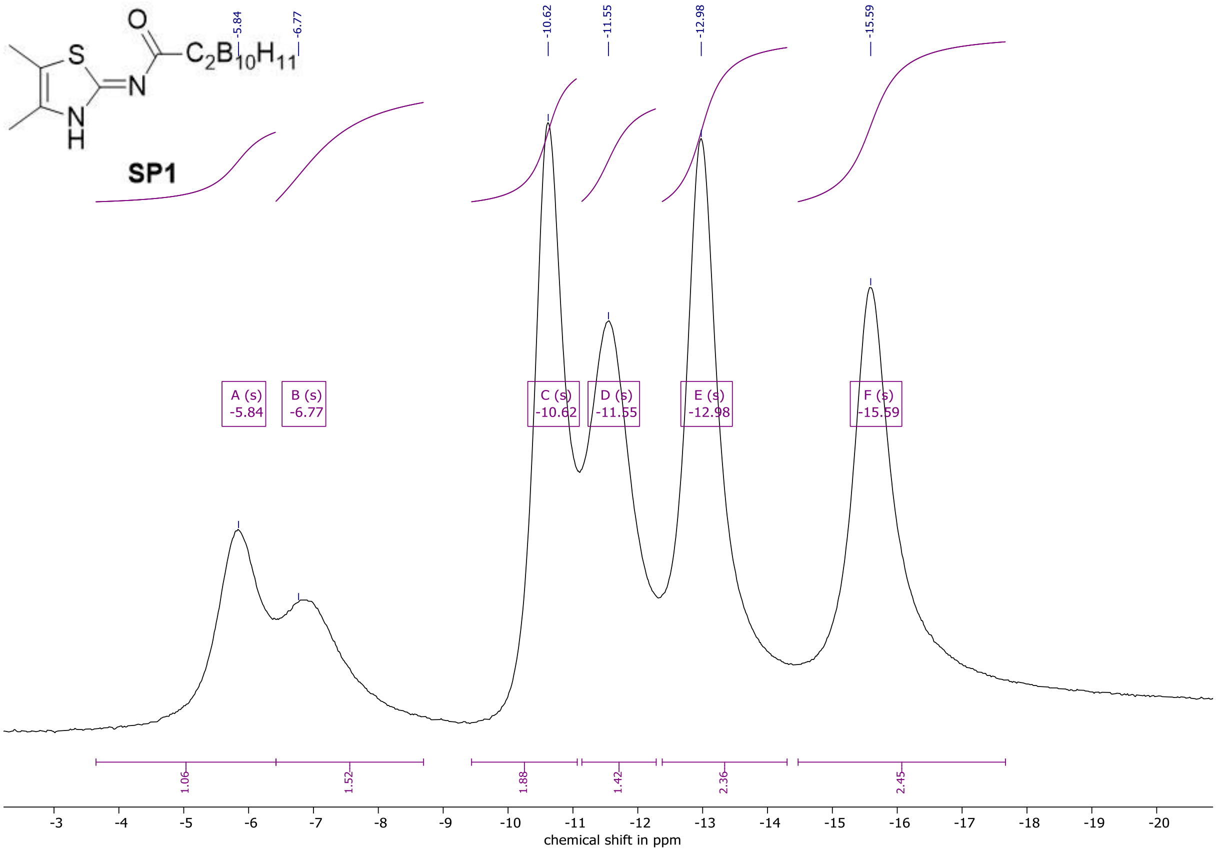

Figure S41. ^{1}H NMR spectrum of compound **17** in $CDCl_3$.

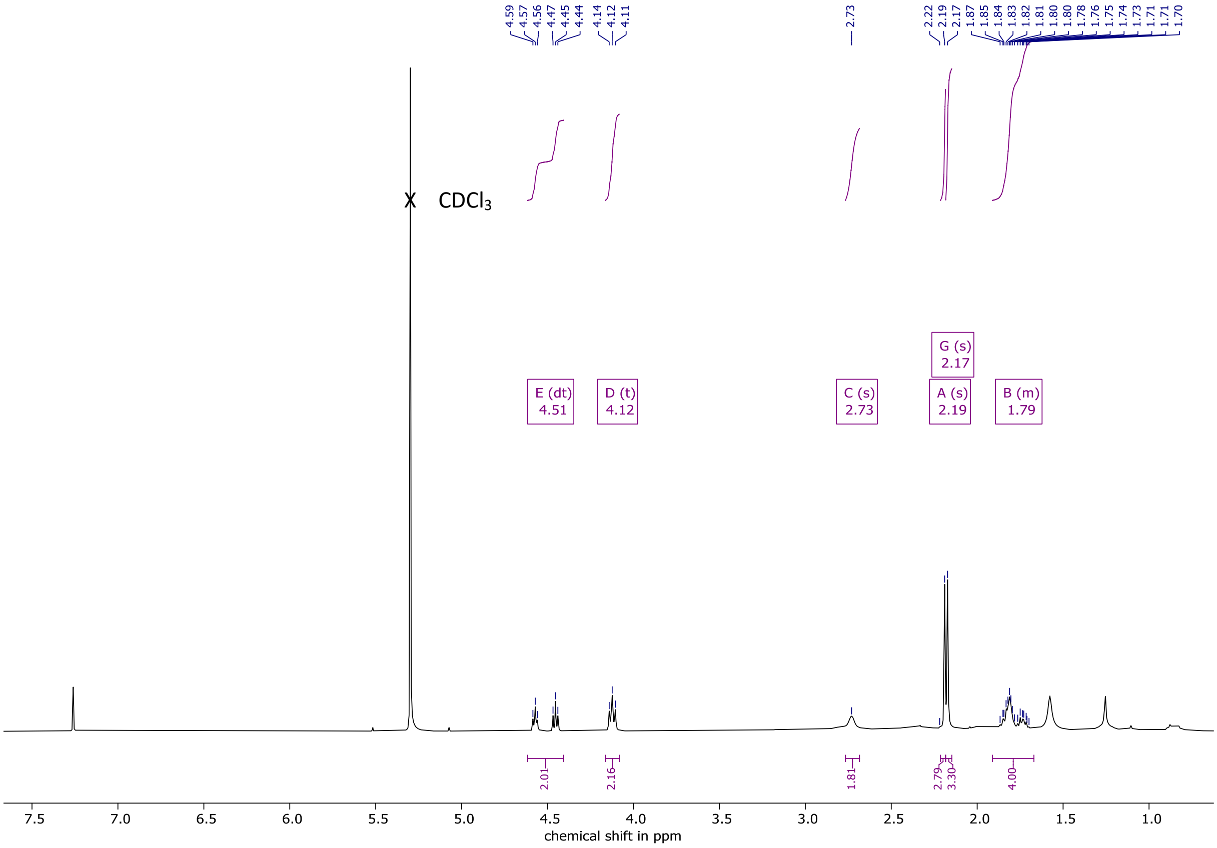

Figure S42. $^{11}B\{^{1}H\}$ NMR spectrum of compound **17** in $CDCl_3$.

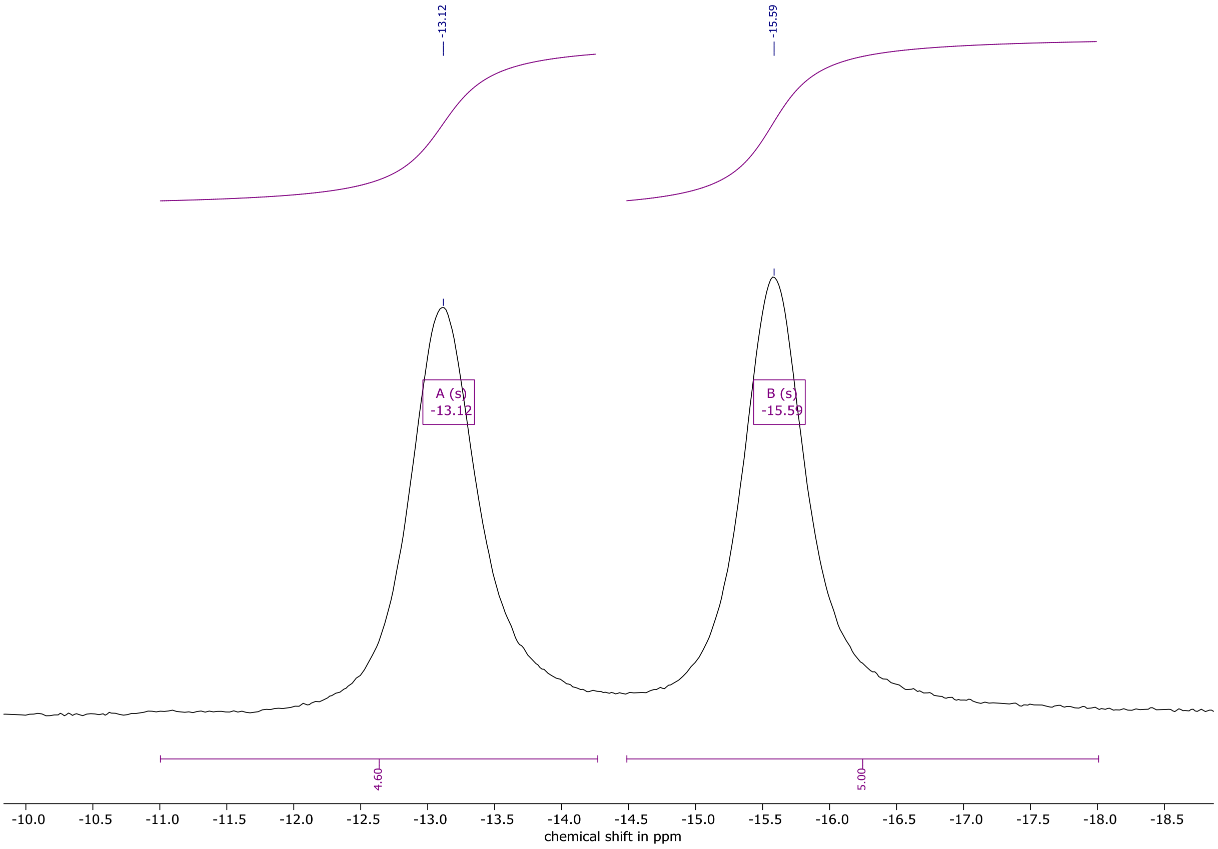

Figure S43. $^{13}C\{^{1}H\}$ NMR spectrum of compound **17** in $CDCl_3$.

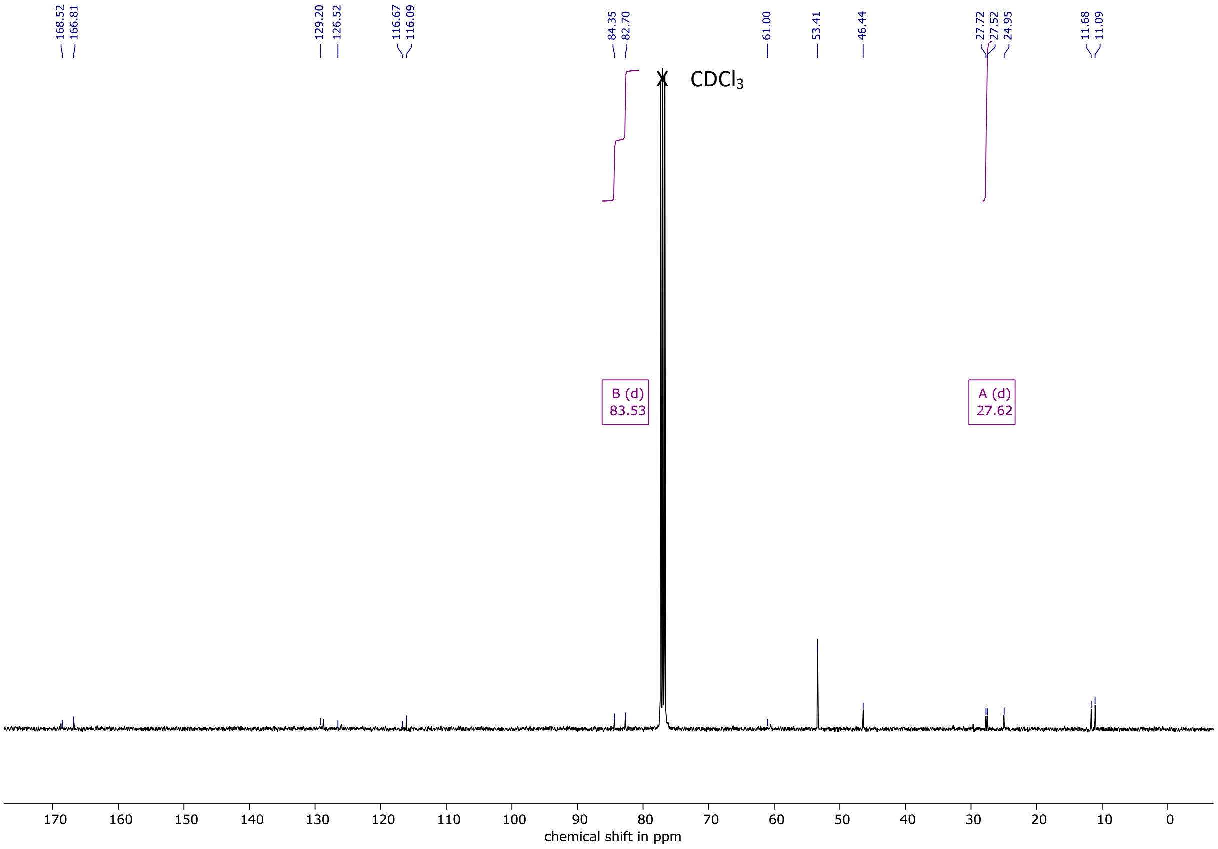

Figure S44. COSY(^1H-^1H) NMR spectrum of compound **17** in $CDCl_3$.

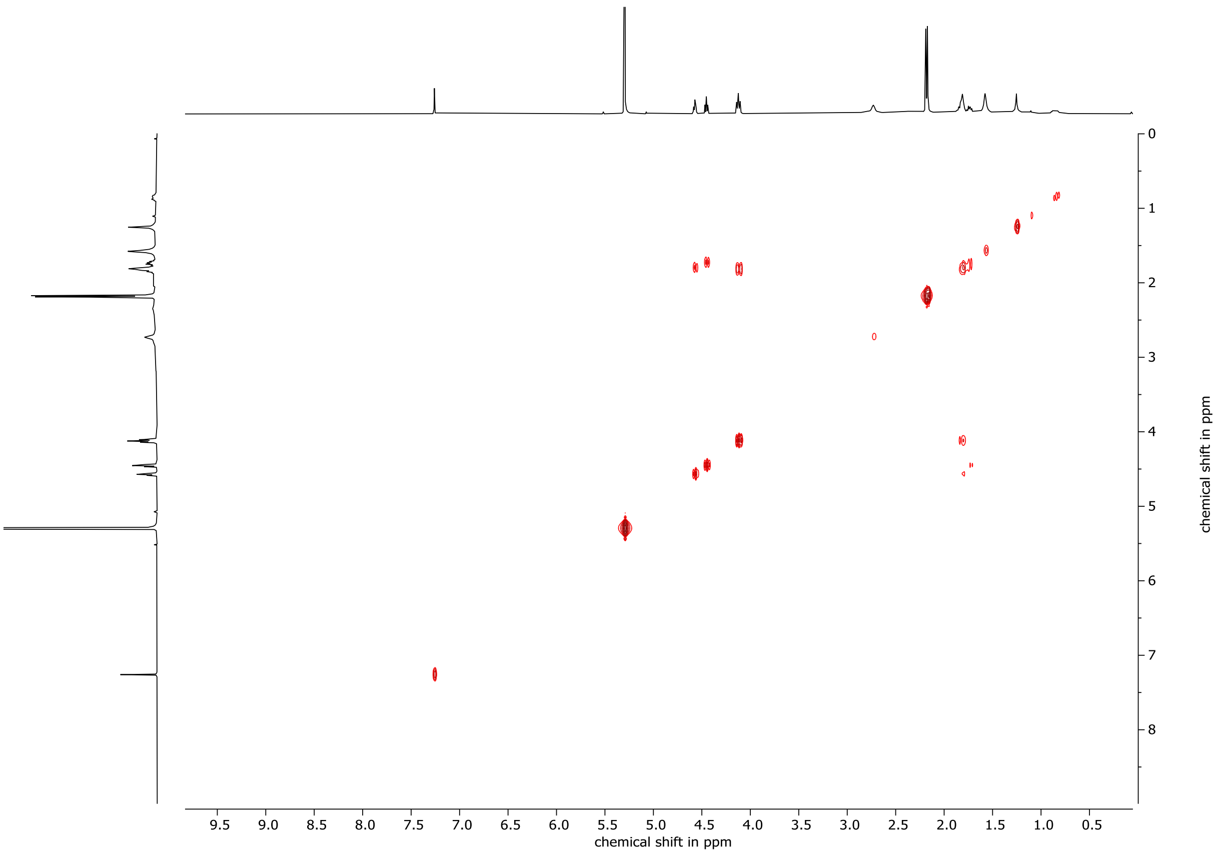

Figure S45. HSQC(^1H-^{13}C} NMR spectrum of compound **17** in $CDCl_3$.

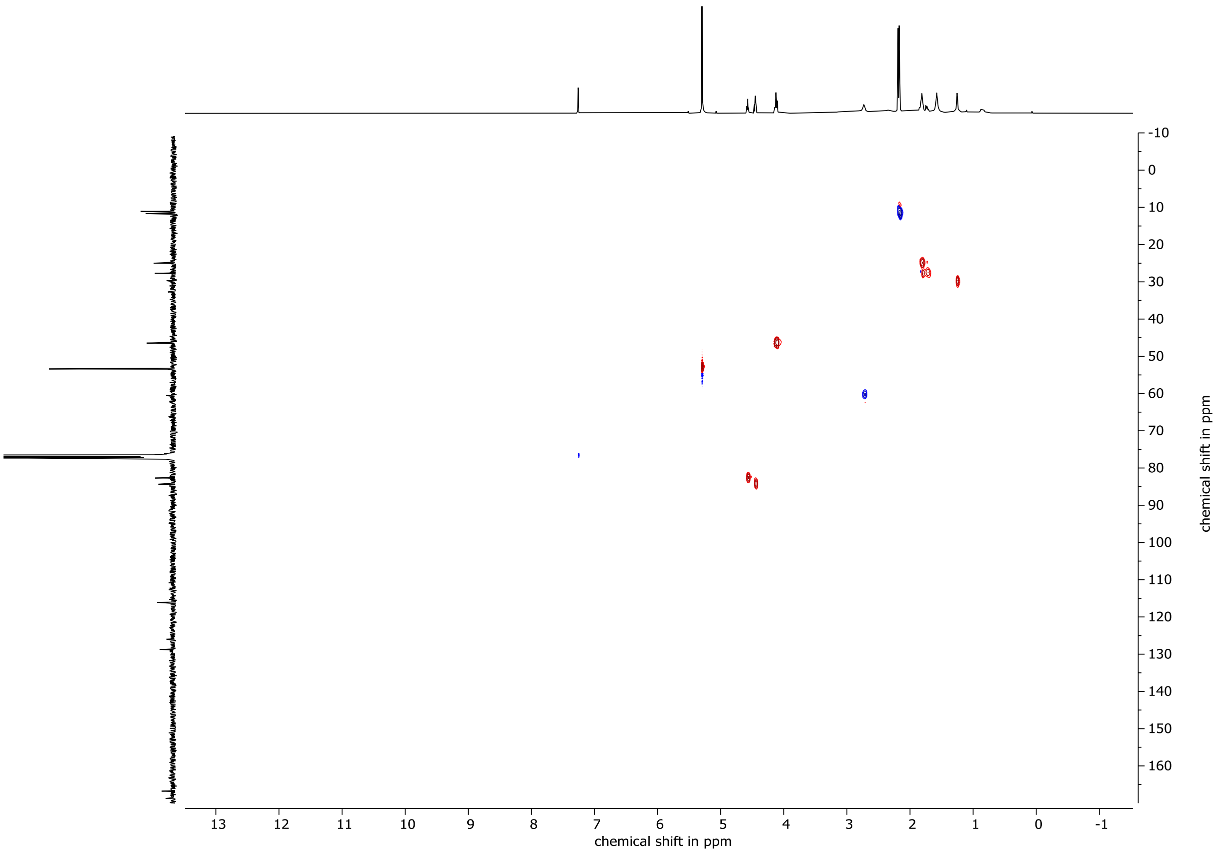

Figure S46. HMBC(^{1}H-^{13}C) NMR spectrum of compound **17** in $CDCl_3$**.**

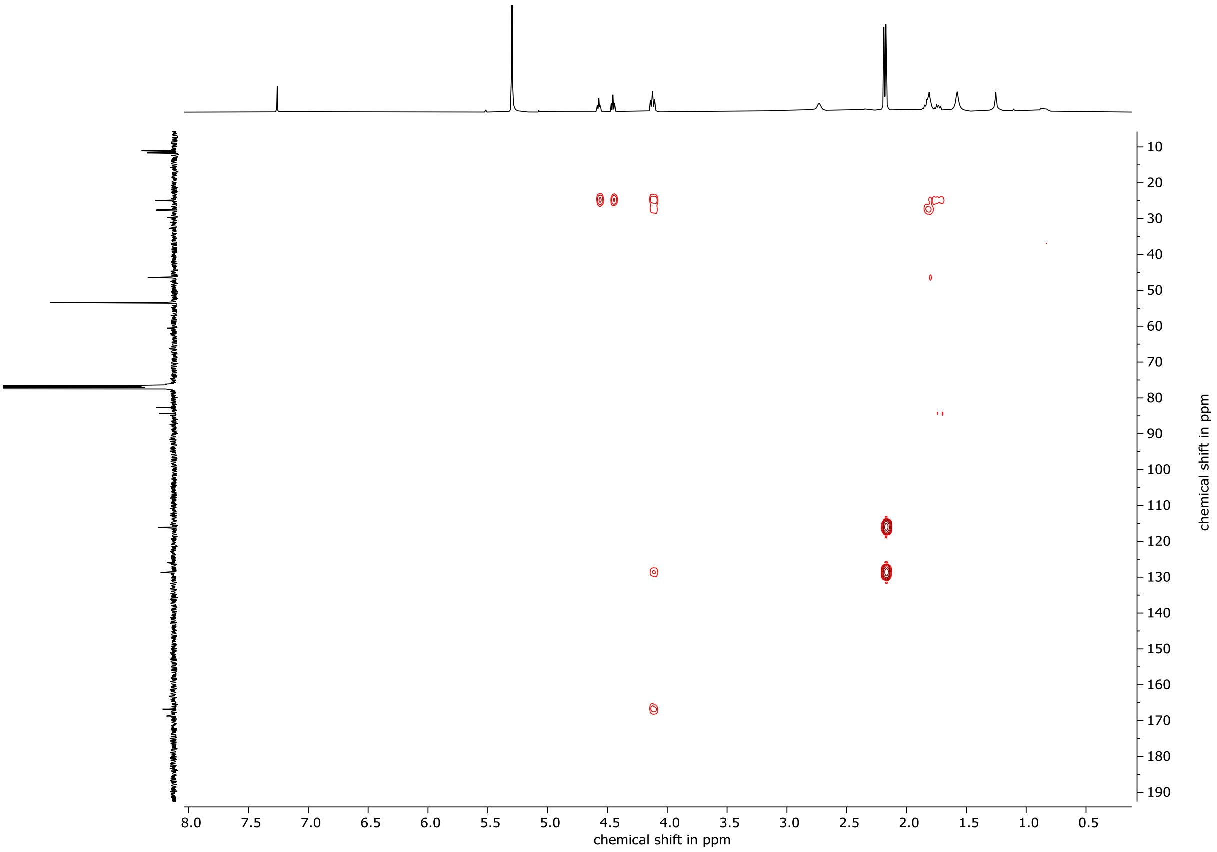

Figure S47. ^{1}H NMR spectrum of compound **18** in $CDCl_3$.

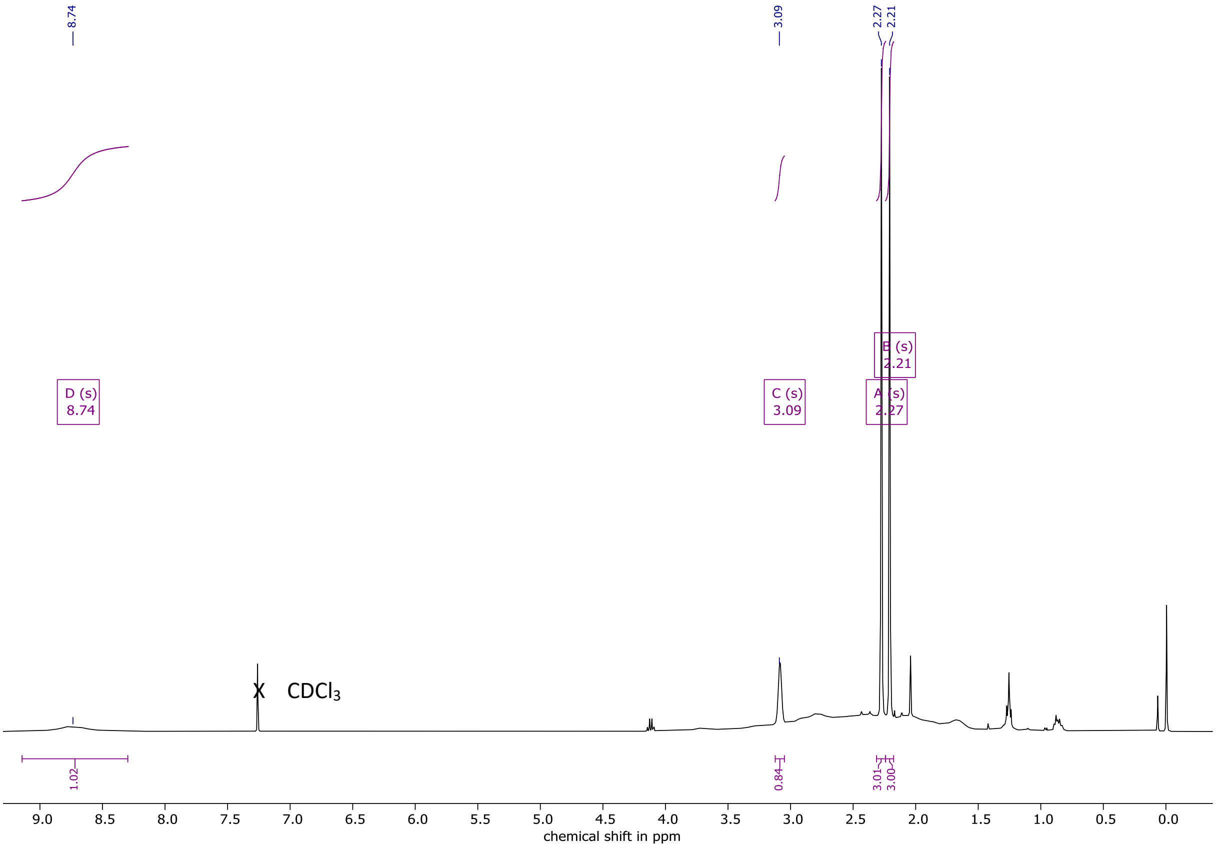

Figure S48. $^{11}B\{^{1}H\}$ NMR spectrum of compound **18** in $CDCl_3$.

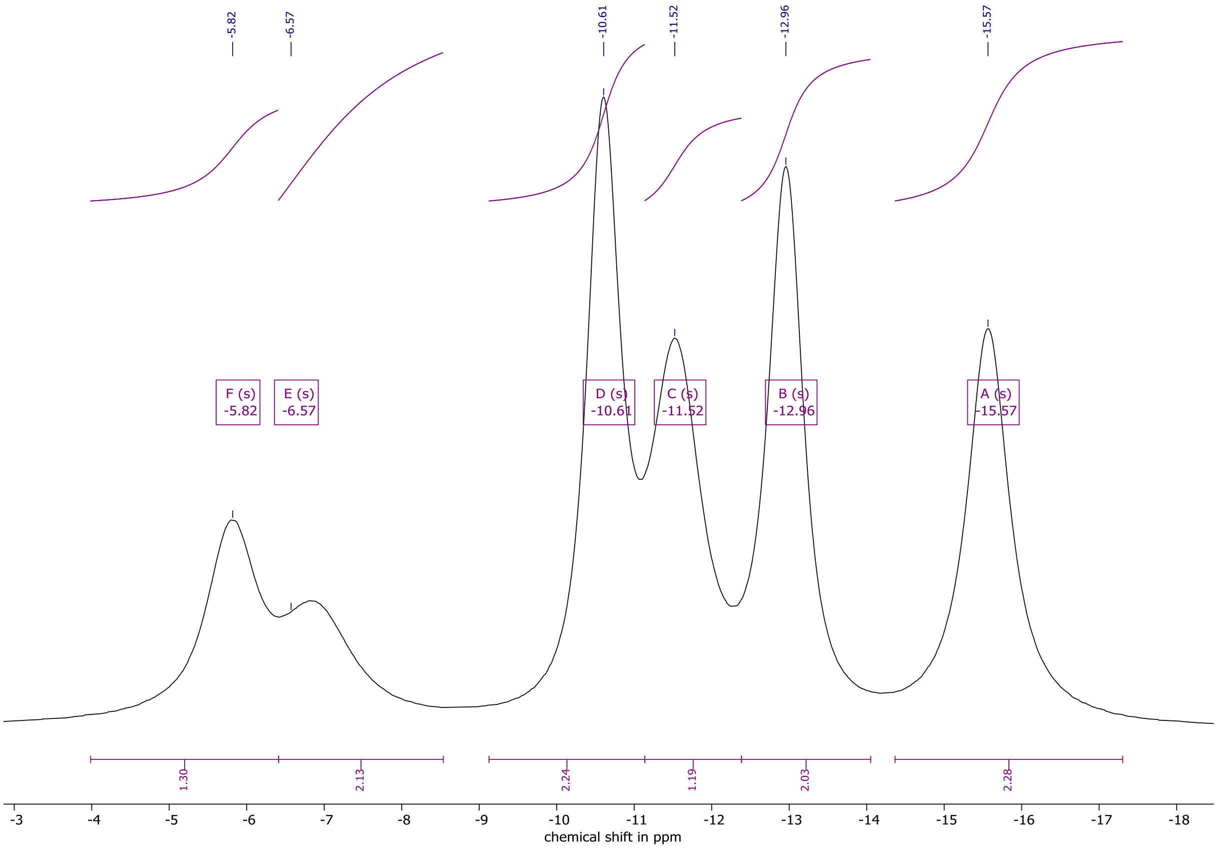

Figure S49. ^{1}H NMR spectrum of compound **19** in $CDCl_3$ before HPLC-purification.

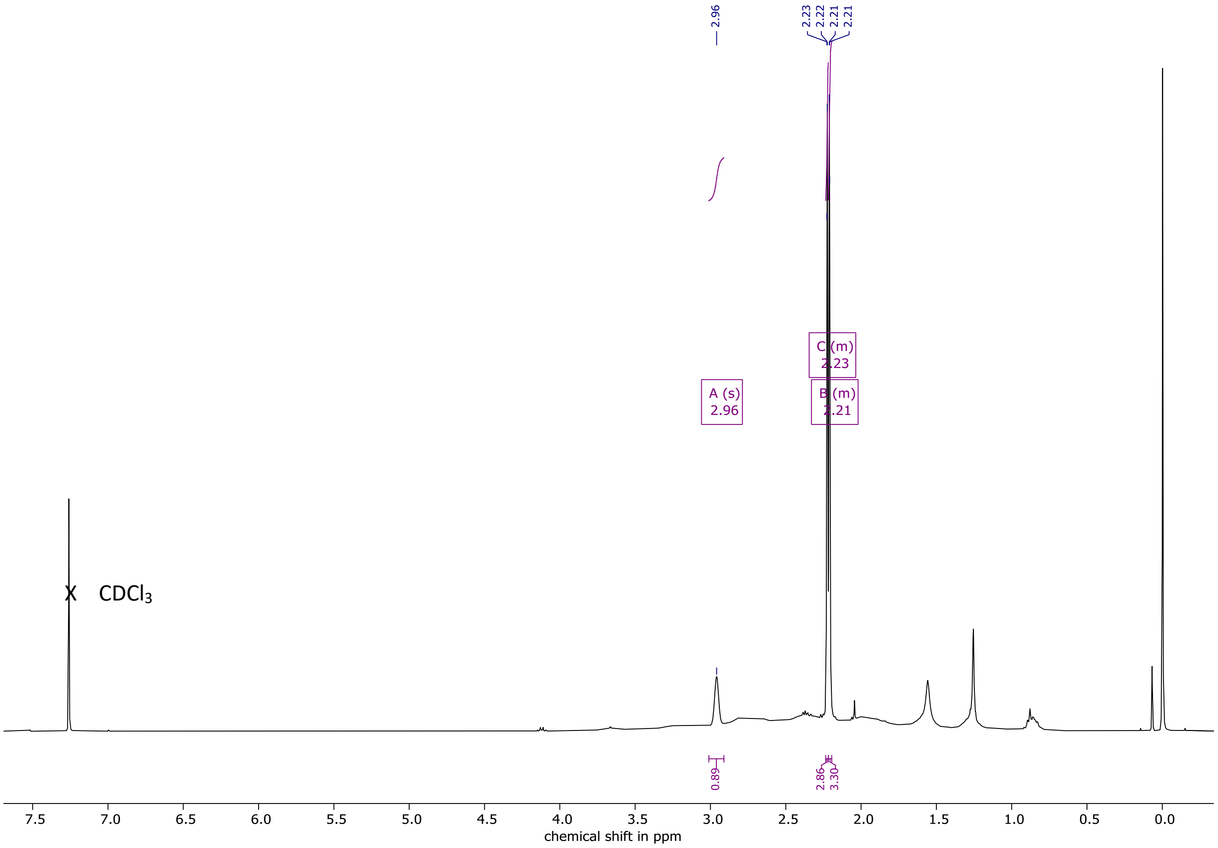

Figure S50. $^{11}B\{^{1}H\}$ NMR spectrum of compound **19** in $CDCl_3$ before HPLC-purification.

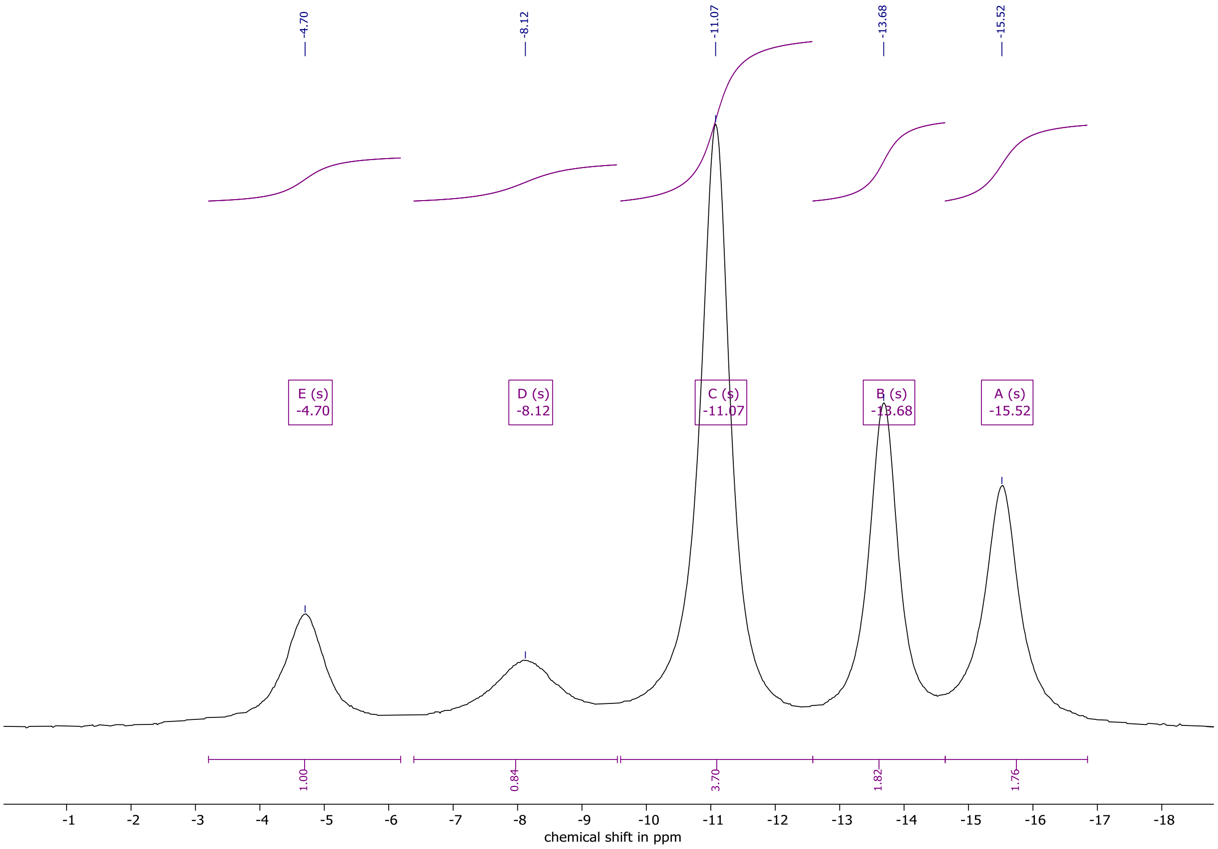

Figure S51. $^{13}C\{^{1}H\}$ NMR spectrum of compound **19** in $CDCl_3$ before HPLC-purification.

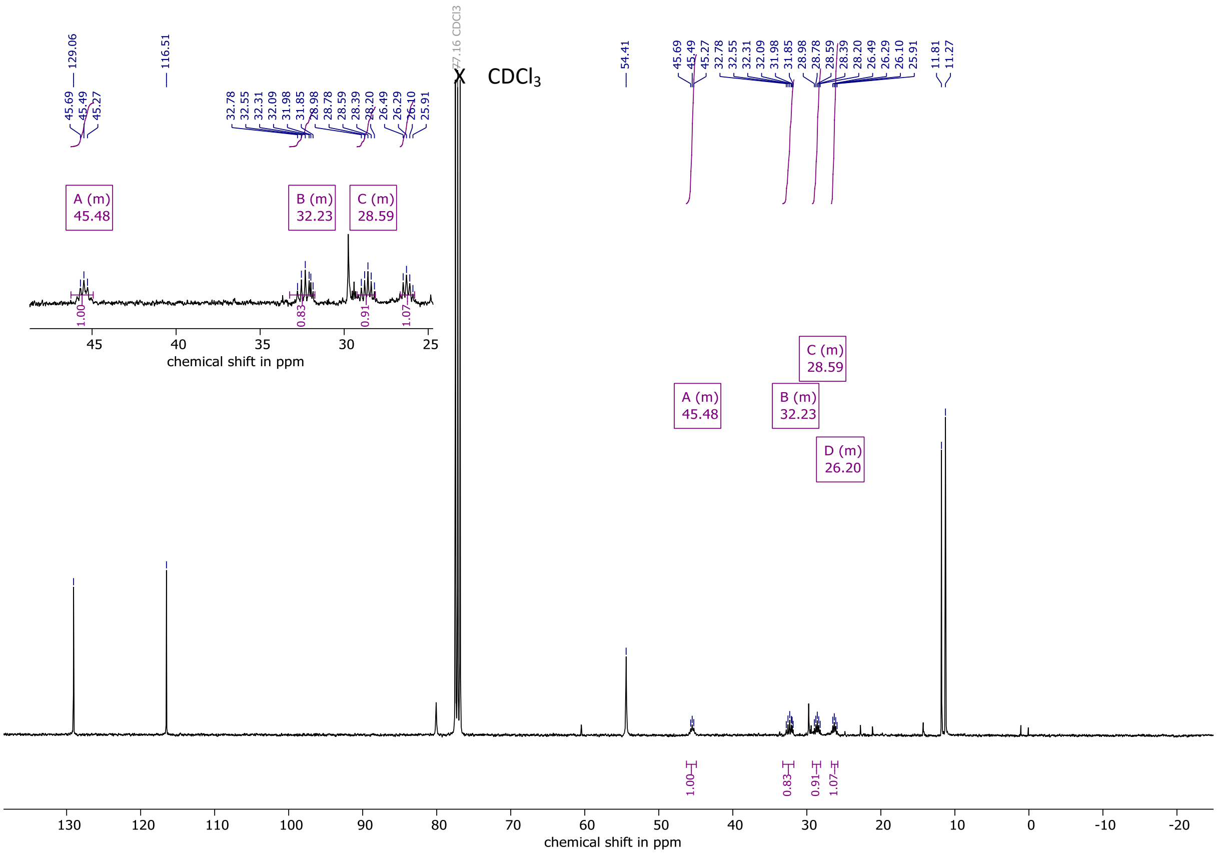

2 HR-ESI Mass Spectra of Compounds 9, 10, 11, 15 (Procedure 1, 2), LUZ5, SP1, 17, 19

Figure S52. HR(ESI+) mass spectrum of compound **9** in CH_3CN.

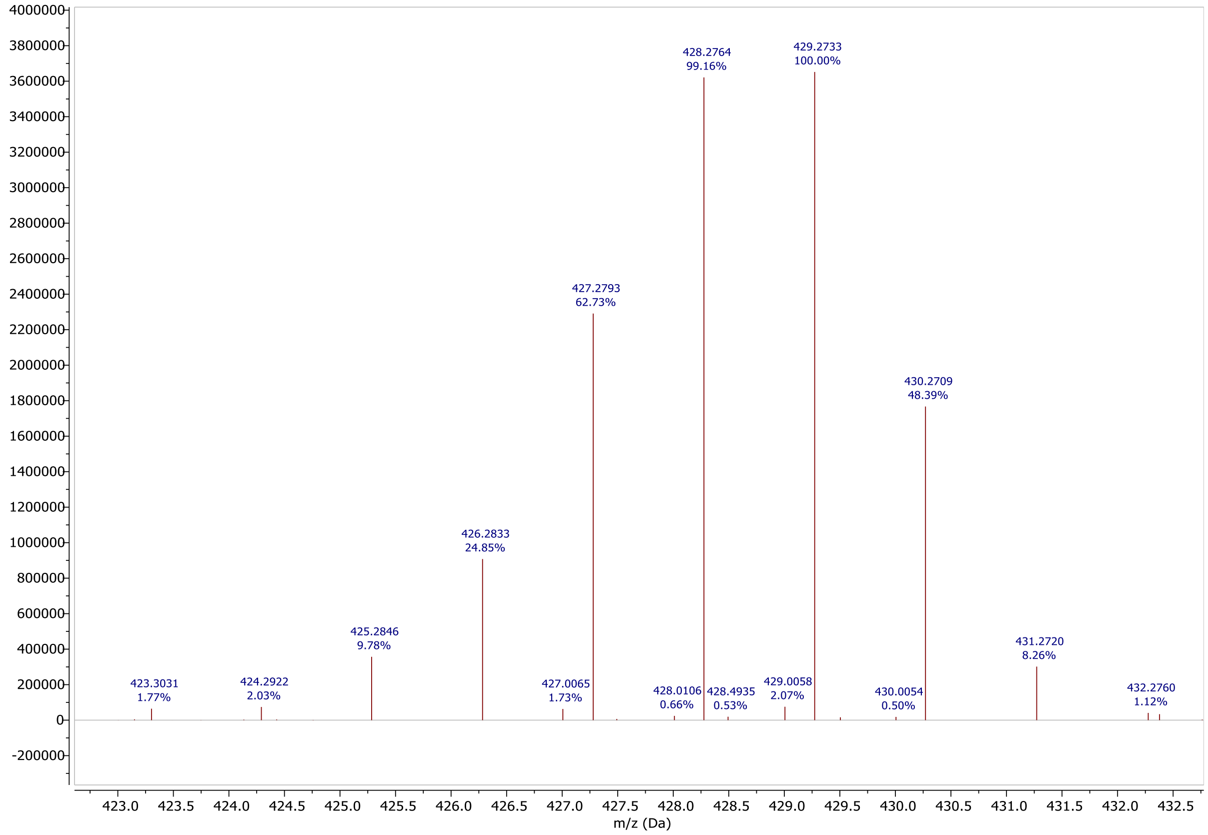

Supporting Information

Figure S53. HR(ESI+) mass spectrum of compound **10** in CH_3CN.

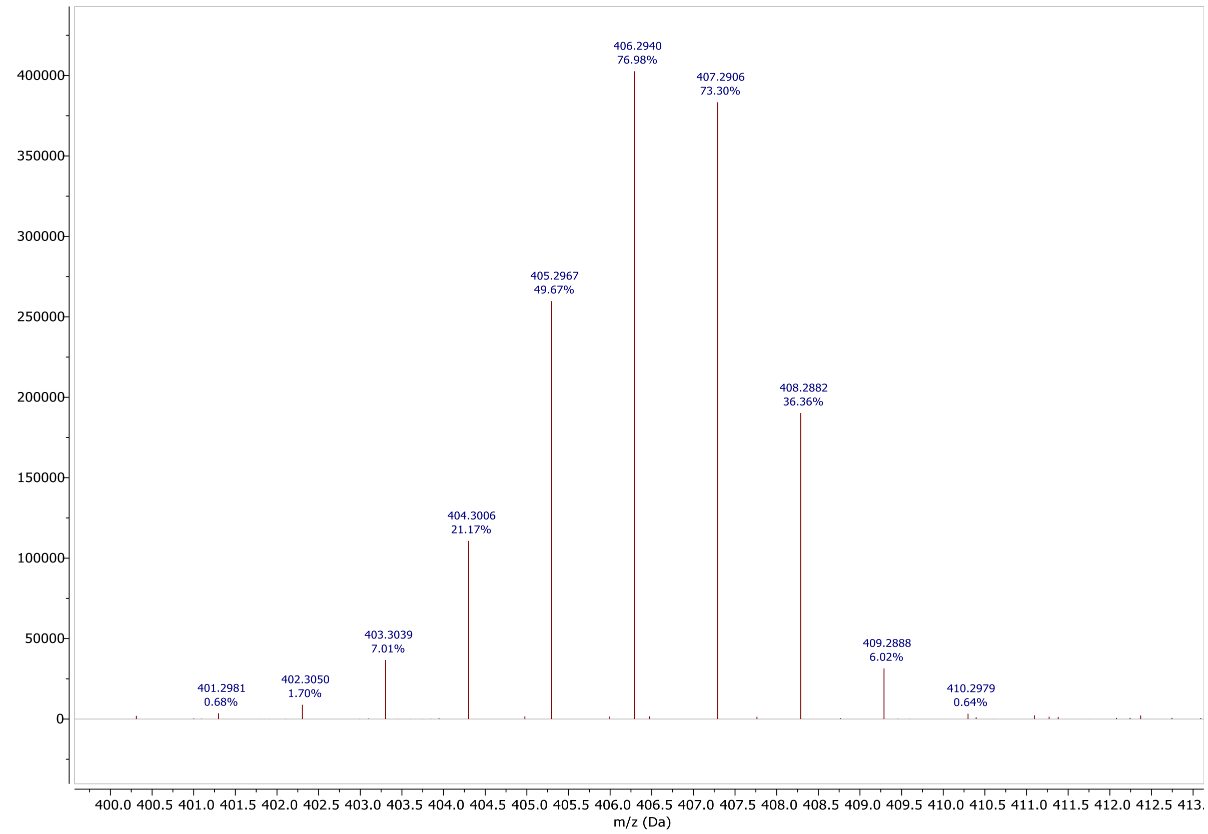

Figure S54. HR(ESI+) mass spectrum of compound **11** in CH_3CN.

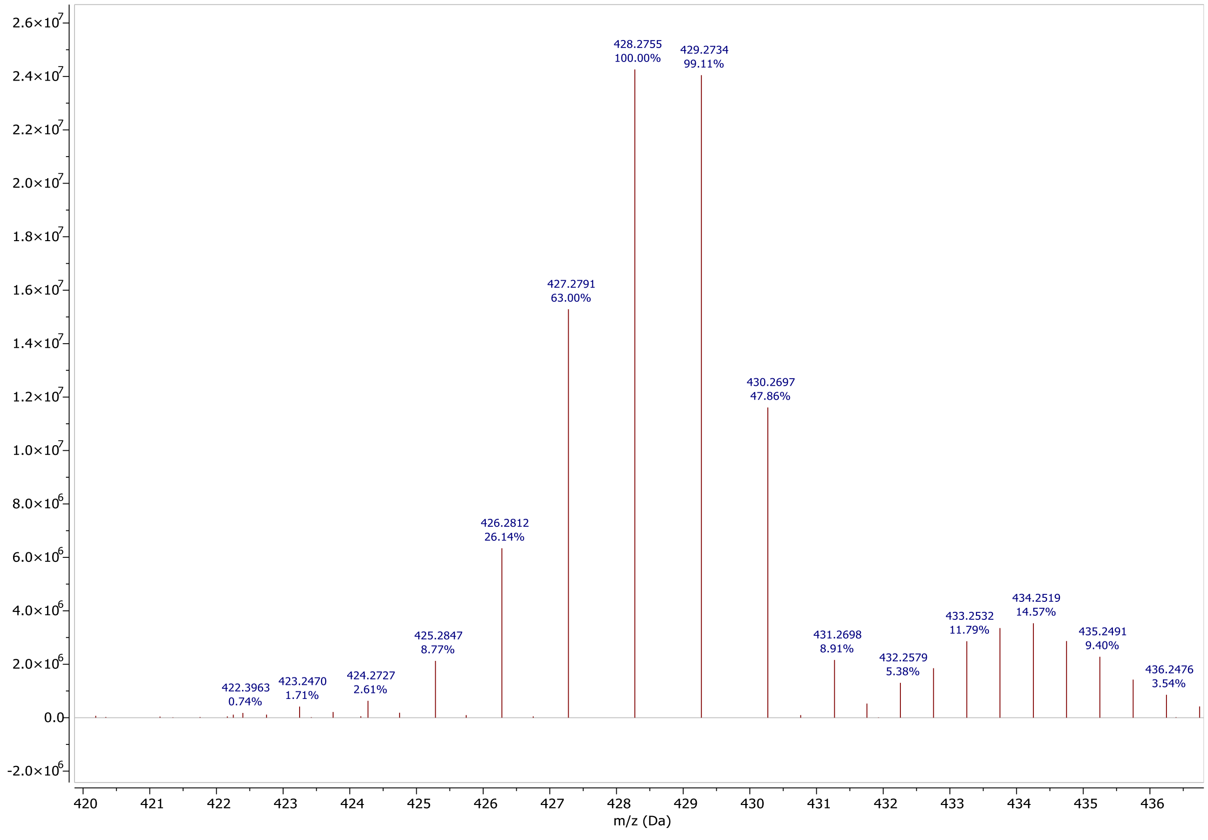

Supporting Information

Figure S55. HR(ESI+) mass spectrum of compound **15 (Procedure 1)** in CH_3CN.

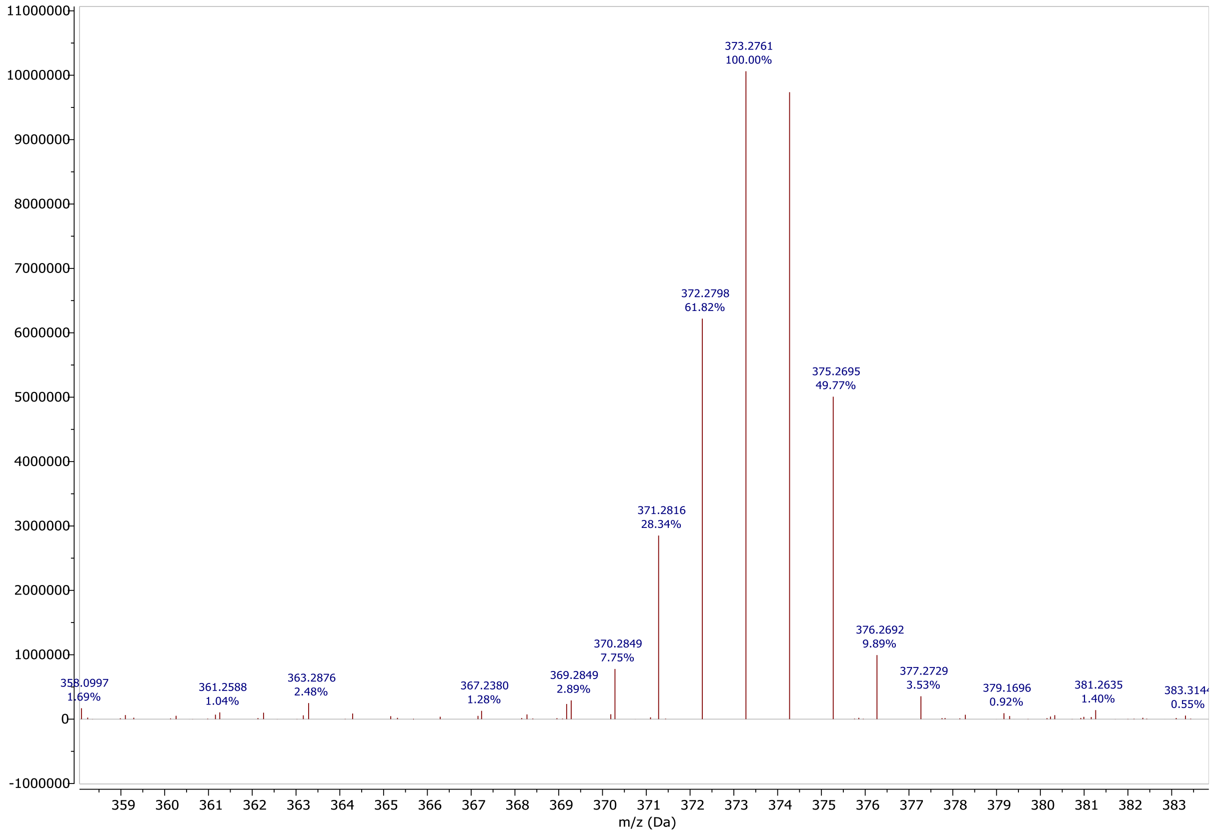

Figure S56. HR(ESI+) mass spectrum of compound **15 (Procedure 2)** in CH_3CN.

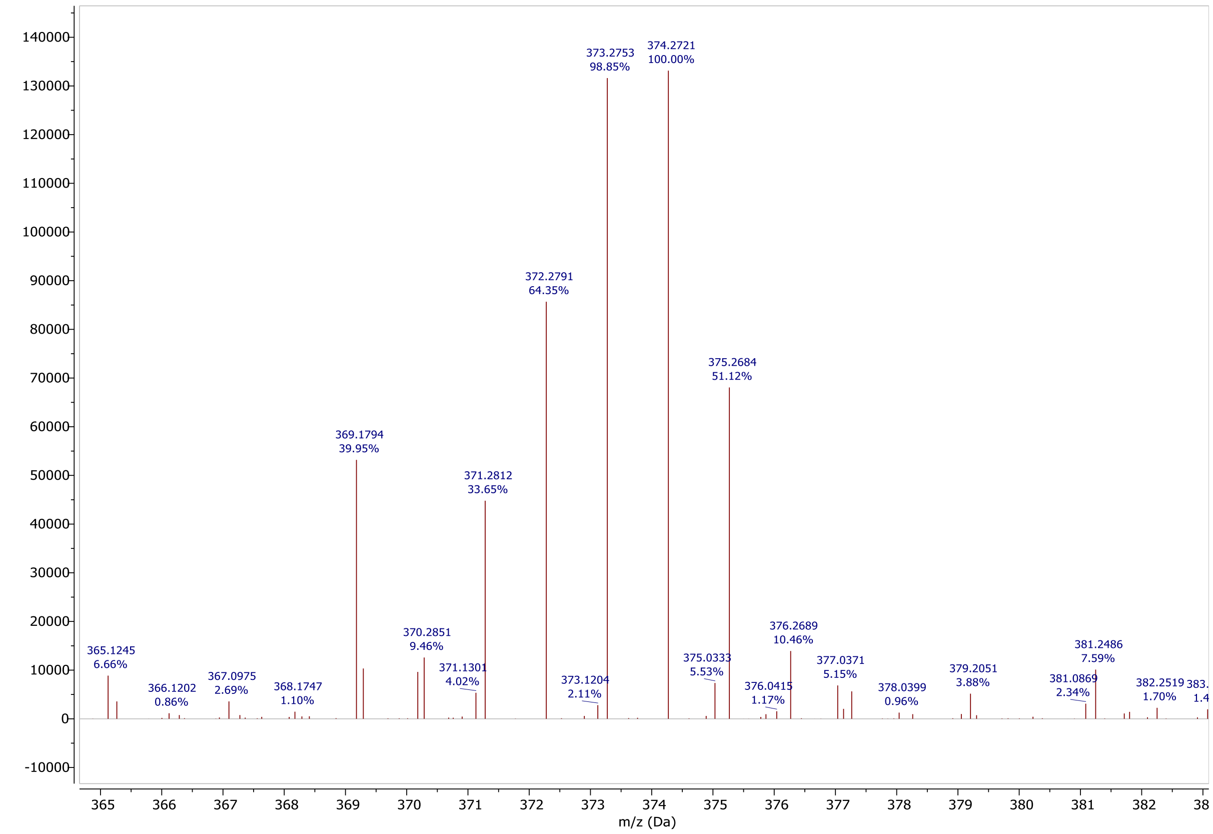

Supporting Information

Figure S57. HR(ESI+) mass spectrum of compound **LUZ5** in CH_3CN.

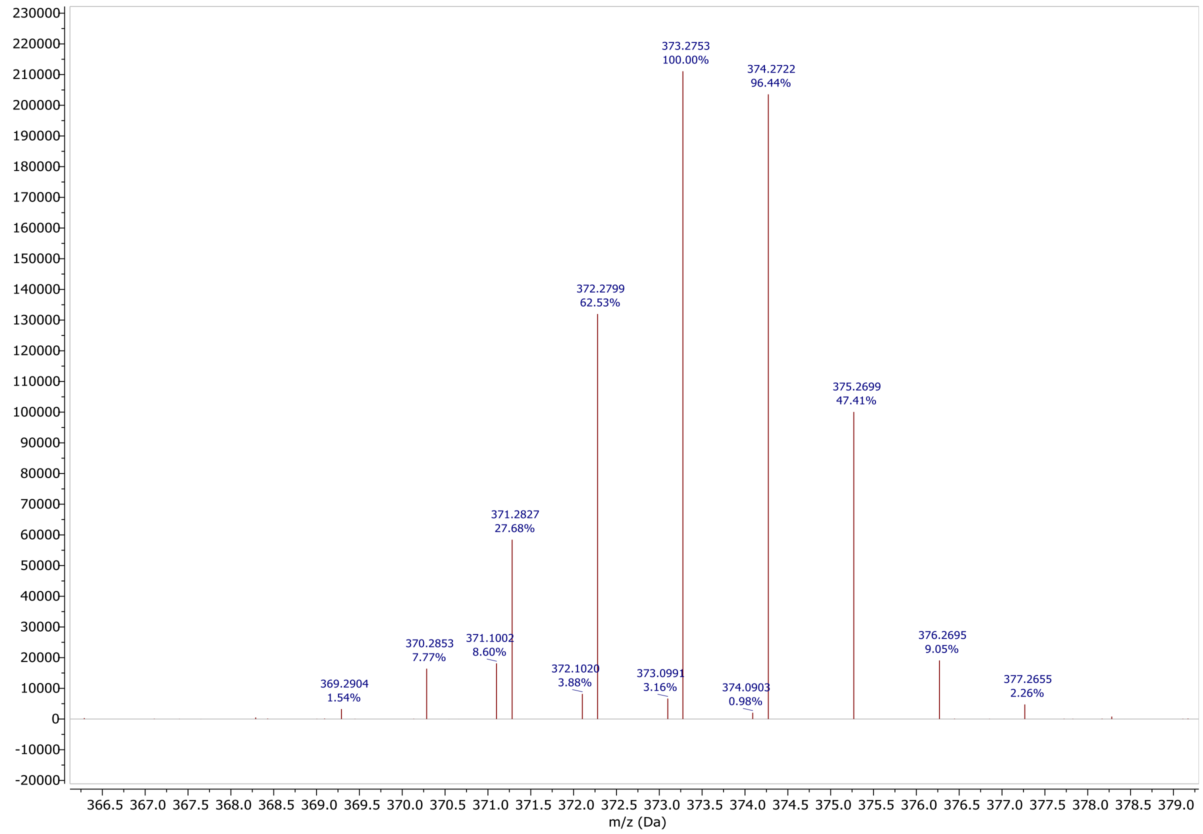

Figure S58. HR(ESI+) mass spectrum of side product **SP1** in CH_3CN.

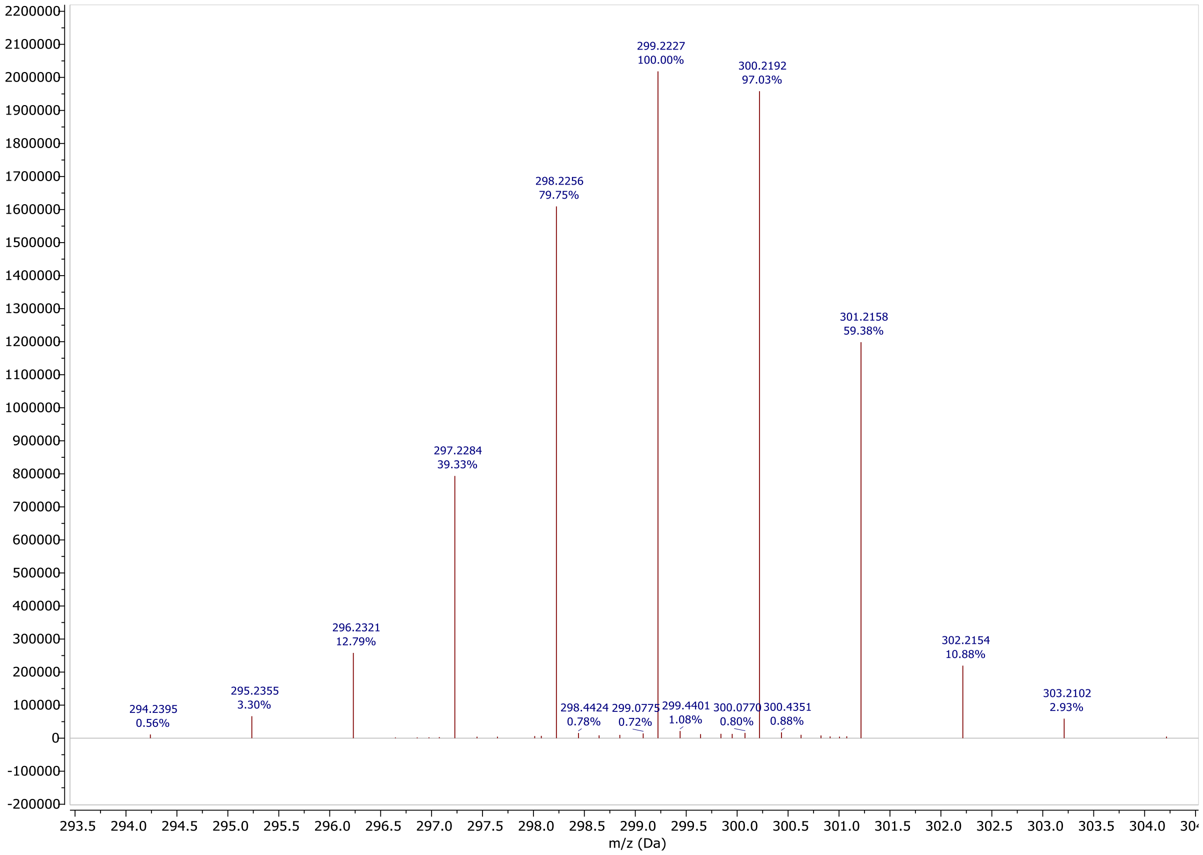

Supporting Information

Figure S59. HR(ESI+) mass spectrum of compound **17** in CH_3CN.

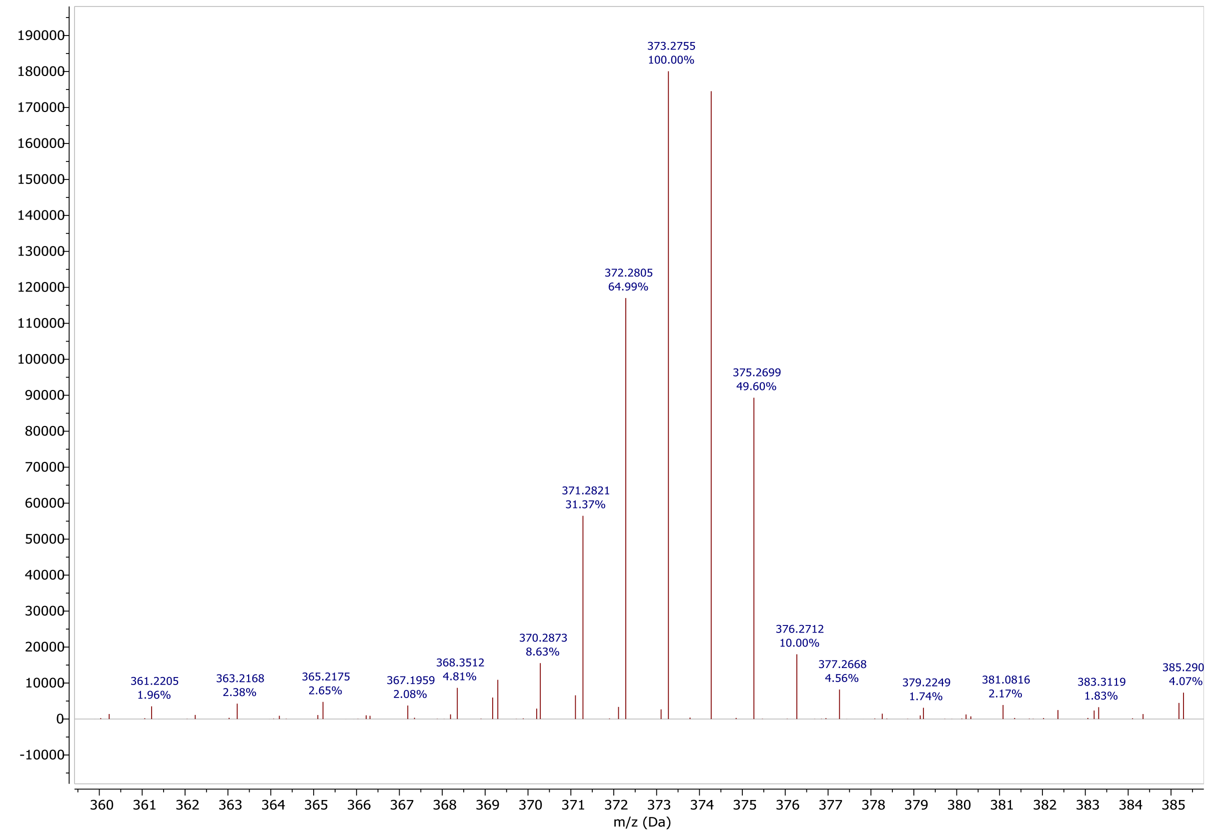

Figure S60. HR(ESI+) mass spectrum of compound **19** in CH_3CN.

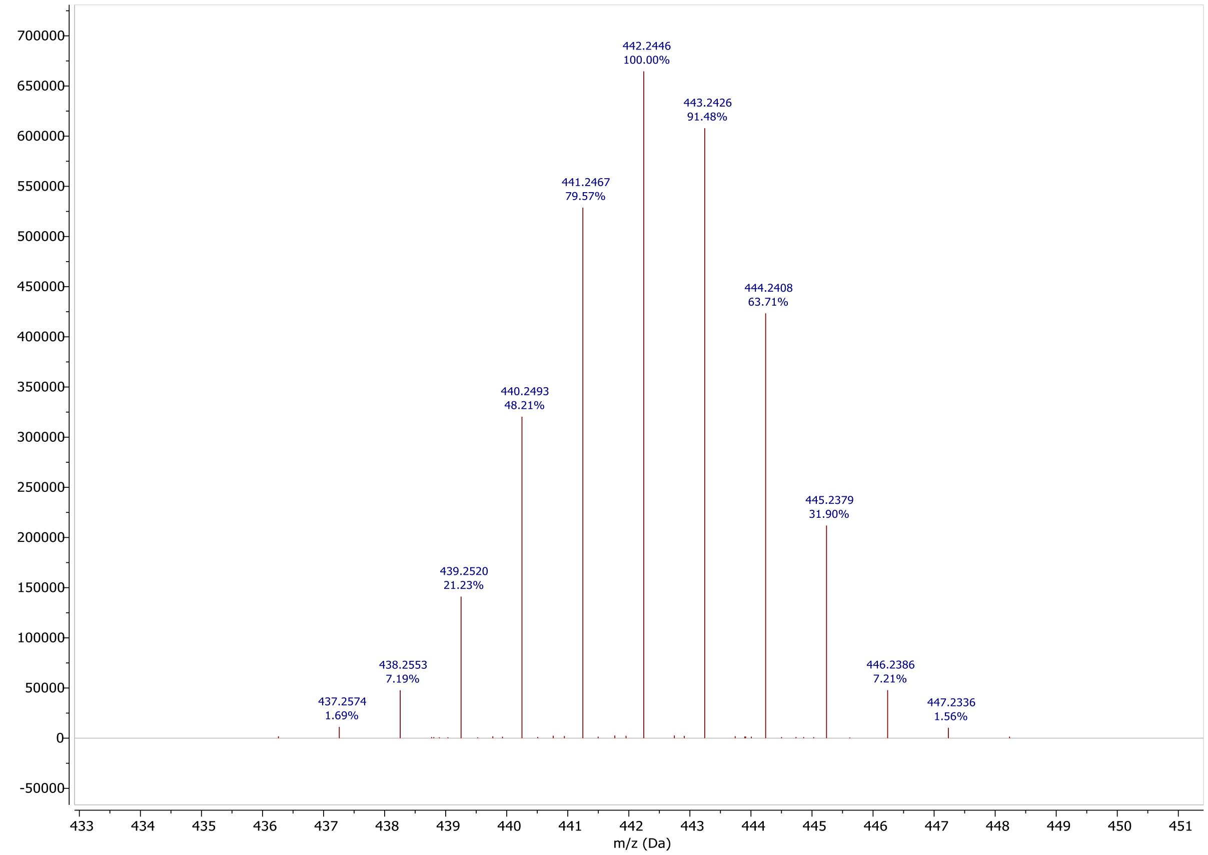

3 HPLC Purity Determination of Compounds 9, 10, 11, 12, 15 (Procedure 2), LUZ5, 17

Figure S61. HPLC trace and according values of compound **9**.

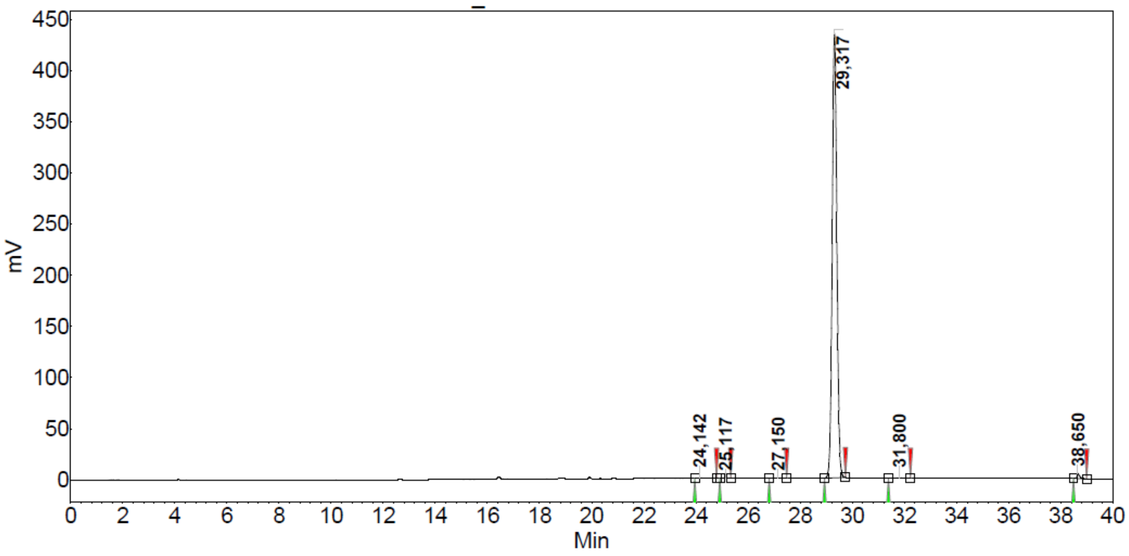

Peak results :

Index	Name	Time [Min]	Quantity [% Area]	Height [mV]	Area [mV.Min]	Area % [%]
2	UNKNOWN	24,142	0,13	0,6	0,1	0,133
3	UNKNOWN	25,117	0,04	0,2	0,0	0,039
4	UNKNOWN	27,150	0,09	0,4	0,1	0,089
1	UNKNOWN	29,317	98,99	434,3	89,8	98,994
5	UNKNOWN	31,800	0,13	0,5	0,1	0,130
6	UNKNOWN	38,650	0,62	4,9	0,6	0,616
Total			100,00	440,9	90,7	100,000

Figure S62. HPLC trace and according values of compound **10**.

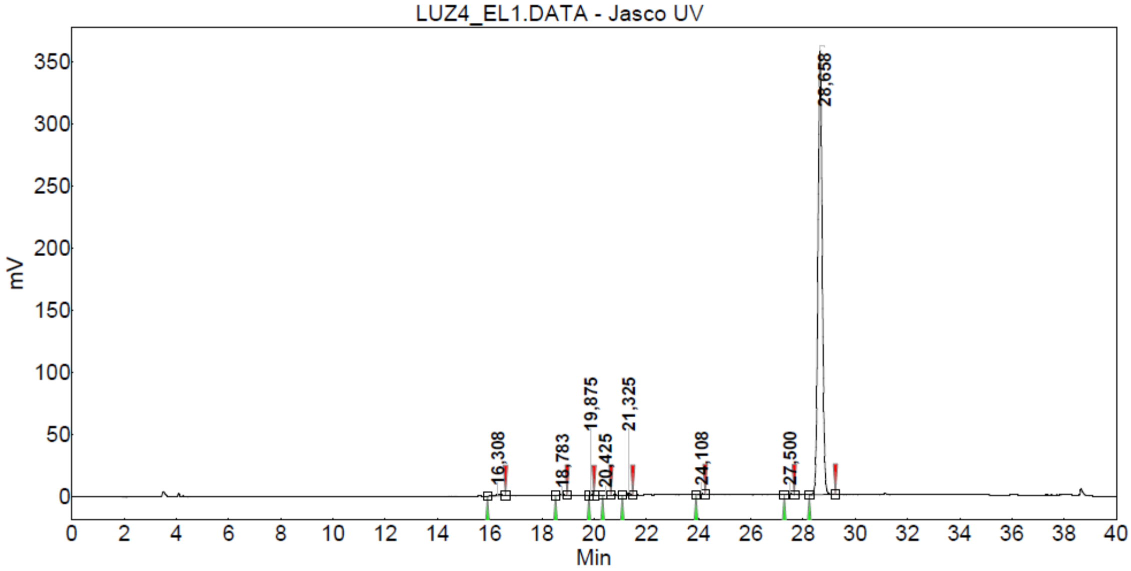

Peak results :

Index	Name	Time [Min]	Quantity [% Area]	Height [mV]	Area [mV.Min]	Area % [%]
1	UNKNOWN	16,308	0,34	1,3	0,2	0,337
4	UNKNOWN	18,783	0,13	0,5	0,1	0,130
7	UNKNOWN	19,875	0,06	0,4	0,0	0,056
8	UNKNOWN	20,425	0,05	0,2	0,0	0,048
2	UNKNOWN	21,325	0,38	2,6	0,3	0,383
3	UNKNOWN	24,108	0,17	1,0	0,1	0,169
5	UNKNOWN	27,500	0,27	1,2	0,2	0,270
6	UNKNOWN	28,658	98,61	357,5	68,1	98,607
Total			100,00	364,6	69,0	100,000

Supporting Information

Figure S63. HPLC trace and according values of compound **11**.

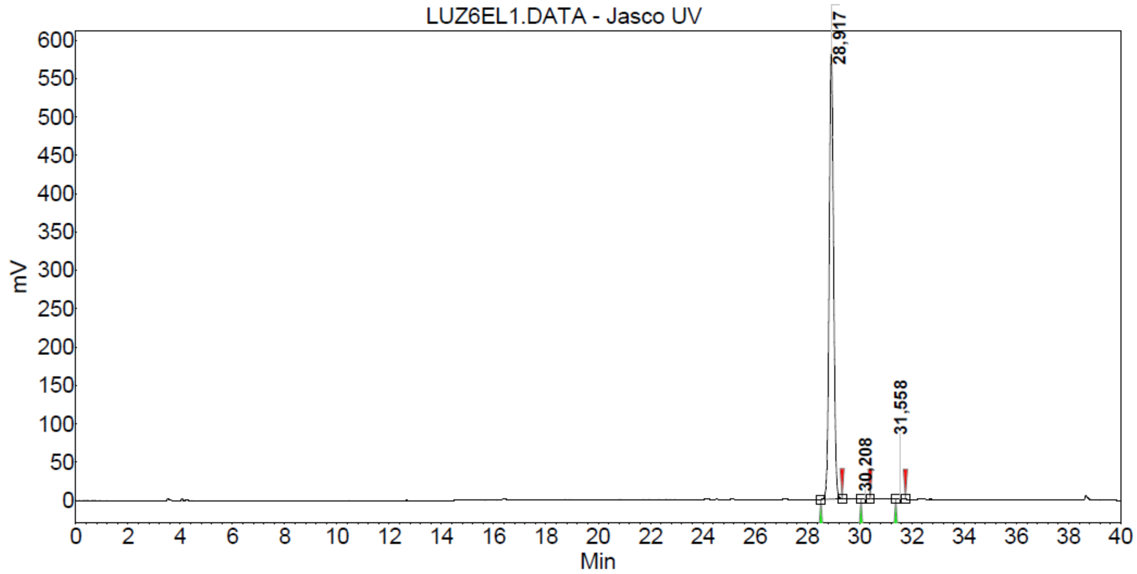

Peak results :

Index	Name	Time [Min]	Quantity [% Area]	Height [mV]	Area [mV.Min]	Area % [%]
1	UNKNOWN	28,917	99,72	581,0	113,7	99,720
2	UNKNOWN	30,208	0,12	0,8	0,1	0,119
3	UNKNOWN	31,558	0,16	0,9	0,2	0,161
Total			100,00	582,7	114,0	100,000

Figure S64. HPLC trace and according values of compound **12**.

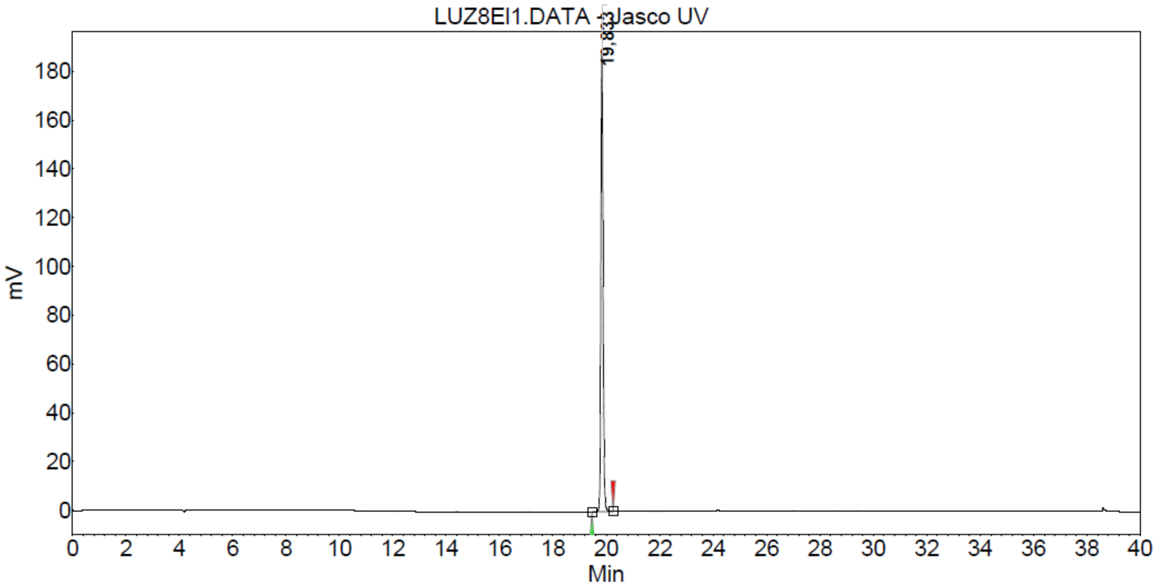

Peak results :

Index	Name	Time [Min]	Quantity [% Area]	Height [mV]	Area [mV.Min]	Area % [%]
1	UNKNOWN	19,833	100,00	187,5	17,2	100,000
Total			100,00	187,5	17,2	100,000

Figure S65. HPLC trace and according values of compound **15 (Procedure 2)**.

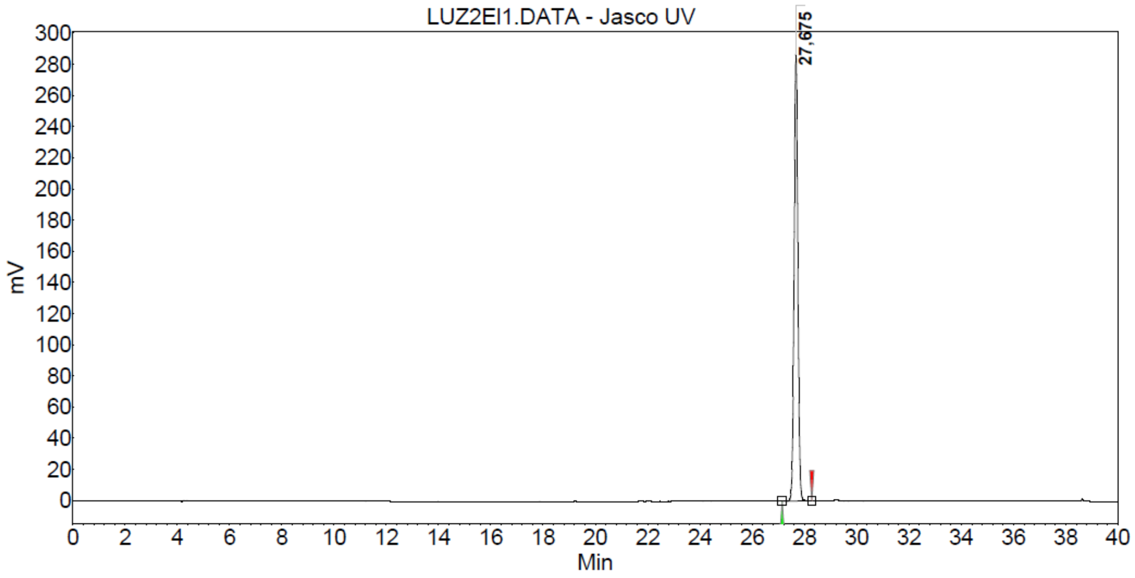

Peak results :

Index	Name	Time [Min]	Quantity [% Area]	Height [mV]	Area [mV.Min]	Area % [%]
1	UNKNOWN	27,675	100,00	287,0	47,6	100,000
Total			100,00	287,0	47,6	100,000

Figure S66. HPLC trace and according values of compound **LUZ5**.

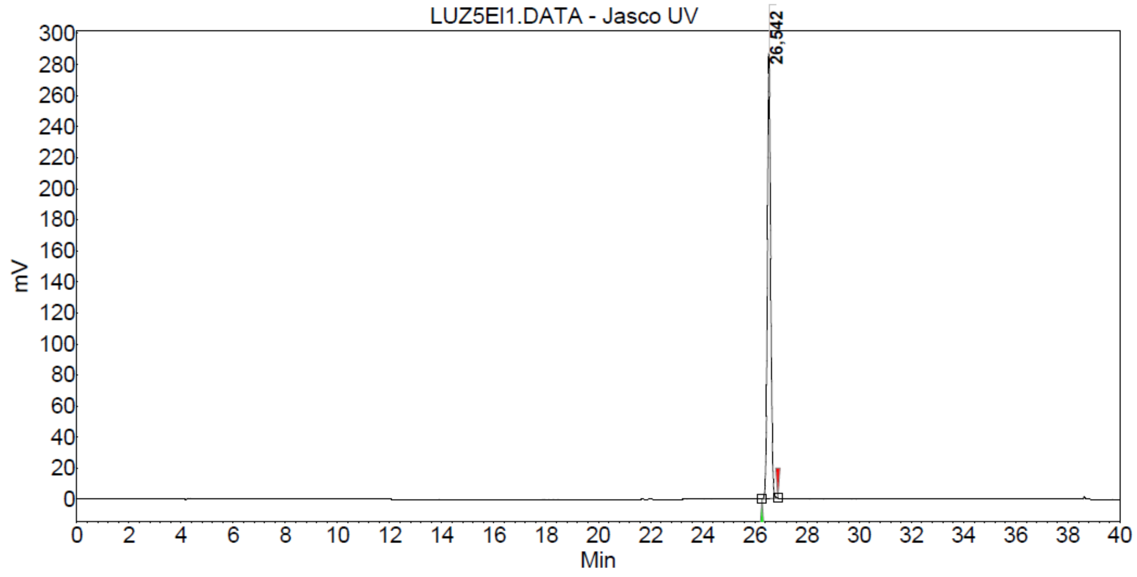

Peak results :

Index	Name	Time [Min]	Quantity [% Area]	Height [mV]	Area [mV.Min]	Area % [%]
1	UNKNOWN	26,542	100,00	287,0	41,9	100,000
Total			100,00	287,0	41,9	100,000

Figure S67. HPLC trace and according values of compound **17**.

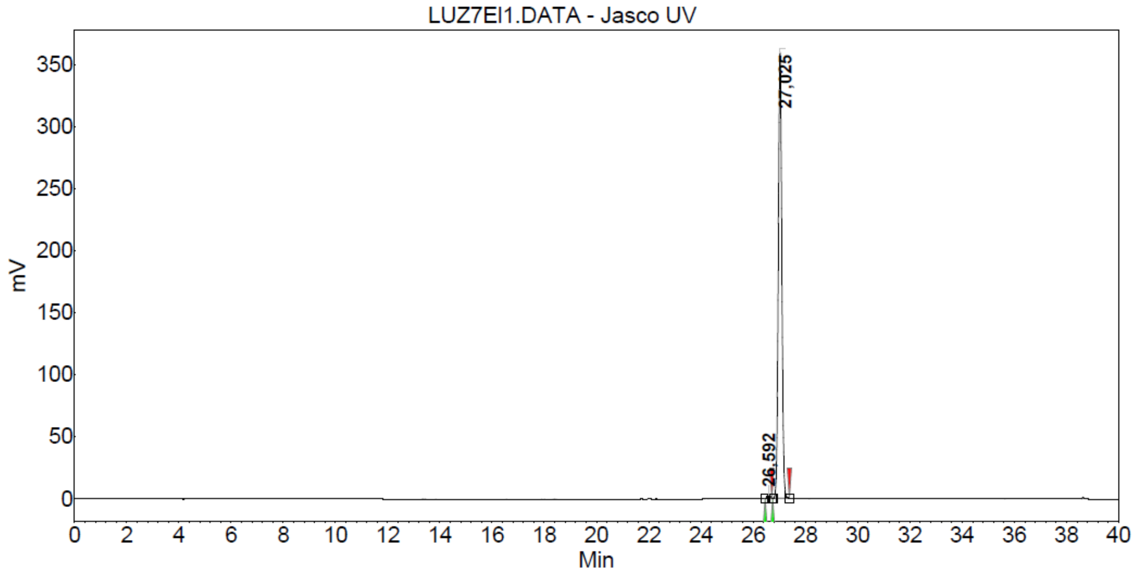

Peak results :

Index	Name	Time [Min]	Quantity [% Area]	Height [mV]	Area [mV.Min]	Area % [%]
1	UNKNOWN	26,592	0,65	2,7	0,4	0,646
2	UNKNOWN	27,025	99,35	359,6	56,1	99,354
Total			100,00	362,4	56,5	100,000

4 Stability Data of Compounds 9, 10, 11, 15 (Procedure 2), LUZ5, 17

4.1 Measurements in aq. DMSO-d_6

Figure S68. ^{1}H NMR spectra for stability determination of compound **9** in DMSO-d_6.

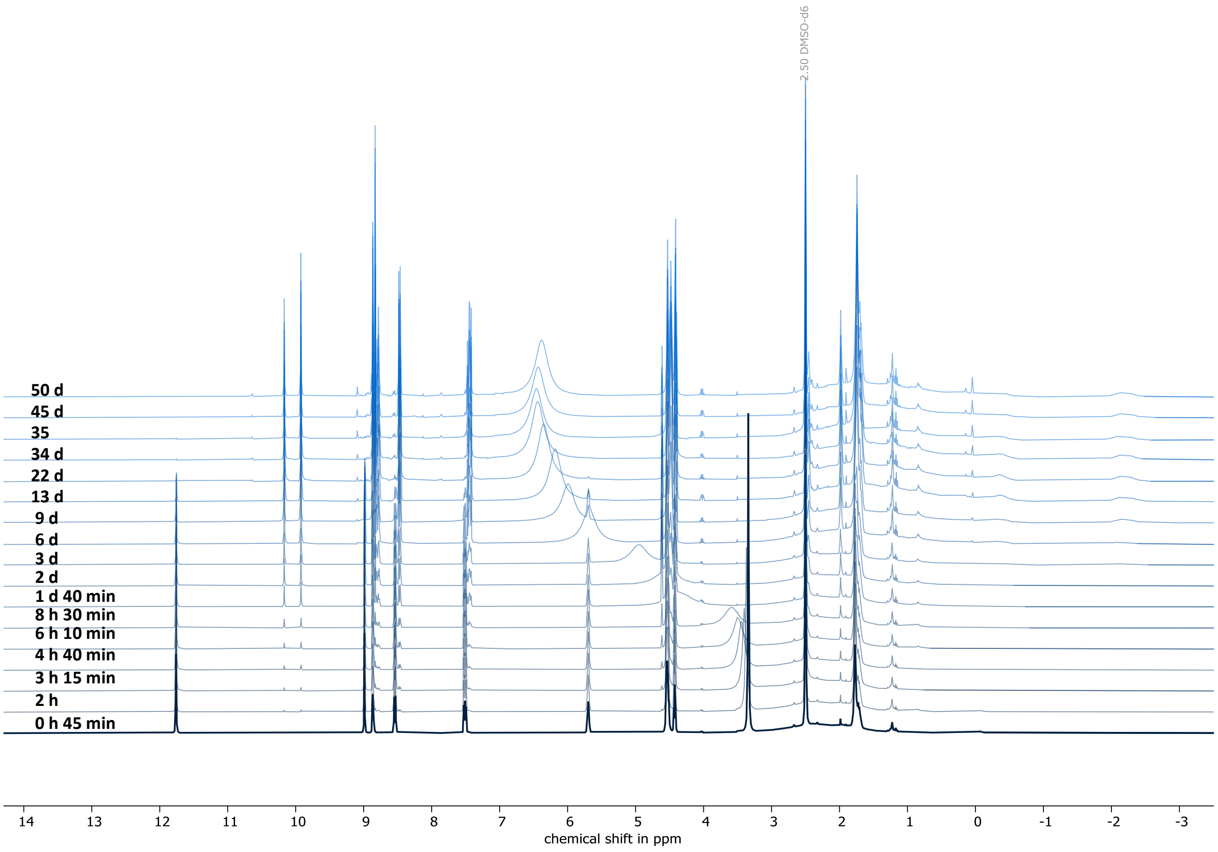

Figure S69. $^{11}B\{^1H\}$ NMR spectra for stability determination of compound **9** in DMSO-d_6.

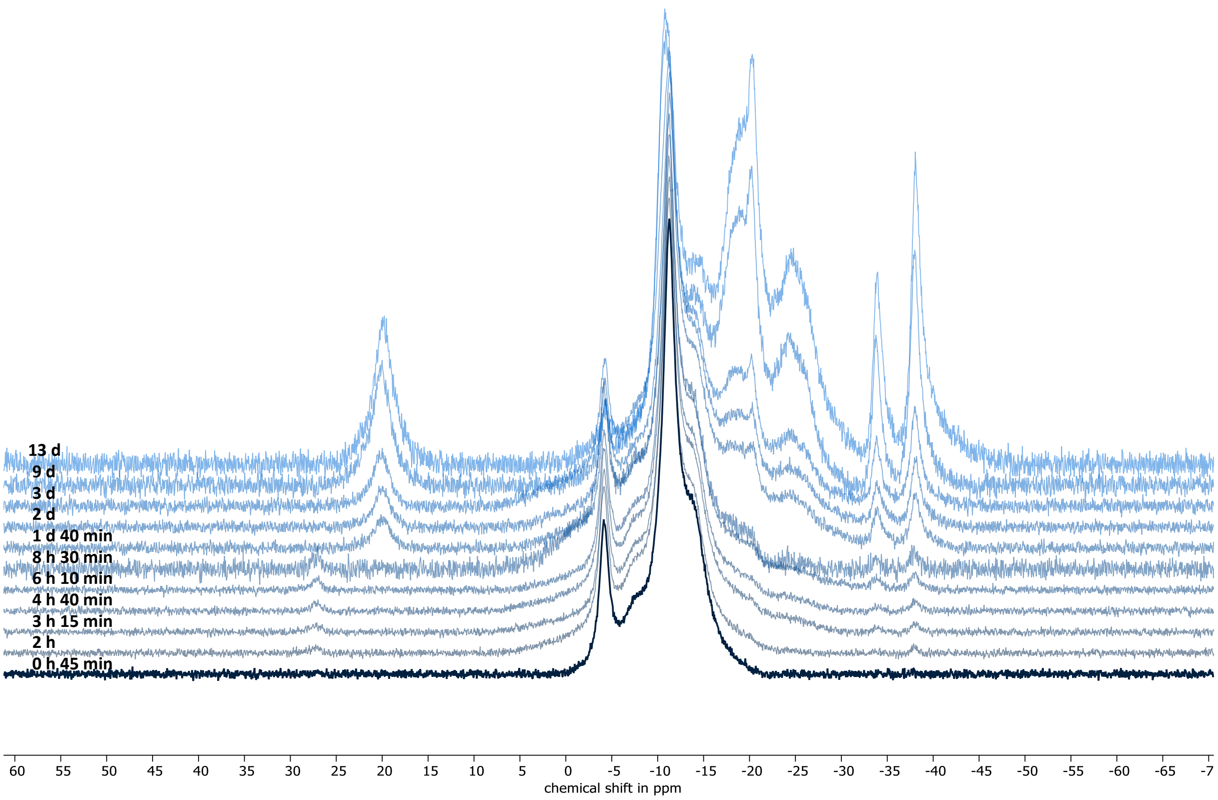

Figure S70. ^1H NMR spectra for stability determination of compound **10** in DMSO-d_6.

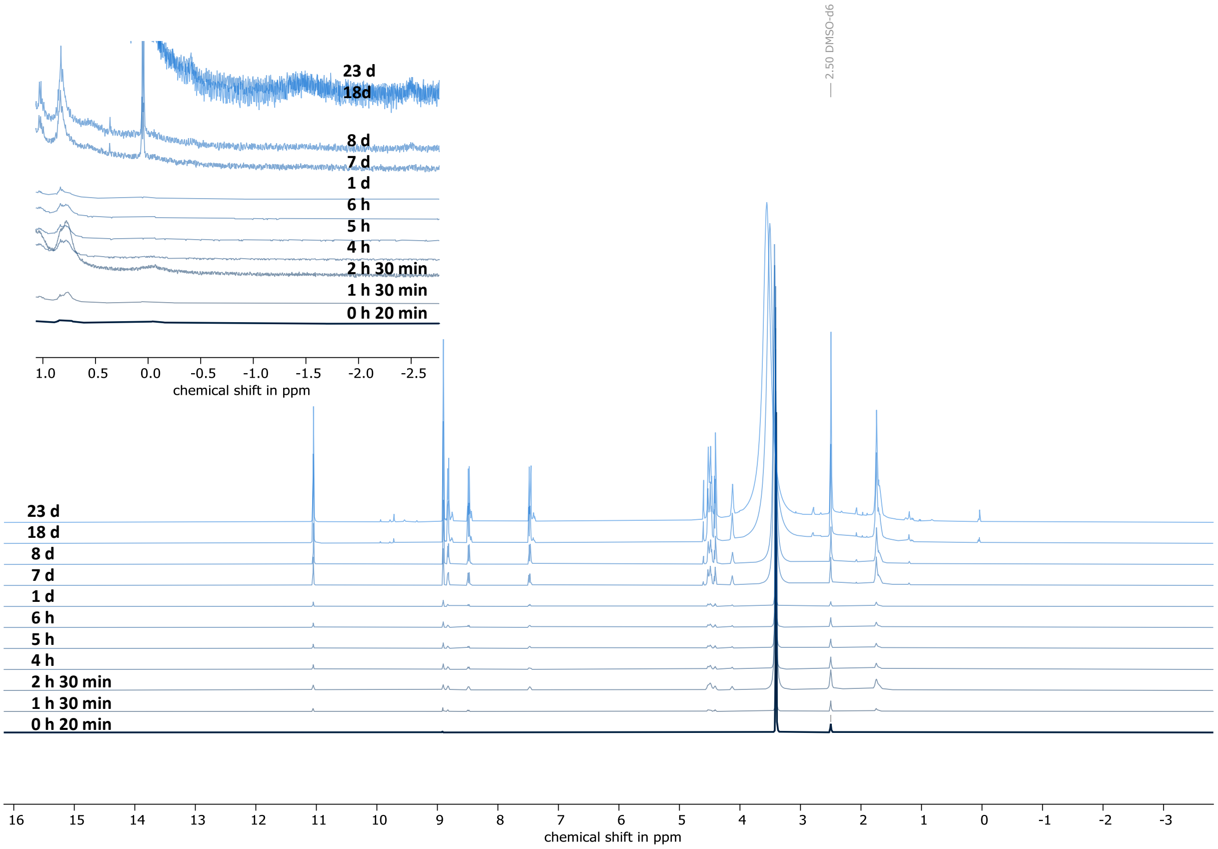

Figure S71. $^{11}B\{^{1}H\}$ NMR spectra for stability determination of compound **10** in DMSO-d_6.

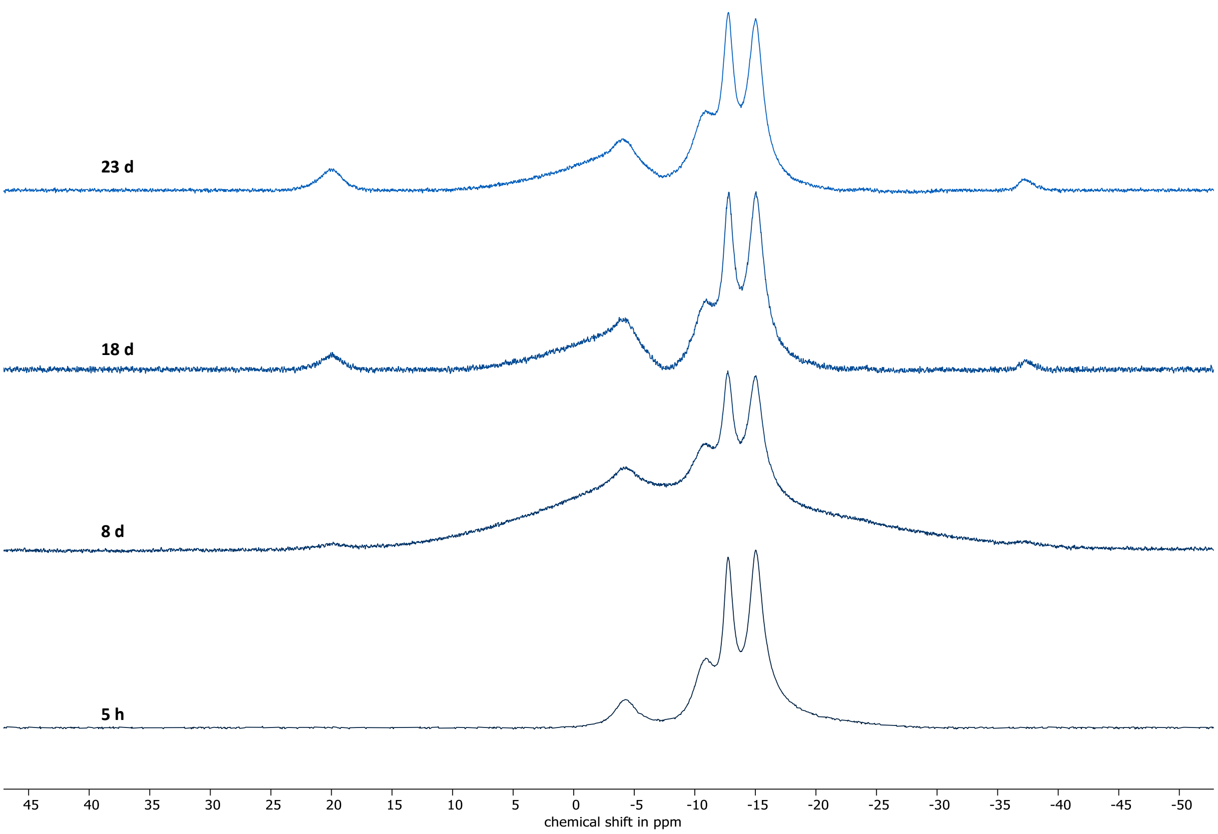

Figure S72. ^{1}H NMR spectra for stability determination of compound **11** in DMSO-d_6.

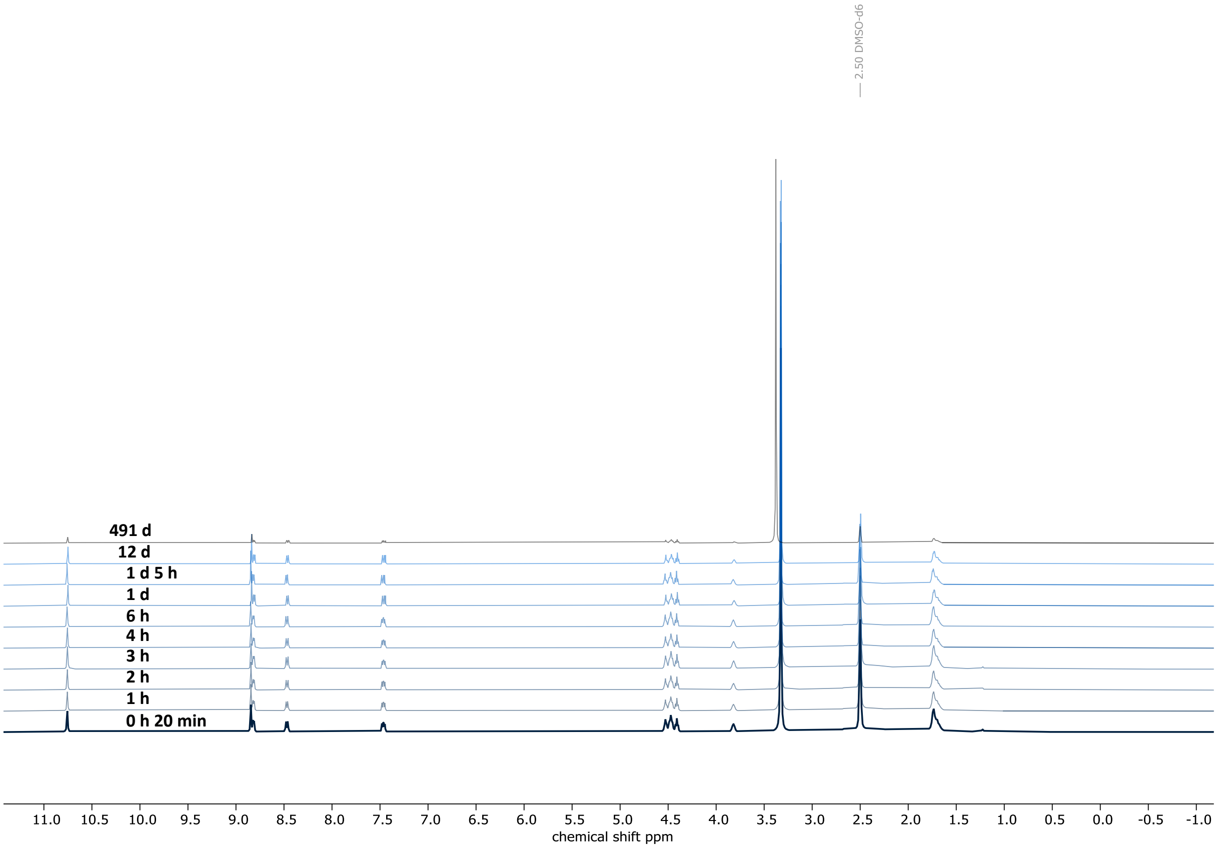

Figure S73. $^{11}B\{^{1}H\}$ NMR spectra for stability determination of compound **11** in DMSO-d_6.

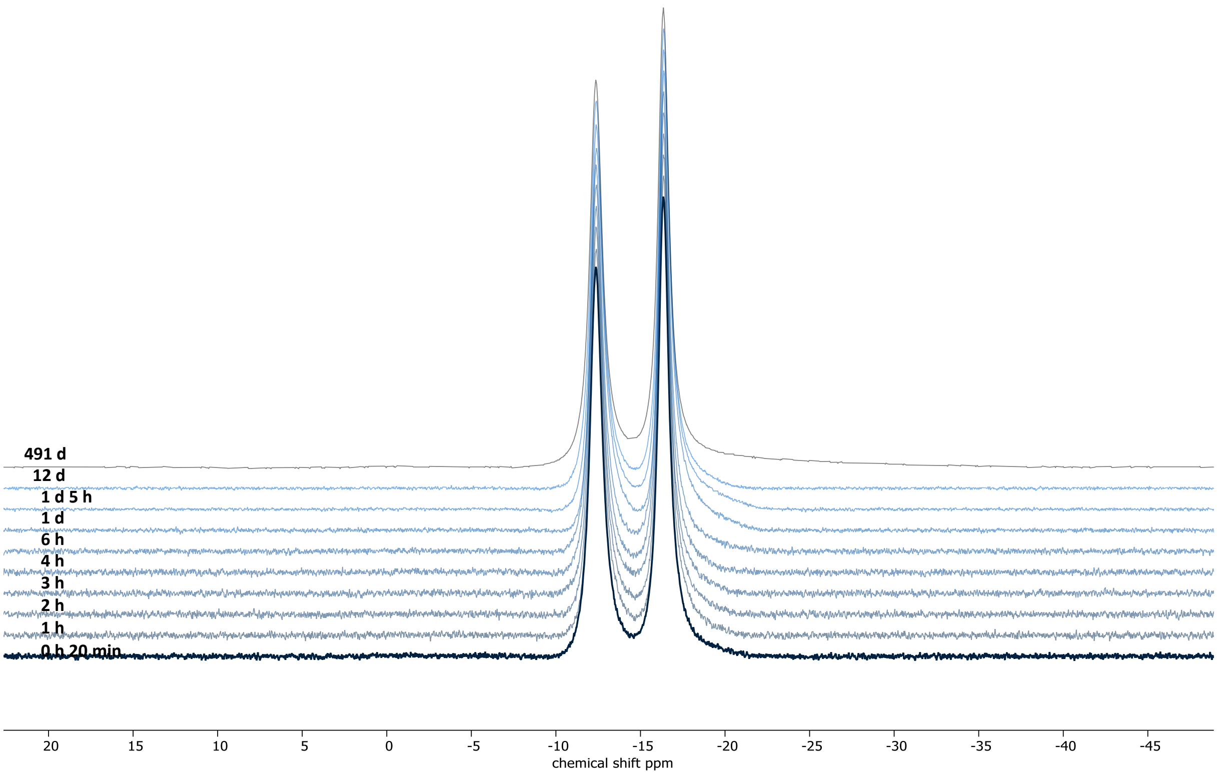

Figure S74. ^{1}H NMR spectra for stability determination of compound **15 (Procedure 2)** in DMSO-d_6.

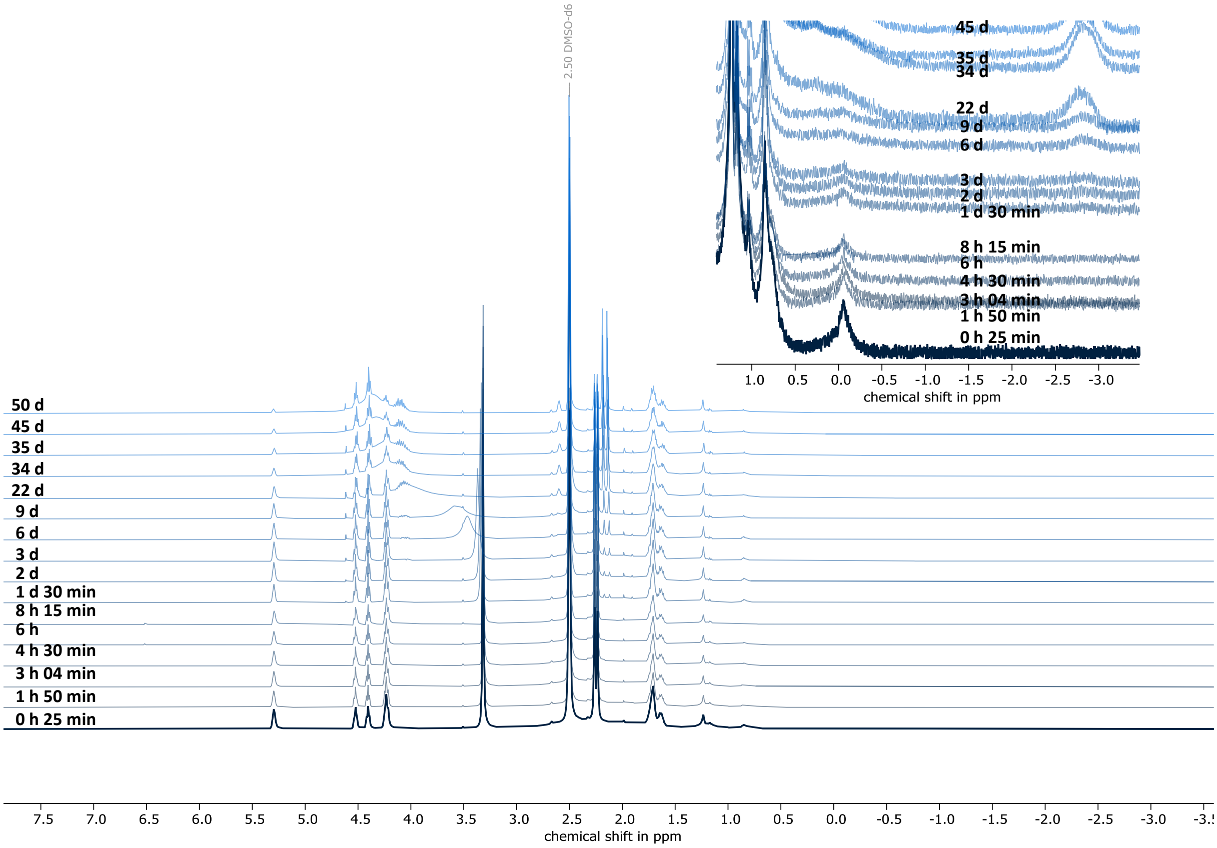

Figure S75. $^{11}B\{^{1}H\}$ NMR spectra for stability determination of compound **15 (Procedure 2)** in DMSO-d_6.

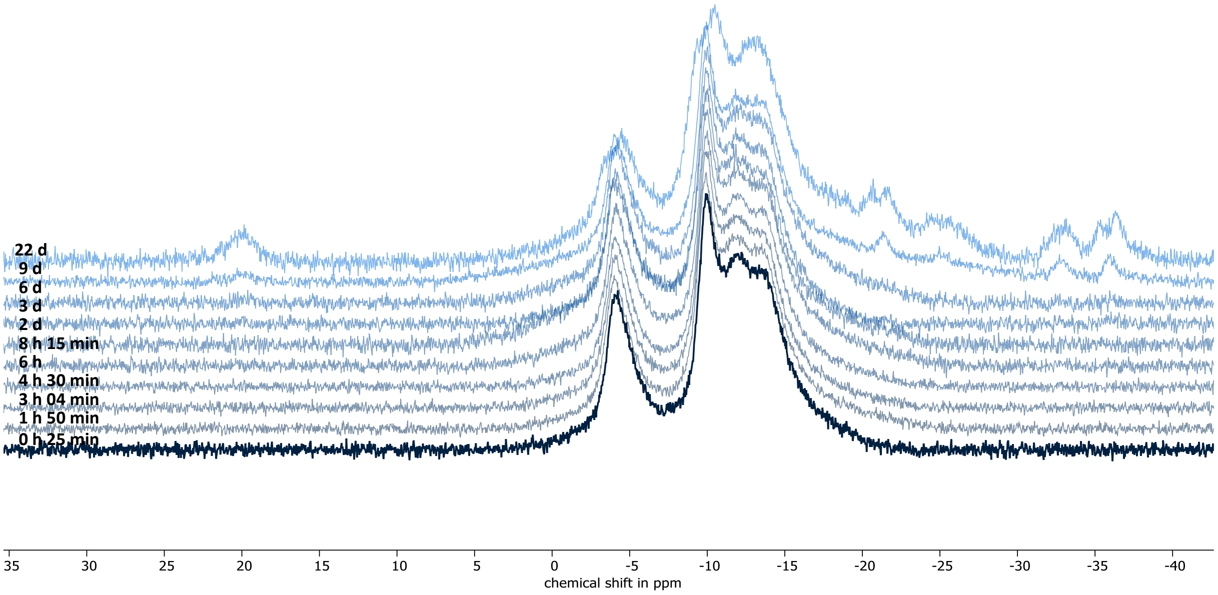

Figure S76. ^{1}H NMR spectra for stability determination of compound **LUZ5** in DMSO-d_6.

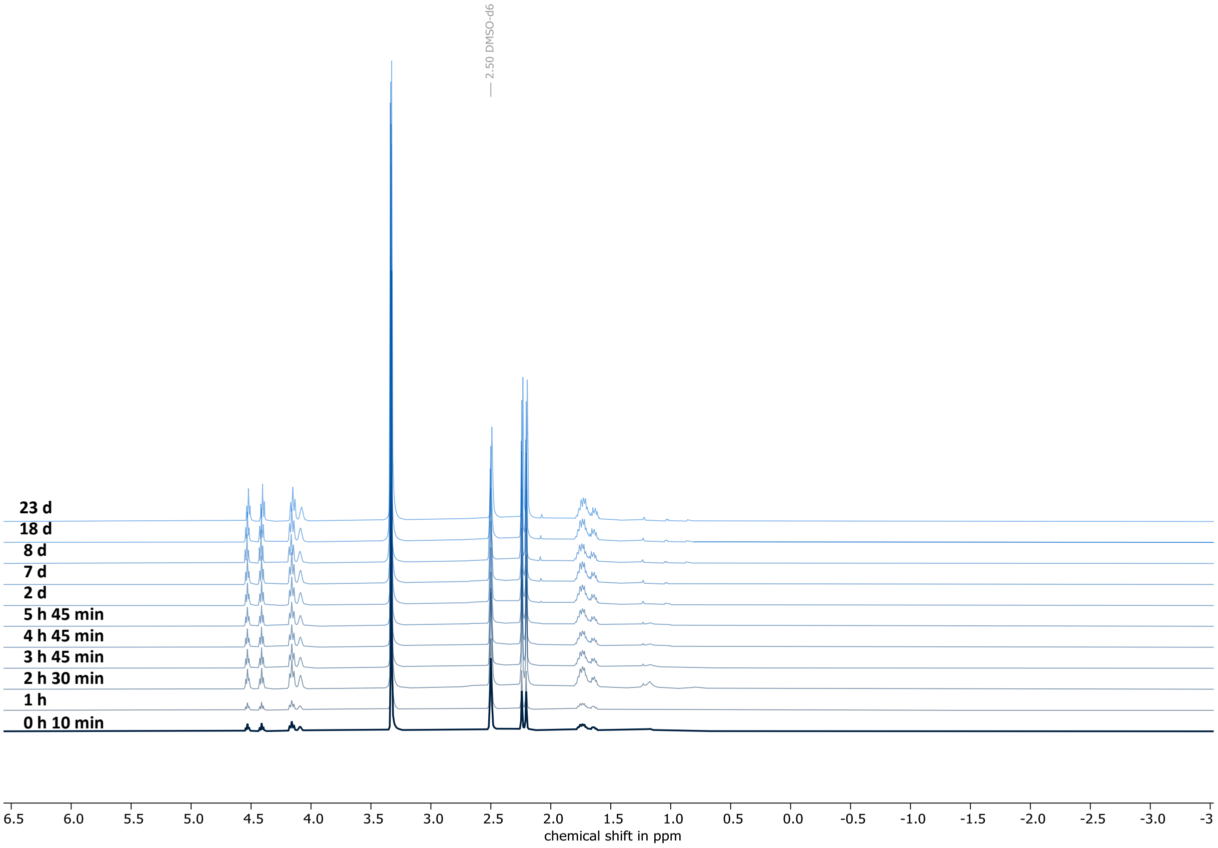

Figure S77. $^{11}B\{^{1}H\}$ NMR spectra for stability determination of compound **LUZ5** in DMSO-d_6.

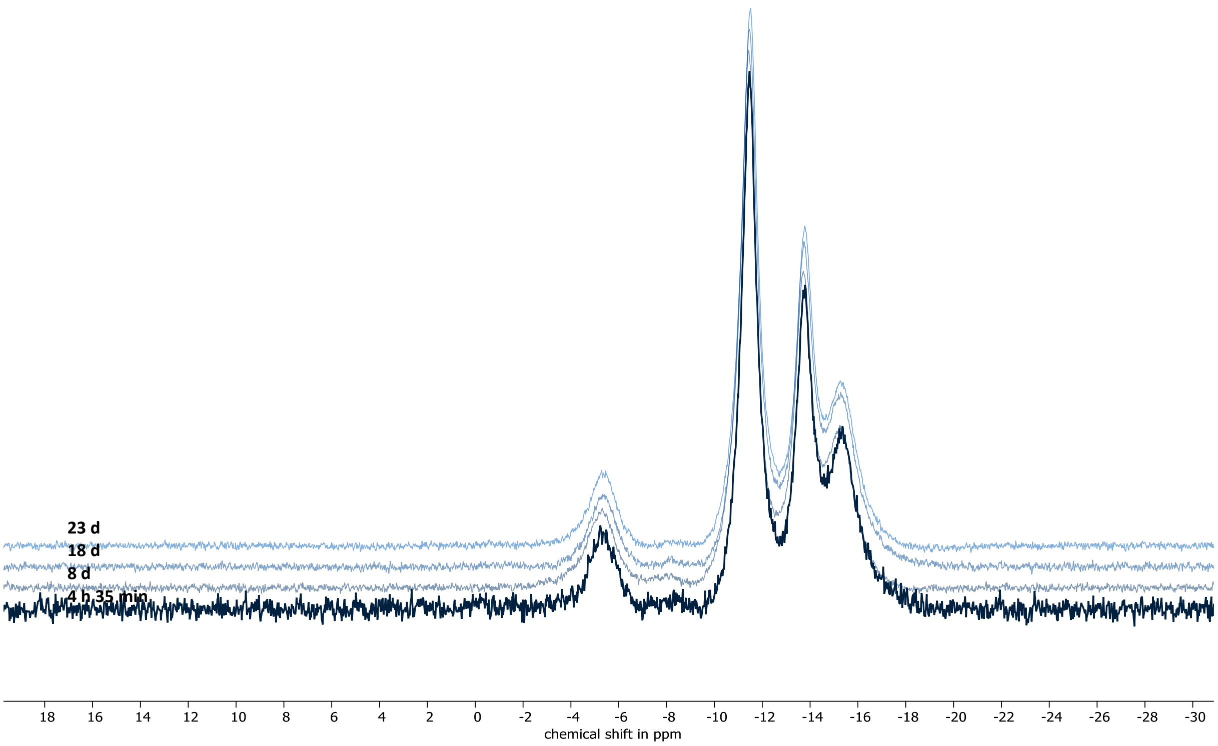

Figure S78. ^{1}H NMR spectra for stability determination of compound **17** in DMSO-d_6.

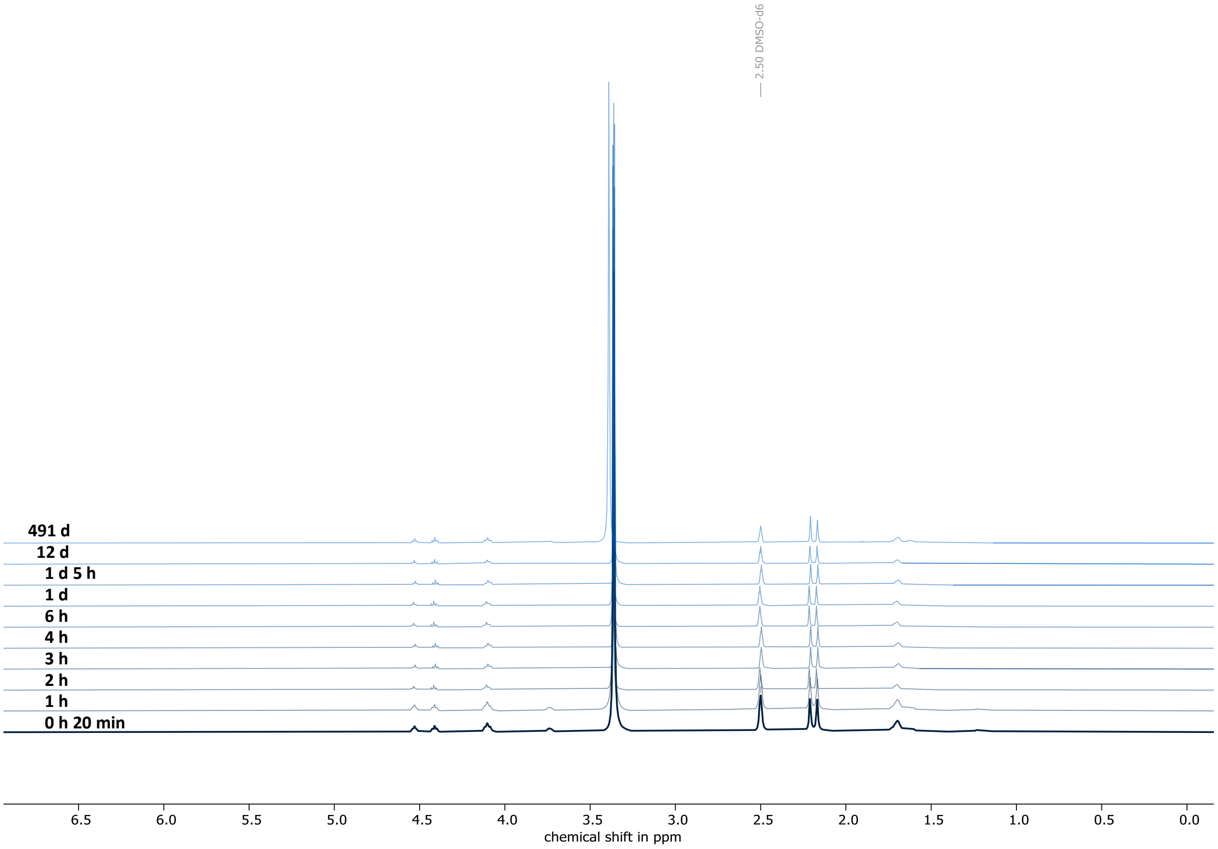

Figure S79. $^{11}B\{^{1}H\}$ NMR spectra for stability determination of compound **17** in DMSO-d_6.

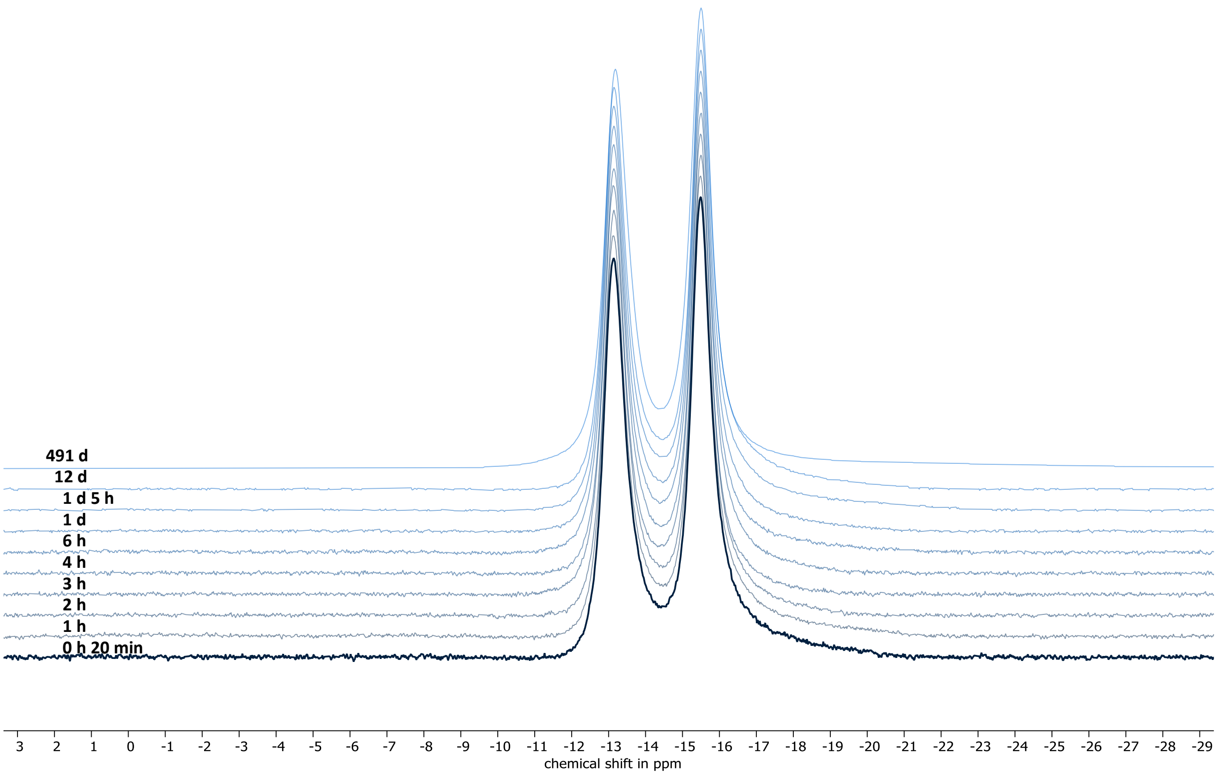

4.2 Measurements of LUZ5 in aq. DMSO-d_6 with increased H_2O/ D_2O amount

Figure S80. $^{11}B\{^{1}H\}$ NMR spectra for stability determination of compound **LUZ5** in DMSO-d_6 with increased amount of H_2O (0.1 mL H_2O and 0.5 mL DMSO-d_6).

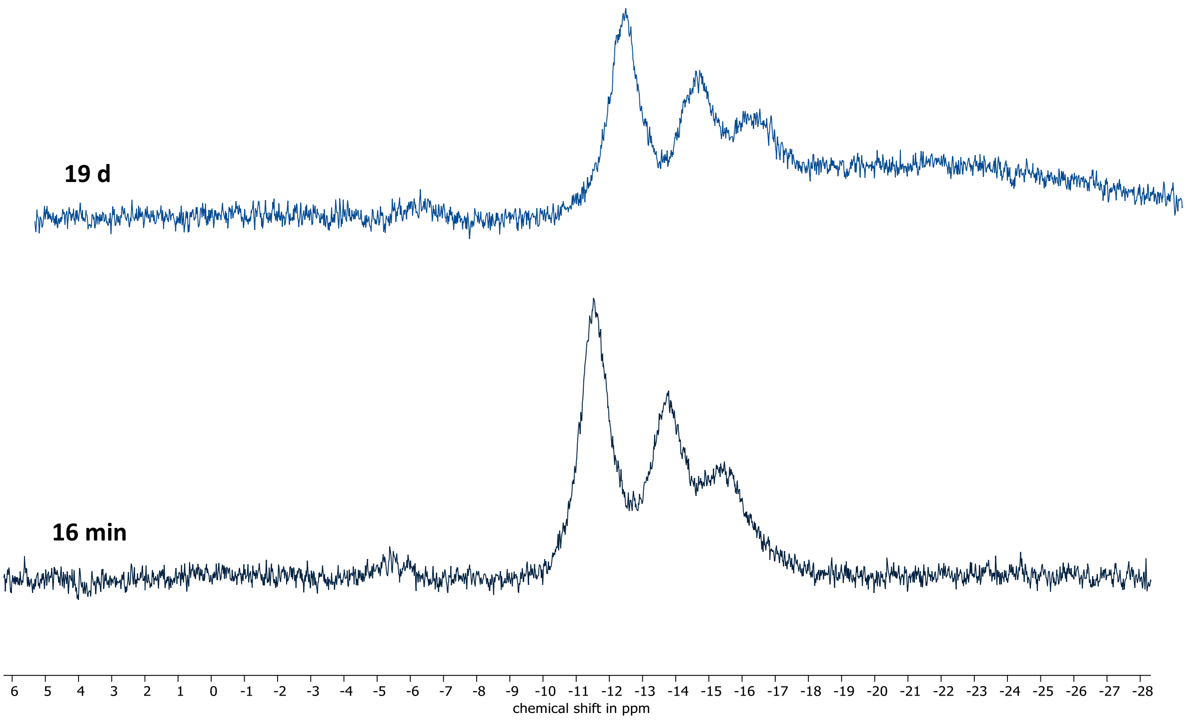

Figure S81. $^{11}B\{^{1}H\}$ NMR spectra for stability determination of compound **LUZ5** in DMSO-d_6 with increased amount of D_2O (0.1 mL D_2O and 0.5 mL DMSO-d_6).

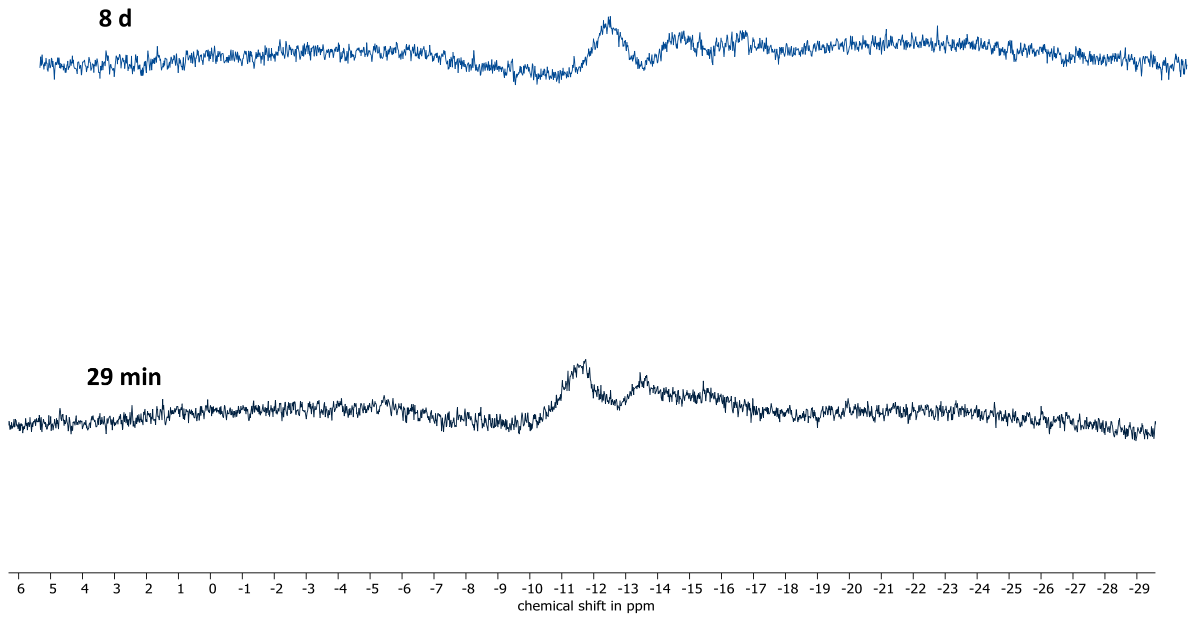

5 X-ray Crystallography Data of Compounds 9, 10, 11, 15 (Procedure 2), LUZ5, 17 and Molecular Structures of 11, 17

The data were collected on a Gemini diffractometer (Rigaku Oxford Diffraction) using Mo-Kα or Cu-Kα radiation and ω-scan rotation. Data reduction was performed with CrysAlisPro[1] including the program SCALE3 ABSPACK[2] for empirical absorption correction. All structures were solved by dual space methods with SHELXT[3] and the refinement was performed with SHELXL[4]. Except the minor 19 % disordered part of compound **9**, all non-hydrogen atoms were refined with anisotropic displacement parameters. Hydrogen atoms for **LUZ5** and for disordered fragments of a structure were calculated on idealized positions using the riding model, whereas for all other cases a difference-density Fourier map was used to locate hydrogen atoms. Especially the *n*-butylfluoro substituents of these compounds are prone for disorder (**11**, **LUZ5**), and for **9** even the whole carborane substituent is affected. Only compounds **10**, **15** and **17** are not disordered. With the exception of **LUZ5** all carborane carbon atoms could be localized with a bond length and displacement parameter analysis. For **LUZ5** the carborane carbon atom C2 is most likely disordered over the entire CB_4 ring. As a result of a phase transition of **LUZ5** below room temperature, the data had to been recorded at 293 K to avoid twinning. Structure figures were generated with DIAMOND-4[5].

CCDC deposition numbers given in Table S1 contains the supplementary crystallographic data for this paper. These data can be obtained free of charge via https://summary.ccdc.cam.ac.uk/structure-

summary-form (or from the Cambridge Crystallographic Data Centre, 12 Union Road, Cambridge CB2 1EZ, UK; fax: (+44)1223-336-033; or deposit@ccdc.cam.uk).

Table S1. Fundamental structure parameters of **9**, **10**, **11**, **15**, **LUZ5**, **17**.

Compound	**9**	**10**	**11**
Empirical formula	$C_{15}H_{24}B_{10}FN_3O_2$	$C_{15}H_{24}B_{10}FN_3O_2$	$C_{15}H_{24}B_{10}FN_3O_2$
Formula weight	405.47	405.47	405.47
Temperature [K]	130(2)	130(2)	130(2)
Wavelength [pm]	71.073	71.073	71.073
Crystal system	Monoclinic	Triclinic	Monoclinic
Space group	P 2_1/n	$P\bar{1}$	I 2/a
Unit cell dimensions			
a [pm]	1533.36(8)	694.99(2)	2266.67(7)
b [pm]	732.47(3)	1223.43(5)	749.77(2)
c [pm]	1889.73(9)	1301.66(5)	2448.45(7)
α [deg]	90	109.825(4)	90
β [deg]	105.003(5)	94.822(3)	97.632(3)
γ [deg]	90	92.968(3)	90
Volume [nm^3]	2.0501(2)	1.03367(7)	4.1242(2)
Z	4	2	8
$\rho_{(calculated)}$ [Mg/m^3]	1.314	1.303	1.306
μ [mm^{-1}]	0.084	0.083	0.083
F(000)	840	420	1680
Crystal size [mm^3]	0.40 x 0.03 x 0.02	0.44 x 0.37 x 0.09	0.45 x 0.15 x 0.10
Θ_{Min} / Θ_{Max} [deg]	1.982 / 28.262	2.830 / 32.473	2.301 / 28.237
Index ranges	-20 ≤ h ≤ 19<br>-9 ≤ k ≤ 9<br>-22 ≤ l ≤ 24	-9 ≤ h ≤ 10<br>-18 ≤ k ≤ 18<br>-19 ≤ l ≤ 18	-29 ≤ h ≤ 29<br>-9 ≤ k ≤ 9<br>-31 ≤ l ≤ 30
Reflections collected	17036	21330	23757
Indp. reflections (R_{int})	4476 (0.0618)	6887 (0.0338)	4617 (0.0466)
Completeness (Θ_{Max})	100.0 % (25.35)	100.0 % (30.51)	99.9 % (25.35)
T_{Max} / T_{Min}	1.00000 / 0.71503	1.00000 / 0.99334	1.00000 / 0.94685

Restraints / parameters	80 / 401	0 / 376	14 / 372
Gof on F^2	1.011	1.024	1.016
R1 / wR2 (I>2σ(I))	0.0565 / 0.1171	0.0489 / 0.1261	0.0557 / 0.1236
R1 / wR2 (all data)	0.1122 / 0.1427	0.0640 / 0.1371	0.0934 / 0.1453
Residual electron density [e·Å^{-3}]	0.203 / -0.250	0.467 / -0.242	0.377 / -0.368
CCDC No	2232528	2232530	2232532

Table S1: continued

Compound	**15**	**16 (LUZ5)**	**17**
Empirical formula	$C_{12}H_{25}B_{10}FN_2OS$	$C_{12}H_{25}B_{10}FN_2OS$	$C_{12}H_{25}B_{10}FN_2OS$
Formula weight	372.50	372.50	372.50
Temperature [K]	130(2)	293(2)	130(2)
Wavelength [pm]	71.073	71.073	154.184
Crystal system	Monoclinic	Monoclinic	Triclinic
Space group	P 2_1/n	P 2_1/n	P $\overline{1}$
Unit cell dimensions			
a [pm]	720.38(2)	1325.67(4)	689.18(2)
b [pm]	2421.49(6)	742.99(2)	1043.96(3)
c [pm]	1095.08(3)	2061.05(6)	1405.92(3)
α [deg]	90	90	99.793(2)
β [deg]	93.956(2)	104.120(3)	100.469(2)
γ [deg]	90	90	92.309(2)
Volume [nm^3]	1.90570(9)	1.9687(1)	0.97754(5)
Z	4	4	2
$\rho_{(calculated)}$ [Mg/m^3]	1.298	1.257	1.266
μ [mm^{-1}]	0.184	0.178	1.561
F(000)	776	776	388
Crystal size [mm^3]	0.30 x 0.25 x 0.05	0.40 x 0.13 x 0.09	0.42 x 0.15 x 0.09
Θ_{Min} / Θ_{Max} [deg]	1.682 / 30.479	2.925 / 28.424	3.250 / 67.974
Index ranges	-10 ≤ h ≤ 10<br>-32 ≤ k ≤ 34	-17 ≤ h ≤ 17<br>-9 ≤ k ≤ 9	-6 ≤ h ≤ 8<br>-12 ≤ k ≤ 12

	-15 ≤ l ≤ 15	-26 ≤ l ≤ 26	-16 ≤ l ≤ 16
Reflections collected	18117	22093	15966
Indp. reflections (R_{int})	5284 (0.0509)	4464 (0.0372)	3551 (0.0256)
Completeness (Θ_{Max})	100.0 % (28.29)	99.9 % (25.35)	99.4 % (67.97)
T_{Max} / T_{Min}	1.00000 / 0.96008	1.00000 / 0.99604	1.00000 / 0.83477
Restraints / parameters	6 / 344	71 / 284	0 / 344
Gof on F^2	1.023	1.021	1.047
R1 / wR2 (I>2σ(I))	0.0493 / 0.1071	0.0501 / 0.1229	0.0368 / 0.0991
R1 / wR2 (all data)	0.0751 / 0.1206	0.0861 / 0.1427	0.0388 / 0.1015
Residual electron density [e·Å^{-3}]	0.272 / -0.329	0.191 / -0.236	0.338 / -0.283
CCDC No	2232529	2232531	2232533

Figure S82. Molecular Structure of **11** (disordered atoms not shown).

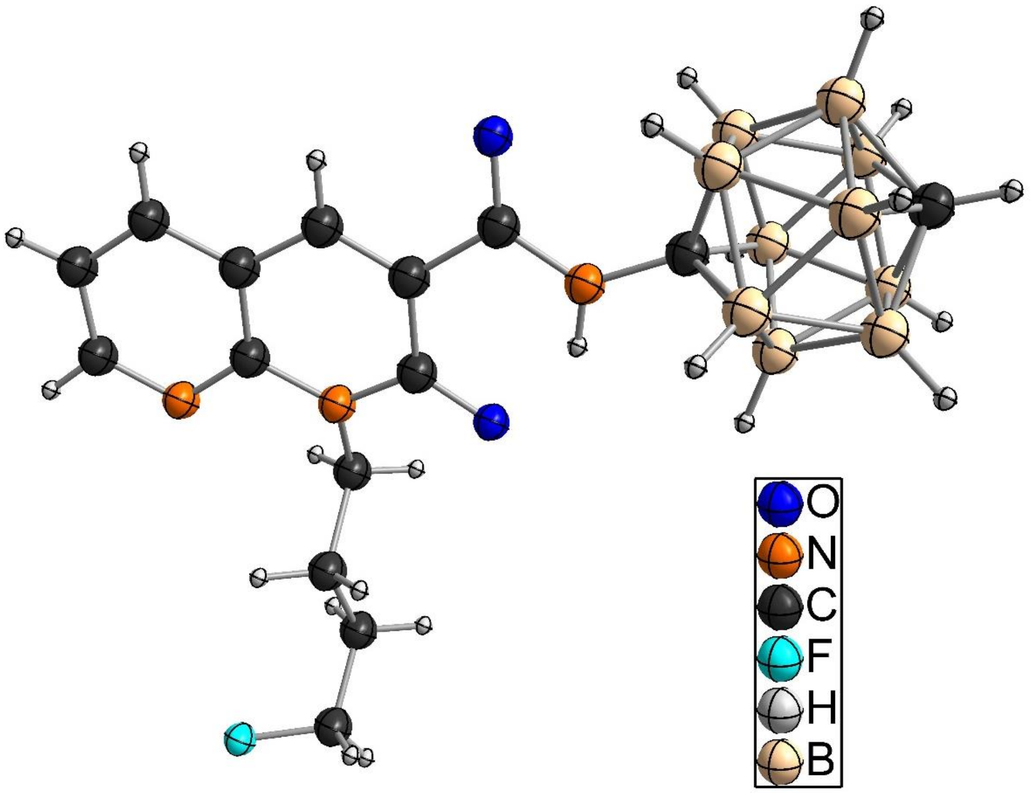

Figure S83. Molecular Structure of **17**.

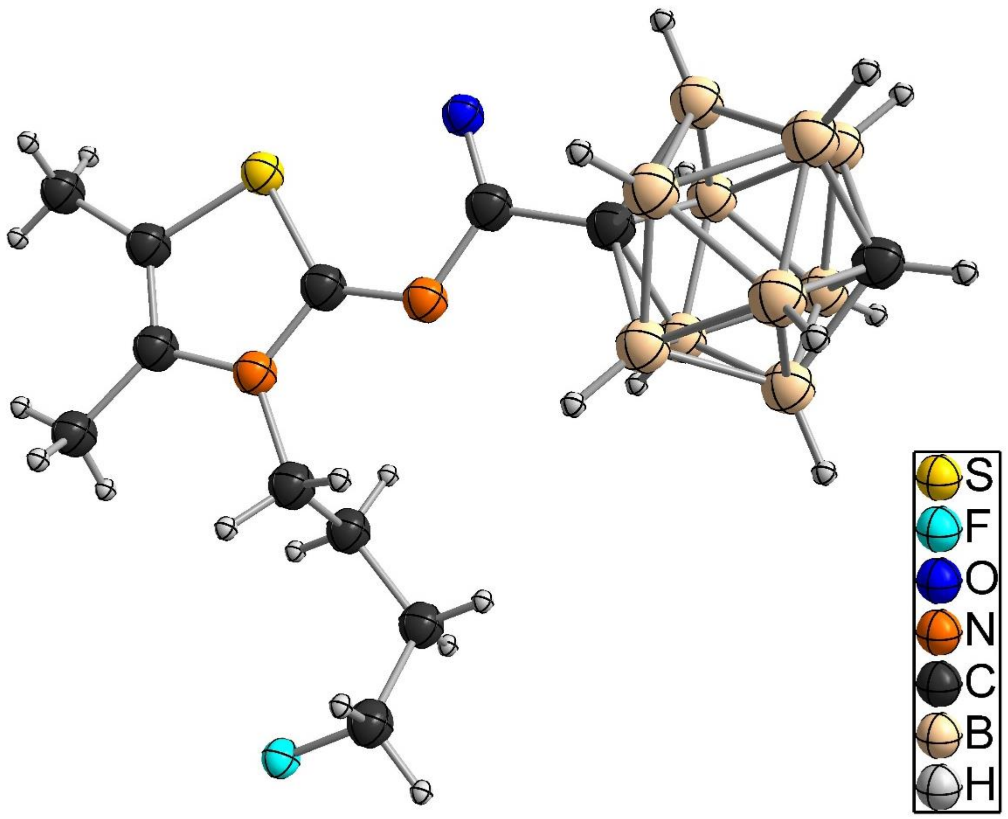

6 *In vitro* Binding Assay: Inhibition Curves of Compounds 9, 10, 11, 15 (Procedure 2), LUZ5, 17

6.1 Inhibition Curves of Binding Affinity Assays of Compounds 9, 10, 11, 15 (Procedure 2), LUZ5, 17 to CB_2R

Figure S84. Inhibition curve of compound **9** to CB_2R.

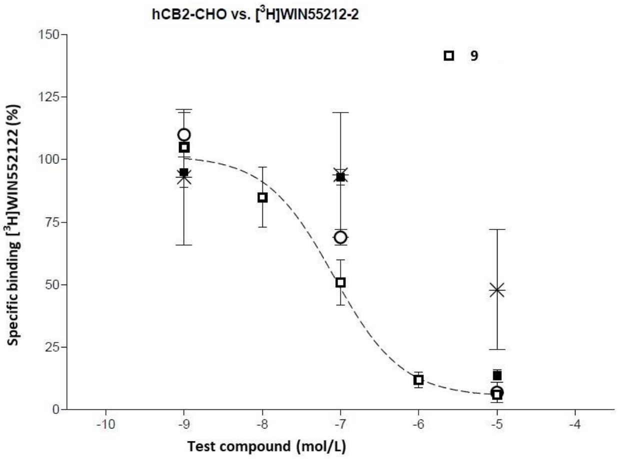

Supporting Information

Figure S85. Inhibition curves of compounds **15 (Procedure 2)** and **LUZ5** (**16**) to CB_2R.

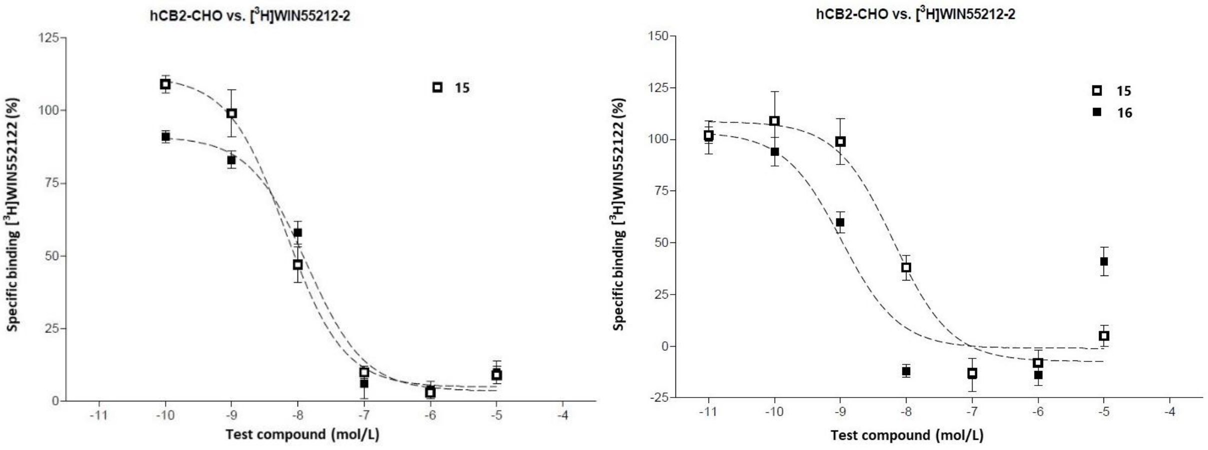

Figure S86. Inhibition curves of compounds **10** and **LUZ5** (**16**) to CB_2R.

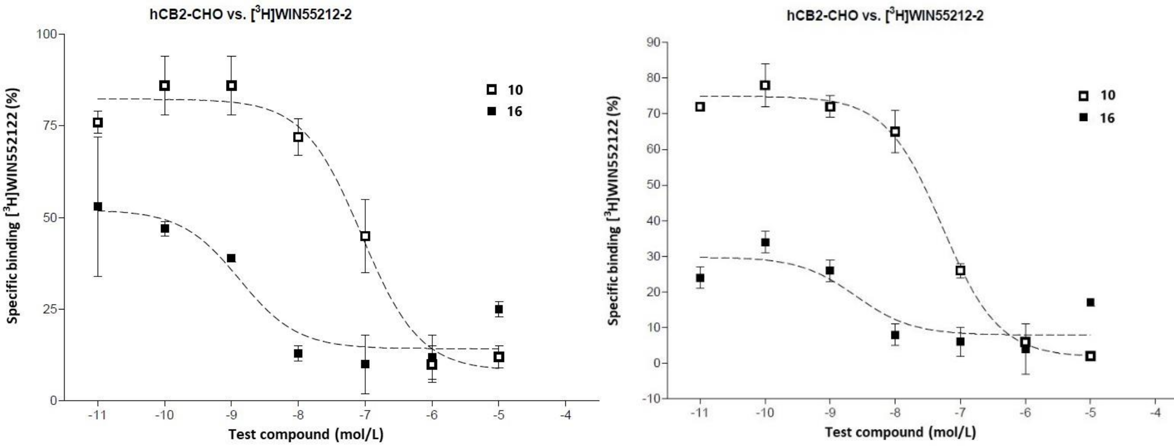

Figure S87. Inhibition curves of compounds **11** and **17** to CB_2R.

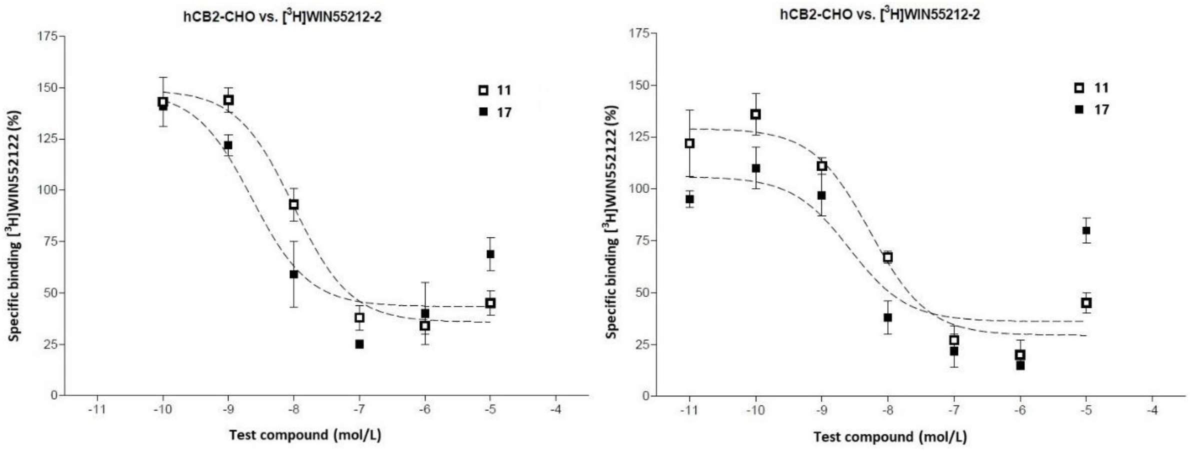

6.2 Inhibition Curves of Binding Affinity Assays of Compounds 9, 10, 11, 15 (Procedure 2), LUZ5, 17 to CB_1R

Figure S88. Inhibition curve of compound **LUZ5** (**16**) to CB_1R.

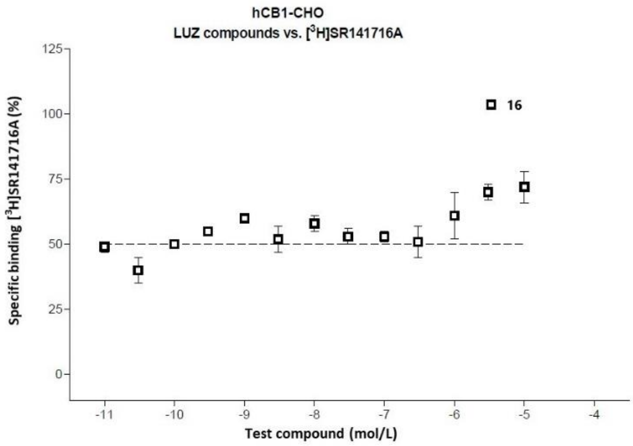

Figure S89. Inhibition curves of compounds **9**, **10**, **11**, **15 (Procedure 2)**, **LUZ5** (**16**), **17** to CB_1R.

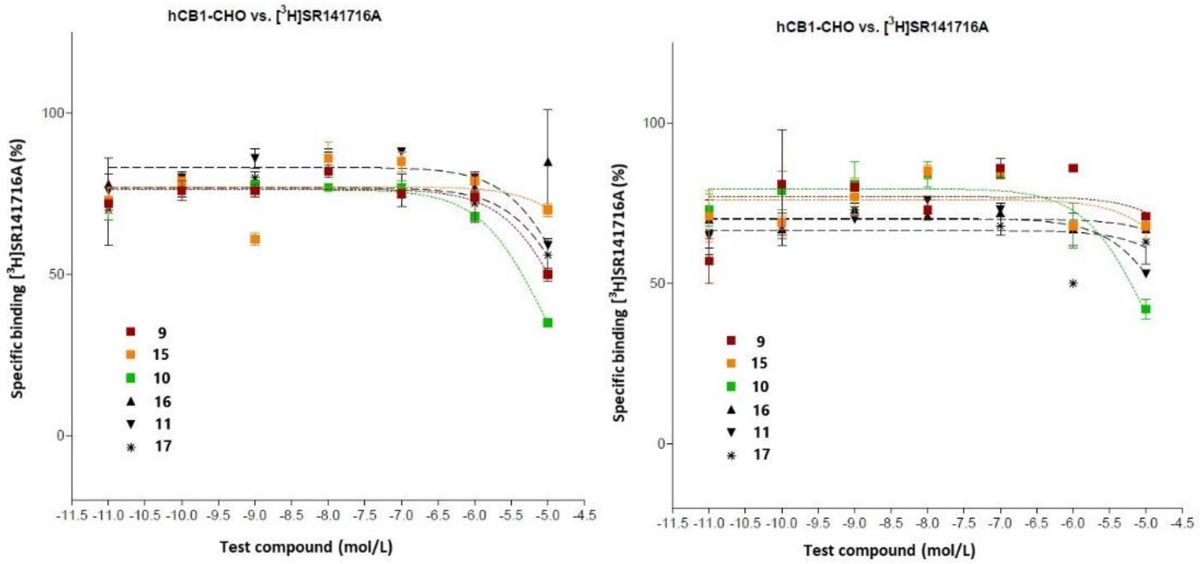

7 Docking Data of Compounds 9, 15 (Procedure 2), LUZ5

The crystal structure of the cannabinoid receptor type 2 (CB_2R) was prepared before the docking. The X-ray structure of the CB_2R is available from the protein data bank (PDB) under the code 5ZTY[6] and contains the synthetic ligand AM2051 in the binding pocket shown in Figure S90. The ligand was removed from the binding pocket and the receptor was protonated using Reduce program[7].

The molecular docking was performed with AutoDockTools4[8] software using the Lamarckian Genetic Algorithm[9]. The force-field parameters for boron atoms were added manually in the parameter file of the AutoDockTools4 library. The water molecules were eliminated, and the non-polar hydrogens were merged. The docking area was limited by the constructed grid box of the size 74 x 88 x 70 centered at 5.86, −4.65, −52.77 of x,y,z-coordinates (based on the position of the LBD). The following docking parameters were used in our docking: number of hybrid GA-LS runs: 500; population size: 150; maximum number of energy evaluations: 25000000, maximum number of top individuals to survive to next generation: 1; rate of gene mutation: 0.02; rate of crossover: 0.8; Mean of Cauchy distribution for gene mutation: 0.0; variance of Cauchy distribution for gene mutation: 1.0.

The binding energy (the energy scoring function), according to AutoDock, is estimated as the difference between the energies of the bound and unbound protein-compound system. It is the sum of the energetic terms dependent on intramolecular (inside the receptor and compounds) and intermolecular (between the receptor and compounds) interactions mentioned in the following equation[9]:

$$\Delta G = (\Delta G_{bound}^{L-L} - \Delta G_{unbound}^{L-L}) + (\Delta G_{bound}^{P-P} - \Delta G_{unbound}^{P-P}) + (\Delta G_{bound}^{P-L} - \Delta G_{unbound}^{P-L} + \Delta S_{conf}T)$$
$$= \Delta G_{vdW} + {}^{\circ}\Delta G_{elec} + {}^{\circ}\Delta G_{Hbond} + {}^{\circ}\Delta G_{desolv} + {}^{\circ}\Delta G_{torsional}$$

In this equation L and P are referred to the ligand (L, docked molecules) and protein (P, receptor). The other parameters are: ΔS_{conf} – conformational entropy which is lost upon binding, ΔG_{vdW} – energy of van der Waals interactions (estimated as a 6/12 potential), ΔG_{elec} – energy of electrostatic forces (estimated based on Coulomb forces), ΔG_{Hbond} – energy of the hydrogen bonds (estimated as a 10/12 potential), ΔG_{desolv} – desolvation energy and $\Delta G_{torsional}$ – torsional energy.

The next example of calculation is illustrated for compound **9**. The algorithm generated the conformational cluster of the structure **9**. The conformation with the lowest binding energy is stabilized by several energetic contributions according to eq. 1. For instance, the value of the sum for the best generated conformation ($\Delta G_{vdW} + {}^{\circ}\Delta G_{Hbond} + {}^{\circ}\Delta G_{desolv}$) is negative and made up -8.51 kcal mol^{-1}. ΔG_{elec} is equal to -0.04 kcal mol^{-1} and has no high stabilizing impact. However, $\Delta G_{torsional}$ is positive (+3.88 kcal/mol) and destabilizes the conformation. Therefore, the lowest binding energy in the cluster is:

$$\Delta G = -8.51 - 0.04 + 3.88 = -4.67\ \text{kcal mol}^{-1}$$

Subsequently, the algorithm calculated the mean binding energy in the cluster which made up -4.53 kcal mol^{-1}. The binding energies for compounds **15** and **LUZ5** were calculated accordingly.

The crucial influence of agonists/antagonists on the activity of CB_2R is based on the interactions of these molecules with amino acids in the binding pocket. In particular the pharmacological difference between CB_2R agonists and antagonists could be in the ability or inability to change the conformation of *tryptophane 258*[6,10] (toggle switch; Figure S90). The docking of the compounds **9**, **15** and **LUZ5** hypothetically estimates the possible conformations of the carborane analogues in the CB_2R binding pocket and their interactions with the amino acid residues. Interestingly, the possible alignment of the structure **9** among other compounds was similar to the reference molecule AM2051 (Figure S90,

A vs B). The alkyl 'arm' of **9** may interact with toggle switch W258 inducing the conformational changes (Figure S90, B). However, this compound exhibited the lowest affinity in comparison with the structures **15** and **LUZ5** (binding energy value of compound **9**: -4.53 kcal mol^{-1}) with the estimated inhibition constant K_i equal 375 µM. The best docked poses of **15** and **LUZ5** are displaced to the loop of the receptor, where the interactions of those compounds with H95, F94, F183 and F87 residues could probably induce the conformational changes of the loop (Figure S90, C and D). This could have a crucial impact on the CB_2R functions, which are dependent on the loop shape[11]. Additionally, the binding energy of **15** made up -10.87 kcal mol^{-1} with the predicted inhibition constant in nanomolar range (K_i = 6.37 nM). The interactions of compound **LUZ5** with the loop was insignificantly destabilized, demonstrating lower affinity with the slightly higher binding energy -9.68 kcal mol^{-1} (K_i = 80.80 nM).

Figure S90. *In silico* investigation of the binding modes based on docking of the compounds **9**, **15** and **LUZ5**. The molecular structure of the literature-known compound **AM2051** in the binding pocket of cannabinoid receptor type 2 (CB_2R) is shown in tan (**A**); the compounds violet **9** (**B**), blue **15** (**C**) and orange **LUZ5** (**D**) in the binding pocket of the receptor are shown as the best docked models. Amino acid residues participating in non-covalent interaction are shown. The toggle switch of the CB_2R activity is represented as the dark-blue sticks and balls. The crystallographic data of CB_2R as the starting point for docking was obtained from the Protein Data Bank (PDB ID: 5ZTY).

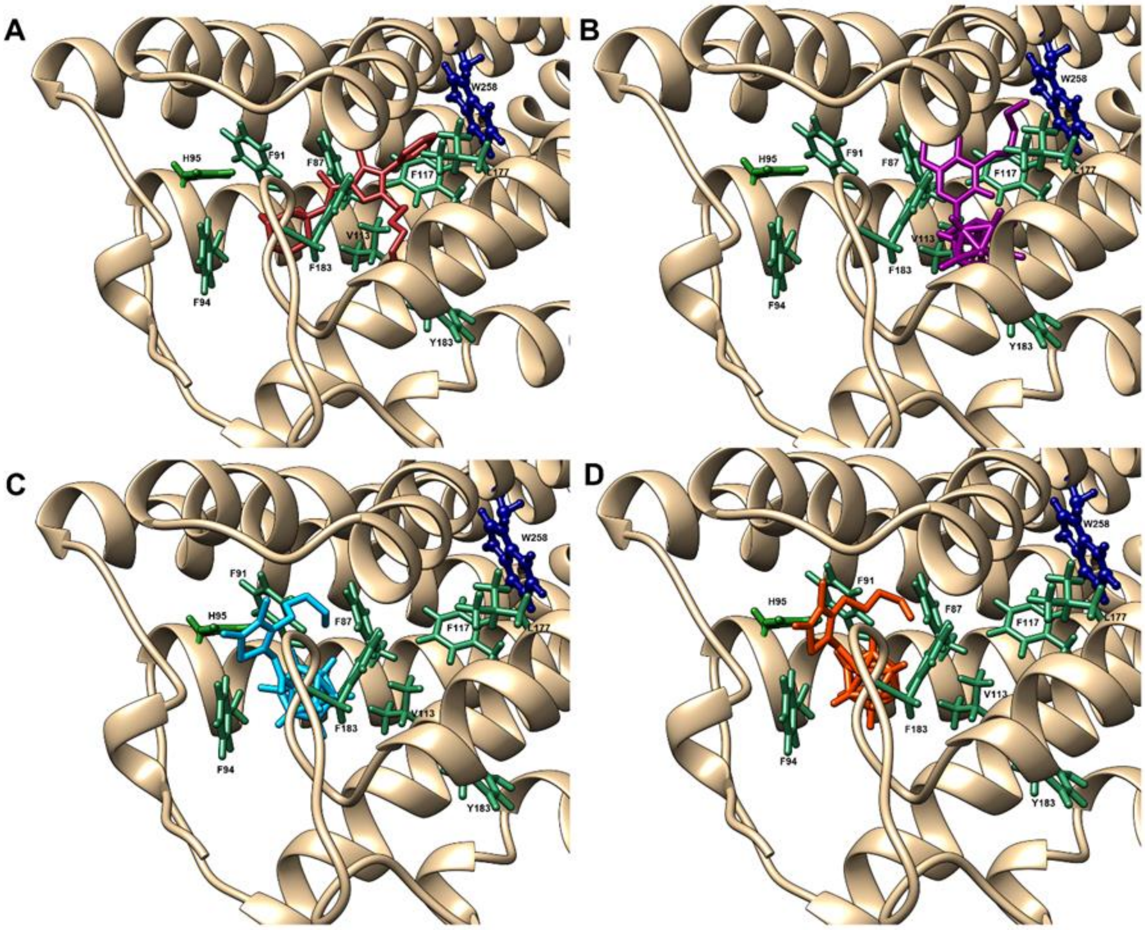

8 *In vitro* Metabolism Studies: LC-MS Exemplified Spectrum of LUZ5

Figure S91. Exemplified HPLC chromatogram (detection: 323 nm) of **LUZ5** after 60 min of incubation.

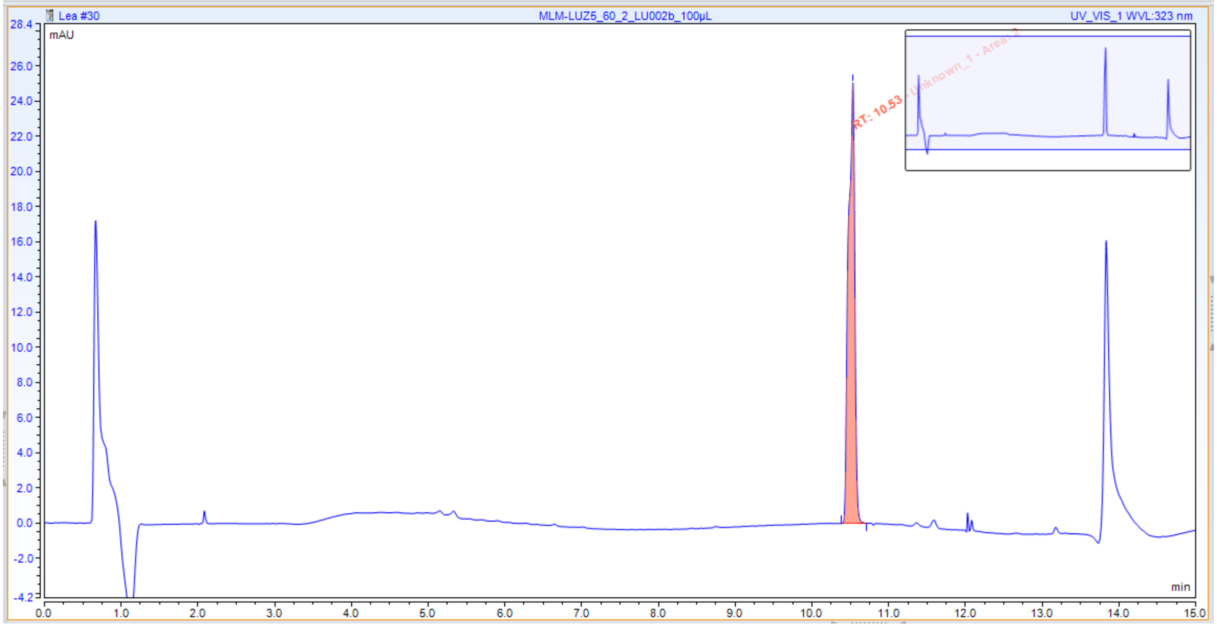

Figure S92. Exemplified mass spectrum of **LUZ5** after 60 min of incubation.

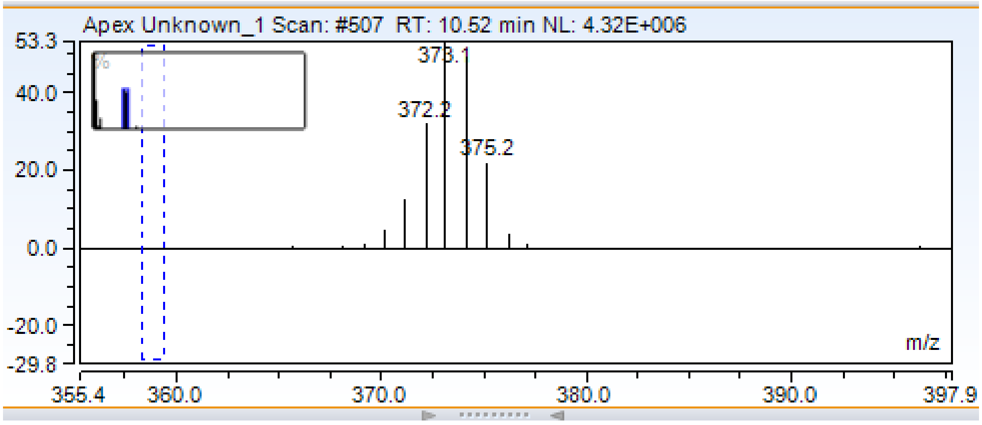

9 UV Profile of Isolated [^{18}F]LUZ5-d_8

Figure S93. Representative HPLC UV trace of a molar activity sample of **[^{18}F]LUZ5-d_8**. HPLC conditions: Reprosil-Pur C18-AQ column (250 × 4.6 mm, 5 µm), 66% CH_3CN/20 mM $NH_4OAc_{aq.}$, flow rate 1 mL/min, 312 nm.

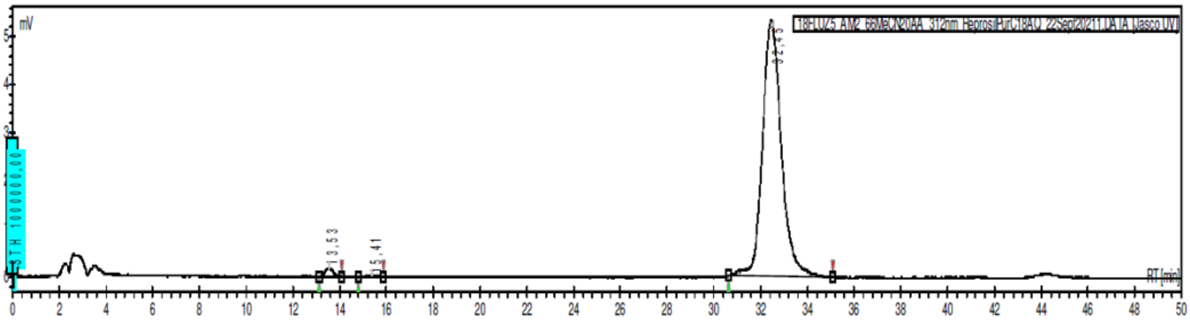

10 Assessment of the Biodistribution of [^{18}F]LUZ5-d_8 in Rodent Models

Figure S94. TACs of different tissues derived dynamic PET studies after intravenous injection of **[^{18}F]LUZ5-*d*$_8$** in male Wistar rats pre-treated with or without 1.5 mg/kg GW405833 injected 10 minutes prior to the radiotracer (n = 3) expressed in mean standardized uptake values (SUV$_{mean}$ ± SD), as well as the percentage of injected dose of the radiotracer (% ID) in the bladder.

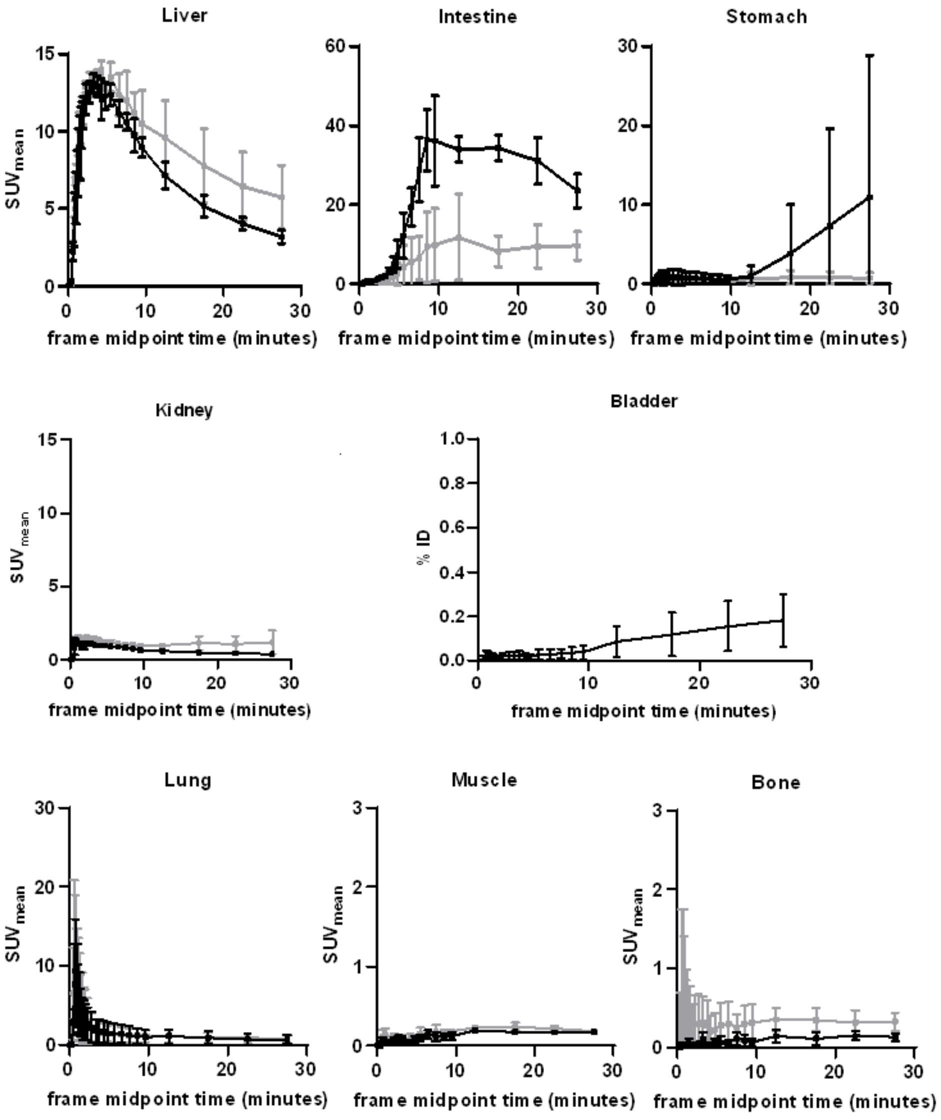

Figure S95. Biodistribution study of **[^{18}F]LUZ5-*d*$_8$** injected intravenously in a female CD1 mouse. A) Maximal intensity projections MIPs of MR (T1-weighted) and the dynamic whole body PET record presented in averaged time frames as indicated, as well as the merged images of MR and PET; B) Time activity curves (TACs) of the corresponding PET recording of the indicated tissues in mean standardized uptake values (SUV_{mean} ± SD) and the spleen uptake normalized to the blood pool activity SUVr(spleen-to ventricle), as well as the percentage of injected dose of the radiotracer (% ID) in the bladder.

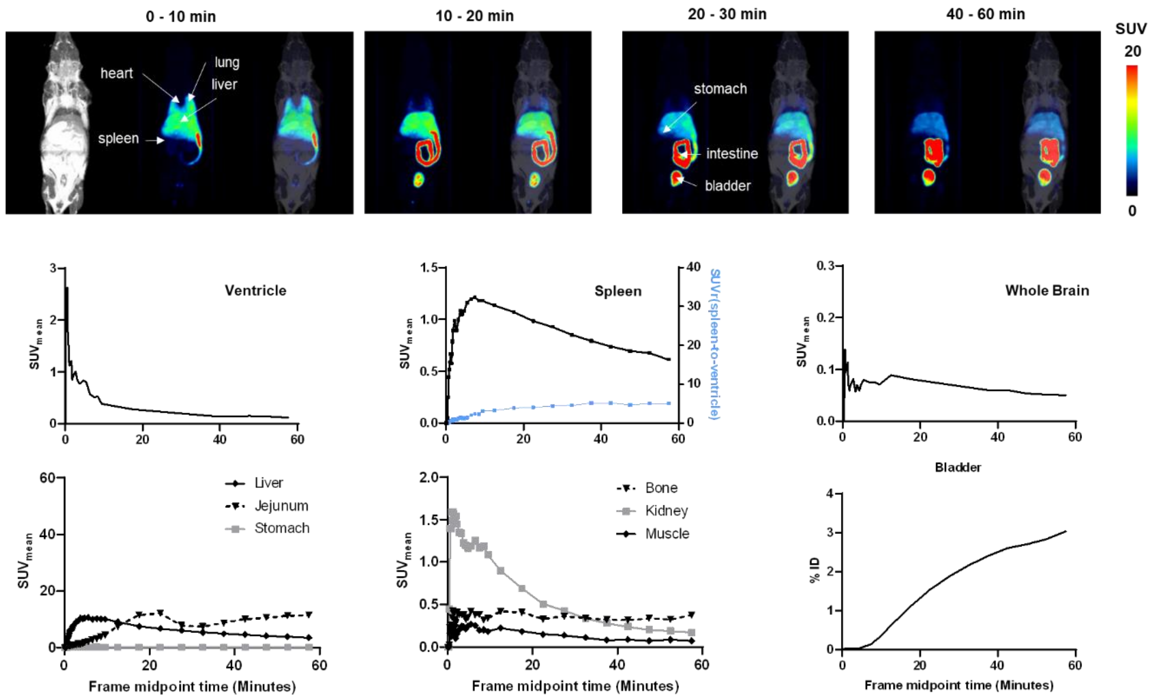

11 Chemical Structures of CB$_2$R Agonists and CB$_1$R Antagonist/Inverse Agonist Used in Biological Experiments

Figure S96. Chemical structures of CB$_2$R agonists: WIN55212-2, GW405883 and CB$_1$R antagonist/inverse agonist SR141716A.

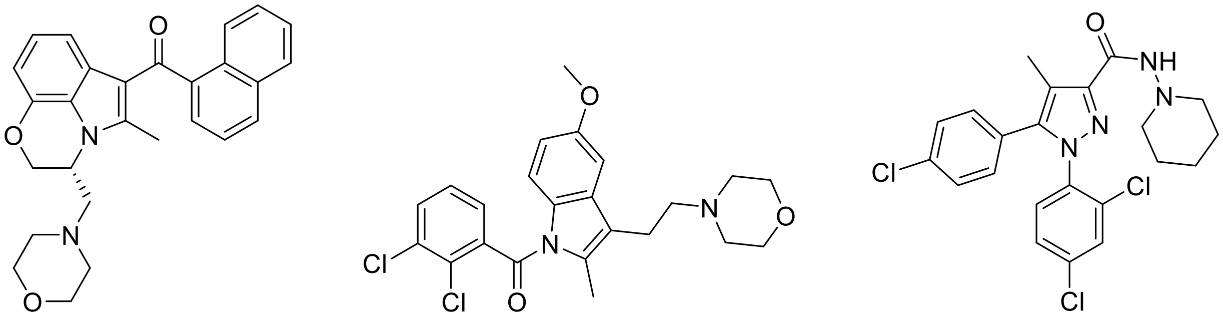

WIN55212-2 **GW405883** **SR141716A**

12 References

(1) *CrysAlis Pro*: *Data Collection and Data Reduction Software Package*; Rigaku Oxford Diffraction, Oxford, UK.

(2) *SCALE3 ABSPACK*: *Empirical Absorption Correction using Spherical Harmonics*.

(3) Sheldrick, G. M. SHELXT - integrated space-group and crystal-structure determination. *Acta Cryst. A* **2015**, *71*, 3–8. DOI: 10.1107/S2053273314026370.

(4) Sheldrick, G. M. Crystal structure refinement with SHELXL. *Acta Cryst. C* **2015**, *71*, 3–8. DOI: 10.1107/S2053229614024218.

(5) *Diamond*: *Crystal Impact*, 2014, Bonn, Germany.

(6) Bank, R. P. D. *RCSB PDB - 5ZTY: Crystal structure of human G protein coupled receptor*. https://www.rcsb.org/structure/5ZTY (accessed 2022-10-05).

(7) Word, J. M.; Lovell, S. C.; Richardson, J. S.; Richardson, D. C. Asparagine and Glutamine: Using Hydrogen Atom Contacts in the Choice of Side-Chain Amide Orientation. *J. Mol. Biol.* **1999**, *285*, 1735–1747. https://doi.org/10.1006/jmbi.1998.2401.

(8) Morris, G. M.; Huey, R.; Lindstrom, W.; Sanner, M. F.; Belew, R. K.; Goodsell, D. S.; Olson, A. J. AutoDock4 and AutoDockTools4: Automated Docking with Selective Receptor Flexibility. *J. Comput. Chem.* **2009**, *30*, 2785–2791. https://doi.org/10.1002/jcc.21256.

(9) Morris, G. M.; Goodsell, D. S.; Halliday, R. S.; Huey, R.; Hart, W. E.; Belew, R. K.; Olson, A. J. Automated Docking Using a Lamarckian Genetic Algorithm and an Empirical Binding Free Energy Function. *J. Comput. Chem.* **1998**, *19*, 1639–1662. https://doi.org/10.1002/(SICI)1096-987X(19981115)19:14<1639::AID-JCC10>3.0.CO;2-B.

(10) Lucchesi, V.; Hurst, D. P.; Shore, D. M.; Bertini, S.; Ehrmann, B. M.; Allarà, M.; Lawrence, L.; Ligresti, A.; Minutolo, F.; Saccomanni, G.; Sharir, H.; Macchia, M.; Di Marzo, V.; Abood, M. E.; Reggio, P. H.; Manera, C. CB2-Selective Cannabinoid Receptor Ligands: Synthesis, Pharmacological Evaluation, and Molecular Modeling Investigation of 1,8-Naphthyridin-2(1H)-One-3-Carboxamides. *J. Med. Chem.* **2014**, *57*, 8777–8791. https://doi.org/10.1021/jm500807e.

(11) Fiser, A.; Sali, A. ModLoop: Automated Modeling of Loops in Protein Structures. *Bioinformatics* **2003**, *19*, 2500–2501. https://doi.org/10.1093/bioinformatics/btg362.
